# Investigating 3,3-diaryloxetanes as potential bioisosteres through matched molecular pair analysis†

**DOI:** 10.1039/d1md00248a

**Published:** 2021-10-06

**Authors:** Maryne A. J. Dubois, Rosemary A. Croft, Yujie Ding, Chulho Choi, Dafydd R. Owen, James A. Bull, James J. Mousseau

**Affiliations:** Department of Chemistry, Molecular Sciences Research Hub, White City Campus, Imperial College London Wood Lane London W12 0BZ UK j.bull@imperial.ac.uk; Medicine Design, Pfizer Worldwide Research, Development and Medical 445 Eastern Point Rd. Groton CT 06340 USA james.mousseau@haldathera.com; Pfizer Medicine Design 610 Main St Cambridge MA 02139 USA

## Abstract

Oxetanes have received increasing interest in medicinal chemistry as attractive polar and low molecular weight motifs. The application of oxetanes as replacements for methylene, methyl, *gem*-dimethyl and carbonyl groups has been demonstrated to often improve chemical properties of target molecules for drug discovery purposes. The investigation of the properties of 3,3-diaryloxetanes, particularly of interest as a benzophenone replacement, remains largely unexplored. With recent synthetic advances in accessing this motif we studied the effects of 3,3-diaryloxetanes on the physicochemical properties of ‘drug-like’ molecules. Here, we describe our efforts in the design and synthesis of a range of drug-like compounds for matched molecular pair analysis to investigate the viability of the 3,3-diaryloxetane motif as a replacement group in drug discovery. We conclude that the properties of the diaryloxetanes and ketones are similar, and generally superior to related alkyl linkers, and that diaryloxetanes provide a potentially useful new design element.

## Abstract 2

Physical property and metabolic stability comparisons are presented for designed series of drug-like compounds, containing diaryloxetanes, ketones and alkyl linkers.

## Graphical abstract

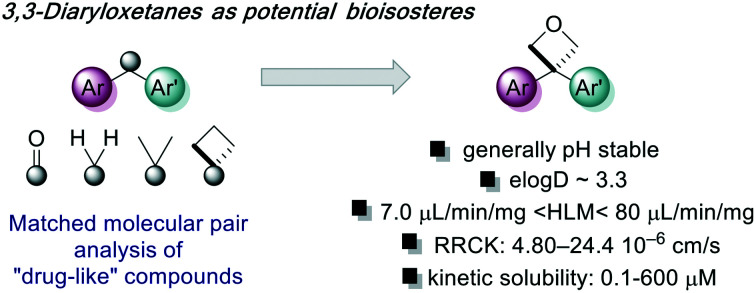

## Introduction

The application of bioisosteres to replace problematic functionality is a common strategy in medicinal chemistry. In doing so, the goal is often to maintain target activity and binding kinetics, while simultaneously improving aspects of physicochemical properties and/or improving toxicological profiles.^1^ Synthetic efforts continue to focus on providing new or improved access to novel isosteres,^2^ to both accelerate and improve design options in drug discovery.^3^ The subsequent understanding of the properties of new motifs as well as their effects in a drug discovery setting allows chemical synthesis to have a significant influence in driving novel analogue design. Notable examples include the adoption of sulfoximines^4^ and bicyclo[1.1.1]pentane motifs,^2*a*^ as well as oxetanes,^5,6^ each offering attractive physicochemical properties and increased 3-D character.

**Fig. 1:**
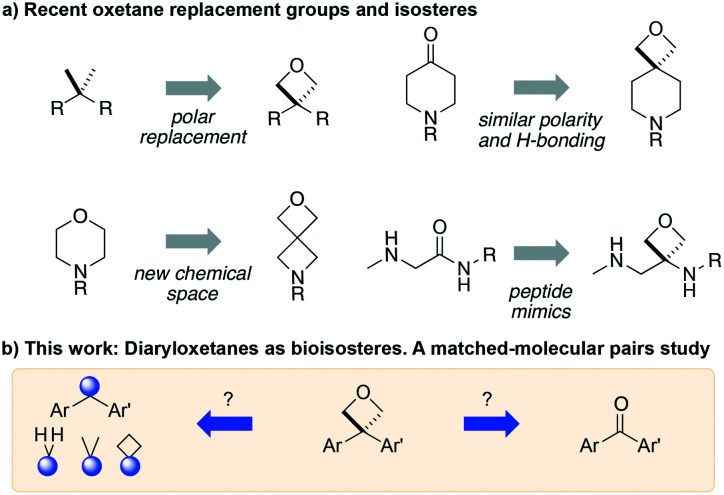
Developments of oxetane bioisosteres.

Studies on oxetanes in medicinal chemistry came to prominence with Carriera's work illustrating the potential of 3,3-disubstituted oxetanes as a replacement group for *gem*-dimethyl motifs, and as isosteres for cyclic ketones and morpholines, in spirocyclic examples (Fig. 1).^6^ The incorporation of this polar and low molecular weight moiety has been demonstrated to afford compounds with enhanced properties: improved metabolic stability, solubility and lipophilicity is often observed, while also increasing the sp^3^-content (Fsp^3^) of a target compound.^5–7^ The ring structure gives the endocyclic oxygen atom increased Lewis basicity and accessibility of the lone pairs, providing hydrogen bond acceptor capability comparable to that of ketones.^8^ Other recent reports include applications as peptidomimetics whereby the carbonyls of amide groups are replaced by 3-aminooxetanes,^9^ as well as oxetan-3-ol as an alternative isosteric replacement of carboxylic acid.^10,11^

3-Mono- and 2-substituted-oxetanes have also seen significant synthetic and medicinal investigation, and are increasingly investigated in SAR studies, and are present in bioactive compounds.^5,12,13^ However, 3,3-disubstituted derivatives have seen the most attention, as they benefit from not introducing a stereocenter, and increase steric protection to avoid ring opening pathways.

We became interested in 3,3-diaryloxetanes as potential bioisosteres for benzophenones that may offer improved properties (Fig. 1b). While benzophenones have been applied to ligate probes in chemical biology applications,^14^ this innate reactivity often makes this highly activated ketone undesirable in medicinal chemistry programs. They often act as photosensitizers, exhibiting phototoxicity through nucleobase modifications.^15^ At Pfizer, as a strategy to mitigate this phenomenon, several internal programs also described a need for 3,3-diaryloxetanes for key analogues. However, mining of internal data demonstrated that this architectural structure was often challenging to access, and as a result attempts towards these design targets were often halted. This was corroborated by a survey of the literature, whereby prior to early 2015, there were few viable synthetic methods reported towards 3,3-diaryloxetanes. Most relied on the low yielding ring closure of 1,3-diols,^16^ or a Paternò–Büchi cycloaddition, which gave undesired 2,2,3,3-tetrasubstituted oxetanes.^17^ Given the knowledge of our internal and external data, we initiated an industrial-academic collaboration between Pfizer and the Bull lab to discover new methods to form these aliphatic heterocycles. This partnership expeditiously developed a new way into 3,3-diaryloxetanes through a lithium-catalyzed Friedel–Crafts manifold, which provided unprecedented access to decorated 3,3-diaryloxetane motifs.^18^ Mechanistic understanding enabled the development of other interesting 3,3-disubstituted oxetanes.^11,19^ Furthermore, we have been able to demonstrate that 3,3-diaryloxetanes are robust to a range of chemical transformations, enabling access to a range of compounds with diverse functionality.

With these new methods in hand, and cognizant of the lack of understanding of the impact of 3,3-diaryloxetanes on physicochemical properties, we proceeded to a matched molecular pair analysis (MMPA) to probe the effect of replacing a benzophenone with a diaryloxetane on lipophilicity, cell permeability, clearance, solubility, and chemical stability, amongst others. In addition we rationalized that the oxetane could be an effective isostere for metabolically labile diarylmethane groups, as well as lipophilic *gem*-dimethyl, and cyclobutane derivatives. Herein we disclose our efforts in the preparation of these drug-like compounds as well as the analysis of the subsequent MMPA of physicochemical and biological properties of oxetane, ketone, and other linkers. The aim of this study is to assess the viability of the 3,3-diaryloxetane for putative application in lead optimization in medicinal chemistry settings.

## Results and discussion

**Fig. 2:**
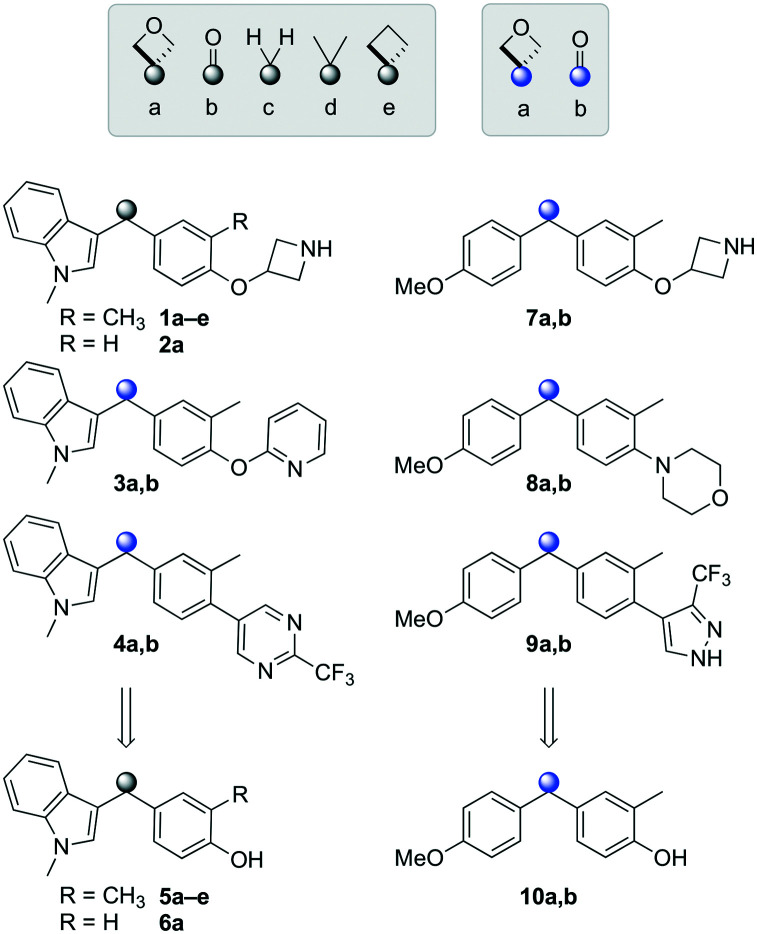
Compounds selected for the proposed pairwise analysis.

Initially, a series of >150 lead-like and drug-like compounds was designed targeting properties often desirable in a pharmaceutical setting.^20^ Their properties were predicted with Pfizer's proprietary methods, from which 16 compounds were selected for synthesis with a range of lipophilic values (clog *D* 0.5–5.0) as well as eight phenolic precursors to provide a handle for divergent synthesis. Two series were established: 1) the indole series including five linkers (**a**–**e**), and 2) the *p*-methoxyphenyl series that constituted oxetane-ketone (**a**,**b**) pairs (Fig. 2). This provided a comparison of compounds differing by a single structural modification either by the linker in the central carbon or by the R group.

**Scheme 1:**
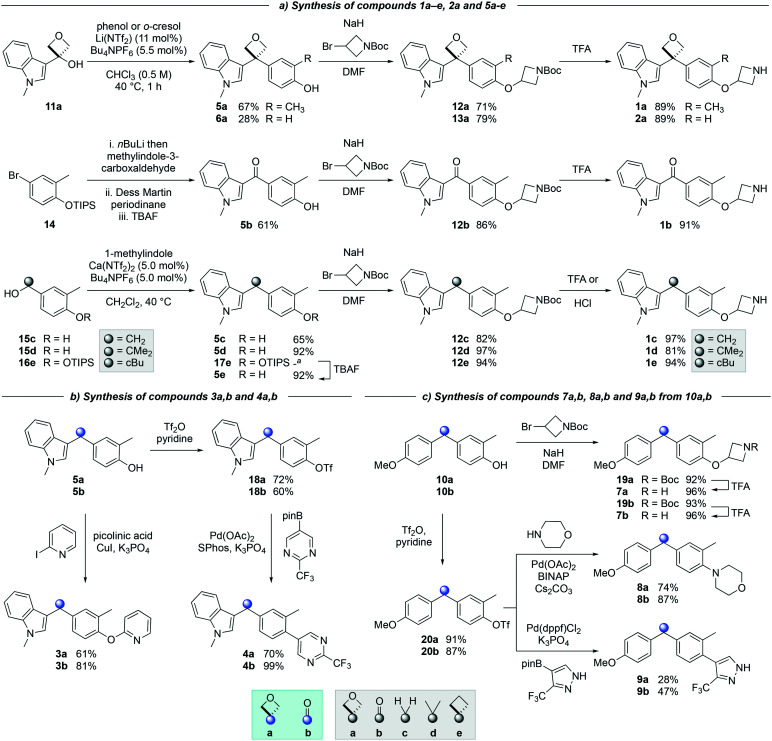
Synthesis of matched pairs. ^*a*^ Not isolated. Yield of 5e corresponds to yield over 2 steps.

The synthesis of targets 1a and 2a (Scheme 1a) was achieved *via* a catalytic Friedel–Crafts alkylation from a readily accessible alcohol precursor, a method developed in our previously disclosed work.^18^ 3-Indolyloxetanol 11a was furnished from commercially available oxetanone and 3-iodo-methylindole in the presence of *n*BuLi. Subjecting oxetanol 11a to a Friedel–Crafts alkylation using *o*-cresol or phenol and an inexpensive lithium triflimide catalyst under mild conditions led to diaryloxetane 5a in 67% yield, whereas oxetane 6a was obtained in 28%. This lower yield was due to the formation of oxetano-ether and dihydrobenzofuran side products consistent with previous work.^18,19*a*^

Treatment of arylbromide 14 with *n*BuLi and methylindol-3-carboxaldehyde resulted in an unstable alcohol, which after oxidation and deprotection steps gave desired ketone 5b in 61% yield over 3 steps (Scheme 1a). The synthesis of compounds 5c–5e was initially attempted using 3-indolylmethanols analogous to oxetanol 11a. However, in contrast to methylindolyl-oxetanol 11a, 3-indolylmethanols 11c and 11e (not shown) were unstable under acidic conditions when subjected to the Friedel–Crafts reaction. Dimerization, trimerization, and decomposition of the indolylmethanol precursors were observed (see ESI†).^21^ Alternatively, Friedel–Crafts alkylation of accessible alcohols 15c,d and 16e provided access to derivatives 5c–5e (Scheme 1a). Both methylene 5c and *gem*-dimethyl 5d derivatives were synthesized in good to excellent yields, and cyclobutane 5e was obtained in 92% yield after removal of the TIPS protecting group. Interestingly, the calcium triflimide catalyst was found to be more efficient for the synthesis of compounds 5c, 5d and 17e relative to the lithium triflimide catalyst which was optimized for the oxetane system.^22^

Alkylation of phenols 5a–5e and 6a with an *N*-Boc protected azetidine gave ethers 12a–12e and 13a in excellent yield from 71% to 97% (Scheme 1a). Of note, the use of trifluoroacetic acid (TFA) to deprotect the azetidine amines did not lead to ring opening or decomposition of the oxetane ring, delivering both products 1a and 2a in excellent yield, and providing further support to the robustness and chemical stability of the 3,3-diaryloxetane motif. Notably, this was in contrast to the *gem*-dimethyl (1d) and cyclobutane (1e) analogues, which were found to be unstable under these conditions. Instead, 1d and 1e were obtained using HCl in 81% and 94% yield respectively. The ketone (1b) and methylene (1c) derivatives were smoothly obtained using TFA. Compounds 1a–1e were made in 20–81% yield in three to five steps from the corresponding alcohol or bromoarene.

Copper-catalyzed Ullman couplings proceeded with both phenols 5a and 5b yielding oxetane 3a and ketone 3b in 61% and 81% yield respectively (Scheme 1b). The phenols were readily converted to the triflates under standard triflation conditions. Subjecting triflates 18a and 18b to Suzuki–Miyaura cross-coupling with a pyrimidine pinacol boronic ester gave both arylated products 4a and 4b in 70% and 99% yield.

Diaryloxetane 10a was formed using similar conditions as described for 5a, whereas ketone 10b was synthesized *via* the treatment of a Weinreb amide with an aryllithium (see ESI†). Then, the addition of the *N*-Boc-protected azetidine and subsequent Boc-deprotection was achieved successfully yielding both amines 7a/7b in 96% yield (Scheme 1c). From triflates 20a and 20b, Buchwald–Hartwig amination gave morpholine substituted oxetane 8a and ketone 8b in 74 and 87% yield respectively. Suzuki–Miyaura cross-coupling afforded biaryl 9a and 9b in 28% and 47% yield.

**Fig. 3:**
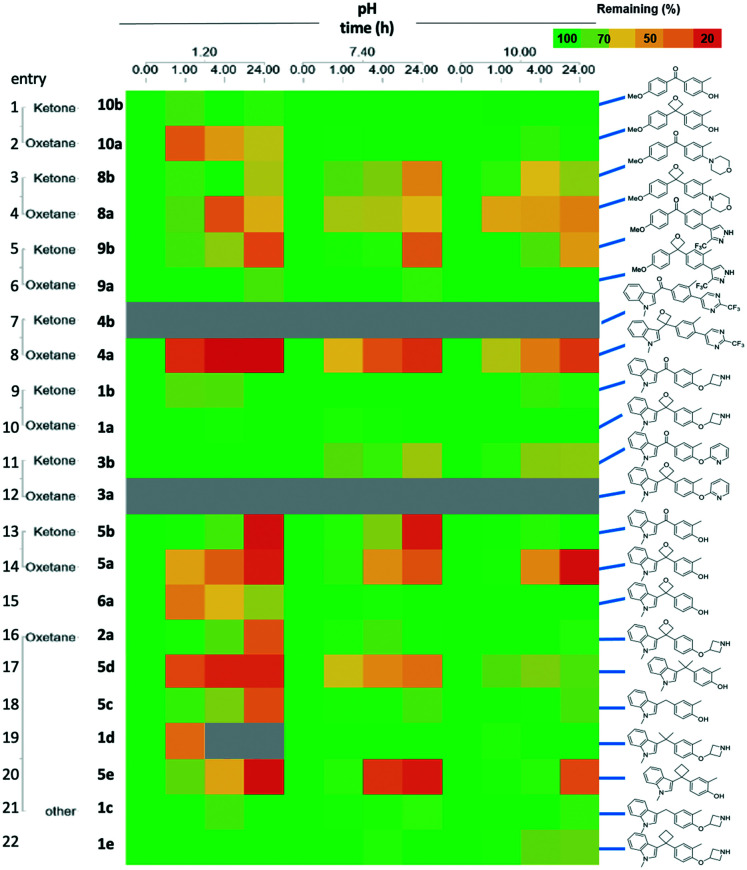
Stability of selected compounds indicating the % of compound remaining at different time points across at three different pH (1.2, 7.4, 10.0). Grey shading indicates data could not be obtained.

With the targets in hand we proceeded with our analysis. Given the common question that arises on the chemical stability of oxetanes, which could preclude application of these motifs in medicinal chemistry programs, we subjected the pairs to a pH stability assay. Fig. 3 displays selected pairs of ketone/oxetane, as well as the alkyl linkers.

We were pleased to observe that most 3,3-diaryloxetanes were tolerated at low pH, even after 4 h, as well as generally stable at higher pH. The stability of the ketones was similar to oxetanes. While the indole phenol oxetane and ketone 5a and 5b did demonstrate poorer stability at low pH (rows 13, 14), this was on par with the corresponding *gem*-dimethyl (row 17), methylene (row 18) and cyclobutyl (row 20) derivatives, suggesting that the cause of the instability is the designed chemotype and not the linking functional group. Interestingly, removal of the *o-*methyl group on the phenol improved the stability (row 15).

**Fig. 4:**
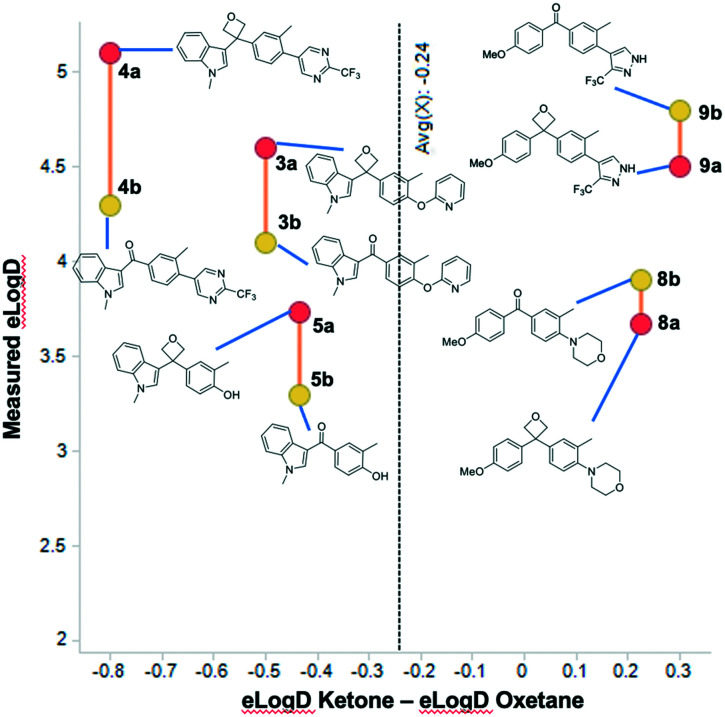
log *D* shift of selected compounds indicating the difference between oxetane (red circles) and ketone (yellow circle).

The measured lipophilicity of the oxetane compounds (elog *D*, pH 7.4) was across a range from 1.7–5.1, with an average of 3.4 over 10 compounds, and similar to the calculated values (see ESI† for full details, as well as sflog *D* data). It was clearly notable that the oxetane led to a significant decrease in measured elog *D* (0.81 units in elog *D* on average) in comparison to each of the methylene, *gem*-dimethyl, or cyclobutyl analogues, attributable to the polarity of the oxetane. Of particular interest was the effect relative to the corresponding ketone, and we compared five matched pairs. Previous work with other 3,3-disubstituted oxetanes suggested an increase in lipophilicity of 0.1 to 0.7 log *D* units.^6*b*^ Interestingly, the indole series indicated an average increase of 0.58 log units moving from the ketone to the oxetane (Fig. 4). However, the *p*-methoxyphenyl series exhibited the opposite trend, whereby the lipophilicity was decreased by 0.30 log units upon incorporation of the oxetane. Although the data disclosed herein suggests that the phenomenon will be series dependent, the combined values infer a similar trend to that reported by Carreira and others with an expected slight log *D* increase for the oxetane in comparison to the ketone (0.24 elog *D* units). It is interesting to observe that the trends were similar for measured log *D* (elog *D* and sflog *D*), whereas calculated clog *P* values were routinely higher for the ketone derivatives than the oxetanes (see Table S1†). An analysis of H-bonding potential may provide further insight but was outside of the scope of this study and was not explicitly assessed.^23^

Clearance by human liver microsomes was generally low to acceptable across the selection of compounds, with the exception of high clearance noted with the phenolic variants. In fact, with the five pairs for which data could be obtained, clearance by human liver microsomes were unchanged between compounds bearing the carbonyl and oxetane groups (Table 1).

**Table 1:** Clearance by human liver microsomes (HLM) of oxetanes *vs.* ketones

Oxetane	HLM Clint (μL min^−1^ mg^−1^)	Ketone	HLM Clint (μL min^−1^ mg^−1^)
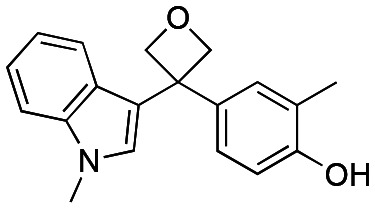	22.9	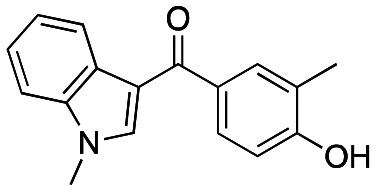	18
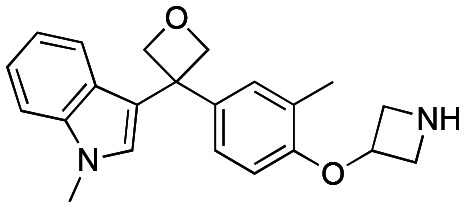	<7	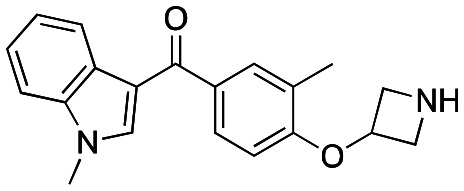	<7
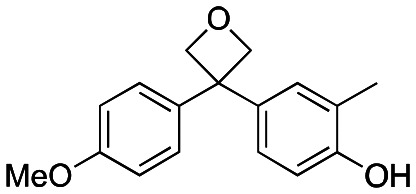	80	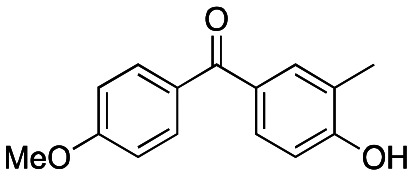	77.4
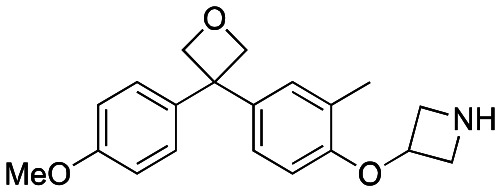	7.5	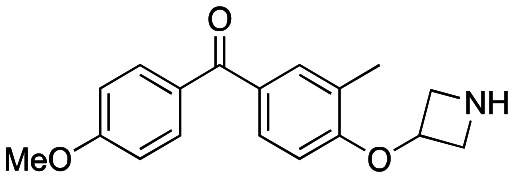	75
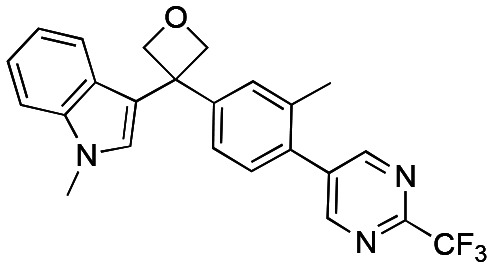	8.5	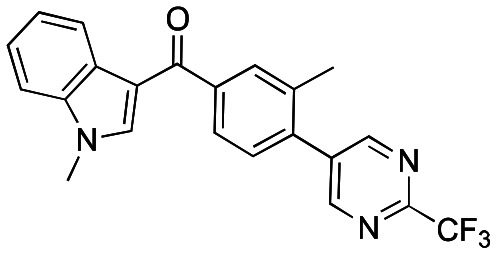	<7

**Fig. 5:**
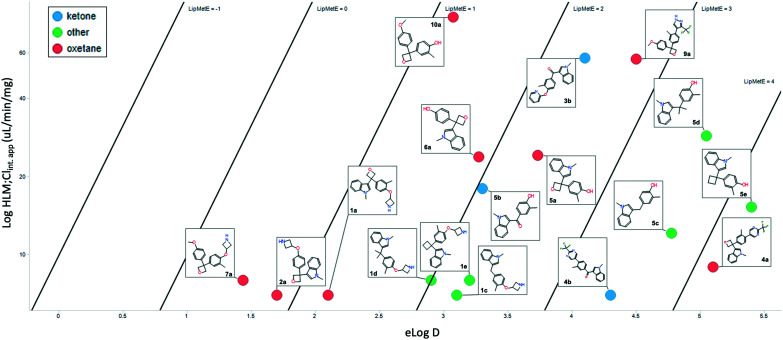
Lipophilic metabolism efficiency (LipMetE) analysis.

Cognizant that the clearance could be driven by log *D*, and given the similarity of those values between the pairs, a lipophilic metabolism efficiency (LipMetE) analysis was performed. LipMetE is an approach that was put forth in 2013 to describe the efficiency of a compound's metabolic stability relative to its lipophilicity.^12*b*^ Seventeen compounds where metabolism and log *D* values could be obtained were plotted (Fig. 5). Of the few values obtained with ketones, the LipMetE profile was slightly decreased relative to the oxetane. This could have been expected given the similarity in clearance, and in certain cases, lower log *D* of the ketones. The observation of note was the comparison of oxetanes relative to other linker groups. The majority of the compounds exhibited a LipMetE of >1, suggesting that clearance as a function of log *D* was favorable for most linkers. The two compounds with values <1, while oxetanes, demonstrated low clearance values with acceptable log *D* values. The absolute clearance values for some of the non-oxygenated linkers is modest relative to their corresponding log *D*. This potentially surprising observation is valuable for medicinal chemists as it suggests the possibility of the introduction of ‘friendly grease’. It is important to note that there does not appear to be a metabolic liability associated with the 3,3-diaryloxetane motif, and there is an improvement in LipMetE moving from the ketone to the oxetane.

**Fig. 6:**
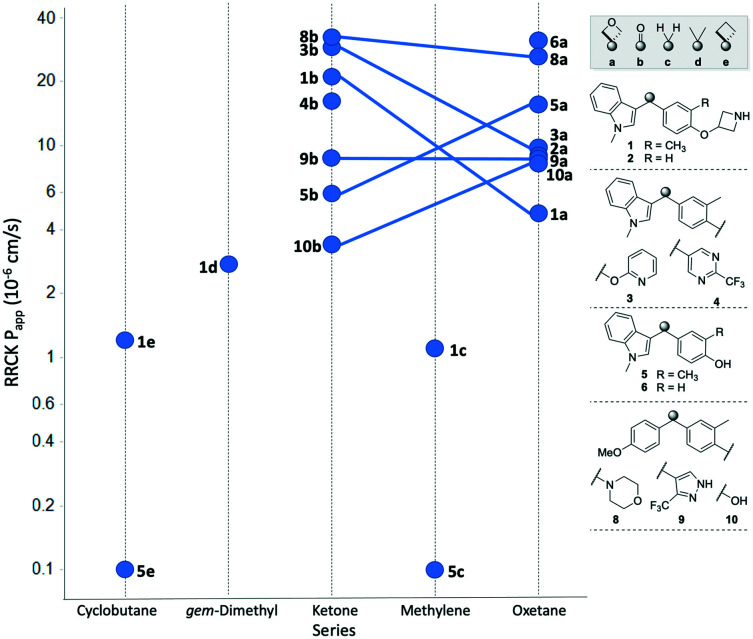
Comparison of cell permeability across the different linker types. Oxetane/ketone pairs indicated by linking line.

The permeability of the 3,3-diaryloxetanes was universally improved relative to the corresponding methylene, *gem*-dimethyl and cyclobutyl variants (Fig. 6).

For example, both the methylene and cyclobutyl indole-cresol derivatives (5c and 5e) were determined to exhibited very poor cell permeability with an RRCK of 0.1 × 10^−6^ cm s^−1^. When replacing the linker with an oxetane the permeability improved to a very respectable 15.6 × 10^−6^ cm s^−1^. The conformational constraints between the cyclobutyl and oxetanyl variants would be expected to be similar, and hence the improvement is due to the additional oxygen atom. The ketones were overall found to behave similarly to the oxetanes in cell permeability, with no clear trend when comparing the ketone and oxetanes. In three oxetane/ketone pairs the permeability decreased moving from ketone to oxetane, one pair was unchanged, and two pairs demonstrated improved permeability with the oxetane. This data suggests that the inclusion of an 3,3-diaryloxetane can be a tactic to improve cell permeability relative to the comparable all-carbon derivatives, but does not necessarily equate to an improvement relative to the corresponding benzophenone.

**Fig. 7:**
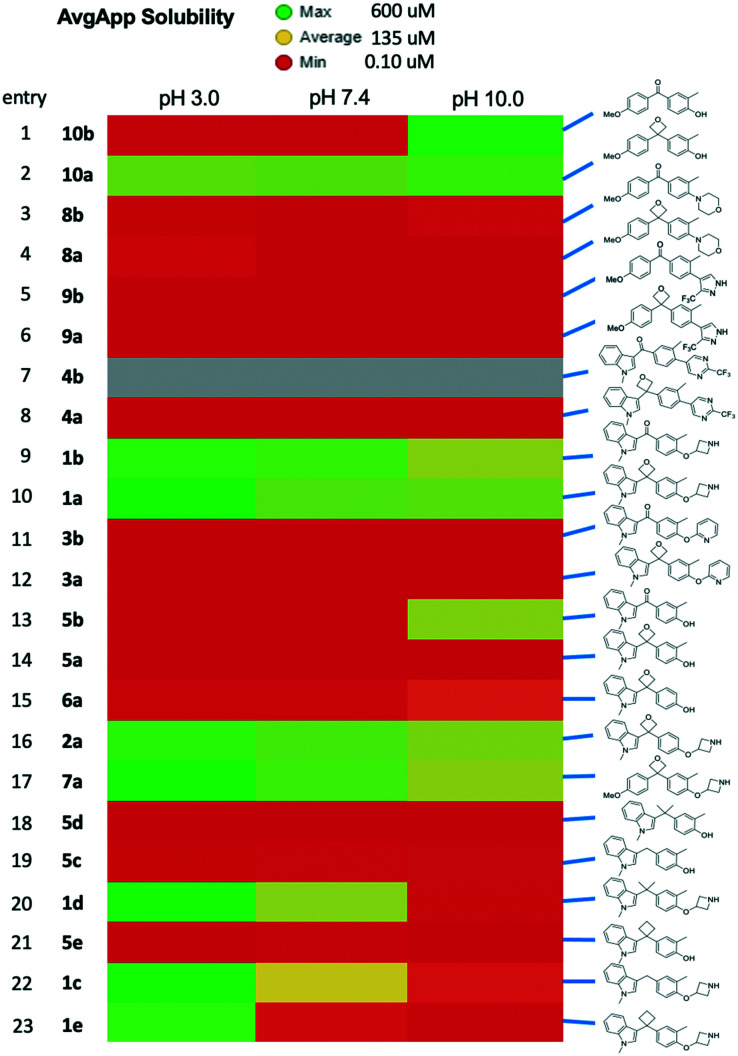
Comparison of solubility. Kinetic solubility at 3 pH values with color indicating solubility range as indicated.

As our last parameter we considered the kinetic solubility of the prepared compounds. The solubility varied significantly based on peripheral structure.^24^ Comparison of the obtained results with the 3,3-diaryloxetanes and the corresponding ketones indicates that the solubility was largely unchanged by the inclusion of the oxetane. While some oxetanes did display improved profiles relative to the their isosteric counterparts (Fig. 7, rows 1/2, 9/10, 16), the solubility of the molecules appear to be governed primarily by the aryl components, and as such in these architectures, oxetanes cannot be guaranteed to improve a low solubility profile.

## Conclusions

In summary, we have conducted the first study of the property space of 3,3-diaryloxetanes and related compounds in drug-like space, with direct comparisons to ketone and alkyl variants (methylene, *gem*-dimethyl, and cyclobutyl derivatives). We observed that the oxetane compounds are chemically stable to a range of pH and are no more prone to decomposition than other linker motifs. Indeed the oxetane examples showed advantageous stability within the synthetic sequences. The change in lipophilicity profile between oxetane and ketone was not uniform, as certain motifs displayed an increase in log *D* while others were lowered. The log *D* of the oxetane derivatives was significantly improved in comparison to the carbon-linkers. There was little change in metabolic stability when switching from a ketone to an oxetane, however improved profiles were observed relative to other isosteric equivalents. Cell permeability is markedly improved by changing a methylene, *gem*-dimethyl or cyclobutane to an oxetane or ketone, however, as with the log *D*, improvements from ketone to oxetane vary on a case by case basis. Lastly, the inclusion of an oxetane did not reliably improve solubility compared to ketones in these examined series.

Overall, the 3,3-diaryloxetanes displayed similar physicochemical properties to diaryl ketones, without the electrophilic and potential photochemical liabilities, and overall improved properties compared to methylene, *gem*-dimethyl and cyclobutane analogues. We have also further demonstrated the potential of the catalytic Friedel–Crafts methodology from 3-aryloxetan-3-ols to form 3,3-diaryloxetanes in compounds with drug-like and lead-like properties. We conclude that the diaryloxetane motif may provide a valuable replacement for diarylketones, as well as for other diarylmethane motifs. Furthermore, we propose that the diaryloxetane unit itself presents an attractive motif in underexplored chemical space that may find broader application in drug discovery as a new analogue design option without intrinsic liabilities.

## Conflicts of interest

There are no conflicts to declare.

## Supplementary Material

MD-012-D1MD00248A-s001
