# Supplementary material for: Investigating 3,3-diaryloxetanes as potential bioisosteres through matched molecular pair analysis
Source: RSC Med Chem. 2021 Oct 6;12(12):2045–52. doi: 10.1039/d1md00248a (PMC8672821; doi:10.1039/d1md00248a)
Supplement: MD-012-D1MD00248A-s001 [file MD-012-D1MD00248A-s001.pdf]

Electronic Supplementary Material (ESI) for RSC Medicinal Chemistry.
This journal is © The Royal Society of Chemistry 2021

SUPPORTING INFORMATION

Investigating 3,3-Diaryl Oxetanes as Potential Bioisosteres Through Matched Molecular Pair Analysis

Maryne A. J. Dubois,[a] Rosemary A. Croft,[a] Yujie Ding,[a] Chulho Choi,[b] Dafydd R. Owen,[c] James A. Bull[a] and James J. Mousseau[b]

[a] Department of Chemistry, Imperial College London, Molecular Sciences Research Hub, White City Campus, Wood Lane W12 0BZ, UK
[b] Medicine Design, Pfizer Worldwide Research, Development and Medical, 445 Eastern Point Rd., Groton, CT 06340, USA
[c] Pfizer Medicine Design, 610 Main St, Cambridge, MA 02139, USA

Table of Contents

Table of contents S1
General experimental considerations S2
Synthesis of compounds **1a–1e and 2a** S3
- Synthesis of 3,3-diaryloxetanes **1a** and **2a** S3
- Synthesis of diarylketone **1b** S7
- Synthesis of diarylmethane **1c** S10
- Synthesis of *gem*-dimethyl **1d** S12
- Synthesis of cyclobutane **1e** S14

Synthesis of 3,3-diaryloxetanes and diarylketones **3a–4a** and **3b-4b** S17
Synthesis of 3,3-diaryloxetanes **7a–10a** S21
Synthesis of diarylketones **7b–10b** S25
Synthesis and reactivity of 3-indolylmethanols **11c** and **11e** S29
ADMET S31
Table S1: cLogP, elogD and *sf*LogD of compound **1–10** S33
^{1}H and ^{13}C NMR Spectra for selected compounds S36
References S86

General Experimental Considerations

All non-aqueous reactions were carried out under an inert atmosphere (argon) with flame-dried glassware, using standard techniques, unless specified. Friedel–Crafts reactions were carried out under inert atmosphere with flame-dried glassware, using standard techniques, using non-dried CH_2Cl_2, $CHCl_3$, and toluene. Anhydrous solvents were obtained by filtration through drying columns (THF, DMF, EtOH, MeCN) or used as supplied (1,4-dioxane, DME, CH_2Cl_2). Flash column chromatography was performed using 230-400 mesh silica, with the indicated solvent system according to standard techniques. Analytical thin-layer chromatography (TLC) was performed on precoated glass-backed silica gel plates. Visualisation of the developed chromatogram was performed by UV absorbance (254 nm) and stained with aqueous potassium permanganate solution, phosphomolybdic acid solution, *para*-anisaldehyde solution or ninhydrin solution in ethanol. Infrared spectra (νmax, FTIR ATR) were recorded in reciprocal centimetres (cm^{-1}). Nuclear magnetic resonance spectra were recorded on 400 or 500 MHz spectrometers. Chemical shifts for ^1H NMR spectra are recorded in parts per million (ppm) from tetramethylsilane with the solvent resonance as the internal standard ($CDCl_3$: δ = 7.27 ppm, DMSO-d_6: δ = 2.50 ppm, MeOD-d_4: δ = 3.35 ppm, acetone-d_6: δ = 2.05 ppm). Data is reported as follows: chemical shift (multiplicity [s = singlet, d = doublet, t = triplet, q = quartet, p = pentet, h = heptet, m = multiplet and br = broad], coupling constant (in Hz), integration and assignment). ^{13}C NMR spectra were recorded with complete proton decoupling. Chemical shifts are reported in parts per million from tetramethylsilane with the solvent resonance as the internal standard ($^{13}CDCl_3$: δ = 77.0 ppm, $(^{13}CD_3)_2SO$: δ = 39.5 ppm, $^{13}CD_3OD$: δ = 49.0 ppm, $^{13}(CD_3)_2CO$: δ= 29.8 ppm). Assignments of ^1H and ^{13}C spectra were based upon the analysis of δ and *J* values, as well as DEPT, COSY, HSQC and HMBC experiments where appropriate. ^{19}F spectra were recorded with or without complete proton decoupling. Decoupling is indicated as $^{19}F\{^1H\}$ where relevant this is stated in each assignment. ^{19}F NMR spectra are indirectly referenced to $CFCl_3$ automatically *via* direct measurement of the absolute frequency of the deuterium lock signal by the spectrometer hardware. Melting points were recorded using Optimelt MPA100 apparatus and are uncorrected. The high resolution mass spectrometry (HRMS) analyses were performed using electrospray ion source (ESI), atmospheric pressure chemical ionisation (APCI), nanospray ionisation (NSI), or electron impact ionisation (EI). ESI was performed using a Waters LCT Premier equipped with an ESI source operated in positive or negative ion mode. In most cases the software used was MassLynx 4.1. This software does not account for the electron for +ESI and the calibrations/references are calculated accordingly, i.e. $[M+H]^+$ is detected and the mass is calibrated to output [M+H].

Reagents: Commercial reagents were used as supplied, or purified by standard techniques where necessary. Catalysts were purchased from the following suppliers, stored in a desiccator and used without further purification.

- Iron(III) chloride (98%, CAS: 7705-08-0) was purchased from Acros Organics, and stored under argon atmosphere.
- Bis(trifluoromethane)sulfonimide lithium salt (>99%, CAS: 90076-65-6) was purchased from Sigma-Aldrich.
- Calcium(II) bis(trifluoromethanesulfonyl)imide (> 97%, CAS: 165324-09-4) was purchased from TCI chemicals.

Procedure for purification with SCX® Column: SCX Cartridge 5 g/25 mL were purchased from ThermoScientific. The column was washed with three volumes of methanol. The sample was then loaded onto the column in a minimum amount of solvent (1:1 MeOH/DCM). The column was washed through with three column volumes of MeOH and then the free amine was eluted with three volumes of 5% NH_3 (28% aqueous solution)/MeOH.

Compounds purity: the purity of the compounds synthesised was judged by ^1H NMR and was at least of 90% unless otherwise stated.

Synthesis of compounds 1a–1e and 2a

Synthesis of 3,3-diaryloxetanes 1a–2a

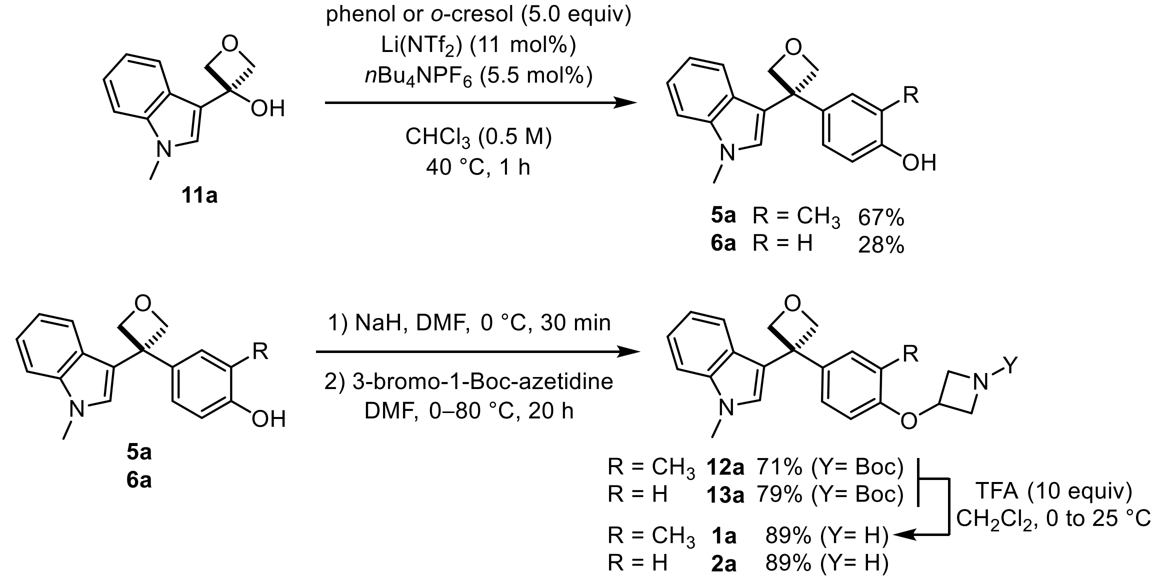

Figure S1.

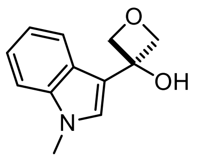

3-(1-Methyl-1*H*-indol-3-yl)oxetan-3-ol (11a)

*n*BuLi (1.47 M in hexane, 6.50 mL, 9.54 mmol) was added dropwise over 5 min to a solution of 3-iodo-1-methyl-1*H*-indole[1] (2.68 g, 10.4 mmol) in THF (21 mL) at –78 °C. The reaction mixture was stirred at –78 °C for 40 min. Oxetan-3-one (556 µL, 8.67 mmol) was added dropwise to the reaction mixture. Following a further 30 min at –78 °C the reaction mixture was warmed to 25 °C for 17 h then quenched with water (80 mL). Et_2O (80 mL) was added and the layers were separated. The aqueous portion was extracted with Et_2O (2 × 80 mL). The organic extracts were combined, dried over Na_2SO_4, filtered and concentrated under reduced pressure. Purification by flash column chromatography (50 to 100% Et_2O/pentane) afforded oxetanol **11a** (1.35 g, 77%) as a reddish oil. R_f = 0.15 (50% Et_2O/pentane); IR (film)/cm^{-1} 3365 (br, OH), 2948, 2873, 1728, 1553, 1229, 967, 738; ^{1}H NMR (400 MHz, $CDCl_3$) δ 7.78–7.76 (m, 1 H, $Ar_{(indole)}$-CH), 7.37–7.35 (m, 1 H, $Ar_{(indole)}$-CH), 7.30 (ddd, *J* = 8.2, 7.0, 1.1 Hz, 1 H, $Ar_{(indole)}$-CH), 7.18 (ddd, *J* = 8.2, 7.0, 1.1 Hz, 1 H, $Ar_{(indole)}$-CH), 7.13 (s, 1 H, N-Ar-CH), 5.07 (d, *J* = 7.1 Hz, 2 H, C*H*HOC*H*H), 5.03 (d, *J* = 7.1 Hz, 2 H, CH*H*OCH*H*), 3.81 (s, 3 H, NCH_3), 2.51 (s, 1 H, OH); ^{13}C NMR (101 MHz, $CDCl_3$) δ 137.6 ($Ar_{(indole)}$-C_q), 125.9 (N-Ar-CH), 125.3 ($Ar_{(indole)}$-C_q), 122.2 ($Ar_{(indole)}$-CH), 119.7 ($Ar_{(indole)}$-CH), 119.6 ($Ar_{(indole)}$-CH), 116.3 (N-Ar-CH-C_q), 109.6 ($Ar_{(indole)}$-CH), 84.8 (CH_2OCH_2), 72.9 (C_q), 32.7 (NCH_3); HRMS (+NSI) *m/z* calcd for $C_{12}H_{14}O_2N^+$ $[M+H]^+$: 204.1019, Found: 204.1019.

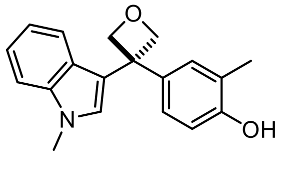

4-[3-(1-Methyl-1*H*-indol-3-yl)oxetan-3-yl]-2-methylphenol (5a)

Lithium bis(trifluoromethane)sulfonimide (80.9 mg, 0.28 mmol) and tetrabutylammonium hexafluorophosphate (64.8 mg, 0.17 mmol) were added to a solution of oxetanol **11a** (619 mg, 3.04 mmol) and 2-methylphenol (1.60 mL, 15.2 mmol) in $CHCl_3$ (6.1 mL). The reaction mixture was stirred at 40 °C for 1 h then sat. aq. $NaHCO_3$ (50 mL) was added followed by EtOAc (50 mL). The layers were separated and the aqueous portion was extracted with EtOAc (3 × 50 mL). The organic extracts were combined, dried over Na_2SO_4, filtered and concentrated under reduced pressure. Purification by flash column chromatography (30% Et_2O/pentane) afforded oxetane **5a** (594 mg, 67%) as a yellow solid. R_f = 0.15 (25% EtOAc/pentane); mp = 233–235 °C; IR (film)/cm^{-1} 3226 (br, OH), 2957, 1611, 1514, 1483, 1462, 1373,

1330, 1269, 1214, 1125, 967, 768, 734; ^{1}H NMR (400 MHz, DMSO-d_6) δ 9.18 (s, 1 H, OH), 7.40 (d, *J* = 8.5 Hz, 2 H, 2 × $Ar_{(indole)}$-CH), 7.16–7.12 (m, 1 H, $Ar_{(indole)}$-CH), 7.05 (d, *J* = 2.1 Hz, 1 H, Ar-CH), 7.00 (s, 1 H, N-Ar-CH), 6.98–6.91 (m, 2 H, $Ar_{(indole)}$-CH, Ar-CH), 6.72 (d, *J* = 8.3 Hz, 1 H, Ar-CH), 5.14–5.11 (m, 4 H, C*H_2*OC*H_2*), 3.73 (s, 3 H, NCH_3), 2.09 (s, 3 H, Ar-CH_3); ^{13}C NMR (101 MHz, DMSO-d_6) δ 153.7 (Ar-C_q-OH), 137.2 (Ar-*C_q*-C_q), 135.8 ($Ar_{(indole)}$-C_q), 128.4 (Ar-CH), 127.1 (N-Ar-CH), 125.5 ($Ar_{(indole)}$-C_q), 124.4 ($Ar_{(indole)}$-CH), 123.5 (Ar-*C_q*-CH_3), 121.2 ($Ar_{(indole)}$-CH), 119.4 (N-Ar-CH-*C_q*), 119.2 ($Ar_{(indole)}$-CH), 118.6 (Ar-CH), 114.1 (Ar-CH), 109.9 ($Ar_{(indole)}$-CH), 82.9 (*C*H_2O*C*H_2), 45.5 (C_q), 32.3 (NCH_3), 16.2 (Ar-CH_3); HRMS (+ESI) *m/z* calcd for $C_{19}H_{20}NO_2$ [M+H]: 294.1494, Found:294.1483.

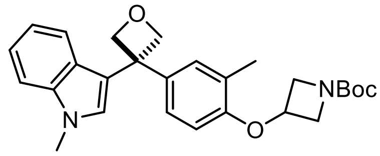

tert-butyl 3-(2-methyl-4-(3-(1-methyl-1*H*-indol-3-yl)oxetan-3-yl)phenoxy)azetidine-1-carboxylate (12a)

A solution of oxetane **5a** (249 mg, 1.04 mmol) in DMF (3.5 mL) was added to a solution of NaH (60% dispersion in mineral oil, 50 mg, 1.25 mmol) in DMF (0.5 mL) at 0 °C. The reaction mixture was stirred for 30 min at 0 °C and *tert*-butyl-3-bromoazetidine-1-carboxylate (504 mg, 2.10 mmol) was weighed into a flask, dissolved in DMF (0.2 mL) and added to the reaction mixture *via* syringe. DMF (0.5 mL) was then added to the previous flask and added to the reaction. The reaction mixture was heated up to 80 °C and stirred for 29 h. Water (20 mL) was added followed by EtOAc (20 mL). The layers were separated and the aqueous portion was extracted with EtOAc (3 × 20 mL). The organic extracts were combined, dried over Na_2SO_4, filtered and concentrated under reduced pressure. Purification by flash column chromatography (15% EtOAc/pentane to 50% EtOAc/pentane) afforded oxetane **12a** (332 mg, 71%) as a white powder. R_f = 0.29 (60% Et_2O/pentane); mp = 69–71 °C; IR (film)/cm^{-1} 2952, 2876, 1697 (C=O), 1391, 1365, 1239, 1128, 986, 740; ^{1}H NMR (400 MHz, $CDCl_3$) δ 7.71 (d, *J* = 8.0 Hz, 1 H, $Ar_{(indole)}$-CH), 7.34 (d, *J* = 8.0 Hz, 1 H, $Ar_{(indole)}$-CH), 7.29–7.25 (m, 1 H, $Ar_{(indole)}$-CH), 7.20 (d, *J* = 1.9 Hz, 1 H, Ar-CH), 7.13–7.09 (m, 1 H, $Ar_{(indole)}$-CH), 6.99 (dd, *J* = 8.3, 1.9 Hz, 1 H, Ar-CH), 6.50 (s, 1 H, N-Ar-CH), 6.41 (d, *J* = 8.3 Hz, 1 H, Ar-CH), 5.38 (d, *J* = 5.5 Hz, 2 H, C*H*HOC*H*H), 5.28 (d, *J* = 5.5 Hz, 2 H, CH*H*OCH*H*), 4.91–4.86 (m, 1 H, OCH), 4.34–4.30 (m, 2 H, C*H*HNC*H*H), 4.04 (dd, *J* = 9.9, 4.1 Hz, 2 H, CH*H*NCH*H*), 3.74 (s, 3 H, NCH_3), 2.26 (s, 3 H, Ar-CH_3), 1.47 (s, 9 H, 3 × CH_3); ^{13}C NMR (101 MHz, $CDCl_3$) δ 156.2 (C=O), 153.3 (Ar-*C_q*-OCH), 138.3 (Ar-*C_q*-C_q), 137.6 ($Ar_{(indole)}$-C_q), 129.3 (Ar-CH), 127.0 (N-Ar-CH), 127.0 ($Ar_{(indole)}$-C_q), 126.1 (Ar-*C_q*-CH_3), 124.8 (Ar-CH), 121.9 ($Ar_{(indole)}$-CH), 120.0 ($Ar_{(indole)}$-CH), 119.8 (N-Ar-CH-*C_q*), 119.3 ($Ar_{(indole)}$-CH), 110.3 (Ar-CH), 109.5 ($Ar_{(indole)}$-CH), 84.2 (*C*H_2O*C*H_2), 79.8 (*C_q*(CH_3)$_3$), 65.7 (OCH), 56.5 (br, *C*H_2N*C*H_2), 46.1 (C_q), 32.7 (NCH_3), 28.3 (C_q(*C*H_3)$_3$), 16.4 (Ar-CH_3); HRMS (+ESI) *m/z* calcd for $C_{27}H_{33}N_2O_4$ [M+H]: 449.2440, Found: 449.2447.

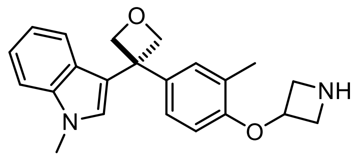

3-(3-(4-(Azetidin-3-yloxy)-3-methylphenyl)oxetan-3-yl)-1-methyl-1*H*-indole (1a)

Trifluoroacetic acid (430 µL, 5.70 mmol) was added dropwise to a solution of **12a** (301 mg, 0.67 mmol) in CH_2Cl_2 (3.0 mL) at 0 °C. The reaction mixture was stirred at 0 °C for further 30 min and then at 25 °C for 15 h. A solution of NaOH (1 M, 20 mL) was then added and the reaction mixture was stirred for 5 min. CH_2Cl_2 (20 mL) was then added and the layers were separated. The aqueous portion was extracted with CH_2Cl_2 (4 × 20 mL). The organic extracts were combined, dried over Na_2SO_4, filtered and concentrated under reduced pressure. Purification by SCX column afforded amine **1a** (207 mg, 89%) as a yellow powder. R_f = 0.22 (95:5:1 CH_2Cl_2/Methanol/NEt_3); mp = 100–103 °C; IR (film)/cm^{-1} 2946 (NH), 2872, 1501, 1239, 1150, 984, 808, 737; ^{1}H NMR (400 MHz, $CDCl_3$) δ 7.72 (d, *J* = 8.0 Hz, 1 H, $Ar_{(indole)}$-CH), 7.34 (d, *J* = 8.2 Hz, 1 H, $Ar_{(indole)}$-CH), 7.29–7.25 (m, 1 H, $Ar_{(indole)}$-CH), 7.19 (d, *J* = 2.0 Hz, 1 H, Ar-CH), 7.13–7.09 (m, 1 H, $Ar_{(indole)}$-CH), 6.98 (dd, *J* = 8.3, 2.4 Hz, 1 H, Ar-CH), 6.50 (s, 1 H, N-Ar-CH), 6.48 (d, *J* = 8.3 Hz, 1 H, Ar-CH), 5.38 (d, *J* = 5.5 Hz, 2 H, C*H*HOC*H*H), 5.29 (d, *J* = 5.5 Hz, 2 H, CH*H*OCH*H*), 5.01 (p, *J* = 6.1 Hz, 1 H, OCH),

3.97–3.93 (m, 2 H, C*H*HNC*H*H), 3.86–3.82 (m, 2 H, CH*H*NCH*H*), 3.73 (s, 3 H, NCH_3), 2.26 (s, 3 H, Ar-CH_3), 1.93 (s, 1 H, NH); ^{13}C NMR (101 MHz, $CDCl_3$) δ 153.8 (Ar-*C_q*-OCH), 137.8 (Ar-*C_q*-C_q), 137.6 ($Ar_{(indole)}$-C_q), 129.1 (Ar-CH), 127.0 (N-Ar-CH), 126.7 ($Ar_{(indole)}$-C_q), 126.2 (Ar-*C_q*-CH_3), 124.8 (Ar-CH), 121.9 ($Ar_{(indole)}$-CH), 120.1 ($Ar_{(indole)}$-CH), 119.9 (N-Ar-CH-*C_q*), 119.2 ($Ar_{(indole)}$-CH), 110.7 (Ar-CH), 109.4 ($Ar_{(indole)}$-CH), 84.3 (*C*H_2O*C*H_2), 70.6 (OCH), 55.0 (*C*H_2N*C*H_2), 46.1 (C_q), 32.6 (NCH_3), 16.4 (Ar-CH_3); HRMS (+ESI) *m/z* calcd for $C_{22}H_{25}N_2O_2$ [M+H] : 349.1916, Found: 349.1922.

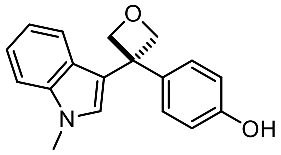

4-(3-(1-Methyl-1*H*-indol-3-yl)oxetan-3-yl)phenol (6a)

Lithium bis(trifluoromethane)sulfonimide (83.3 mg, 0.29 mmol) and tetrabutylammonium hexafluorophosphate (55.8 mg, 0.14 mmol) were added to a solution of oxetanol **11a** (532 mg, 2.62 mmol) and phenol (1.23 g, 13.1 mmol) in $CHCl_3$ (5.2 mL). The reaction mixture was stirred at 40 °C for 3 h then sat. aq. $NaHCO_3$ (50 mL) was added followed by EtOAc (50 mL). The layers were separated, and the aqueous portion was extracted with EtOAc (3 × 50 mL). The organic extracts were combined, dried over Na_2SO_4, filtered and concentrated under reduced pressure. Purification by flash column chromatography (30% EtOAc/pentane) afforded oxetane **6a** (204 mg, 28%) as a yellow solid. R_f = 0.43 (50% EtOAc/pentane); mp = 207–209 °C; IR (film)/cm^{-1} 3477, 3131 (br, OH), 2953, 2880, 1516, 1374, 1218, 837, 814, 741; ^{1}H NMR (400 MHz, DMSO-*d_6*) δ 9.33 (s, 1 H, OH), 7.39 (dd, *J* = 14.0, 8.1 Hz, 2 H, 2 × $Ar_{(indole)}$-CH), 7.16–7.13 (m, 3 H, $Ar_{(indole)}$-CH, 2 × Ar-CH), 7.03 (s, 1 H, N-Ar-CH), 6.99–6.97 (m, 1 H, $Ar_{(indole)}$-CH), 6.73 (d, *J* = 8.6 Hz, 2 H, 2 × Ar-CH), 5.14 (dd, *J* = 13.9, 5.6 Hz, 4 H, C*H_2*OC*H_2*), 3.73 (s, 3 H, NCH_3); ^{13}C NMR (101 MHz, DMSO-*d_6*) δ 155.6 (Ar-C_q-OH), 137.2 (Ar-*C_q*-C_q), 136.0 ($Ar_{(indole)}$-C_q), 127.3 (2 × Ar-CH), 127.1 (N-Ar-CH), 125.6 ($Ar_{(indole)}$-C_q), 121.3 ($Ar_{(indole)}$-CH), 119.3 (N-Ar-CH-*C_q*), 119.3 ($Ar_{(indole)}$-CH), 118.7 ($Ar_{(indole)}$-CH), 115.0 (2 × Ar-CH), 110.0 ($Ar_{(indole)}$-CH), 83.0 (*C*H_2O*C*H_2), 45.5 (C_q), 32.3 (NCH_3); HRMS (+ESI) *m/z* calcd for $C_{18}H_{18}NO_2$ [M+H] : 280.1338, Found: 280.1346.

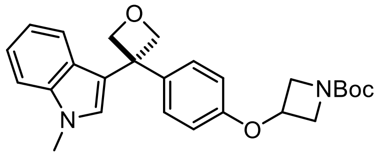

***tert*-Butyl 3-(4-(3-(1-methyl-1*H*-indol-3-yl)oxetan-3-yl)phenoxy)azetidine-1-carboxylate (13a)**

A solution of oxetane **6a** (163 mg, 0.58 mmol) in DMF (1.5 mL) was added to a solution of NaH (60% dispersion in mineral oil, 59.0 mg, 1.48 mmol) in DMF (0.3 mL) at 0 °C. The reaction mixture was stirred for 30 min at 0 °C and then *tert*-butyl-3-bromoazetidine-1-carboxylate (274 mg, 1.16 mmol) was weighed into a flask, dissolved in DMF (0.3 mL) and added to the reaction mixture *via* syringe. DMF (0.7 mL) was added to the previous flask and then added to the reaction. The reaction mixture was heated to 80 °C. After 24 h, additional *tert*-butyl-3-bromoazetidine-1-carboxylate (137 mg, 0.58 mmol) was added to the reaction mixture *via* syringe and the reaction mixture was stirred at 80 °C for 22 h before addition of NaH (60% dispersion in mineral oil, 12 mg, 0.29 mmol). The reaction mixture was stirred for a further 23 h and then water (20 mL) was added followed by EtOAc (20 mL). The layers were separated and the aqueous portion was extracted with EtOAc (3 × 20 mL). The organic extracts were combined, dried over Na_2SO_4, filtered and concentrated under reduced pressure. Purification by flash column chromatography (15% EtOAc/pentane to 30% EtOAc/pentane) afforded oxetane **13a** (199 mg, 79%) as a white powder. R_f = 0.14 (50% Et_2O/pentane); mp = 65–70 °C; IR (film)/cm^{-1} 2974, 1694 (C=O), 1595, 1523, 1465, 1383, 1364, 1264, 1246, 1135, 1111, 772, 748; ^{1}H NMR (400 MHz, $CDCl_3$) δ 7.68 (d, *J* = 8.0 Hz, 1 H, $Ar_{(indole)}$-CH), 7.36 (d, *J* = 8.3 Hz, 1 H, $Ar_{(indole)}$-CH), 7.31–7.28 (m, 3 H, $Ar_{(indole)}$-CH, 2 × Ar-CH), 7.14–7.10 (m, 1 H, $Ar_{(indole)}$-CH), 6.75 (d, *J* = 8.7 Hz, 2 H, 2 × Ar-CH), 6.53 (s, 1 H, N-Ar-CH), 5.41 (d, *J* = 5.6 Hz, 2 H, C*H*HOC*H*H), 5.28 (d, *J* = 5.6 Hz, 2 H, CH*H*OCH*H*), 4.93–4.88 (m, 1 H, OCH), 4.32 (dd, *J* = 10.3, 6.4 Hz, 2 H, C*H*HNC*H*H), 4.05 (dd, *J* = 10.3, 4.1 Hz, 2 H, CH*H*NCH*H*), 3.76 (s, 3 H, NCH_3), 1.48 (s, 9 H, 3 × CH_3); ^{13}C NMR (101 MHz, $CDCl_3$) δ 156.1 (C=O), 155.2 (Ar-*C_q*-OCH), 138.8 (Ar-*C_q*-C_q), 137.6 ($Ar_{(indole)}$-C_q), 127.9

(2 × Ar-CH), 127.0 (N-Ar-CH), 126.1 ($Ar_{(indole)}$-C_q), 122.0 ($Ar_{(indole)}$-CH), 120.0 ($Ar_{(indole)}$-CH), 119.6 (N-Ar-CH-*C*$_q$), 119.3 ($Ar_{(indole)}$-CH), 114.4 (2 × Ar-CH), 109.5 ($Ar_{(indole)}$-CH), 84.2 (CH_2OCH_2), 79.8 ($C_q(CH_3)_3$), 65.7 (OCH), 56.3 (br, CH_2NCH_2), 46.1 (C_q), 32.7 (NCH_3), 28.3 ($C_q(CH_3)_3$); HRMS (+ESI) *m/z* calcd for $C_{26}H_{31}N_2O_4$ [M+H] : 435.2284, Found: 435.2284.

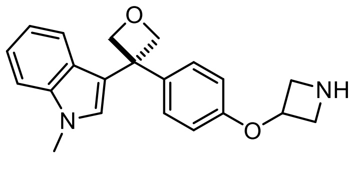

3-(3-(4-(Azetidin-3-yloxy)phenyl)oxetan-3-yl)-1-methyl-1*H*-indole (2a) Trifluoroacetic acid (320 µL, 4.20 mmol) was added dropwise to a solution of **13a** (183 mg, 0.42 mmol) in CH_2Cl_2 (2.1 mL) at 0 °C. The reaction mixture was stirred at 0 °C for further 30 min and then at 25 °C for 18 h. A solution of NaOH (1 M, 20 mL) was then added and the reaction mixture was stirred for 5 min. CH_2Cl_2 (20 mL) was then added and the layers were separated. The aqueous portion was extracted with CH_2Cl_2 (4 × 20 mL). The organic extracts were combined, dried over Na_2SO_4, filtered and concentrated under reduced pressure. Purification by SCX column afforded amine **2a** (125 mg, 89%) as a yellow powder. R_f = 0.18 (95:5:1 CH_2Cl_2/Methanol/NEt_3); mp = 100–103 °C; IR (film)/cm^{-1} 2942 (NH), 2872, 1508, 1237, 1181, 984, 828, 740; ^{1}H NMR (400 MHz, $CDCl_3$) δ 7.66 (d, *J* = 8.0 Hz, 1 H, $Ar_{(indole)}$-CH), 7.32 (d, *J* = 8.2 Hz, 1 H, $Ar_{(indole)}$-CH), 7.25–7.22 (m, 3 H, $Ar_{(indole)}$-CH, 2 × Ar-CH), 7.09 (dd, *J* = 11.0, 3.9 Hz, 1 H, $Ar_{(indole)}$-CH), 6.74 (d, *J* = 8.7 Hz, 2 H, 2 × Ar-CH), 6.50 (s, 1 H, N-Ar-CH), 5.37 (d, *J* = 5.5 Hz, 2 H, C*H*HOC*H*H), 5.26 (d, *J* = 5.5 Hz, 2 H, CH*H*OCH*H*), 5.01–4.98 (m, 1 H, OCH), 3.93 (m, 2 H, C*H*HNC*H*H), 3.82 (m, 2 H, CH*H*NCH*H*), 3.72 (s, 3 H, NCH_3), 2.02 (br, s, 1 H, NH); ^{13}C NMR (101 MHz, $CDCl_3$) δ 155.6 (Ar-*C*$_q$-OCH), 138.3 (Ar-*C*$_q$-C_q), 137.6 ($Ar_{(indole)}$-C_q), 127.8 (2 × Ar-CH), 127.0 (N-Ar-CH), 126.1 ($Ar_{(indole)}$-C_q), 121.9 ($Ar_{(indole)}$-CH), 120.0 ($Ar_{(indole)}$-CH), 119.7 (N-Ar-CH-*C*$_q$), 119.3 ($Ar_{(indole)}$-CH), 114.4 (2 × Ar-CH), 109.5 ($Ar_{(indole)}$-CH), 84.3 (CH_2OCH_2), 70.5 (OCH), 54.8 (CH_2NCH_2), 46.1 (C_q), 32.7 (NCH_3); HRMS (+ESI) *m/z* calcd for $C_{21}H_{23}N_2O_2$ [M+H] : 335.1760, Found: 335.1776.

Synthesis of diarylketone 1b

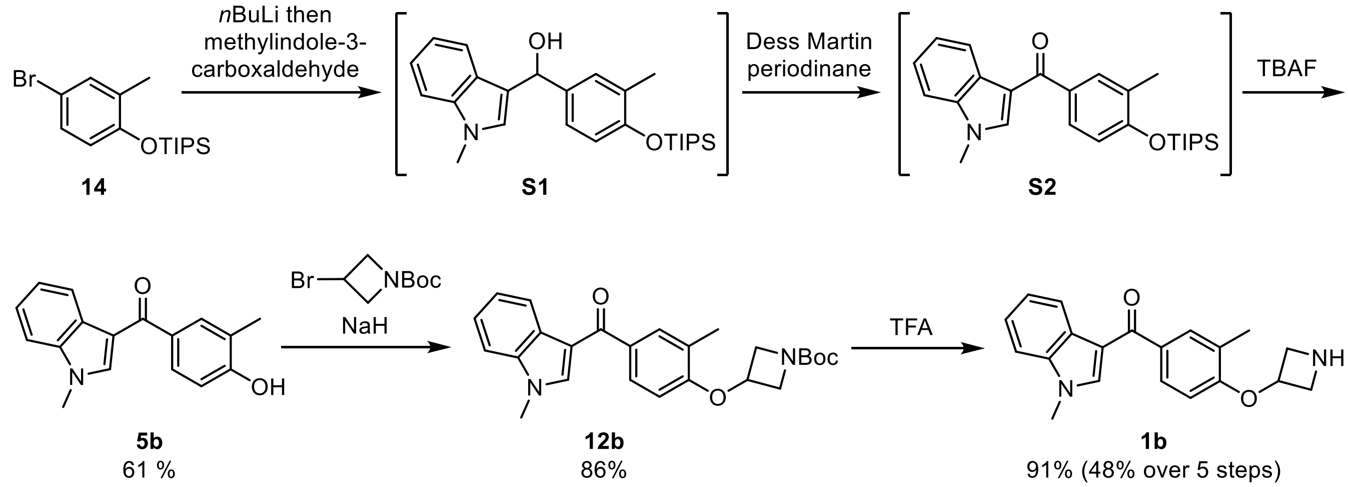

Figure S2.

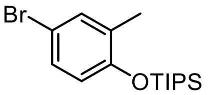

(4-Bromo-2-methylphenoxy)triisopropylsilane (14)

2,6-Lutidine (1.60 mL, 13.8 mmol) was added dropwise to a solution of 4-bromo 2-methylphenol (2.00 g, 10.7 mmol) and triisopropylsilyltrifluoromethanesulfonate (3.45 mL, 12.8 mmol) in CH_2Cl_2 (21.4 mL) at 0 °C. The reaction mixture was stirred for 30 min at 0 °C and then 5 h at 25 °C. Water (100 mL) was added followed by CH_2Cl_2 (80 mL). The layers were separated and the aqueous portion was extracted with CH_2Cl_2 (2 × 80 mL). The organic extracts were combined, dried over Na_2SO_4, filtered and concentrated under reduced pressure. Purification by flash column chromatography (100% pentane) afforded **14** (3.66 g, 99%) as a colourless oil. R_f = 0.63 (100% pentane); IR (film)/cm^{-1} 2945, 2867, 1487, 1394, 1283, 1269, 1186, 1125, 996, 882, 808, 702, 682; ^{1}H NMR (400 MHz, $CDCl_3$) δ 7.26 (d, *J* = 2.6 Hz, 1 H, Ar-CH), 7.15 (dd, *J* = 8.6, 2.6 Hz, 1 H, Ar-CH), 6.66 (d, *J* = 8.6 Hz, 1 H, Ar-CH), 2.23 (s, 3 H, Ar-CH_3), 1.35–1.26 (m, 3 H, 3 × $CH(CH_3)_2$), 1.12 (d, *J* = 7.30 Hz, 18 H, 3 × $CH(CH_3)_2$); ^{13}C NMR (101 MHz, $CDCl_3$) δ 153.5 (Ar-C_q-OTIPS), 133.5 (Ar-CH), 131.0 (Ar-C_q-CH_3), 129.2 (Ar-CH), 119.4 (Ar-CH), 112.5 (Ar-C_q-Br), 18.0 (Ar-CH_3), 16.9 (3 × $CH(CH_3)_2$), 13.0 (3 × $CH(CH_3)_2$). The observed spectroscopic and physical data (^{1}H) for this compound was consistent with that previously reported.[2]

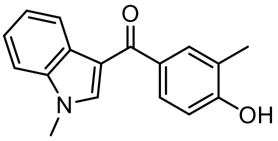

(4-Hydroxy-3-methylphenyl)(1-methyl-1*H*-indol-3-yl)methanone (5b)

*n*BuLi (2.38 M in hexane, 590 µL, 1.40 mmol) was added dropwise in the solution of TIPS-protected bromo-phenol **14** (515 mg, 1.50 mmol) in THF (1.0 mL) at –78 °C and was stirred for 30 min. A solution of 1-methylindole-3-carboxaldehyde (159 mg, 1.00 mmol) in THF (2.3 mL) was added to the reaction mixture and stirred at –78 °C for further 4 h. The reaction was warmed to rt and then water (20 mL) was added followed by Et_2O (20 mL). The layers were separated and the aqueous portion was extracted with Et_2O (3 × 20 mL). The organic extracts were combined, dried over Na_2SO_4, filtered and concentrated under reduced pressure to give alcohol **S1** which was used directly in the next step without further purification due to instability.

S1: ^{1}H NMR (400 MHz, $CDCl_3$) δ 7.58 (d, *J* = 8.0 Hz, 1 H, $Ar_{(indole)}$-CH), 7.31–7.29 (d, *J* = 8.2 Hz, 1 H, $Ar_{(indole)}$-CH), 7.26–7.21 (m, 2 H, Ar-CH, $Ar_{(indole)}$-CH), 7.17–7.14 (dd, *J* = 8.2, 2.2 Hz, 1 H, Ar-CH), 7.10–7.06 (m, 1 H, $Ar_{(indole)}$-CH), 6.83 (s, 1 H, N-Ar-CH), 6.77 (d, *J* = 8.2 Hz, 1 H, Ar-CH) , 6.09 (d, *J* = 4.1 Hz, 1 H, OH), 3.74 (s, 3 H, NCH_3), 2.25 (s, 3 H, Ar-CH_3), 2.09 (d, *J* = 4.1 Hz, 1 H, *CH*OH), 1.35–1.25 (m, 3 H, 3 × $CH(CH_3)_2$), 1.13 (d, *J* = 7.3 Hz, 18 H, 3 × $CH(CH_3)_2$). Compound was too unstable over time in solution to obtain ^{13}C NMR.

Dess-Martin periodinane (509 mg, 1.20 mmol) was added to a solution of alcohol **S1** and pyridine (162 µL, 2.00 mmol) in CH_2Cl_2 (5.0 mL). The reaction mixture was stirred at 25 °C for 18 h and then sat. aq. sodium thiosulfate (20 mL) was added followed by CH_2Cl_2 (20 mL). The layers were separated and the aqueous portion was extracted with CH_2Cl_2 (2 × 20 mL). The organic extracts were combined, dried over Na_2SO_4, filtered and concentrated under reduced pressure. Purification by flash column chromatography (20% Et_2O/pentane) afforded TIPS protected ketone **S2** with an impurity. The mixture was used without further purification.

S2: ^{1}H NMR (400 MHz, $CDCl_3$) δ 8.38 (dd, *J* = 6.3, 2.2 Hz, 1 H, $Ar_{(indole)}$-CH), 7.70 (d, *J* = 2.3 Hz, 1 H, Ar-CH), 7.64–7.55 (m, 2 H, Ar-CH, N-Ar-CH), 7.40–7.33 (m, 3 H, 3 × $Ar_{(indole)}$-CH), 6.85 (d, *J* = 8.3 Hz, 1 H, Ar-CH), 3.88 (s, 3 H, NCH_3), 2.32 (s, 3 H, Ar-CH_3), 1.40–1.32 (m, 3 H, 3 × C*H*(CH_3)$_2$), 1.15 (d, *J* = 7.47 Hz, 18 H, 3 × CH(C*H*$_3$)$_2$); ^{13}C NMR (101 MHz, $CDCl_3$) δ 190.4 (C=O), 157.5 (Ar-C_q-O), 141.8 (N-Ar-CH), 137.4 ($Ar_{(indole)}$-C_q), 133.3 (Ar-CH), 133.2 (Ar-C_q-CO), 132.0 ($Ar_{(indole)}$-CH), 128.6 ($Ar_{(indole)}$-C_q), 128.3 ($Ar_{(indole)}$-CH), 127.3 (Ar-C_q-CH_3), 123.4 ($Ar_{(indole)}$-CH), 122.4 (Ar-CH), 117.3 (Ar-CH), 115.7 (N-Ar-CH-C_q), 109.5 ($Ar_{(indole)}$-CH), 33.5 (NCH_3), 18.0 (3 × CH(*C*H$_3$)$_2$), 17.1 (Ar-CH_3), 13.0 (3 × *C*H(CH_3)$_2$); HRMS (+ESI) m/z calcd for $C_{26}H_{36}NO_2Si$ [M+H]: 422.2515, Found: 422.2515.

A solution of TBAF (1 M in THF, 1.10 mL, 1.10 mmol) was added to a solution of TIPS-protected ketone **S2** in THF (3.0 mL) at 0 °C and stirred at 25 °C for 3 h and water (20 mL) was added followed by EtOAc (20 mL). The layers were separated and the aqueous portion was extracted with EtOAc (2 × 20 mL). The organic extracts were combined, dried over Na_2SO_4, filtered and concentrated under reduced pressure. Purification by flash column chromatography (30% to 50 EtOAc/pentane) afforded **5b** as a yellow solid (162 mg, 61% over three steps). R_f = 0.23 (40% EtOAc/pentane); mp = 239–241 °C; IR (film)/cm^{-1} 3124 (br, OH), 3051, 2925, 1608 (C=O), 1563, 1522, 1510, 1468, 1368, 1340, 1229, 1273, 1250, 1199, 1168, 1156, 1127, 1081, 1045, 1011, 949, 940, 933, 820, 741, 710; ^{1}H NMR (400 MHz, DMSO-*d$_6$*) δ 10.0 (s, 1 H, OH), 8.23–8.21 (m, 1 H, $Ar_{(indole)}$-CH), 7.99 (s, 1 H, N-Ar-CH), 7.59 (d, *J* = 1.5 Hz, 1 H, Ar-CH), 7.56–7.53 (m, 2 H, $Ar_{(indole)}$-CH, Ar-CH), 7.32–7.23 (m, 2 H, 2 × $Ar_{(indole)}$-CH), 6.90 (d, *J* = 8.3 Hz, 1 H, Ar-CH), 3.88 (s, 3 H, NCH_3), 2.20 (s, 3 H, Ar-CH_3); ^{13}C NMR (101 MHz, DMSO-*d$_6$*) δ 188.4 (C=O), 158.6 (Ar-C_q-OH), 138.2 (N-Ar-CH), 137.1 ($Ar_{(indole)}$-C_q), 131.5 (Ar-CH), 131.3 (Ar-C_q-CO), 128.2 ($Ar_{(indole)}$-CH), 126.9 ($Ar_{(indole)}$-C_q), 123.8 (Ar-C_q-CH_3), 122.8 ($Ar_{(indole)}$-CH), 121.8 (Ar-CH), 121.6 ($Ar_{(indole)}$-CH), 114.0 (Ar-CH), 113.9 (N-Ar-CH-C_q), 110.4 ($Ar_{(indole)}$-CH), 33.0 (NCH_3), 16.0 (Ar-CH_3); HRMS (+ESI) m/z calcd for $C_{17}H_{16}NO_2$ [M+H]: 266.1181, Found: 266.1183.

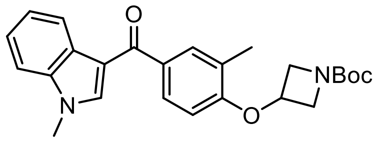

tert-Butyl-3-(2-methyl-4-(1-methyl-1*H*-indole-3-carbonyl)phenoxy)azetidine-1-carboxylate (12b)

A solution of ketone **5b** (124 mg, 0.47 mmol) in DMF (1.80 mL) was added to a solution of NaH (60% dispersion in mineral oil, 22.5 mg, 0.56 mmol) in DMF (0.20 mL) at 0 °C. The reaction mixture was stirred for 30 min at 0 °C and *tert*-butyl-3-bromoazetidine-1-carboxylate (221 mg, 0.94 mmol) in DMF (0.50 mL) was added to the reaction mixture *via* syringe. The reaction mixture was then heated up to 80 °C and stirred for 19 h before addition of further NaH (60% dispersion in mineral oil, 18.6 mg, 0.47 mmol) and *tert*-butyl-3-bromoazetidine-1-carboxylate (110 mg, 0.47 mmol). The reaction mixture was stirred for 24 h and then water (30 mL) was added followed by Et_2O (20 mL). The layers were separated and the aqueous portion was extracted with Et_2O (3 × 30 mL). The organic extracts were combined, dried over Na_2SO_4, filtered and concentrated under reduced pressure. Purification by flash column chromatography (30% Et_2O/pentane) afforded ketone **12b** (169 mg, 86%) as a white powder. R_f = 0.25 (40% EtOAc/pentane); mp = 75–80 °C; IR (film)/cm^{-1} 2952, 2876, 1697 (C=O), 1391, 1365, 1239, 1128, 986, 740; ^{1}H NMR (400 MHz, $CDCl_3$) δ 8.38 (m, 1 H, $Ar_{(indole)}$-CH), 7.71 (d, *J* = 2.2 Hz, 1 H, Ar-CH), 7.64 (dd, *J* = 8.3, 2.2 Hz, 1 H, Ar-CH), 7.54 (s, 1 H, N-Ar-CH), 7.42–7.30 (m, 3 H, 3 × $Ar_{(indole)}$-CH), 6.51 (d, *J* = 8.3 Hz, 1 H, Ar-CH), 4.96 (m, 1 H, OCH), 4.36 (m, 2 H, C*H*HNC*H*H), 4.06 (m, 2 H, CH*H*NCH*H*), 3.86 (s, 3 H, NCH_3), 2.32 (s, 3 H, Ar-CH_3), 1.48 (s,

9 H, 3 × CH_3); ^{13}C NMR (101 MHz, $CDCl_3$) δ 189.8 (C=O), 157.3 ($C=O_{(boc)}$), 156.1 (Ar-C_q-OCH), 137.5 (Ar-C_q-CO), 137.1 (N-Ar-CH), 134.0 ($Ar_{(indole)}$-C_q), 131.9 (Ar-CH), 128.3 (Ar-CH), 127.3 ($Ar_{(indole)}$-C_q), 127.0 (Ar-C_q-CH_3), 123.5 ($Ar_{(indole)}$-CH), 122.7 ($Ar_{(indole)}$-CH), 122.5 ($Ar_{(indole)}$-CH), 115.7 (N-Ar-CH-C_q), 109.9 (Ar-CH), 109.5 ($Ar_{(indole)}$-CH), 79.9 ($C_q(CH_3)_3$), 65.9 (OCH), 56.5 (br, CH_2NCH_2), 33.5 (NCH_3), 28.4 ($C_q(CH_3)_3$),16.3 (Ar-CH_3). HRMS (+ESI) *m/z* calcd for $C_{25}H_{29}N_2O_4$ [M+H] : 421.2127, Found: 421.2126.

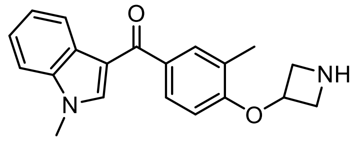

(4-(Azetidin-3-yloxy)-3-methylphenyl)(1-methyl-1*H*-indol-3-yl)methanone (1b)

Trifluoroacetic acid (280 µL, 3.60 mmol) was added dropwise to a solution of **12b** (152 mg, 0.36 mmol) in CH_2Cl_2 (2.0 mL) at 0 °C. The reaction mixture was stirred at 0 °C for 30 min and then at 25 °C for 15 h. A solution of aq. NaOH (1 M, 20 mL) was added and the reaction mixture was stirred for 5 min. Et_2O (20 mL) was added and the layers were separated. The aqueous portion was extracted with Et_2O (3 × 20 mL) and EtOAc (20 mL). The organic extracts were combined, dried over Na_2SO_4, filtered and concentrated under reduced pressure to give amine **1b** (104 mg, 91%) as a white powder. R_f = 0.25 (95:5:1 CH_2Cl_2/Methanol/NEt_3); mp = 70–75 °C; IR (film)/cm^{-1} 3324 (br, NH), 2947, 1596 (C=O), 1520, 1463, 1362, 1263, 1245, 1199, 1145, 1126, 1112, 1075, 772, 748; ^1H NMR (400 MHz, $CDCl_3$) δ 8.42–8.35 (m, 1 H, $Ar_{(indole)}$-CH), 7.70 (d, *J* = 1.3 Hz, 1 H, Ar-CH), 7.64 (dd, *J* = 8.4, 2.3 Hz, 1 H, Ar-CH), 7.54 (s, 1 H, N-Ar-CH), 7.38–7.31 (m, 3 H, 3 × $Ar_{(indole)}$-CH), 6.56 (d, *J* = 8.4 Hz, 1 H, Ar-CH), 5.09 (m, 1 H, OCH), 4.00 (m, 2 H, C*H*HNC*H*H), 3.85 (s, 5 H, CH*H*NCH*H,* NCH_3), 2.31 (s, 3 H, Ar-CH_3), 2.11 (br, s, 1 H, NH); ^{13}C NMR (101 MHz, $CDCl_3$) δ 189.9 (C=O), 157.8 (Ar-C_q-OCH), 137.4 (Ar-C_q-CO), 137.0 (N-Ar-CH), 133.5 ($Ar_{(indole)}$-C_q), 131.7 (Ar-CH), 128.4 (Ar-CH), 127.3 ($Ar_{(indole)}$-C_q), 126.8 (Ar-C_q-CH_3), 123.4 ($Ar_{(indole)}$-CH), 122.7 ($Ar_{(indole)}$-CH), 122.4 ($Ar_{(indole)}$-CH), 115.7 (N-Ar-CH-C_q), 110.2 (Ar-CH), 109.5 ($Ar_{(indole)}$-CH), 70.7 (OCH), 54.8 (CH_2NCH_2), 33.5 (NCH_3), 16.3 (Ar-CH_3); HRMS (+ESI) *m/z* calcd for $C_{20}H_{21}N_2O_2$ [M+H]: 321.1603, Found: 321.1612.

Synthesis of diarylmethane 1c

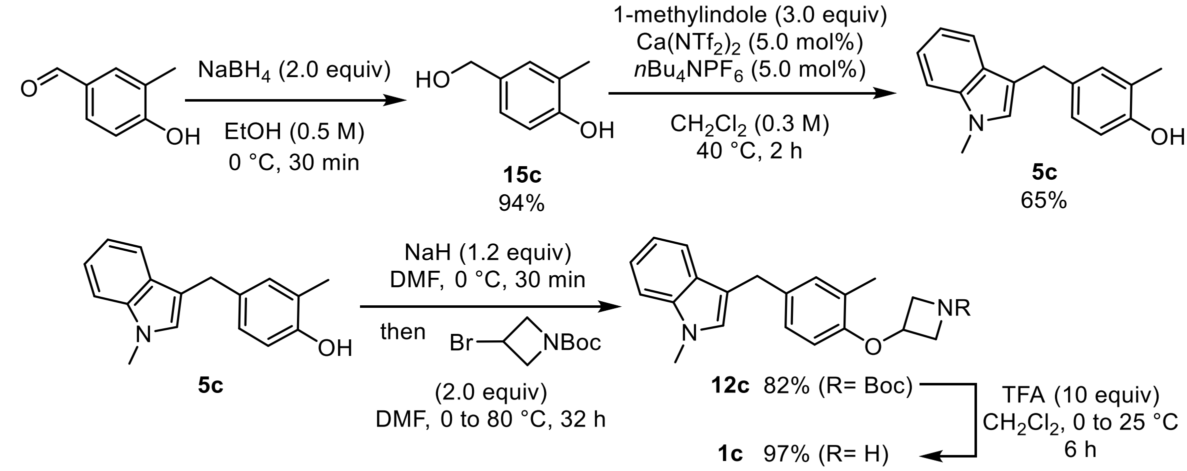

Figure S3.

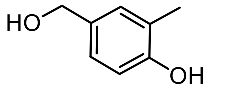

4-(Hydroxymethyl)-2-methylphenol (15c)

$NaBH_4$ (471 mg, 12.4 mmol) was added to a solution of 4-hydroxy-3-methylbenzaldehyde (847 mg, 6.22 mmol) in dry ethanol (12.4 mL) at –5 °C. The reaction mixture was stirred for a further 30 min then the solvent was removed under reduced pressure. EtOAc (50 mL) and water (50 mL) were added. The layers were separated and then the aqueous layer was extracted with EtOAc (3 × 50 mL). The organic extracts were combined, dried over Na_2SO_4, filtered and concentrated under reduced pressure to afford alcohol **15c** (804 mg, 94%) as a pale pink solid. R_f = 0.50 (100% Et_2O); mp = 86–88 °C; IR (film)/cm^{-1} 3405 (br, OH), 3182 (br, OH), 2868, 1739, 1611, 1509, 1472, 1458, 1420, 1352, 1250, 1208, 1114, 1002, 820; ^{1}H NMR (400 MHz, DMSO-d_6) δ 9.10 (s, 1 H, Ar-OH), 6.98 (s, 1 H, Ar-CH), 6.91 (dd, J = 8.1, 1.9 Hz, 1 H, Ar-CH), 6.70 (d, J = 8.1 Hz, 1 H, Ar-CH), 4.89 (t, J = 5.7 Hz, 1 H, CH_2OH), 4.32 (d, J = 5.7 Hz, 2 H, CH_2), 2.10 (s, 3 H, CH_3); ^{13}C NMR (101 MHz, DMSO-d_6) δ 154.2 (Ar-C_q-OH), 132.7 (Ar-C_q-CH_2), 129.5 (Ar-CH), 125.4 (Ar-CH), 123.3 (Ar-C_q-CH_3), 114.3 (Ar-CH), 63.0 (CH_2), 16.2 (Ar-CH_3); HRMS (+EI) m/z calcd for $C_8H_{10}O_2^{\bullet}$ $[M]^{\bullet}$: 138.0681, Found: 138.0677. Compound previously reported.[3]

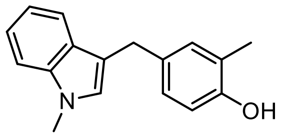

2-Methyl-4((1-methyl-1*H*-indol-3-yl)methyl)phenol (5c)

Calcium(II) bis(trifluoromethanesulfonimide) (174 mg, 0.29 mmol) and tetrabutylammonium hexafluorophosphate (112 mg, 0.29 mmol) were added to a solution of 4-(hydroxymethyl)-2-methylphenol **15c** (799 mg, 5.78 mmol) and 1-methyl-1*H*-indole (2.20 mL,17.4 mmol) in CH_2Cl_2 (19.3 mL). The reaction mixture was stirred at 40 °C for 2 h then sat. aq. $NaHCO_3$ (30 mL) was added followed by CH_2Cl_2 (30 mL). The layers were separated and the aqueous portion was extracted with CH_2Cl_2 (2 × 30 mL). The organic extracts were combined, dried over Na_2SO_4, filtered and concentrated under reduced pressure. Purification by flash column chromatography (10% EtOAc/hexane) afforded diarylmethane **5c** (947 mg, 65%) as a light pink powder. R_f = 0.48 (50% Et_2O/pentane); mp = 77–79 °C; IR (film)/cm^{-1} 3407 (br, OH), 2904, 1506,1483, 1251, 1108, 736; ^{1}H NMR (400 MHz, $CDCl_3$) δ 7.54 (d, J = 7.9 Hz, 1 H, $Ar_{(indole)}$-CH), 7.30 (d, J = 8.2 Hz, 1 H, $Ar_{(indole)}$-CH), 7.24–7.20 (m, 1 H, $Ar_{(indole)}$-CH), 7.10–7.05 (m, 2 H, $Ar_{(indole)}$-CH, Ar-CH), 7.00 (dd, J = 8.0, 2.0 Hz, 1 H, Ar-CH), 6.75 (s, 1 H, N-Ar-CH), 6.69 (d, J = 8.0 Hz, 1 H, Ar-CH), 4.52 (s, 1 H, OH), 3.99 (s, 2 H, CH_2), 3.74 (s, 3 H, NCH_3), 2.22 (s, 3 H, Ar-CH_3); ^{13}C NMR (101 MHz, $CDCl_3$) δ 151.8 (Ar-C_q-OH), 137.1 (Ar-C_q-CH_2), 133.6 ($Ar_{(indole)}$-C_q), 131.2 (Ar-CH), 127.8 (Ar-C_q-CH_3), 127.1 (Ar-CH), 127.0 (N-Ar-CH), 123.4 ($Ar_{(indole)}$-C_q), 121.5 ($Ar_{(indole)}$-CH), 119.2 ($Ar_{(indole)}$-CH), 118.7 ($Ar_{(indole)}$-CH), 114.8 (N-Ar-CH-C_q), 114.7 (Ar-CH), 109.1 ($Ar_{(indole)}$-CH), 32.6 (NCH_3), 30.6 (CH_2), 15.7 (Ar-CH_3); HRMS (+ESI) m/z calcd for $C_{17}H_{18}NO$ [M+H] : 252.1388, Found: 252.1393.

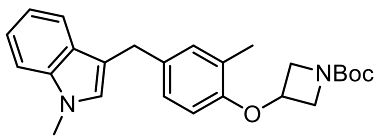

tert-Butyl-3-(2-methyl-4-((1-methyl-1H-indol-3-yl)methyl)phenoxy)azetidine-1-carboxylate (12c)

A solution of **5c** (251 mg, 0.99 mmol) in DMF (2.5 mL) was added to a solution of NaH (60% dispersion in mineral oil, 47.7 mg, 1.20 mmol) in DMF (0.5 mL) at 0 °C. The reaction mixture was stirred for 30 min at 0 °C and then a solution of *tert*-butyl-3-bromoazetidine-1-carboxylate (468 mg, 1.98 mmol) in DMF (1.0 mL) added to the reaction mixture *via* syringe. The reaction was heated up to 80 °C, stirred for 32 h and then water (20 mL) was added followed by EtOAc (20 mL). The layers were separated and the aqueous portion was extracted with EtOAc (3 × 20 mL). The organic extracts were combined, dried over Na_2SO_4, filtered and concentrated under reduced pressure. Purification by flash column chromatography (5% to 10% Acetone/pentane) afforded the ether **12c** (330 mg, 82%) as a white solid. R_f = 0.28 (10% acetone/pentane); mp = 44–47 °C; IR (film)/cm^{-1} 2973, 1696 (C=O), 1391, 1365, 1248, 1218, 1147, 1127, 738; ^{1}H NMR (400 MHz, $CDCl_3$) δ 7.53 (d, *J* = 7.9 Hz, 1 H, $Ar_{(indole)}$-CH), 7.30 (d, *J* = 8.2 Hz, 1 H, $Ar_{(indole)}$-CH), 7.24–7.20 (m, 1 H, $Ar_{(indole)}$-CH), 7.10–7.06 (m, 2 H, $Ar_{(indole)}$-CH, Ar-CH), 7.02 (dd, *J* = 8.2, 1.9 Hz, 1 H, Ar-CH), 6.76 (s, 1 H, N-Ar-CH), 6.36 (d, *J* = 8.2 Hz, 1 H, Ar-CH), 4.84 (m, 1 H, OCH), 4.29 (dd, *J* = 9.6, 6.4 Hz, 2 H, C*H*HNC*H*H), 4.01 (m, *J* = 4.3 Hz, 4 H, CH*H*NCH*H*, CH_2), 3.74 (s, 3 H, NCH_3), 2.21 (s, 3 H, Ar-CH_3), 1.46 (s, 9 H, 3 × CH_3); ^{13}C NMR (101 MHz, $CDCl_3$) δ 156.2 (C=O), 153.0 (Ar-C_q-OCH), 137.2 (Ar-C_q-CH_2), 134.1 ($Ar_{(indole)}$-C_q), 131.5 (Ar-CH), 127.8 (Ar-CH), 126.9 ($Ar_{(indole)}$-C_q), 126.7 (Ar-C_q-CH_3), 126.6 (N-Ar-CH), 121.5 ($Ar_{(indole)}$-CH), 119.2 ($Ar_{(indole)}$-CH), 118.7 ($Ar_{(indole)}$-CH), 114.6 (N-Ar-CH-C_q), 110.7 (Ar-CH), 109.1 ($Ar_{(indole)}$-CH), 79.7 ($C_q(CH_3)_3$), 65.6 (OCH), 56.3 (br, CH_2NCH_2), 32.6 (NCH_3), 30.6 (CH_2), 28.4 ($C_q(CH_3)_3$), 16.2 (Ar-CH_3); HRMS (+ESI) *m/z* calcd for $C_{25}H_{31}N_2O_3$ [M+H]: 407.2337, Found: 407.2335.

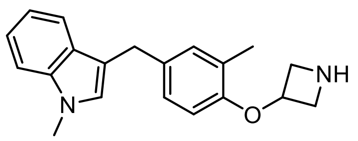

3-(4-(Azetidin-3-yloxy)-3-methylbenzyl)-1-methyl-1H-indole (1c)

Trifluoroacetic acid (600 µL, 7.80 mmol) was added dropwise to a solution of **12c** (318 mg, 0.78 mmol) in CH_2Cl_2 (3.9 mL) at 0 °C. The reaction mixture was stirred at 0 °C for further 30 min and then at 25 °C for 6 h. A solution of NaOH (1 M, 20 mL) was then added and the reaction mixture was stirred for 5 min. CH_2Cl_2 (20 mL) was added and the layers were separated. The aqueous portion was then extracted with CH_2Cl_2 (4 × 20 mL). The organic extracts were combined, dried over Na_2SO_4, filtered and concentrated under reduced pressure. Purification by SCX column afforded amine **1c** (231 mg, 97%) as a beige solid. R_f = 0.26 (95:5:1 CH_2Cl_2/Methanol/NEt_3); mp = 80–82 °C; IR (film)/cm^{-1} 2943 (NH), 1499, 1471, 1248, 1217, 1148, 737; ^{1}H NMR (400 MHz, $CDCl_3$) δ 7.58 (d, *J* = 7.8 Hz, 1 H, $Ar_{(indole)}$-CH), 7.32 (d, *J* = 8.1 Hz, 1 H, $Ar_{(indole)}$-CH), 7.28–7.24 (m, 1 H, $Ar_{(indole)}$-CH), 7.14–7.10 (m , 2 H, $Ar_{(indole)}$-CH, Ar-CH), 7.05 (d, *J* = 8.4 Hz, 1 H, Ar-CH), 6.78 (s, 1 H, N-Ar-CH), 6.45 (d, *J* = 8.2 Hz, 1 H, Ar-CH), 4.99 (m, 1 H, OCH), 4.05 (s, 2 H, CH_2), 3.96 (m, 2 H, C*H*HNC*H*H), 3.84 (m, 2 H, CH*H*NCH*H*), 3.75 (s, 3 H, NCH_3), 2.72 (br, s, 1 H, NH), 2.25 (s, 3 H, Ar-CH_3); ^{13}C NMR (101 MHz, $CDCl_3$) δ 153.3 (Ar-C_q-OCH), 137.1 (Ar-C_q-CH_2), 133.6 ($Ar_{(indole)}$-C_q), 131.3 (Ar-CH), 127.7 ($Ar_{(indole)}$-C_q), 126.9 (N-Ar-CH), 126.5 (Ar-CH), 126.5 (Ar-C_q-CH_3), 121.4 ($Ar_{(indole)}$-CH), 119.1 ($Ar_{(indole)}$-CH), 118.6 ($Ar_{(indole)}$-CH), 114.6 (N-Ar-CH-C_q), 111.0 (Ar-CH), 109.0 ($Ar_{(indole)}$-CH), 70.3 (OCH), 54.9 (CH_2NCH_2), 32.5 (NCH_3), 30.5 (CH_2), 16.2 (Ar-CH_3); HRMS (+ESI) *m/z* calcd for $C_{20}H_{23}N_2O$ [M+H]: 307.1810, Found: 307.1816.

Synthesis of *gem*-dimethyl 1d

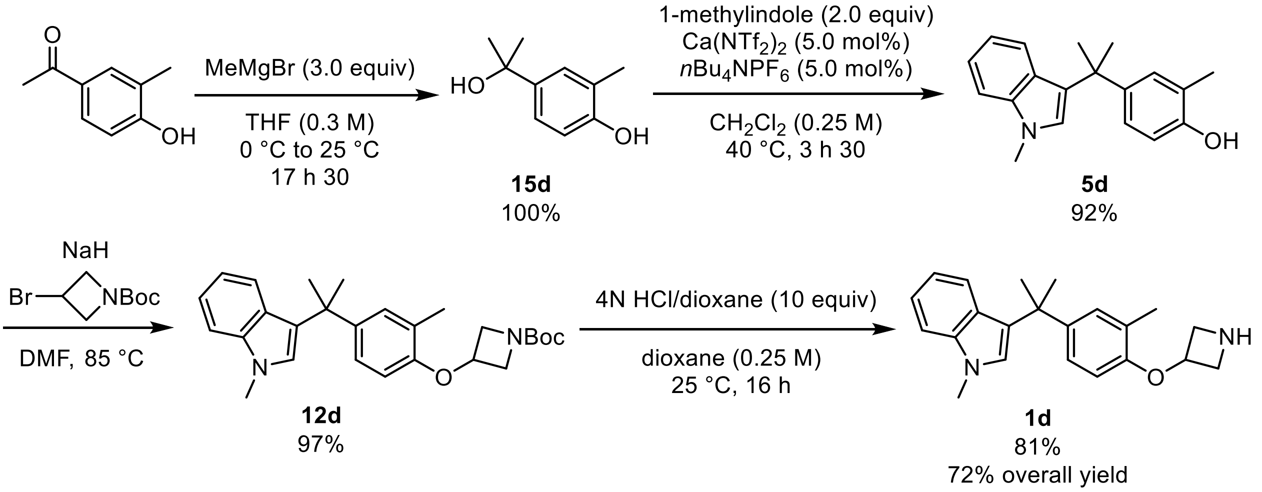

Figure S4.

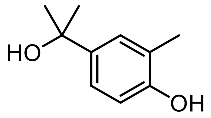

4-(2-Hydroxypropan-2-yl)-2-methylphenol (15d)

A solution of methylmagnesium bromide (2.1 M in THF, 2.85 mL, 6.0 mmol) was added dropwise over 5 min to a solution of 4′-Hydroxy-3′-methylacetophenone (300 mg, 2.0 mmol) in THF (7.0 mL) at 0 °C. The reaction mixture was stirred for 30 min at 0 °C and then 16 h 30 min at 25 °C and then 15 mL of sat. aq. NH_4Cl (15 mL) was added followed by Et_2O (15 mL). The layers were separated and the aqueous portion was extracted with Et_2O (2 × 15 mL). The organic extracts were combined, dried over Na_2SO_4, filtered and concentrated under reduced pressure to give alcohol **15d** (332 mg, 100%) as a yellow brown powder. R_f = 0.24 (50% Et_2O/pentane); mp = 137–140 °C; IR (film)/cm^{-1} 3309 (br, OH), 2972 (br, OH), 1508, 1361, 1281, 1152, 1104, 951, 832, 824; ^{1}H NMR (400 MHz, DMSO-*d₆*) δ 9.00 (s, 1 H, Ar-OH), 7.13 (s, 1 H, Ar-CH), 7.04 (d, *J* = 8.0 Hz, 1 H, Ar-CH), 6.66 (d, *J* = 8.0 Hz, 1 H, Ar-CH), 4.75 (s, 1 H, OH), 2.10 (s, 3 H, Ar-CH_3), 1.36 (s, 6 H, CH_3CCH_3); ^{13}C NMR (101 MHz, DMSO-d_6) δ 153.4 (Ar-C_q-OH), 140.8 (Ar-C_q-CH_3), 126.9 (Ar-CH), 122.7 (Ar-CH), 122.5 (Ar-C_q-$C(CH_3)_2$), 113.7 (Ar-CH), 70.2 ($C_q(CH_3)_2$), 32.1 ($C_q(CH_3)_2$), 16.3 (Ar-CH_3). HRMS (EI+) *m/z* calcd for $C_{10}H_{14}O_2^{\bullet}$ $[M]^{\bullet}$: 166.0994, Found: 166.0988.

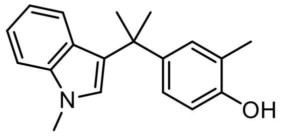

2-Methyl-4-(2-(1-methyl-1*H*-indol-3-yl)propan-2-yl)phenol (5d)

Calcium(II) bis(trifluoromethanesulfonimide) (99.0 mg, 0.17 mmol) and tetrabutylammonium hexafluorophosphate (63.9 mg, 0.17 mmol) were added to a solution of **15d** (548 mg, 3.30 mmol) and 1-methyl-1*H*-indole (824 µL, 6.60 mmol) in CH_2Cl_2 (15.0 mL). The reaction mixture was stirred at 40 °C for 3 h then sat. aq. $NaHCO_3$ (50 mL) was added followed by CH_2Cl_2 (50 mL). The layers were separated and the aqueous portion was extracted with CH_2Cl_2 (3 × 50 mL). The organic extracts were combined, dried over Na_2SO_4, filtered and concentrated under reduced pressure. Purification by flash column chromatography (5% EtOAc/pentane) afforded *gem*-dimethyl derivative **5d** (850 mg, 92%) as a yellow solid. R_f = 0.80 (50% Et_2O/pentane); mp = 110–112 °C; IR (film)/cm^{-1} 3517 (br, OH), 2958, 2929, 1506, 1482, 1324, 1103, 818, 750; ^{1}H NMR (400 MHz, $CDCl_3$) δ 7.29 (d, *J* = 8.2 Hz, 1 H, $Ar_{(indole)}$-CH), 7.18–7.11 (m, 3 H, 2 × $Ar_{(indole)}$-CH, Ar-CH), 7.05 (dd, *J* = 8.3, 2.4 Hz, 1 H, Ar-CH), 6.94 (s, 1 H, N-Ar-CH), 6.92–6.88 (m, 1 H, $Ar_{(indole)}$-CH), 6.65 (d, *J* = 8.3 Hz, 1 H, Ar-CH), 4.53 (s, 1 H, OH), 3.79 (s, 3 H, NCH_3), 2.21 (s, 3 H, Ar-CH_3), 1.75 (s, 6 H, CH_3CCH_3); ^{13}C NMR (101 MHz, $CDCl_3$) δ 151.5 (Ar-C_q-OH), 142.4 (Ar-C_q-$C(CH_3)_2$), 137.8 ($Ar_{(indole)}$-C_q), 129.0 (Ar-CH), 126.4 ($Ar_{(indole)}$-C_q), 125.4 (N-Ar-CH), 125.1 (Ar-CH), 124.9 (Ar-C_q-CH_3), 122.8 (N-Ar-CH-C_q), 121.5 ($Ar_{(indole)}$-CH), 121.1 ($Ar_{(indole)}$-CH), 118.2 ($Ar_{(indole)}$-CH), 114.3 (Ar-CH), 109.0 ($Ar_{(indole)}$-CH), 38.2 ($C_q(CH_3)_2$), 32.6 (NCH_3), 31.0 ($C_q(CH_3)_2$), 16.0 (Ar-CH_3); HRMS (+ESI) *m/z* calcd for $C_{19}H_{22}NO$ [M+H] : 280.1701, Found: 280.1715.

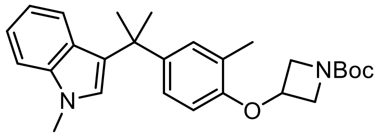

tert-Butyl-3-(2-methyl-4-(2-(1-methyl-1*H*-indol-3-yl)propan-2-yl)phenoxy)azetidine-1-carboxylate (12d)

A solution of **5d** (500 mg, 1.79 mmol) in DMF (4.7 mL) was added to a solution of NaH (60% dispersion in mineral oil, 85.9 mg, 2.15 mmol) in DMF (1.1 mL) at 0 °C. The reaction mixture was stirred for 30 min at 0 °C and then *tert*-butyl-3-bromoazetidine-1-carboxylate (845 mg, 3.58 mmol) was weighed into a flask, dissolved in DMF (0.5 mL) and then added to the reaction mixture *via* a syringe. DMF (1.4 mL) was added to the previous flask and then added to the reaction. The reaction mixture was then heated to 85 °C for further 24 h, then water (30 mL) was added followed by EtOAc (40 mL). The layers were separated and the aqueous portion was extracted with EtOAc (3 × 40 mL). The organic extracts were combined and washed with sat. aq. NaCl (100 mL), dried over Na_2SO_4, filtered and concentrated under reduced pressure. Purification by flash column chromatography (5% EtOAc/pentane) afforded the ether **12d** (757 mg, 97%) as a white solid. R_f = 0.10 (5% EtOAc/pentane); mp = 60–63 °C; IR (film)/cm^{-1} 2964, 1700 (C=O), 1501, 1400, 1365, 1248, 1130, 738; ^1H NMR (400 MHz, $CDCl_3$) δ 7.28 (d, *J* = 8.2 Hz, 1 H, $Ar_{(indole)}$-CH), 7.18–7.05 (m, 4 H, 3 × $Ar_{(indole)}$-CH, Ar-CH), 6.93–6.88 (m, 2 H, Ar-CH, N-Ar-CH), 6.32 (d, *J* = 12 Hz, 1 H, Ar-CH), 4.85–4.82 (m, 1 H, OCH), 4.28 (dd, *J* = 9.5, 6.4, 2 H, C*H*HNC*H*H), 4.01 (dd, *J* = 9.5, 4.1 Hz, 2 H, CH*H*NCH*H*), 3.79 (s, 3 H, NCH_3), 2.19 (s, 3 H, Ar-CH_3), 1.74 (s, 6 H, CH_3CCH_3), 1.47 (s, 9 H, 3 × CH_3); ^{13}C NMR (101 MHz, $CDCl_3$) δ 156.2 (C=O), 152.6 (Ar-C_q-OCH), 142.7 (Ar-C_q-$C(CH_3)_2$), 137.7 ($Ar_{(indole)}$-C_q), 129.3 (Ar-CH), 126.4 ($Ar_{(indole)}$-C_q), 126.0 (Ar-C_q-CH_3), 125.4 (N-Ar-CH), 124.6 (N-Ar-CH-C_q), 124.5 ($Ar_{(indole)}$-CH), 121.4 ($Ar_{(indole)}$-CH), 121.1 ($Ar_{(indole)}$-CH), 118.2 (Ar-CH), 110.1 (Ar-CH), 109.0 ($Ar_{(indole)}$-CH), 79.7 ($C_q(CH_3)_3$), 65.5 (OCH), 56.6 (CH_2NCH_2), 38.2 (CH_3CCH_3), 32.6 (NCH_3), 30.9 ($C_q(CH_3)_2$), 28.3 ($C_q(CH_3)_3$), 16.4 (Ar-CH_3); HRMS (+ESI) *m/z* calcd for $C_{27}H_{35}N_2O_3$ [M+H]: 435.2648, Found: 435.2667.

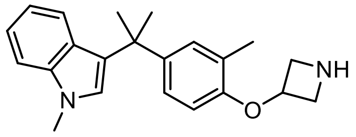

3-(2-(4-(Azetidin-3-yloxy)-3-methylphenyl)propan-2-yl)-1-methyl-1*H*-indole (1d)

A solution of HCl (4 M in dioxane, 1.7 mL, 6.90 mmol) was added to a solution of **12d** (301 mg, 0.69 mmol) in dioxane (2.7 mL) at 25 °C. The reaction mixture was stirred for 20 h and then a solution of aqueous NaOH (1 M, 50 mL) was added until the colour of the mixture changed, followed by the addition of Et_2O (50 mL). The layers were separated and the aqueous portion was extracted with Et_2O (3 × 50 mL). The organic extracts were combined, dried over Na_2SO_4, filtered and concentrated under reduced pressure. Purification by SCX column gave amine **1d** (168 mg, 81%) as a white solid. R_f = 0.35 (95:5:1 CH_2Cl_2/MeOH/NEt_3); mp = 75–80 °C; IR (film)/cm^{-1} 2959 (br, NH), 1499, 1359, 1248, 1149, 1108, 807, 738; ^1H NMR (400 MHz, $CDCl_3$) δ 7.30–7.25 (m, 1 H, $Ar_{(indole)}$-CH), 7.18–7.09 (m, 3 H, 2 × $Ar_{(indole)}$-CH, Ar-CH), 7.05 (dd, *J* = 8.4, 2.4 Hz, 1 H, $Ar_{(indole)}$-CH), 6.94–6.86 (m, 2 H, Ar-CH, N-Ar-CH), 6.38 (d, *J* = 8.5 Hz, 1 H, Ar-CH), 4.99–4.91 (m, 1 H, OCH), 3.94–3.87 (m, 2 H, C*H*HNC*H*H), 3.85–3.78 (m, 2 H, CH*H*NCH*H*), 3.78 (s, 3 H, NCH_3), 2.18 (s, 3 H, Ar-CH_3), 1.96 (br, s, 1 H, NH), 1.74 (s, 6 H, CH_3CCH_3); ^{13}C NMR (101 MHz, $CDCl_3$) δ 153.0 (Ar-C_q-OCH), 142.3 (Ar-C_q-$C(CH_3)_2$), 137.7 ($Ar_{(indole)}$-C_q), 129.1 (Ar-CH), 126.4 ($Ar_{(indole)}$-C_q), 125.8 (Ar-C_q-CH_3), 125.4 (N-Ar-CH), 124.8 (N-Ar-CH-C_q), 124.5 ($Ar_{(indole)}$-CH), 121.5 ($Ar_{(indole)}$-CH), 121.1 ($Ar_{(indole)}$-CH), 118.2 (Ar-CH), 110.5 (Ar-CH), 109.0 ($Ar_{(indole)}$-CH), 70.5 (OCH), 55.1 (CH_2NCH_2), 38.2 ($C_q(CH_3)_2$), 32.6 (NCH_3), 30.9 ($C_q(CH_3)_2$), 16.5 (Ar-CH_3); HRMS (+ESI) *m/z* calcd for $C_{22}H_{27}N_2O$ [M+H]: 335.2123, Found: 335.2133.

Synthesis of cyclobutane 1e

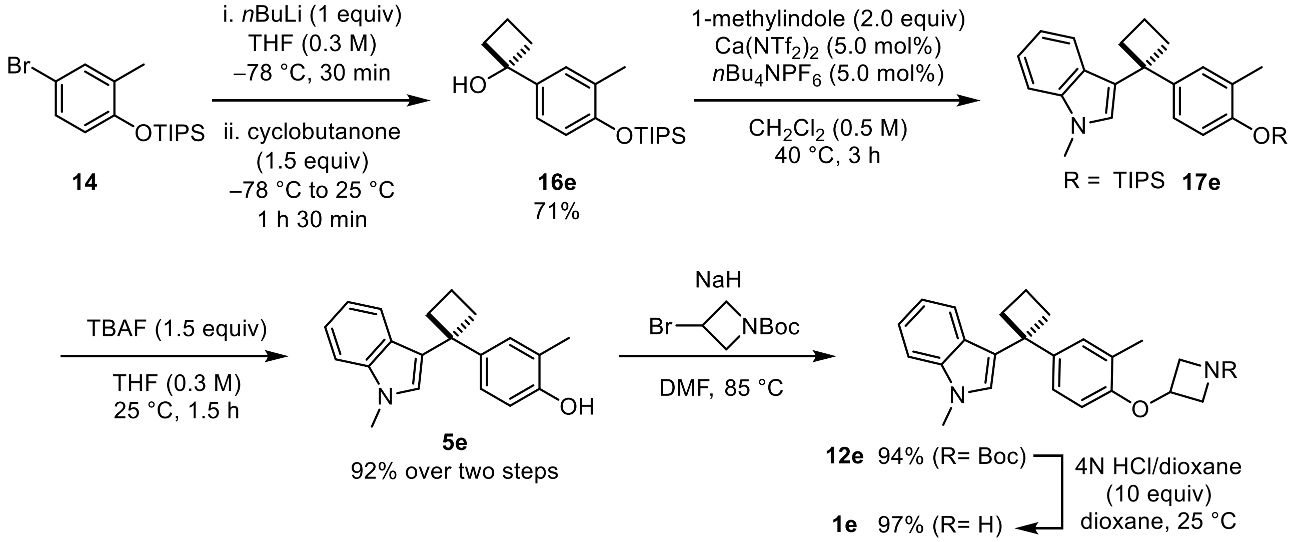

Figure S5.

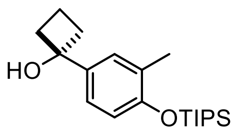

1-(3-Methyl-4-((triisopropylsilyl)oxy)phenyl)cyclobutan-1-ol (16e)

*n*BuLi (2.3 M in THF, 970 µL, 2.24 mmol) was added dropwise over 5 min to a solution of **14** (698 mg, 2.03 mmol) in THF (6.7 mL) at –78 °C. The reaction mixture was stirred at –78 °C for 30 min. Cyclobutanone (200 µL, 3.04 mmol) was added dropwise to the reaction mixture. Following a further 1 h 30 min at –78 °C the reaction mixture was warmed to 25 °C then quenched with water (30 mL). Et_2O (30 mL) was added and the layers were separated. The aqueous portion was extracted with Et_2O (3 × 30 mL). The organic extracts were combined, dried over Na_2SO_4, filtered and concentrated under reduced pressure. Purification by flash column chromatography (10% Et_2O/hexane) afforded cyclobutanol **16e** (480 mg, 71%) as a white crystalline solids. R_f = 0.18 (10% Et_2O/pentane); IR (film)/cm^{-1} 3325 (br, OH), 2944, 2866, 1501, 1263, 1245, 1134, 1120, 940, 883, 817, 782, 720, 681; ^{1}H NMR (400 MHz, $CDCl_3$) δ 7.26 (d, *J* = 2.2 Hz, 1 H, Ar-CH), 7.16 (dd, *J* = 8.3, 2.2 Hz, 1 H, Ar-CH), 6.77 (d, *J* = 8.3 Hz, 1 H, Ar-CH), 2.58–2.52 (m, 2 H, C*H*HCH$_2$C*H*H), 2.38–2.30 (m, 2 H, CH*H*CH$_2$CH*H*), 2.26 (s, 3 H, Ar-CH$_3$), 1.99–1.95 (m, 1 H, CH$_2$C*H*HCH$_2$), 1.94 (s, 1 H, OH), 1.68–1.59 (m, 1 H, CH$_2$CH*H*CH$_2$), 1.35–1.26 (m, 3 H, 3 × C*H*(CH$_3$)$_2$), 1.13 (d, *J* = 7.31 Hz, 18 H, 3 × CH(C*H$_3$*)$_2$); ^{13}C NMR (101 MHz, $CDCl_3$) δ 153.5 (Ar-C$_q$-OTIPS), 138.2 (Ar-*C$_q$*-C$_q$), 128.4 (Ar-*C$_q$*-CH$_3$), 127.8 (Ar-CH), 123.2 (Ar-CH), 117.5 (Ar-CH), 76.8 (C$_q$), 36.7 (*C*H$_2$CH$_2$*C*H$_2$), 18.0 (3 × CH(*C*H$_3$)$_2$), 17.2 (Ar-CH$_3$), 13.0 (3 × *C*H(CH$_3$)$_2$), 12.9 (CH$_2$*C*H$_2$CH$_2$); HRMS (+ESI) *m/z* calcd for $C_{20}H_{33}OSi^\bullet$ [M-OH]$^\bullet$: 317.2301, Found: 317.2313.

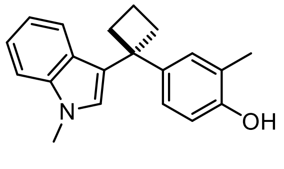

2-Methyl-4-(1-(1-methyl-1*H*-indol-3-yl)cyclobutyl)phenol (5e)

Calcium(II) bis(trifluoromethanesulfonimide) (27.2 mg, 0.04 mmol) and tetrabutylammonium hexafluorophosphate (15.5 mg, 0.04 mmol) were added to a solution of **16e** (273 mg, 0.81 mmol) and 1-methyl-1*H*-indole (206 µL, 1.63 mmol) in CH_2Cl_2 (2.8 mL). The reaction mixture was stirred at 40 °C for 3 h then sat. aq. $NaHCO_3$ (30 mL) was added followed by CH_2Cl_2 (30 mL). The layers were separated and the aqueous portion was extracted with CH_2Cl_2 (4 × 30 mL). The organic extracts were combined, dried over Na_2SO_4, filtered and concentrated under reduced pressure. Attempted purification by flash column chromatography (100 % pentane) afforded inseparable mixture of product **17e** containing unidentified impurity. The mixture was taken on the next step without further purification. A solution of TBAF (1 M in THF, 1.20 mL, 1.20 mmol) was added dropwise over two min to a solution of **17e** (363 mg, 0.81 mmol) in THF (2.7 mL) at 0 °C. The reaction mixture was stirred for 10 min at 0 °C and then 1 h 30 min at 25 °C. Water (40 mL) was added followed by Et_2O (40 mL). The layers were separated and the aqueous portion was extracted

with Et_2O (3 × 40 mL). The organic extracts were combined, dried over Na_2SO_4, filtered and concentrated under reduced pressure. Purification by flash column chromatography (20% Et_2O/hexane) afforded cyclobutane **5e** (218 mg, 92% over 2 steps) as a white solid. R_f = 0.46 (50% Et_2O/pentane); mp = 120–121 °C; IR (film)/cm^{-1} 3532 (OH), 2980, 2944, 1507, 1481, 1324, 1271, 1113, 817, 748, 726; ^{1}H NMR (400 MHz, $CDCl_3$) δ 7.42 (d, *J* = 8.0 Hz, 1 H, $Ar_{(indole)}$-CH), 7.28 (d, *J* = 8.3 Hz, 1 H, $Ar_{(indole)}$-CH), 7.20–7.11 (m, 3 H, $Ar_{(indole)}$-CH, 2 × Ar-CH), 7.01–6.97 (m, 1 H, $Ar_{(indole)}$-CH), 6.95 (s, 1 H, N-Ar-CH), 6.68 (d, *J* = 8.2 Hz, 1 H, Ar-CH), 4.52 (s, 1 H, OH), 3.78 (s, 3 H, NCH_3), 2.76–2.72 (m, 4 H, $CH_2CH_2CH_2$), 2.22 (s, 3 H, Ar-CH_3), 2.14–1.97 (m, 2 H, $CH_2CH_2CH_2$); ^{13}C NMR (101 MHz, $CDCl_3$) δ 151.4 (Ar-C_q-OH), 142.0 (Ar-C_q-C_q), 137.8 ($Ar_{(indole)}$-C_q), 129.0 (Ar-CH), 126.5 ($Ar_{(indole)}$-C_q), 125.7 (N-Ar-CH), 124.8 (Ar-CH), 123.1 (Ar-C_q-CH_3), 123.0 (N-Ar-CH-C_q), 121.3 ($Ar_{(indole)}$-CH), 120.7 ($Ar_{(indole)}$-CH), 118.4 ($Ar_{(indole)}$-CH), 114.3 (Ar-CH), 109.2 ($Ar_{(indole)}$-CH), 45.4 (C_q), 34.9 ($CH_2CH_2CH_2$), 32.6 (NCH_3), 17.0 ($CH_2CH_2CH_2$), 16.0 (Ar-CH_3). HRMS (+ESI) *m/z* calcd for $C_{20}H_{22}NO$ [M+H]: 292.1700, Found: 292.1696.

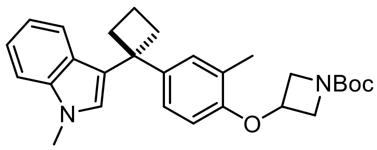

tert-Butyl-3-(2-methyl-4-(1-(1-methyl-1*H*-indol-3-yl)cyclobutyl)phenoxy)azetidine-1-carboxylate (12e)

A solution of **5e** (363 mg, 0.81 mmol) in DMF (2.7 mL) was added to a solution of NaH (60% dispersion in mineral oil, 32.7 mg, 0.74 mmol) in DMF (0.3 mL) at 0 °C. The reaction mixture was stirred for 30 min at 0 °C and then *tert*-butyl-3-bromoazetidine-1-carboxylate (293 mg, 1.24 mmol) was weighed into a flask, dissolved in DMF (0.1 mL) and added to the reaction mixture *via* syringe. DMF (0.5 mL) was added to the previous flask and then added to the reaction. The reaction mixture was then heated to 85 °C for 24 h. The reaction was cooled down to rt and water (25 mL) was added followed by EtOAc (50 mL). The layers were separated and the aqueous portion was extracted with EtOAc (3 × 20 mL). The organic extracts were combined and washed with sat. aq. NaCl (80 mL) and then dried over Na_2SO_4, filtered and concentrated under reduced pressure. Purification by flash column chromatography (5% to 20% EtOAc/pentane) afforded cyclobutane **12e** (262 mg, 94%) as a white powder. R_f = 0.08 (5% EtOAc/pentane); mp = 148 °C; IR (film)/cm^{-1} 2972, 2950, 1692 (C=O), 1502, 1417, 1364, 1241, 1229, 1167, 1146, 1132, 1012, 813, 742; ^{1}H NMR (400 MHz, $CDCl_3$) δ 7.40 (d, *J* = 7.9 Hz, 1 H, $Ar_{(indole)}$-CH), 7.28 (d, *J* = 6.2 Hz, 1 H, $Ar_{(indole)}$-CH), 7.19–7.12 (m, 3 H, 2 × $Ar_{(indole)}$-CH, Ar-CH), 7.00–6.95 (m, 2 H, Ar-CH, N-Ar-CH), 6.34 (d, *J* = 8.3 Hz, 1 H, Ar-CH), 4.82 (m, 1 H, OCH), 4.28 (dd, *J* = 9.2, 6.7 Hz, 2 H, C*H*HNC*H*H), 4.00 (dd, *J* = 9.2, 3.9 Hz, 2 H, CH*H*NCH*H*), 3.78 (s, 3 H, NCH_3), 2.75–2.72 (m, 4 H, $CH_2CH_2CH_2$), 2.20 (s, 3 H, Ar-CH_3), 2.13–1.99 (m, 2 H, $CH_2CH_2CH_2$), 1.46 (s, 9 H, 3 × CH_3); ^{13}C NMR (101 MHz, $CDCl_3$) δ 156.2 (C=O), 152.5 (Ar-C_q-OCH), 142.4 (Ar-C_q-C_q), 137.8 ($Ar_{(indole)}$-C_q), 129.3 (Ar-CH), 126.5 ($Ar_{(indole)}$-C_q), 126.3 (Ar-C_q-CH_3), 125.7 (N-Ar-CH), 124.2 (Ar-CH), 123.0 (N-Ar-CH-C_q), 121.3 ($Ar_{(indole)}$-CH), 120.7 ($Ar_{(indole)}$-CH), 118.4 ($Ar_{(indole)}$-CH), 110.2 (Ar-CH), 109.2 ($Ar_{(indole)}$-CH), 79.6 ($C_q(CH_3)_3$), 65.6 (OCH), 56.2 (CH_2NCH_2), 45.4 (C_q), 34.8 ($CH_2CH_2CH_2$), 32.6 (NCH_3), 28.4 ($C_q(CH_3)_3$), 17.0 ($CH_2CH_2CH_2$), 16.4 (Ar-CH_3); HRMS (+ESI) *m/z* calcd for $C_{28}H_{35}N_2O_3$ [M+H]: 447.2648, Found: 447.2645.

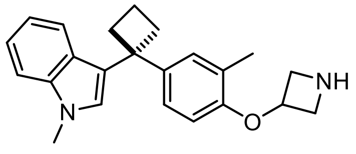

3-(1-(4-(Azetidin-3-yloxy)-3-methylphenyl)cyclobutyl)-1-methyl-1*H*-indole (1e)

A solution of HCl (4 M in dioxane, 1.40 mL, 5.60 mmol) was added to a solution of **12e** (252 mg, 0.56 mmol) in dioxane (2.5 mL) at 25 °C. The reaction mixture was stirred for 23 h and a solution of aqueous NaOH (1 M, 15 mL) was added until the colour of the mixture has changed, followed by the addition of Et_2O (30 mL). The layers were separated and the aqueous portion was extracted with Et_2O (4 × 30 mL). The organic extracts were combined, dried over Na_2SO_4, filtered and concentrated under reduced pressure. Purification by SCX column gave cyclobutane **1e** (188 mg, 97%) as a white solid. R_f = 0.18 (95:5:1

CH_2Cl_2/MeOH/NEt_3); mp = 46–50 °C; IR (film)/cm^{-1} 2939 (br, NH), 2864, 1499, 1483, 1465, 1372, 1247, 1236, 1148, 1128, 807, 736; ^{1}H NMR (400 MHz, $CDCl_3$) δ 7.42 (d, *J* = 8.0 Hz, 1 H, $Ar_{(indole)}$-CH), 7.28 (d, *J* = 6.0 Hz, 1 H, $Ar_{(indole)}$-CH), 7.20–7.12 (m, 3 H, 2 × $Ar_{(indole)}$-CH, Ar-CH), 7.01–6.96 (m, 2 H, Ar-CH, N-Ar-CH), 6.42 (d, *J* = 8.4 Hz, 1 H, Ar-CH), 4.95 (p, *J* = 6.2 Hz, 1 H, OCH), 3.93–3.89 (m, 2 H, C*H*HNC*H*H), 3.83–3.78 (m, 5 H, CH*H*NCH*H*, NCH_3), 2.74 (t, *J* = 7.5 Hz, 4 H, $CH_2CH_2CH_2$), 2.21 (s, 3 H, Ar-CH_3), 2.12–1.99 (m, 2 H, $CH_2CH_2CH_2$), 1.93 (br, s, 1 H, NH); ^{13}C NMR (101 MHz, $CDCl_3$) δ 152.9 (Ar-C_q-OCH), 142.0 (Ar-C_q-C_q), 137.7 ($Ar_{(indole)}$-C_q), 129.0 (Ar-CH), 126.5 ($Ar_{(indole)}$-C_q), 126.1 (Ar-C_q-CH_3), 125.7 (N-Ar-CH), 124.2 (Ar-CH), 123.1 (N-Ar-CH-C_q), 121.3 ($Ar_{(indole)}$-CH), 120.7 ($Ar_{(indole)}$-CH), 118.3 ($Ar_{(indole)}$-CH), 110.5 (Ar-CH), 109.2 ($Ar_{(indole)}$-CH), 70.5 (OCH), 55.1 (CH_2NCH_2), 45.4 (C_q), 34.9 ($CH_2CH_2CH_2$), 32.6 (NCH_3), 17.0 ($CH_2CH_2CH_2$), 16.4 (Ar-CH_3); HRMS (+ESI) *m/z* calcd for $C_{23}H_{27}N_2O$ [M+H] : 347.2123, Found: 347.2138.

Synthesis of 3,3-diaryloxetanes 3a–4a and diarylketones 3b–4b

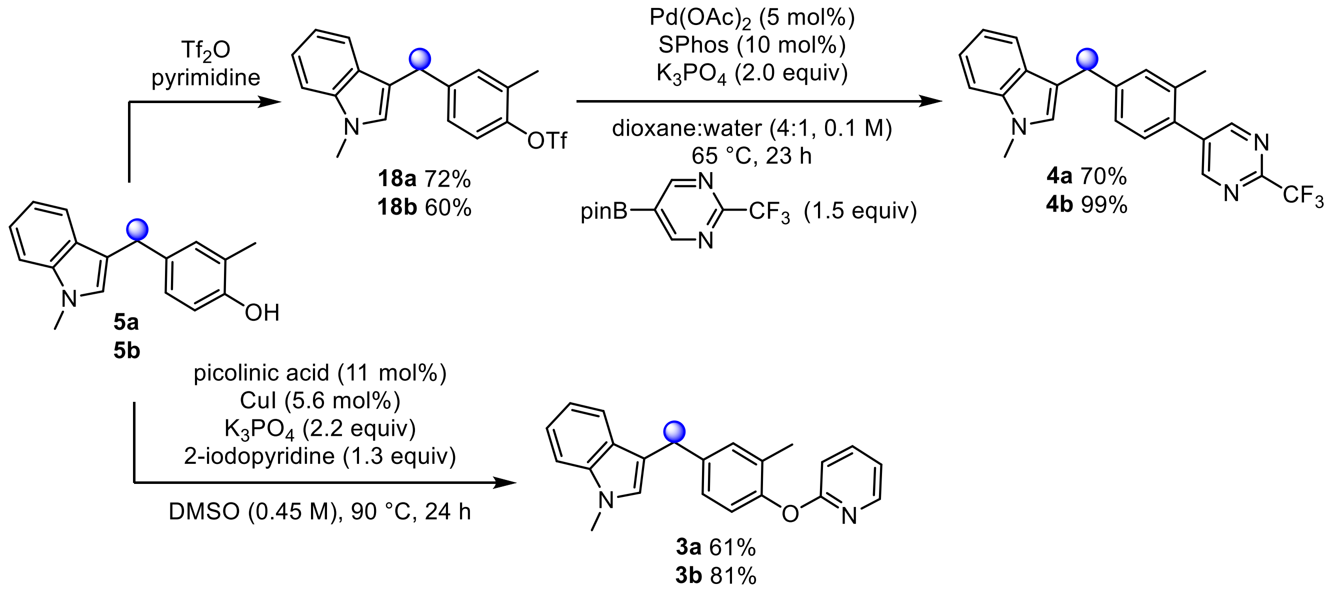

Figure S6.

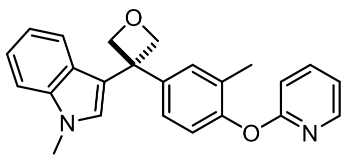

1-Methyl-3-(3-(3-methyl-4-(pyridin-2-yloxy)phenyl)oxetan-3-yl)-1*H*-indole (3a)

Using Ullmann conditions developed by Buchwald,[4] oxetane indole **5a** (78.4 mg, 0.27 mmol), picolinic acid (3.70 mg, 0.03 mmol), CuI (2.9 mg, 0.015 mmol) and K_3PO_4 (127 mg, 0.60 mmol) were added to a reaction vial. The reaction vial was evacuated and then refilled with nitrogen three times. 2-Iodopyridine (38 µL, 0.36 mmol) followed by DMSO (0.6 mL) were added via syringe into the vial. The reaction mixture was stirred at 90 °C for 23 h then cooled to rt. Water (20 mL) was added followed by EtOAc (20 mL). The layers were separated and the aqueous portion was extracted with EtOAc (2 × 20 mL). The combined organic extracts were washed with sat. aq. NaCl (2 × 20 mL), dried over Na_2SO_4, filtered and then concentrated under reduced pressure. Purification by flash column chromatography (25% EtOAc/hexane) afforded oxetane **3a** (60.9 mg, 61%) as a white solid. R_f = 0.16 (25% EtOAc/hexane); mp = 175–177 °C; IR (film)/cm^{-1} 3062, 3954, 3930, 2875, 1588, 1465, 1427, 1375, 1331, 1247, 1214, 1154, 982, 793, 739; ^{1}H NMR (400 MHz, $(CD_3)_2SO$) δ 8.09–8.08 (ddd, *J* = 5.0, 2.0, 0.7 Hz, 1 H, $Ar_{(py)}$-CH), 7.84–7.79 (ddd, *J* = 8.3, 7.2, 2.0, 1 H, $Ar_{(py)}$-CH), 7.44–7.42 (m, 2 H, 2 × $Ar_{(indole)}$-CH), 7.35 (d, *J* = 2.1 Hz, 1 H, Ar-CH), 7.20–7.14 (m, 3 H, $Ar_{(py)}$-CH, N-Ar-CH, Ar-CH), 7.08–7.05 (ddd, *J* = 7.2, 5.0, 0.7 Hz, 1 H, $Ar_{(py)}$-CH), 7.01–6.98 (m, 3 H, 2 × $Ar_{(indole)}$-CH, Ar-CH), 5.22 (m, 4 H, CH_2OCH_2), 3.75 (s, 3 H, N-CH_3), 2.06 (s, 3 H, Ar-CH_3); ^{13}C NMR (101 MHz, $(CD_3)_2SO$) δ 163.0 ($Ar_{(py)}$-C_q), 150.2 (Ar-C_q-$O_{(Py)}$), 147.3 ($Ar_{(py)}$-CH), 142.4 (Ar-C_q-C_q), 140.1 ($Ar_{(py)}$-CH), 137.1 ($Ar_{(indole)}$-C_q), 129.9 (Ar-C_q-CH_3), 128.9 (Ar-CH), 127.0 (N-Ar-CH), 125.4 ($Ar_{(indole)}$-C_q), 125.0 ($Ar_{(py)}$-CH), 121.7 ($Ar_{(indole)}$-CH), 121.3 (Ar-CH), 119.1 ($Ar_{(indole)}$-CH), 118.9 (Ar-CH), 118.8 ($Ar_{(py)}$-CH), 118.5 (N-Ar-CH-C_q), 110.7 ($Ar_{(indole)}$-CH), 110.0 ($Ar_{(indole)}$-CH), 82.8 (CH_2OCH_2), 45.9 (C_q (oxetane)), 32.4 (N-CH_3), 16.2 (Ar-CH_3). HRMS (ESI+) m/z calcd for $C_{24}H_{23}N_2O_2$ [M+H]: 371.1760, Found: 371.1769.

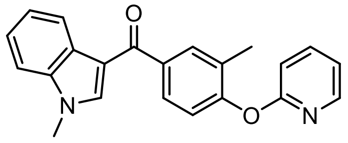

(1-Methyl-1*H*-indol-3-yl)(3-methyl-4-(pyridin-2-yloxy)phenyl)methanone (3b)

Using Ullmann conditions developed by Buchwald,[4] indole ketone **5b** (77.5 mg, 0.29 mmol), picolinic acid (3.7 mg, 0.03 mmol), CuI (2.9 mg, 0.015 mmol) and K_3PO_4 (127 mg, 0.60 mmol) were added to a reaction vial. The reaction vial was evacuated and then refilled with nitrogen three times. 2-Iodopyridine (38 µL, 0.36 mmol) then DMSO

(0.6 mL) were added via syringe. The reaction mixture was stirred at 90 °C for 24 h, cooled to rt. Water (20 mL) was added followed by EtOAc (15 mL). The layers were separated and the aqueous portion was extracted with EtOAc (2 × 15 mL). The combined organic extracts were washed with sat. aq. NaCl (30 mL), dried over Na_2SO_4, filtered and the solvent was removed under reduced pressure. Purification by flash column chromatography (20% EtOAc/hexane) afforded ketone **3b** (80.3 mg, 81%) as a white solid. R_f = 0.17 (30% EtOAc/hexane); mp = 123–124 °C; IR (film)/cm^{-1} 2930, 1615, 1572, 1521, 1462, 1427, 1370, 1341, 1283, 1237, 1192, 1167, 1157, 1141, 1128, 1076, 1038, 991, 938, 870, 843, 822, 771, 736; ^{1}H NMR (400 MHz, $CDCl_3$) δ 8.47–8.44 (m, 1 H $Ar_{(indole)}$-CH), 8.23–8.21 (dd, *J* = 5.1, 2.0 Hz, 1 H, $Ar_{(py)}$-CH), 7.79 (s, 1 H, Ar-CH), 7.75–7.70 (m, 2 H, Ar-CH, $Ar_{(py)}$-CH), 7.62 (s, 1 H, $N_{(indole)}$-Ar-CH), 7.40–7.34 (m, 3 H, 3 × $Ar_{(indole)}$-CH), 7.15 (d, *J* = 8.4 Hz, 1 H, Ar-CH), 7.08–7.02 (m, 1 H, $Ar_{(py)}$-CH), 6.96 (d, *J* = 8.1 Hz, 1 H, $Ar_{(py)}$-CH), 3.86 (s, 3 H, N-CH_3), 2.29 (s, 3 H, Ar-CH_3); ^{13}C NMR (101 MHz, $CDCl_3$) δ 189.8 (C=O), 163.2 ($Ar_{(py)}$-C_q), 154.9 (Ar-C_q-O-C_q), 147.7 ($Ar_{(py)}$-CH), 139.6 ($Ar_{(py)}$-CH), 137.7 ($N_{(indole)}$-Ar-CH), 137.5 ($Ar_{(indole)}$-C_q, Ar-C_q-CO), 132.0 (Ar-CH), 130.6 (Ar-C_q-CH_3), 127.9 (Ar-CH), 127.2 ($Ar_{(indole)}$-C_q), 123.5 ($Ar_{(indole)}$-CH), 122.7 ($Ar_{(indole)}$-CH), 122.6 ($Ar_{(indole)}$-CH), 121.0 ($Ar_{(py)}$-*C*H), 118.5 ($Ar_{(py)}$-CH), 115.5 ($N_{(indole)}$-Ar-CH-C_q), 111.2 (Ar-CH), 109.5 ($Ar_{(indole)}$-CH), 33.5 (N-CH_3), 16.5 (Ar-CH_3). HRMS (ESI+) m/z calcd for $C_{22}H_{19}N_2O_2$ [M+H]: 343.1447, Found: 343.1447.

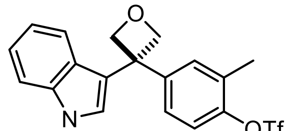

2-Methyl-4-(3-(1-methyl-1*H*-indol-3-yl)oxetan-3-yl)phenyl trifluoromethanesulfonate (18a)

Pyridine (0.24 mL, 3.00 mmol) followed by triflic anhydride (0.34 mL, 2.00 mmol) were added at 0 °C to a solution of indole oxetane **5a** (189 mg, 0.64 mmol) in CH_2Cl_2 (2.0 mL). The reaction mixture was stirred at rt for 4 h. Water (20 mL) was added followed by CH_2Cl_2 (25 mL). The layers were separated and the aqueous portion was extracted with CH_2Cl_2 (2 × 25 mL). The combined organic extracts were washed with sat. aq. NaCl (2 × 25 mL), dried over Na_2SO_4, filtered and solvent was removed under reduced pressure. Purification by flash column chromatography (30% Et_2O/pentane) afforded triflated oxetane **18a** (195 mg, 72%) as a brown solid. R_f = 0.16 (30% Et_2O/pentane); mp = 137–139 °C; IR (film)/cm^{-1} 3064, 2963, 2870, 1485, 1417, 1377, 1205, 1137, 1092, 988, 865, 745; ^{1}H NMR (400 MHz, $CDCl_3$) δ 7.63–7.60 (dt, *J* = 8.0, 1.0 Hz, 1 H, $Ar_{(indole)}$-CH), 7.38–7.34 (m, 2 H, $Ar_{(indole)}$-CH, Ar-CH), 7.31–7.27 (m, 1 H, $Ar_{(indole)}$-CH), 7.22–7.21 (m, 2 H, 2 × Ar-CH), 7.14–7.10 (ddd, *J* = 8.0, 7.0, 1.0 Hz, 1 H, $Ar_{(indole)}$-CH), 6.55 (s, 1 H, N-Ar-CH), 5.41 (d, *J* = 5.6 Hz, 2 H, C*H*HOC*H*H), 5.26 (d, *J* = 5.6 Hz, 2 H, CH*H*OCH*H*), 3.77 (s, 3 H, N-CH_3), 2.40 (s, 3 H, Ar-CH_3); ^{13}C NMR (101 MHz, $CDCl_3$) δ 147.0 (Ar-C_q-OTf), 145.9 (Ar-C_q-C_q), 137.6 ($Ar_{(indole)}$-C_q), 130.9 (Ar-C_q-CH_3), 130.2 (Ar-CH), 126.9 (N-Ar-CH), 125.91 ($Ar_{(indole)}$-C_q), 125.90 (Ar-CH), 122.2 ($Ar_{(indole)}$-CH), 121.1 (Ar-CH), 119.8 ($Ar_{(indole)}$-CH), 119.5 ($Ar_{(indole)}$-*C*H), 118.6 (q, J_{C-F} = 320.3 Hz, CF_3), 118.6 (N-Ar-CH-C_q), 109.6 ($Ar_{(indole)}$-CH), 83.9 (*C*H_2O*C*H_2), 46.4 (C_q (oxetane)), 32.7 (N-CH_3), 16.6 (Ar-CH_3); ^{19}F{^{1}H} NMR (377 MHz, $CDCl_3$) δ –73.8; HRMS (ESI+) m/z calcd for $C_{20}H_{19}F_3NO_4S$ [M+H]: 426.0987, Found: 426.0982.

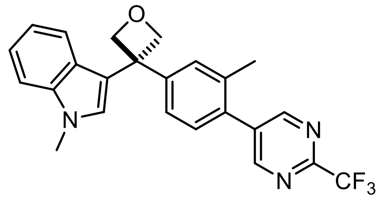

1-Methyl-3-(3-(3-methyl-4-(2-(trifluoromethyl)pyrimidin-5-yl)phenyl)oxetan-3-yl)-1*H*-indole (4a)

Triflate **18a** (44.2 mg, 0.10 mmol), pyrimidine pinacol ester (41.1 mg, 0.15 mmol), $Pd(OAc)_2$ (1.1 mg, 0.005 mmol), SPhos (4.1 mg, 0.010 mmol) and K_3PO_4 (42.4 mg, 0.20 mmol) were added to a reaction vial. The reaction vial was evacuated and then refilled with nitrogen three times. Dioxane/water (4:1, 1.0 mL) was added *via* syringe. The reaction mixture was stirred at 65 °C for 23 h then cooled to rt. Et_2O (20 mL) was added and the crude mixture was filtered through celite® and then concentrated under reduced pressure. Purification by flash column chromatography (10% to 20% EtOAc/hexane) afforded oxetane **4a** (29.4 mg, 70%) as a white solid. R_f = 0.29 (30% Et_2O/pentane); mp = 181–182 °C; IR (film)/cm^{-1} 3052, 2962, 2883, 1611, 1551, 1469, 1351, 1209, 1145, 1113, 970, 814, 742,

732; ^{1}H NMR (400 MHz, $CDCl_3$) δ 8.92 (s, 2 H, 2 × $Ar_{(pyrimidine)}$-CH), 7.66–7.63 (dt, *J* = 7.9, 1.0, 1 H, $Ar_{(indole)}$-CH), 7.42 (d, *J* = 1.1 Hz, 1 H, Ar-CH), 7.38–7.36 (m, 1 H, $Ar_{(indole)}$-CH), 7.32–7.27 (m, 2 H, Ar-CH, $Ar_{(indole)}$-CH), 7.23 (d, *J* = 8.0 Hz, 1 H, Ar-CH), 7.15–7.11 (ddd, *J* = 7.9, 6.9, 1.0 Hz, 1 H, $Ar_{(indole)}$-CH), 6.64 (s, 1 H, N-Ar-CH), 5.44 (d, *J* = 5.7 Hz, 2 H, C*H*HOC*H*H), 5.33 (d, *J* = 5.7 Hz, 2 H, CH*H*OCH*H*), 3.79 (s, 3 H, NCH_3), 2.34 (s, 3 H, Ar-CH_3); ^{13}C NMR (101 MHz, $CDCl_3$) δ 157.6 (2 × $Ar_{(pyrimidine)}$-CH), 155.0 (q, $^2J_{C\text{-}CF3}$ = 37.2 Hz, C_q-CF_3), 147.4 (Ar-C_q-C_q), 137.6 ($Ar_{(indole)}$-C_q), 137.0 ($C_{q(pyrimidine)}$), 135.9 (Ar-C_q-CH_3), 131.2 (Ar-C_q-$C_{q(pyrimidine)}$), 129.9 (Ar-CH), 129.1 (Ar-CH), 126.9 (N-Ar-CH), 125.9 ($Ar_{(indole)}$-C_q), 125.0 (Ar-CH), 122.1 ($Ar_{(indole)}$-CH), 119.8 ($Ar_{(indole)}$-CH), 119.7 (q, $^1J_{C\text{-}F}$ = 273.8 Hz, CF_3), 119.4 ($Ar_{(indole)}$-CH), 118.8 (N-Ar-CH-C_q), 109.6 ($Ar_{(indole)}$-CH), 83.9 (CH_2OCH_2), 46.5 (C_q), 32.8 (NCH_3), 20.5 (Ar-CH_3); ^{19}F{^{1}H} NMR (377 MHz, $CDCl_3$) δ –70.1; HRMS (+ESI) m/z calcd for $C_{24}H_{21}F_3N_3O$ [M+H]: 424.1637, Found: 424.1632.

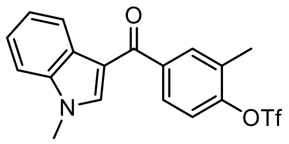

2-Methyl-4-(1-methyl-1*H*-indole-3-carbonyl)phenyl trifluoromethanesulfonate (18b)

Pyridine (87 µL, 1.08 mmol) followed by triflic anhydride (0.27 mL, 1.61 mmol) was added to a solution of ketone **5b** (142 mg, 0.54 mmol) in CH_2Cl_2 (1.0 mL) at 0 °C. The reaction mixture was stirred at rt for 3 h. Water (10 mL) was added followed by CH_2Cl_2 (10 mL). The layers were separated and the aqueous portion was extracted with CH_2Cl_2 (2 × 10 mL). The combined organic extracts were dried over Na_2SO_4, filtered and concentrated under reduced pressure. Purification by flash column chromatography (20% Et_2O/pentane) afforded triflated ketone **18b** (129 mg, 60%) as a white solid. R_f = 0.15 (20% Et_2O/pentane); mp = 157–159 °C; IR (film)/cm^{-1} 1612 (C=O), 1587, 1520, 1487, 1462, 1421, 1397, 1369, 1241, 1208, 1183, 1041, 1012, 908, 884, 823, 753, 726; ^{1}H NMR (400 MHz, $CDCl_3$) δ 8.44–8.40 (m, 1 H, $Ar_{(indole)}$-CH), 7.76 (d, *J* = 2.1 Hz, 1 H, Ar-CH), 7.70–7.68 (dd, 1 H, *J* = 8.4, 2.1 Hz, Ar-CH), 7.52 (s, 1 H, $Ar_{(indole)}$-CH), 7.42–7.35 (m, 4 H, 3 × $Ar_{(indole)}$-CH, Ar-CH), 3.88 (s, 3 H, N-CH_3), 2.47 (s, 3 H, Ar-CH_3); ^{13}C NMR (101 MHz, $CDCl_3$) δ 188.8 (C=O), 149.9 (Ar-C_q-OTf), 140.8 (Ar-C_q-C_q), 137.9 (N-Ar-CH), 137.6 ($Ar_{(indole)}$-C_q), 132.3 (Ar-CH), 131.2 (Ar-C_q-CH_3), 127.9 (Ar-CH), 127.0 ($Ar_{(indole)}$-C_q), 123.9 ($Ar_{(indole)}$-CH), 123.0 ($Ar_{(indole)}$-CH), 122.6 ($Ar_{(indole)}$-CH), 121.1 (Ar-CH), 118.6 (q, $J_{C\text{-}F}$ = 319.9 Hz, CF_3), 115.2 (N-Ar-CH-C_q), 109.7 ($Ar_{(indole)}$-CH), 33.6 (N-CH_3), 16.4 (Ar-CH_3); ^{19}F{^{1}H} NMR (377 MHz, $CDCl_3$) δ –73.7; HRMS (ESI+) m/z calcd for $C_{18}H_{15}F_3NO_4S$ [M+H]: 398.0674, Found: 398.0668.

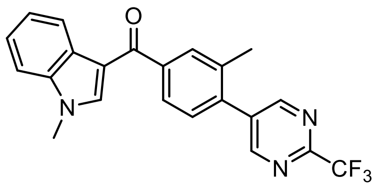

(1-Methyl-1*H*-indol-3-yl)(3-methyl-4-(2-(trifluoromethyl)pyrimidin-5-yl)phenyl)methanone (4b)

Triflate **18b** (64.1 mg, 0.161 mmol), pyrimidine pinacol ester (66.2 mg, 0.242 mmol), $Pd(OAc)_2$ (1.8 mg, 0.008 mmol), SPhos (6.6 mg, 0.016 mmol) and K_3PO_4 (65.4 mg, 0.308 mmol) were added to a reaction vial. The reaction vial was evacuated and then refilled with nitrogen three times. Dioxane/water (4:1, 1.6 mL) was added *via* syringe. The reaction mixture was stirred at 65 °C for 22 h then cooled to rt. Et_2O (20 mL) was added and the crude mixture was filtered through celite® and then concentrated under reduced pressure. Purification by flash column chromatography (20% Et_2O/pentane) afforded oxetane **4b** (63 mg, 99%) as a yellow solid. R_f = 0.29 (30% Et_2O/pentane); mp = 185–187 °C; IR (film)/cm^{-1} 3057, 1598 (C=O), 1521, 1464, 1367, 1350, 1231, 1186, 1141, 1118, 1106, 1077, 812, 773, 742, 733; ^{1}H NMR (400 MHz, $CDCl_3$) δ 8.94 (s, 2 H, 2 × $Ar_{(pyrimidine)}$-CH), 8.48–8.40 (m, 1 H, $Ar_{(indole)}$-CH), 7.81 (s, 1 H, Ar-CH), 7.76 (d, *J* = 7.6 Hz, 1 H, Ar-CH), 7.59 (s, 1 H, N-Ar-CH), 7.45–7.32 (m, 4 H, 3 × $Ar_{(indole)}$-CH, Ar-CH), 3.89 (s, 3 H, NCH_3), 2.40 (s, 3 H, Ar-CH_3); ^{13}C NMR (101 MHz, $CDCl_3$) δ 189.7 (C=O), 157.4 (2 × $Ar_{(pyrimidine)}$-CH), 155.4 (q, $^2J_{C\text{-}CF3}$ = 36.8 Hz, C_q-CF_3), 142.1 (Ar-C_q-CO), 137.8 (N-Ar-CH), 137.6 ($Ar_{(indole)}$-C_q), 136.6 ($C_{q\ (pyrimidine)}$), 136.1 (Ar-C_q-CH_3), 135.3 (Ar-$C_{q\ (pyrimidine)}$), 131.1 (Ar-CH), 129.7 (Ar-CH), 127.0 ($Ar_{(indole)}$-C_q), 126.8 (Ar-CH), 123.8 ($Ar_{(indole)}$-CH), 122.9 ($Ar_{(indole)}$-CH), 122.7 ($Ar_{(indole)}$-CH), 119.6 (q,

$J_{C\text{-}F}$ = 275.9 Hz, CF_3), 115.4 (N-Ar-CH-C_q), 109.7 ($Ar_{(indole)}$-CH), 33.6 (NCH_3), 20.3 (Ar-CH_3); $^{19}F\{^1H\}$ NMR (377 MHz, $CDCl_3$) δ –70.1; HRMS (+ESI) m/z calcd for $C_{22}H_{17}F_3N_3O$ [M+H]: 396.1324, Found: 396.1344.

Synthesis of 3,3-diaryloxetanes 7a–10a

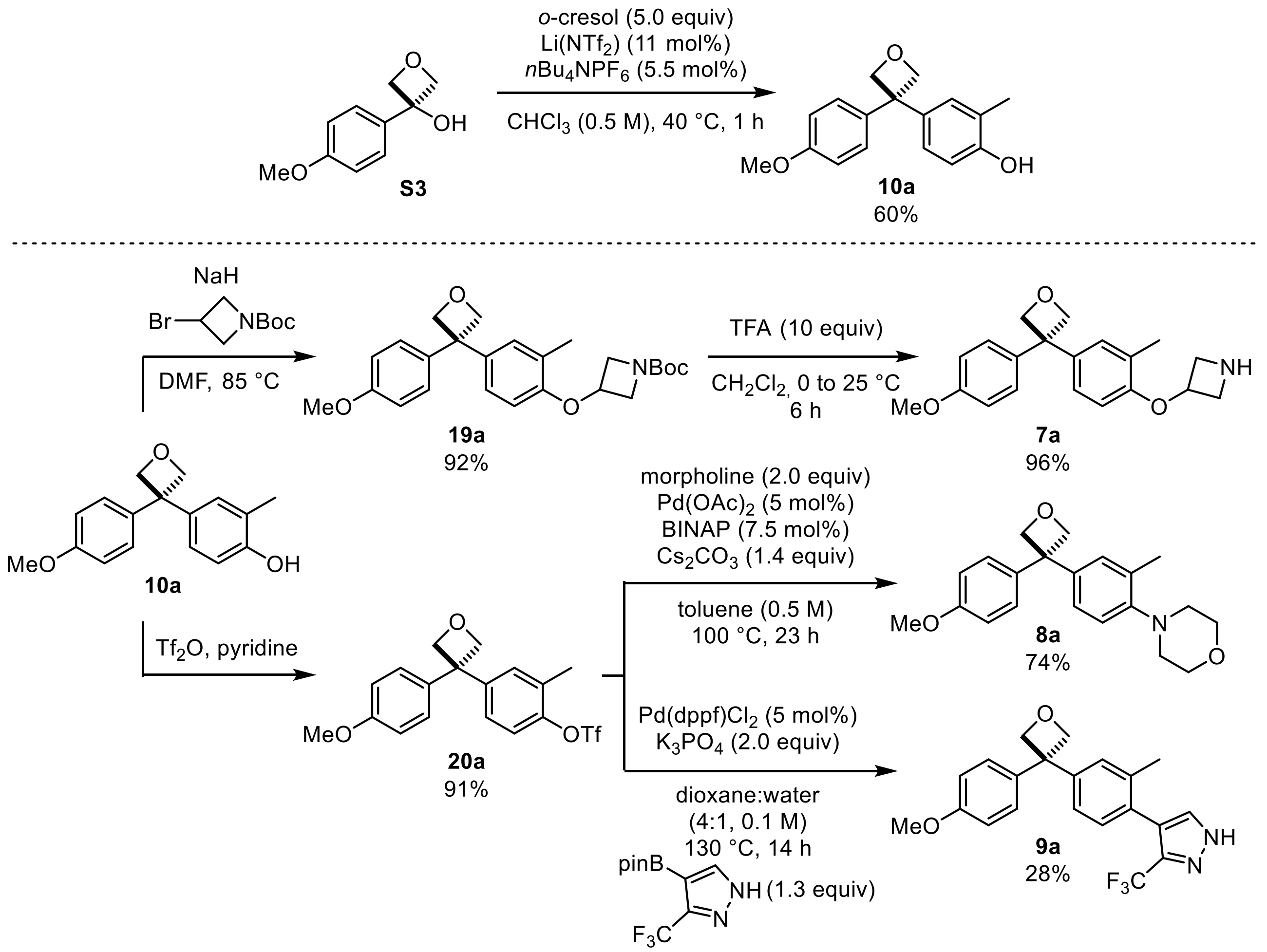

Figure F7.

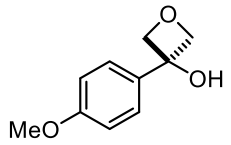

3-(4-Methyoxyphenyl) oxetan-3-ol (S3)

*n*BuLi (2.19 M in hexane, 5.50 mL, 12.0 mmol) was added dropwise over 5 min to a solution of 4-bromoanisole (1.63 mL, 13.0 mmol) in THF (40 mL) at –78 °C. The reaction mixture was stirred at –78 °C for 20 min. Oxetan-3-one (640 µL, 10.0 mmol) was added dropwise to the reaction mixture. Following a further 10 min at –78 °C the reaction mixture was warmed to 25 °C then quenched with water (40 mL). Et_2O (30 mL) was added and the layers were separated. The aqueous portion was extracted with Et_2O (2 × 30 mL). The organic extracts were combined, washed with sat. aq. NaCl (3 × 30 mL), dried over Na_2SO_4, filtered and concentrated under reduced pressure. Purification by flash column chromatography (45% EtOAc/hexane) afforded oxetanol **S3** (1.59 g, 88%) as a white solid. R_f = 0.29 (45% EtOAc/hexane); mp = 53–55 °C (52–53 °C)[6]. IR (film)/cm^{-1} 3313 (br, OH), 2995, 2949, 2882, 1607, 1581, 1511, 1462, 1440, 1300, 1236, 1217, 1178, 1028, 968, 950, 839, 815, 721, 663; ^{1}H NMR (400 MHz, $CDCl_3$) δ 7.51–7.49 (m, 2 H, 2 × Ar-CH), 6.97–6.95 (m, 2 H, 2 × Ar-CH), 4.93 (d, J = 6.2 Hz, 2 H, CH*H*OCH*H*), 4.90 (d, *J* = 6.2 Hz, 2 H, C*H*HOC*H*H), 3.84 (s, 3 H, OCH_3), 2.47 (s, 1 H, OH); ^{13}C NMR (101 MHz, $CDCl_3$) δ 159.3 (Ar-*C*$_q$-OMe), 134.5 (Ar-*C*$_q$-C$_q$), 125.9 (2 × Ar-CH), 114.0 (2 × Ar-CH), 85.5 (CH_2OCH_2), 75.7 (C$_q$ (oxetane)), 55.3 (OCH_3). The observed spectroscopic data for this compound was consistent with that previously reported.[5]

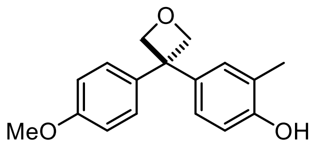

4-(3-(4-Methoxyphenyl) oxetan-3-yl)-2-methylphenol (10a)

Lithium bis(trifluoromethanesulfonimide) (158 mg, 0.55 mmol) and tetrabutylammonium hexafluorophosphate (107 mg, 0.28 mmol) were added to a solution of oxetanol **S3** (901 mg, 5.00 mmol) and 2-methylphenol (2.70 g, 25.0 mmol) in $CHCl_3$ (10 mL). The reaction mixture was stirred at 40 °C for 1 h and then sat. aq. $NaHCO_3$ (30 mL) was added followed by CH_2Cl_2 (30 mL). The aqueous portion was extracted with CH_2Cl_2 (2 × 30 mL). The organic extracts were combined, dried over Na_2SO_4, filtered and concentrated under reduced pressure. Purification by flash column chromatography (1% EtOAc/CH_2Cl_2) afforded oxetane **10a** (816 mg, 60%) as a white solid. R_f = 0.14 (1% EtOAc/CH_2Cl_2); mp = 140–143 °C (141–143 °C)[6]; IR (film)/cm^{-1} 3239 (br, OH), 2956, 1610, 1508, 1461, 1243, 1218, 1179, 1116, 1029, 973, 941, 901, 833, 764, 725; ^{1}H NMR (400 MHz, $CDCl_3$) δ 7.15–7.13 (m, 2 H, 2 × Ar-CH), 7.00 (d, *J* = 1.9 Hz, 1 H, Ar-CH), 6.90– 6.86 (m, 3 H, 3 × Ar-CH), 6.75 (d, *J* = 8.2 Hz, 1 H, Ar-CH), 5.20 (d, J = 5.6 Hz, 2 H, CH*H*OCH*H*), 5.18 (d, *J* = 5.6 Hz, 2 H, C*H*HOC*H*H), 4.67 (s, 1 H, OH), 3.82 (s, 1 H, OCH_3), 2.25 (s, 3 H, Ar-CH_3); ^{13}C NMR (101 MHz, $CDCl_3$) δ 158.1 (Ar-C_q-OMe), 152.5 (Ar-C_q-OH), 138.3 ($Ar_{(OMe)}$-C_q-C_q), 138.2 ($Ar_{(OH)}$-C_q-C_q), 129.1 (Ar-CH), 127.6 (2 × Ar-CH), 125.2 (Ar-CH), 123.9 (Ar-C_q-CH_3), 114.8 (Ar-CH), 113.9 (2 × Ar-CH), 85.0 (CH_2OCH_2), 55.3 (OCH_3), 50.3 (C_q (oxetane)), 15.9 (Ar-CH_3). The observed spectroscopic data for this compound was consistent with that previously reported.[5]

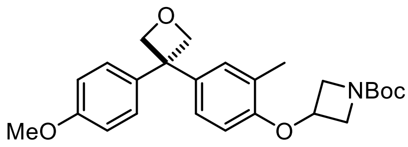

***tert*-Butyl-3-(4-(3-(4-methoxyphenyl)oxetan-3-yl)-2-methylphenoxy) azetidine-1-carboxylate (19a)**

A solution of oxetane **10a** (135 mg, 0.50 mmol) in DMF (1.3 mL) was added dropwise to a solution of NaH (60% dispersion in mineral oil, 26.0 mg, 0.65 mmol) in DMF (300 µL) at 0 °C. The reaction mixture was stirred for 30 min at 0 °C and then *tert*-butyl 3-bromoazetidine-1-carboxylate (236 mg, 1.00 mmol) was weighed into a flask, dissolved in DMF (100 µL) and added to the reaction mixture *via* syringe. DMF (900 µL) was added to the previous flask and then added to the reaction. The reaction mixture was then heated to 80 °C for 20 h. The reaction was cooled down to rt and then water (30 mL) was added followed by EtOAc (30 mL). The layers were separated and the aqueous portion was extracted with EtOAc (3 × 30 mL). The organic extracts were combined and washed with a solution of NaOH (1 M, 80 mL) and then dried over Na_2SO_4, filtered and concentrated under reduced pressure. Purification by flash column chromatography (40% Et_2O/pentane) afforded oxetane **19a** (195 mg, 92%) as a white solid. R_f = 0.20 (40% Et_2O/pentane); mp = 107–109 °C; IR (film)/cm^{-1} 2956, 2879, 1699 (C=O),1611, 1512, 1403, 1367, 1297, 1247, 1180, 1133, 1032, 988, 907, 831, 808, 728; ^{1}H NMR (400 MHz, $CDCl_3$) δ 7.14–7.07 (m, 2 H, 2 × Ar-CH), 7.07 (d, *J* = 2.4 Hz, 1 H, Ar-CH), 6.91–6.86 (m, 3 H, 3 × Ar-CH), 6.40 (d, *J* = 8.4 Hz, 1 H, Ar-CH), 5.19 (s, 4 H, C*H₂*OC*H₂*), 4.89–4.84 (m, 1 H, OCH), 4.33–4.29 (dd, *J* = 9.6, 6.4 Hz, 2 H, C*H*HNC*H*H), 4.03–4.00 (dd, *J* = 9.6, 4.2 Hz, 2 H, CH*H*NCH*H*), 3.82 (s, 3 H, OCH_3) , 2.24 (s, 3 H, Ar-CH_3), 1.46 (s, 9 H, 3 × CH_3); ^{13}C NMR (101 MHz, $CDCl_3$) δ 158.2 (Ar-C_q-OCH_3), 156.1 (C=O), 153.4 (Ar-C_q-OCH), 138.8 ($Ar_{(OMe)}$-C_q-C_q), 138.1 ($Ar_{(OCH)}$-C_q-C_q), 129.3 (Ar-CH), 127.6 (2 × Ar-CH), 127.2 (Ar-C_q-CH_3), 124.8 (Ar-CH), 113.9 (2 × Ar-CH), 110.5 (Ar-CH), 84.9 (CH_2OCH_2), 79.8 ($C_q(CH_3)_3$), 65.7 (OCH), 56.8 (CH_2NCH_2), 55.3 (OCH_3), 50.3 (C_q), 28.4 ($C_q(CH_3)_3$), 16.4 (Ar-CH_3); HRMS (+ESI) m/z calcd for $C_{25}H_{32}NO_5$ [M+H]: 426.2280, Found: 426.2281.

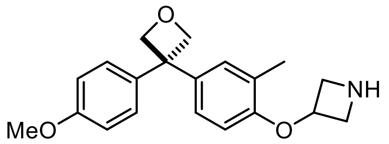

3-(4-(3-(4-Methoxyphenyl)oxetan-3-yl)-2-methylphenoxy) azetidine (7a)

Trifluoroacetic acid (128 µL, 1.67 mmol) was added to a solution of **19a** (71.0 mg, 0.17 mmol) in CH_2Cl_2 (0.9 mL) at 0 °C. The reaction mixture was stirred at 0 °C for 20 min and then at 25 °C for 24 h. A solution of aq. NaOH (1 M, 20 mL) was added and the reaction mixture was stirred for 5 min. EtOAc (20 mL) was then added and the layers were separated. The aqueous portion was extracted with EtOAc (4 × 20 mL). The organic extracts were combined, dried over Na_2SO_4, filtered and concentrated under reduced pressure. Purification by SCX column (see general procedure) gave amine **7a** (52.1 mg, 96%) as a white solid. R_f = 0.07 (95:5:1 CH_2Cl_2/Methanol/NEt_3); mp = 108–115 °C, IR (film)/cm^{-1} 2949, 2872, 1609, 1503, 1464, 1243, 1180, 1149, 1028, 988, 829, 806. ^1H NMR (400 MHz, $CDCl_3$) δ 7.14–7.12 (m, 2 H, 2 × Ar-CH), 7.08 (d, *J* = 2.4 Hz, 1 H, Ar-CH), 6.90–6.85 (m, 3 H, 3 × Ar-CH), 6.46 (d, *J* = 8.4 Hz, 1 H, Ar-CH), 5.19 (s, 4 H, C*H₂*OC*H₂*), 5.01–4.98 (m, 1 H, OCH), 3.99–3.95 (m, 2 H, C*H*HNC*H*H), 3.86–3.82 (m, 5 H, CH*H*NCH*H*, OCH_3), 2.23 (s, 3 H, Ar-CH_3), 1.94 (br, s, 1 H, NH); ^{13}C NMR (101 MHz, $CDCl_3$) δ 158.2 (Ar-*C_q*-OCH_3), 153.7 (Ar-*C_q*-OCH), 138.4 ($Ar_{(OMe)}$-*C_q*-C_q), 138.2 ($Ar_{(OCH)}$-*C_q*-C_q), 129.2 (Ar-CH), 127.6 (2 × Ar-CH), 127.1 (Ar-*C_q*-CH_3), 124.8 (Ar-CH), 113.9 (2 × Ar-CH), 110.8 (Ar-CH), 84.9 (*C*H_2O*C*H_2), 70.2 (OCH), 55.3 (OCH_3), 54.8 (*C*H_2N*C*H_2), 50.3 (C_q), 16.4 (Ar-CH_3); HRMS (+ESI) m/z calcd for $C_{20}H_{24}NO_3$ [M+H]:326.1756, Found: 326.1770.

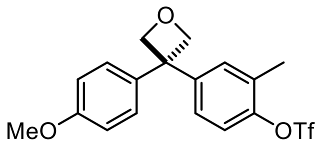

4-(3-(4-Methoxyphenyl)oxetan-3-yl)-2-methylphenyl trifluoromethanesulfonate (20a)

Pyridine (800 µL, 10.0 mmol) followed by triflic anhydride (1.00 mL, 6.00 mmol) was added to a solution of oxetane **10a** (1.35 g, 4.99 mmol) in CH_2Cl_2 (10 mL) at 0 °C. The reaction mixture was stirred at 25 °C for 3 h. Water (30 mL) was added followed by CH_2Cl_2 (30 mL) and the layers were separated. The aqueous portion was extracted with CH_2Cl_2 (3 × 20 mL). The organic extracts were combined, washed with sat. aq. NaCl (60 mL), dried over Na_2SO_4, filtered and concentrated under reduced pressure. Purification by flash column chromatography (20% EtOAc/hexane) afforded triflated oxetane **20a** (1.83 g, 91%) as a white solid. R_f = 0.23 (20% EtOAc/hexane); mp = 87–91 °C; IR (film)/cm^{-1} 2945, 2877, 2837, 1610, 1579, 1512, 1498, 1409, 1249, 1202, 1135, 1088, 1030, 997, 907, 893, 867, 829, 771, 729,710; ^1H NMR (400 MHz, $CDCl_3$) δ 7.22–7.20 (m, 2 H, 2 × Ar-CH), 7.11–7.07 (m, 3 H, 3 × Ar-CH), 6.94–6.90 (m, 2 H, 2 × Ar-CH), 5.24 (d, *J* = 5.7 Hz, 2 H, C*H*HOC*H*H), 5.16 (d, *J* = 5.7 Hz, 2 H, CH*H*OCH*H*), 3.83 (s, 3 H, OCH_3), 2.38 (s, 3 H, Ar-CH_3); ^{13}C NMR (101 MHz, $CDCl_3$) δ 158.4 (Ar-*C_q*-OMe), 147.0 (Ar-C_q-OTf), 146.5 (Ar-*C_q*-C_q), 136.8 (Ar-*C_q*-C_q), 131.0 (Ar-CH), 130.2 (Ar-*C_q*-CH_3) 127.6 (2 × Ar-CH), 125.8 (Ar-CH), 121.3 (Ar-CH), 118.6 (q, $J_{C\text{-}F}$ = 320.3 Hz, CF_3), 114.1 (2 × Ar-CH), 84.4 (*C*H_2O*C*H_2), 55.3 (OCH_3), 50.6 (C_q (oxetane)), 16.5 (Ar-CH_3); $^{19}F\{^1H\}$ NMR (377 MHz, $CDCl_3$) δ –73.8; HRMS (ESI-) m/z calcd for $C_{18}H_{16}O_5SF_3$ [M-H]: 401.0671, Found: 401.0674.

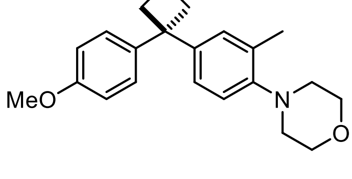

4-(4-(3-(4-Methoxyphenyl)oxetan-3-yl)-2-methylphenyl) morpholine (8a)

Using conditions developed by Buchwald,[6] triflated oxetane **20a** (50.0 mg, 0.12 mmol), $Pd(OAc)_2$ (1.3 mg, 0.006 mmol), BINAP (5.6 mg, 0.009 mmol) and Cs_2CO_3 (55.4 mg, 0.17 mmol) were added to a reaction vial. The reaction vial was evacuated and then refilled with nitrogen three times. Morpholine (20 µL, 0.24 mmol) followed by toluene (240 µL) were added via syringe into the vial. The reaction mixture was stirred at 100 °C for 23 h then cooled to rt. Et_2O (25 mL) was added and the reaction mixture was filtered through celite®. The organic layer was then washed with a solution of aq. NaOH (1 M, 3 × 20 mL), dried over Na_2SO_4, filtered and concentrated under reduced pressure. Purification by flash column

chromatography (10% to 20% EtOAc/pentane) afforded morpholine substituted oxetane **8a** (30 mg, 74%) as a white solid. R_f = 0.21 (20% EtOAc/pentane); mp = 133–135 °C; IR (film)/cm^{-1} 2962, 2930, 2895, 2862, 2813, 1608, 1580, 1513, 1500, 1439, 1298, 1251, 1228, 1184, 1114, 1068, 993, 934, 829, 799; ^{1}H NMR (400 MHz, $CDCl_3$) δ 7.17–7.13 (m, 2 H, 2 × Ar-CH), 7.08 (d, *J* = 1.93 Hz, 1 H, Ar-CH), 7.01–6.96 (m, 2 H, 2 × Ar-CH), 6.92–6.88 (m, 2 H, 2 × Ar-CH), 5.23-5.20 (m, 4 H, C*H₂*OC*H₂*), 3.87–3.85 (m, 4 H, C*H₂*CH₂NCH₂C*H₂*), 3.82 (s, 3 H, OCH_3), 2.93–2.91 (m, 4 H, C*H₂*NC*H₂*), 2.32 (s, 3 H, Ar-CH_3); ^{13}C NMR (101 MHz, $CDCl_3$) δ 158.1 (Ar-C_q-OCH_3), 149.7 (Ar-C_q-N), 140.9 ($Ar_{(OMe)}$-C_q-C_q), 138.2 ($Ar_{(N)}$-C_q-C_q), 132.7 (Ar-C_q-CH_3), 129.3 (Ar-CH), 127.6 (2 × Ar-CH), 124.7 (Ar-CH), 118.8 (Ar-CH), 113.9 (2 × Ar-CH), 84.8 (*C*H₂O*C*H₂), 67.4 (CH₂*C*H₂OCH₂*C*H₂), 55.3 (OCH_3), 52.2 (*C*H₂N*C*H₂), 50.4 (C_q), 18.1 (Ar-CH_3); HRMS (+ESI) m/z calcd for $C_{21}H_{26}NO_3$ [M+H]: 340.1913, Found: 340.1920.

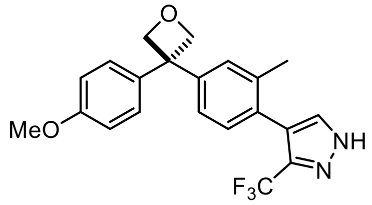

4-(4-(3-(4-Methoxyphenyl)oxetan-3-yl)-2-methylphenyl)-3-(trifluoromethyl)-1*H*-pyrazole (9a)

Triflated oxetane **20a** (121 mg, 0.30 mmol), pyrazole pinacol ester (87.0 mg, 0.33 mmol), Pd(dppf)Cl_2 (11.0 mg, 0.015 mmol) and K_3PO_4 (127 mg, 0.60 mmol) were added to a reaction vial. The reaction vial was evacuated and then refilled with nitrogen three times. Dioxane/water (4:1, 3.0 mL) was added via syringe into the vial. The reaction mixture was stirred at 130 °C for 14 h then cooled to rt. EtOAc (15 mL) was added and the reaction mixture was filtered through celite® and then concentrated under reduced pressure. Purification by flash column chromatography (30% EtOAc/hexane) afforded oxetane **9a** (33 mg, 28%) as a white solid. R_f = 0.15 (30% EtOAc/hexane); mp = 175–177 °C; IR (film)/cm^{-1} 3182 (br, NH), 2954, 2876, 1613, 1512, 1475, 1271, 1250, 1126, 1107, 1079, 1032, 1028, 980, 940, 828, 812, 750; ^{1}H NMR (400 MHz, $CDCl_3$) δ 7.61 (d, *J* = 1.1 Hz, 1 H, N-Ar-CH), 7.21–7.15 (m, 4 H, 4 × Ar-CH), 7.05–7.03 (dd, *J* = 8.4, 2.2 Hz, 1 H, Ar-CH), 6.94–6.91 (m, 2 H, 2 × Ar-CH), 5.28 (d, *J* = 5.6 Hz, 2 H, C*H*HOC*H*H), 5.25 (d, *J* = 5.6 Hz, 2 H, CH*H*OCH*H*), 3.83 (s, 3 H, OCH_3), 2.19 (s, 3 H, Ar-CH_3); ^{13}C NMR (101 MHz, $CDCl_3$) δ 158.3 (Ar-C_q-OMe), 146.1 (Ar-C_q-$C_{q(pyrazole)}$), 140.3 (q, $J_{C\text{-}F}$ = 35.9 Hz, C_q-CF_3), 137.7 (Ar-C_q-C_q), 137.5 (Ar-C_q-C_q), 131.0 (Ar-CH), 130.2 (N-Ar-CH), 128.02 (Ar-C_q-CH_3), 128.00 (Ar-CH), 127.6 (2 × Ar-CH), 123.7 (Ar-CH), 121.7 (q, $J_{C\text{-}F}$ = 270.0 Hz, CF_3), 119.9 (N-Ar-CH-C_q), 114.0 (2 × Ar-CH), 84.7 (*C*H₂O*C*H₂), 55.3 (OCH_3), 50.7 (C_q (oxetane)), 20.4 (Ar-CH_3); ^{19}F{^{1}H} NMR (377 MHz, $CDCl_3$) δ –60.2; HRMS (ESI-) m/z calcd for $C_{21}H_{18}N_2O_2F_3$ [M-H]: 387.1320, Found: 387.1328.

Synthesis of diarylketones 7b–10b

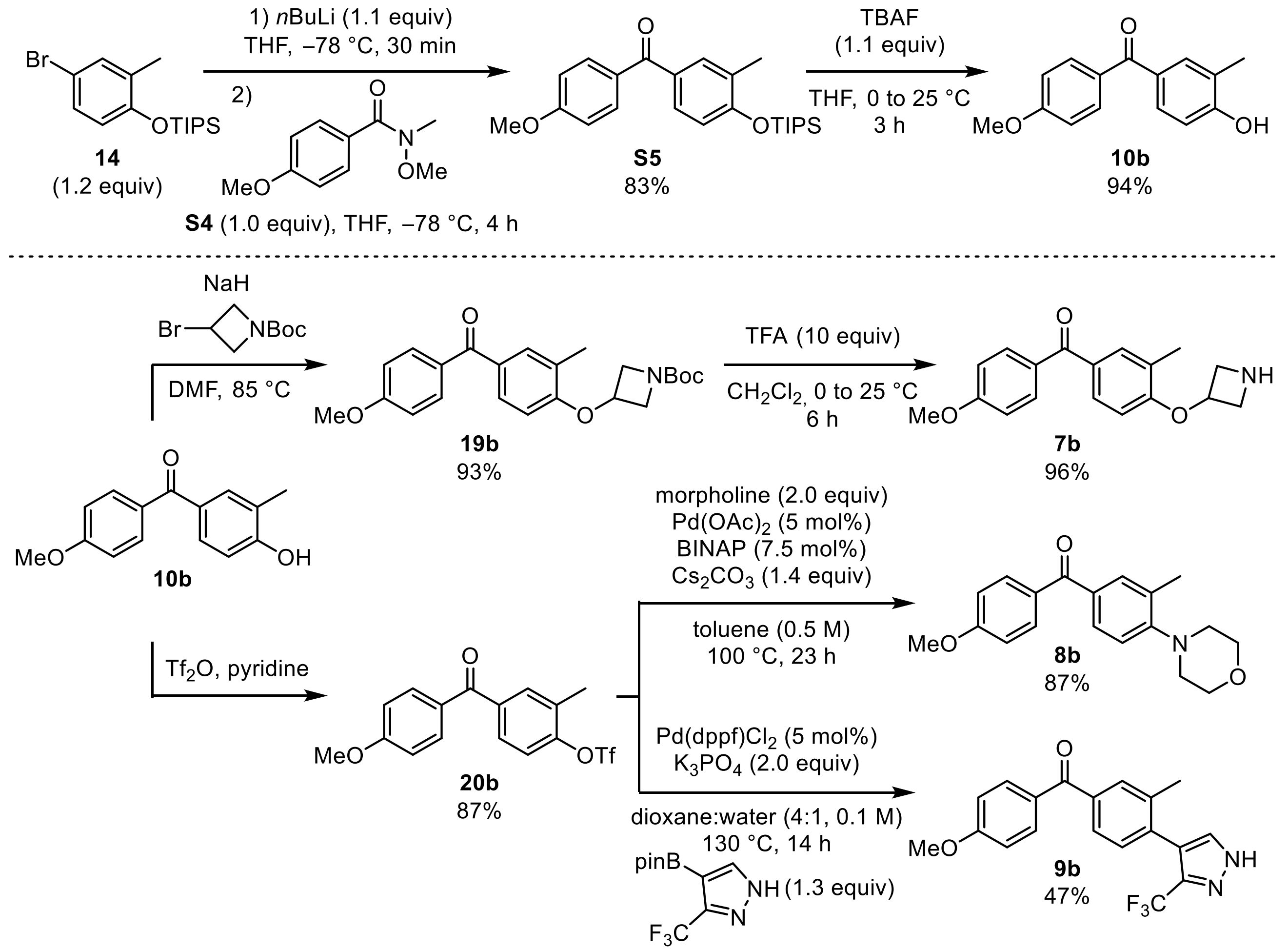

Figure F8.

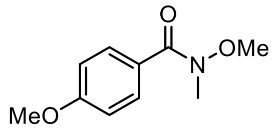

N,4-Dimethoxy-*N*-methylbenzamide (S4)

Pyridine (510 µL, 6.30 mmol) was added to the solution of 4-methoxy benzoyl chloride (512 mg, 3.00 mmol) and *N*,*O*-dimethylhydroxylamine (307 mg, 1.05 mmol) in CH_2Cl_2 (5.0 mL) at 0 °C. The reaction mixture was warmed to rt and then stirred for 15 h. A solution of HCl (3 M, 10 mL) was added dropwise, followed by water (30 mL), and the reaction mixture was then extracted with CH_2Cl_2 (5 × 40 mL). The organic extracts were combined, dried over Na_2SO_4, filtered and concentrated under reduced pressure. Purification by flash column chromatography (40% Et_2O/pentane) afforded Weinreb amide **S4** (577 mg, 99%) as yellow oil. R_f = 0.15 (40% Et_2O/pentane); IR (film)/cm^{-1} 3470, 2936, 2840, 1632 (C=O), 1606, 1575, 1511, 1460, 1420, 1372, 1250, 1215, 1171, 1112, 1027, 994, 976, 840, 796, 755, 702, 676; ^{1}H NMR (400 MHz, $CDCl_3$) δ 7.76–7.72 (m, 2 H, 2 × Ar-CH), 6.93–6.89 (m, 2 H, 2 × Ar-CH), 3.86 (s, 3 H, Ar-OCH_3), 3.57 (s, 3 H, OCH_3), 3.36 (s, 3 H, NCH_3); ^{13}C NMR (101 MHz, $CDCl_3$) δ 169.1 (C=O), 161.3 (Ar-C_q-OCH_3), 130.3 (2 × Ar-CH), 125.8 (Ar-C_q-CO), 113.0 (2 × Ar-CH), 60.6 (OCH_3), 55.1 (Ar-OCH_3), 33.7 (NCH_3). The observed spectroscopic data for this compound was consistent with that previously reported.[7]

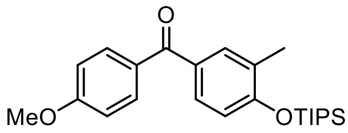

(4-Methoxyphenyl)(3-methyl-4 ((triisopropylsilyl)oxy)phenyl)methanone (S5)

*n*BuLi (2.26 M in hexane, 1.24 mL, 2.82 mmol) was added dropwise over 5 min to a solution of **14** (1.06 g, 3.07 mmol) in THF (5.2 mL) at –78 °C. The reaction mixture was stirred at –78 °C for 40 min. A solution of amide **S4** (505 mg, 2.59 mmol) in THF (5.0 mL) was added dropwise to the reaction mixture. Following a further 3 h 30 min at –78 °C the reaction mixture was warmed to 25 °C then quenched with water (50 mL). Et_2O (50 mL) was added and the layers were separated. The aqueous portion was extracted with Et_2O (3 × 50 mL). The organic extracts were combined, dried over Na_2SO_4, filtered and concentrated under reduced pressure. Purification by flash column chromatography (10% Et_2O/hexane) afforded ketone **S5** (851 mg, 83%) as a colourless oil. R_f = 0.17 (10% Et_2O/pentane); IR (film)/cm^{-1} 2945, 2866, 1646, 1598 (C=O), 1497, 1462, 1304, 1275, 1250, 1168, 968, 916, 881, 844, 823, 707, 683; ^{1}H NMR (400 MHz, $CDCl_3$) δ 7.81–7.78 (m, 2 H, 2 × Ar-CH), 7.65 (d, *J* = 2.4 Hz, 1 H, Ar-CH), 7.54 (dd, *J* = 8.4, 2.4 Hz, 1 H, Ar-CH), 6.98–6.95 (m, 2 H, 2 × Ar-CH), 6.83 (d, *J* = 8.4 Hz, 1 H, Ar-CH), 3.89 (s, 3 H, OCH_3), 2.29 (s, 3 H, Ar-CH_3), 1.39–1.29 (m, 3 H, 3 × C*H*$(CH_3)_2$), 1.14 (d, *J* = 7.5 Hz, 18 H, 3 × CH(C*H*$_3$)$_2$); ^{13}C NMR (101 MHz, $CDCl_3$) δ 194.8 (C=O), 162.7 (Ar-C_q-OCH_3), 158.2 (Ar-C_q-OTIPS), 133.1 (Ar-CH), 132.2 (2 × Ar-CH), 130.9 (Ar$_{(OMe)}$-C_q-CO), 130.6 (Ar$_{(OTIPS)}$-C_q-CO), 129.6 (Ar-CH), 128.6 (Ar-C_q-CH_3), 117.2 (Ar-CH), 113.4 (2 × Ar-CH), 55.4 (OCH_3), 18.0 (Ar-CH_3), 17.0 (3 × *C*H$(CH_3)_2$), 13.0 (3 × CH(*C*H$_3$)$_2$); HRMS (+ESI) m/z calcd for $C_{24}H_{35}O_3Si$ [M+H]: 399.2355, Found: 399.2364.

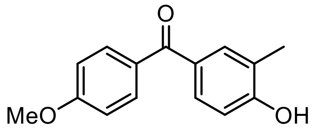

(4-Hydroxy-3-methylphenyl)(4-methoxyphenyl)methanone (10b)

A solution of TBAF (1 M in THF, 2.70 mL, 2.70 mmol) was added dropwise to a solution of **S5** (831 mg, 2.08 mmol) in THF (6.9 mL) at 0 °C. The reaction mixture was stirred for 30 min at 0 °C and then 4 h at 25 °C. Water (60 mL) was then added followed by Et_2O (60 mL). The layers were separated and the aqueous portion was extracted with Et_2O (3 × 60 mL). The organic extracts were combined, dried over Na_2SO_4, filtered and concentrated under reduced pressure. Purification by flash column chromatography (40% Et_2O/pentane) afforded ketone **10b** (476 mg, 94%) as a pink solid. R_f = 0.22 (0.15% Et_2O/pentane); mp = 186–187 °C; IR (film)/cm^{-1} 3100 (br, OH), 1630 (C=O), 1602, 1579, 1560, 1505, 1459, 1449, 1316, 1282, 1255, 1172, 1150, 1108, 1025, 904, 863, 834, 757, 729, 679; ^{1}H NMR (400 MHz, DMSO-*d$_6$*) δ 10.26 (s, 1 H, OH), 7.70–7.66 (m, 2 H, 2 × Ar-CH), 7.51 (d, *J* = 2.2 Hz, 1 H, Ar-CH), 7.45–7.42 (dd, *J* = 8.4, 2.2 Hz, 1 H, Ar-CH), 7.08–7.04 (m, 2 H, 2 × Ar-CH), 6.89 (d, *J* = 8.4 Hz, 1 H, Ar-CH), 3.85 (s, 3 H, OCH_3), 2.17 (s, 3 H, Ar-CH_3); ^{13}C NMR (101 MHz, DMSO-*d$_6$*) δ 193.2 (C=O), 162.2 (Ar-C_q-OCH_3), 159.7 (Ar-C_q-OH), 132.6 (Ar-CH), 131.6 (2 × Ar-CH), 130.4 (Ar$_{(OMe)}$-C_q-CO), 129.7 (Ar-CH), 128.4 (Ar$_{(OH)}$-C_q-CO), 124.0 (Ar-C_q-CH_3), 114.1 (Ar-CH), 113.6 (2 × Ar-CH), 55.4 (OCH_3), 15.9 (Ar-CH_3); HRMS (+ESI) m/z calcd for $C_{15}H_{15}O_3$ [M+H]: 243.1021, Found: 243.1021.

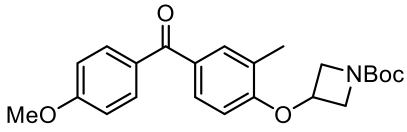

***tert*-Butyl-3-(4-(4-methoxybenzoyl)-2-methylphenoxy) azetidine-1-carboxylate (19b)**

A solution of ketone **10b** (75.0 mg, 0.31 mmol) in DMF (800 µL) was added dropwise to a solution of NaH (60% dispersion in mineral oil, 16.0 mg, 0.40 mmol) in DMF (300 µL) at 0 °C. The reaction mixture was stirred for 30 min at 0 °C and then *tert*-butyl 3-bromoazetidine-1-carboxylate (146 mg, 0.62 mmol) was weighed into a flask, dissolved in DMF (100 µL) and added to the reaction mixture *via* syringe. DMF (400 µL) was added to the previous flask and then added to the reaction. The reaction mixture was heated to 80 °C for 20 h. The reaction was cooled down to rt and a solution of NaOH (1 M, 20 mL) was added followed by Et_2O (30 mL). The layers were separated and the aqueous portion was extracted with Et_2O

(3 × 30 mL). The organic extracts were combined, dried over Na_2SO_4, filtered and concentrated under reduced pressure. Purification by flash column chromatography (40% Et_2O/pentane) afforded ketone **19b** (115 mg, 93%) as a white solid. R_f = 0.43 (25% EtOAc/pentane); mp = 107–108 °C; IR (film)/cm^{-1} 2973, 1702 (C=O), 1647 (C=O), 1601, 1403, 1313, 1254, 1171,1123, 1031, 769; ^{1}H NMR (400 MHz, $CDCl_3$) δ 7.81–7.77 (m, 2 H, 2 × Ar-CH), 7.66 (s, 1 H, Ar-CH), 7.60–7.57 (dd, *J* = 8.4, 2.2 Hz, 1 H, Ar-CH), 6.99–6.95 (m, 2 H, 2 × Ar-CH), 6.49 (d, *J* = 8.4 Hz, 1 H, Ar-CH), 4.99–4.94 (tt, *J* = 6.4, 4.1 Hz, 1 H, OCH), 4.38–4.34 (dd, *J* = 10.2, 6.4 Hz, 2 H, C*H*HNC*H*H), 4.07–4.04 (dd, *J* = 10.2, 4.1 Hz, 2 H, CH*H*NCH*H*), 3.90 (s, 3 H, OCH_3), 2.29 (s, 3 H, Ar-CH_3), 1.47 (s, 9 H, 3 × CH_3); ^{13}C NMR (101 MHz, $CDCl_3$) δ 194.5 (C=O), 162.9 (Ar-C_q-OCH_3), 157.9 (Ar-C_q-OCH), 156.1 ($C=O_{(boc)}$), 133.0 (Ar-CH), 132.2 (2 × Ar-CH), 131.2 ($Ar_{(OMe)}$-C_q-CO), 130.6 ($Ar_{(OCH)}$-C_q-CO), 129.8 (Ar-CH), 127.0 (Ar-C_q-CH_3), 113.4 (2 × Ar-CH), 109.8 (Ar-CH), 80.0 ($C_q(CH_3)_3$), 65.9 (OCH), 56.5 (CH_2NCH_2), 55.5 (OCH_3), 28.4 ($C_q(CH_3)_3$), 16.2 (Ar-CH_3); HRMS (+ESI) m/z calcd for $C_{23}H_{28}NO_5$ [M+H]: 398.1967, Found: 398.1967.

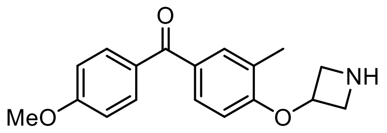

(4-(Azetidin-3-yloxy)-3-methylphenyl)(4-methoxyphenyl) methanone (7b)

Trifluoroacetic acid (220 µL, 2.90 mmol) was added to a solution of **19b** (115 mg, 0.29 mmol) in CH_2Cl_2 (1.5 mL) at 0 °C and stirred for 30 min. The reaction mixture was then stirred at 25 °C for 18 h. A solution of NaOH (1 M, 20 mL) was added and the reaction mixture was stirred for 5 min. The mixture was extracted with Et_2O (2 × 20 mL) followed by EtOAc (2 × 20 mL). The organic extracts were combined, dried over Na_2SO_4, filtered and concentrated under reduced pressure to afford amine **7b** (82.9 mg, 96%) as a pale yellow sticky solid. R_f = 0.04 (95:5:1 CH_2Cl_2/Methanol/NEt_3); mp = 99–103 °C; IR (film)/cm^{-1} 2949, 2868, 1640 (C=O), 1598, 1572, 1509, 1497, 1417, 1292, 1270, 1248, 1171,1119, 1029, 767, 729; ^{1}H NMR (400 MHz, $CDCl_3$) δ 7.81–7.77 (m, 2 H, 2 × Ar-CH), 7.66 (d, *J* = 1.3 Hz, 1 H, Ar-CH), 7.60–7.57 (dd, *J* = 8.4, 2.2 Hz, 1 H, Ar-CH), 6.98–6.95 (m, 2 H, 2 × Ar-CH), 6.54 (d, *J* = 8.4 Hz, 1 H, Ar-CH), 5.13–5.07 (m, 1 H, OCH), 4.03 (m, 2 H, C*H*HNC*H*H), 3.90–3.86 (m, 5 H, CH*H*NCH*H*, OCH_3), 2.29 (s, 3 H, Ar-CH_3), 1.92 (br, s, 1 H, NH); ^{13}C NMR (101 MHz, $CDCl_3$) δ 194.6 (C=O), 162.8 (Ar-C_q-OCH_3), 158.4 (Ar-C_q-OCH), 132.9 (Ar-CH), 132.2 (2 × Ar-CH), 130.8 ($Ar_{(OMe)}$-C_q-CO), 130.7 ($Ar_{(OCH)}$-C_q-CO), 129.9 (Ar-CH), 126.8 (Ar-C_q-CH_3), 113.4 (2 × Ar-CH), 110.1 (Ar-CH), 70.6 (OCH), 55.5 (OCH_3), 54.6 (CH_2NCH_2), 16.3 (Ar-CH_3); HRMS (+ESI) m/z calcd for $C_{18}H_{20}NO_3$ [M+H]: 298.1443, Found: 298.1453.

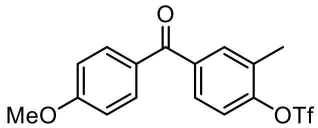

4-(4-Methoxybenzoyl)-2-methylphenyl trifluoromethanesulfonate (20b)

Pyridine (0.61 mL, 7.50 mmol) followed by triflic anhydride (0.84 mL, 5.00 mmol) was added to a solution of ketone **10b** (606 mg, 2.50 mmol) in CH_2Cl_2 (5.0 mL) at 0 °C. The reaction mixture was stirred at rt for 4 h. Water (20 mL) was added followed by extraction with CH_2Cl_2 (3 × 15 mL). The combined organic layers were washed with water (30 mL), then sat. aq. NaCl (2 × 25 mL), dried (Na_2SO_4), filtered and the solvent removed under reduced pressure to afford triflate **20b** (814 mg, 87%) as a brown solid. R_f = 0.58 (40% Et_2O/pentane); mp = 45–47 °C; IR (film)/cm^{-1} 2988, 1651 (C=O), 1599, 1509, 1488, 1437, 1421, 1409, 1394, 1305, 1250, 1215, 1183, 1155, 1084, 1022, 950, 906, 896, 845, 829, 818, 793, 764, 737, 720, 690, 659; ^{1}H NMR (400 MHz, $CDCl_3$) δ 7.84–7.80 (m, 2 H, 2 × Ar-CH), 7.72 (d, *J* = 2.2 Hz, 1 H, Ar-CH), 7.65–7.63 (dd, *J* = 8.5, 2.2 Hz, 1 H, Ar-CH), 7.36 (d, *J* = 8.5 Hz, 1 H, Ar-CH), 7.01–6.97 (m, 2 H, 2 × Ar-CH), 3.91 (s, 3 H, OCH_3), 2.46 (s, 3 H, Ar-CH_3); ^{13}C NMR (101 MHz, $CDCl_3$) δ 193.8 (C=O), 163.6 (Ar-C_q-OMe), 150.5 (Ar-C_q-OTf), 138.2 ($Ar_{(OTf)}$-C_q-CO), 133.4 (Ar-CH), 132.5 (2 × Ar-CH), 131.2 (Ar-C_q-CH_3), 129.4 ($Ar_{(OMe)}$-C_q-CO), 129.1 (Ar-CH), 121.1 (Ar-CH), 118.6 (q, J_{C-F} = 320.1 Hz, CF_3), 113.8 (2 × Ar-CH), 55.5 (OCH_3), 16.4 (Ar-CH_3); ^{19}F{^{1}H}

NMR (377 MHz, $CDCl_3$) δ –73.7; HRMS (ESI+) m/z calcd for $C_{16}H_{14}F_3O_5S$ [M+H]: 375.0514, Found: 375.0509.

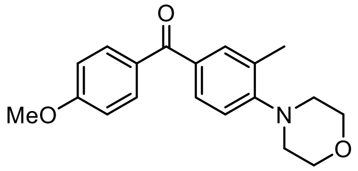

(4-Methoxyphenyl)(3-methyl-4-morpholinophenyl)methanone (8b)

Using conditions developed by Buchwald,[6] triflate **20b** (187 mg, 0.50 mmol), $Pd(OAc)_2$ (5.6 mg, 0.025 mmol), BINAP (23.4 mg, 0.037 mmol) and Cs_2CO_3 (228 mg, 0.70 mmol) were added to a reaction vial. The reaction vial was evacuated and then refilled with nitrogen three times. Morpholine (87.5 µL, 1.00 mmol) followed by toluene (1.0 mL) were added via syringe into the vial. The reaction mixture was stirred at 100 °C for 24 h then cooled to rt. Et_2O(15 mL) was added and the reaction mixture was filtered through celite® and then concentrated under reduced pressure. Purification by flash column chromatography (10% to 20% EtOAc/pentane) afforded morpholine ketone **8b** (136 mg, 87%) as a yellow oil. R_f = 0.22 (30% EtOAc/hexane); IR (film)/cm^{-1} 2958, 2851, 1645 (C=O), 1597, 1574, 1445, 1417, 1372, 1305, 1273, 1249, 1227, 1206, 1168, 1147, 1113, 1069, 1046, 1029, 971, 921, 864, 846, 829, 770, 698; ^{1}H NMR (400 MHz, $CDCl_3$) δ 7.83–7.79 (m, 2 H, 2 × Ar-CH), 7.66 (d, *J* = 2.2 Hz, 1 H, Ar-CH), 7.62–7.59 (dd, *J* = 8.2, 2.2 Hz, 1 H, Ar-CH), 7.03 (d, *J* = 8.2 Hz, 1 H, Ar-CH), 6.98–6.95 (m, 2 H, 2 × Ar-CH), 3.90–3.87 (m, 7 H, OCH_3, C*H$_2$*OC*H$_2$*), 3.02–3.00 (m, 4 H, C*H$_2$*NC*H$_2$*), 2.36 (s, 3 H, Ar-CH_3); ^{13}C NMR (101 MHz, $CDCl_3$) δ 194.9 (C=O), 162.8 (Ar-C_q-OCH_3), 154.8 (Ar-C_q-N), 133.1 (Ar-CH), 132.6 ($Ar_{(N)}$-C_q-CO), 132.3 (2 × Ar-CH), 131.7 (Ar-C_q-CH_3), 130.7 ($Ar_{(OMe)}$-C_q-CO), 129.2 (Ar-CH), 117.8 (Ar-CH), 113.4 (2 × Ar-CH), 67.2 (*C*H_2O*C*H_2), 55.4 (OCH_3), 51.7 (*C*H_2N*C*H_2), 18.4 (Ar-CH_3); HRMS (+ESI) m/z calcd for $C_{19}H_{22}NO_3$ [M+H]: 312.1600, Found: 312.1603.

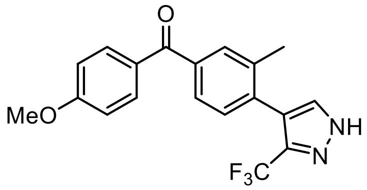

(4-Methoxyphenyl)(3-methyl-4-(3-(trifluoromethyl)-1*H*-pyrazol-4-yl)phenyl)methanone (9b)

Ketone **20b** (111 mg, 0.30 mmol), pyrazole pinacol ester (87.0 mg, 0.33 mmol), Pd(dppf)Cl_2 (11.0 mg, 0.015 mmol) and K_3PO_4 (127 mg, 0.60 mmol) were added to a reaction vial. The reaction vial was evacuated and then refilled with nitrogen three times. Dioxane/water (4:1, 3.0 mL) was added via syringe into the vial. The reaction mixture was stirred at 130 °C for 14 h then cooled to rt. EtOAc (15 mL) was added and the crude mixture was filtered through celite® and then concentrated under reduced pressure. Purification by flash column chromatography (30% EtOAc/hexane) afforded ketone **9b** (51 mg, 47%) as a pale-yellow solid. R_f = 0.13 (30% EtOAc/hexane); mp = 207–208 °C; IR (film)/cm^{-1} 3193 (br, NH), 2924, 1650 (C=O), 1599, 1510, 1475, 1307, 1258, 1172, 1099, 1029, 966, 840, 771; ^{1}H NMR (400 MHz, $(CD_3)_2CO$) δ 13.02 (s, 1 H, NH), 8.02 (d, *J* = 0.9 Hz, 1 H, N-Ar-CH), 7.85–7.82 (m, 2 H, 2 × Ar-CH), 7.67 (m, 1 H, Ar-CH), 7.60–7.57 (dd, *J* = 7.9, 1.4 Hz, 1 H, Ar-CH), 7.38 (d, *J* = 7.9 Hz, 1 H, Ar-CH), 7.11–7.07 (m, 2 H, 2 × Ar-CH), 3.92 (s, 3 H, OCH_3), 2.27 (s, 3 H, Ar-CH_3); ^{13}C NMR (101 MHz, $(CD_3)_2CO$) δ 194.9 (C=O), 164.2 (Ar-C_q-OMe), 138.9 (Ar-C_q-$C_{q(pyrazole)}$), 138.3 (Ar-C_q-CO), 135.5 (Ar-C_q-CO), 133.0 (2 × Ar-CH), 131.6 (Ar-CH), 131.5 (Ar-CH), 131.1 (N-Ar-CH), 130.9 (Ar-C_q-CH_3), 128.8 (C_q-CF_3), 127.4 (Ar-CH), 123.1 (q, $J_{C\text{-}F}$ = 272.1 Hz, CF_3), 119.5 (N-Ar-CH-C_q), 114.5 (2 × Ar-CH), 55.9 (OCH_3), 20.4 (Ar-CH_3); ^{19}F{^{1}H} NMR (377 MHz, $CDCl_3$) δ –60.3; HRMS (ESI-) m/z calcd for $C_{19}H_{14}N_2O_2F_3$ [M-H]: 359.1007, Found: 359.1012.

Reactivity of 3-indolylmethanols 11c and 11e

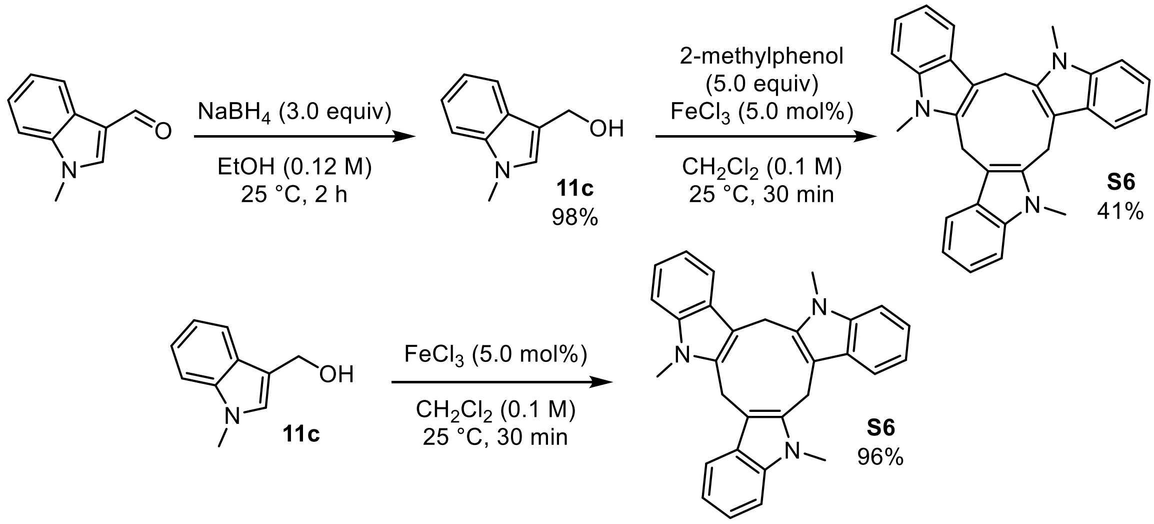

Figure F9.

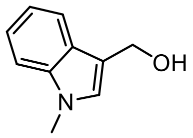

(1-Methyl-1*H*-indol-3-yl)methanol (11c)

$NaBH_4$ (214 mg, 5.65 mmol) was added portionwise to a solution of 1-methyl-1*H*-3-carbaldehyde (300 mg, 1.88 mmol) in dry ethanol (15.7 mL) at 25 °C. The reaction mixture was stirred for 2 h then the solvent was removed under reduced pressure followed by the addition of water (30 mL). The aqueous layer was extracted with Et_2O (3 × 30 mL). The organic extracts were combined, dried over Na_2SO_4, filtered and concentrated under reduced pressure to afford alcohol **11c** (297 mg, 98%) as a yellow oil. R_f = 0.30 (100% Et_2O); IR (film)/cm^{-1} 3317 (br, OH), 2931, 2874, 1474, 1328, 1127, 1064, 1025, 982, 781, 735, 675; ^{1}H NMR (400 MHz, $CDCl_3$) δ 7.77–7.68 (m, 1 H, $Ar_{(indole)}$-CH), 7.34 (d, *J* = 8.2 Hz, 1 H, $Ar_{(indole)}$-CH), 7.30–7.25 (m, 1 H, $Ar_{(indole)}$-CH), 7.20–7.15 (m, 1 H, $Ar_{(indole)}$-CH), 7.09 (s, 1 H, N-Ar-CH), 4.88 (s, 2 H, CH_2), 3.79 (s, 3 H, NCH_3), 1.40, (br, s, 1 H, OH)*; HRMS (+EI) *m/z* calcd for $C_{10}H_{11}NO^{\bullet+}$ $[M]^{\bullet+}$: 161.0841, Found: 161.0835. Compound previously reported without characterisation data.[8] * *^{13}C NMR could not be obtained due to the instability of the product.*

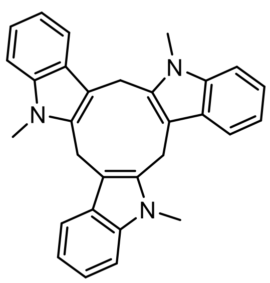

1,4,7-Trihydrocyclononano[2,3-b:5,6-b:8,9-b]tri-1-methylindole (S6)

$FeCl_3$ (4.90 mg, 0.03 mmol) was added to a solution of **11e** (98.0 mg, 0.61 mmol) in CH_2Cl_2 (1.20 mL) at 25 °C. The reaction mixture was stirred for 1 h then sat. aq $NaHCO_3$ (15 mL) was added followed by CH_2Cl_2 (15 mL). The layers were separated and the aqueous portion was extracted with CH_2Cl_2 (2 × 15 mL). The organic extracts were combined, dried over Na_2SO_4, filtered and concentrated under reduced pressure to afford trimer **S6** (83.5 mg, 96%) as a beige powder. R_f = 0.69 (50% Et_2O/pentane); mp = 275–282 °C [lit. = 278–281 °C][10], coloration into a black solid from 235 °C; IR (film)/cm^{-1} 2925, 1469, 1434, 1363, 1176, 1130, 1013, 731; ^{1}H NMR (400 MHz, $CDCl_3$) δ 7.55 (d, *J* = 7.8 Hz, 3 H, 3 × $Ar_{(indole)}$-CH), 7.29 (d, *J* = 8.1 Hz, 3 H, 3 × $Ar_{(indole)}$-CH), 7.21–7.14 (m, 3 H, 3 × $Ar_{(indole)}$-CH), 7.13–7.05 (m, 3 H, 3 × $Ar_{(indole)}$-CH), 4.07 (s, 6 H, 3 × CH_2), 3.76 (s, 9 H, 3 × CH_3); ^{13}C NMR (101 MHz, $CDCl_3$) δ 136.3 (3 × $Ar_{(indole)}$-C_q), 136.0 (3 × $Ar_{(indole)}$-C_q), 127.5 (3 × N-Ar-C_q), 120.8 (3 × $Ar_{(indole)}$-CH), 118.9 (3 × $Ar_{(indole)}$-CH), 117.2 (3 × $Ar_{(indole)}$-CH), 108.9 (3 × $Ar_{(indole)}$-CH), 107.0 (3 × N-Ar-C_q-*C_q*), 29.7 (3 × NCH_3), 20.4 (3 × CH_2); HRMS (+APCI) *m/z* calcd for $C_{30}H_{28}N_3^+$ $[M+H]^+$: 430.2278, Found: 430.2278. The observed spectroscopic and physical data (^{1}H, ^{13}C, mp) for this compound was consistent with that previously reported.[9]

1-(1-Methyl-1*H*-indol-3-yl)cyclobutan-1-ol (11e) and **3,3'-(cyclobutane-1,1-diyl)bis(1-methyl-1*H*-indole) (S7)**

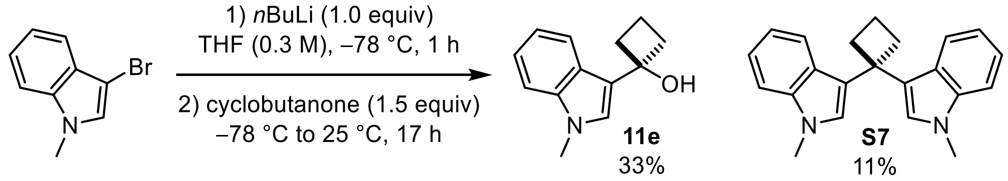

*n*BuLi (2.30 M in hexane, 2.60 mL, 5.93 mmol) was added dropwise over 5 min to a solution of 3-bromo-1-methyl-1*H*-indole[10] (1.34 g, 5.93 mmol) in THF (21 mL) at –78 °C. The reaction mixture was stirred at –78 °C for 1 h. Cyclobutanone (665 µL, 8.90 mmol) was added dropwise to the reaction mixture. Following a further 2 h at –78 °C, the reaction mixture was warmed to 25 °C for 17 h then quenched with water (80 mL). EtOAc (80 mL) was added and the layers were separated. The aqueous portion was extracted with EtOAc (2 × 80 mL). The organic extracts were combined, dried over Na_2SO_4, filtered and concentrated under reduced pressure. Purification by flash column chromatography (5 to 10% EtOAc/pentane) afforded cyclobutane **S7** (205 g, 11%) as a white solid, followed by cyclobutanol **11e** (396 mg, ~60% pure, 33%) as a purple oil.

Cyclobutane **S7**: R_f = 0.52 (10% EtOAc/pentane); mp = 145–149 °C (coloration at 99 °C into a pink then black solid); IR (film)/cm^{-1} 3325 (br, OH), 3046, 2975, 2936 1483, 1462 1378, 1324, 1234, 1156, 1012, 737, 696; ^1H NMR (400 MHz, $CDCl_3$) δ 7.67 (d, *J* = 8.0 Hz, 2 H, 2 × $Ar_{(indole)}$-CH), 7.32 (d, *J* = 8.2 Hz, 2 H, 2 × $Ar_{(indole)}$-CH), 7.22 (m, 2 H, 2 × $Ar_{(indole)}$-CH), 7.05 (m, 2 H, 2 × $Ar_{(indole)}$-CH), 6.97 (s, 2 H, 2 × N-Ar-CH), 3.77 (s, 6 H, 2 × NCH_3), 2.96 (t, *J* = 7.7 Hz, 4 H, $CH_2CH_2CH_2$), 2.22 (t, *J* = 7.7 Hz, 2 H, $CH_2CH_2CH_2$); ^{13}C NMR (101 MHz, $CDCl_3$) δ 137.8 (2 × $Ar_{(indole)}$-C_q), 126.7 (2 × $Ar_{(indole)}$-C_q), 125.9 (2 × N-Ar-CH), 122.6 (2 × N-Ar-CH-C_q), 121.1 (2 × $Ar_{(indole)}$-CH), 120.8 (2 × $Ar_{(indole)}$-CH), 118.1 (2 × $Ar_{(indole)}$-CH), 109.2 (2 × $Ar_{(indole)}$-CH), 40.8 ($C_{q(cyclobutane)}$), 34.6 ($CH_2CH_2CH_2$), 32.6 (2 × NCH_3), 17.2 ($CH_2CH_2CH_2$); HRMS (-ESI) *m/z* calcd for $C_{22}H_{21}N_2^{\bullet+}$ $[M-H]^{\bullet+}$: 313.1705, Found: 313.1708.

Cyclobutanol **11e**: R_f = 0.23 (10% EtOAc/pentane); IR (film)/cm^{-1} 3309 (br, OH), 2933, 1474, 1460, 1416, 1326, 1245, 1217, 1145, 1119, 1095, 953, 820, 737; ^1H NMR (400 MHz, $CDCl_3$) δ 7.83–7.76 (m, 1 H, $Ar_{(indole)}$-CH), 7.34 (dt, *J* = 8.3, 1.1 Hz, 1 H, $Ar_{(indole)}$-CH), 7.31–7.25 (m, 1 H, $Ar_{(indole)}$-CH), 7.15 (ddd, *J* = 8.0, 6.9, 1.1 Hz, 1 H, $Ar_{(indole)}$-CH), 7.07 (s, 1 H, N-Ar-CH), 3.80 (s, 3 H, NCH_3), 2.68–2.58 (m, 2 H, $CHHCH_2CHH$), 2.55–2.45 (m, 2 H, $CHHCH_2CHH$), 1.96–1.84 (m, 1 H, CH_2CHHCH_2), 1.72–1.57 (m, 1 H, CH_2CHHCH_2); ^{13}C NMR (101 MHz, $CDCl_3$) δ 137.9 ($Ar_{(indole)}$-C_q), 125.7 (N-Ar-CH), 124.4 ($Ar_{(indole)}$-C_q), 121.9 ($Ar_{(indole)}$-CH), 120.6 ($Ar_{(indole)}$-CH), 119.8 (N-Ar-CH-C_q), 119.2 ($Ar_{(indole)}$-CH), 109.4 ($Ar_{(indole)}$-CH), 73.1 ($C_{q(cyclobutane)}$), 36.8 ($CH_2CH_2CH_2$), 32.7 (NCH_3), 13.3 ($CH_2CH_2CH_2$). Compound unstable, HRMS not obtained.

ADMET

elogD: An automated reversed phase-HPLC method developed by Lombardo *et al*. was used for logD (pH 7.4) measurements. All the chromatographic runs were performed on a HP-1100 HPLC ChemStation at the ambient temperature. The HPLC columns used were Supelcosil LC-ABZ, 5 µm, 4.6 mm × 50 mm. A diode array detector was used to monitor signals at 210, 225, 245, and 310 nm. The mobile phase consisted of 15−75% of *A* and 85−25% of *B*.
Mobile phase A: methanol with addition of 0.25% 1-octanol.
Mobile phase B: 20 mM of MOPS (morpholine−propane−sulfonic acid) buffer prepared in octanol saturated water and 0.25% of n-decylamine.
Samples were prepared in 1:1 mixture of methanol/water at 100 µg/mL concentration.
The retention of each compound is obtained at three different concentrations of methanol in the mobile phase and extrapolated to 0% methanol (100% water).

HLM Assay: The high throughput human microsomal stability assay was performed in a 384-well format. All liquid handling and incubation were conducted with a Biomek FX, equipped with one 3 position Mecour heat exchangers. Pooled human liver microsomes of 50 donors (Lot: HLM-103) were purchased from BioIVT. Each incubation contained test compound (1 µM), human liver microsomes (0.25 mM CYP protein equivalent to 0.806 mg/mL protein concentration), NADPH (1.3 mM), $MgCl_2$ (3.3 mM) and potassium phosphate buffer (100 mM at pH 7.4). The final reaction volume was 45 mL containing 0.01% DMSO. The incubations were conducted at 37 °C at various time points (1, 4, 7, 12, 20, 25, 45, 60 min). Cold acetonitrile with mass spectrometry (MS) internal standard (IS, CP-628374) was then added to quench the reaction. The plates were centrifuged at 3000 rpm for 10 min at 4 ºC (Sorvall RC 3C Plus, Thermo Scientific, Waltham, MA). The water layer was transferred to the 384-well plate, sealed and subsequently analysed using LCeMS/MS. Control plates were prepared in the same manner without adding the NADPH cofactor to monitor any non-CYP catalysed decline. Propranolol (2D6, 1A2 and 2C19 substrate), quinidine (3A4), diclofenac (2C9), verapamil (3A4, 1A2, 2C), amitriptyline (2D6, 2C19) and naloxone (UGT2B7) were used as positive controls on each plate to monitor the activities of the various CYPs in the assay.

Kinetic solubility: Samples and controls were loaded onto an appropriate assay ready plated and centrifuged at 3000 rpm for 1 min. The samples were then diluted to 600 µM with the appropriate buffers (pH 3, 7.4, 10), shaken and incubated at 37 °C for 24 h. After this time the plates were again shaken, then centrifuged at 3000 rpm for 2 min. The supernatant was filtered through a 22 micron filter, diluted in MeCN and analysed by LCMS in presence of an internal standard.
Assay ready plated: Plate containing 6µL at 30 mM of test compounds in 96 deep well plate (volume = 600 µL). Or: containing 50 nL at 30 mM of test compound in 96 well plate (volume = 500 µL).
Aqueous buffer preparation: pH 3: 50 µL of 1N hydrochloric acid was added to 500 mL of deionized water. The solution was titrated with 1N hydrochloric acid to pH 3, and back titrated with 1N sodium hydroxide. pH 7.4: 100 mM sodium phosphate buffer is made by adding 56.4 g of dibasic sodium phosphate and 10.3 g of monobasic sodium phosphate to 4 L of deionized water. 250 µL of 100 mM sodium phosphate buffer was diluted with 250 µL of deionized water. The solution was titrated with 1N hydrochloric acid to pH 7.4, and back titrated with 1N sodium hydroxide. pH 10: Fisher Scientific pH 10 buffer (SB116-500) containing potassium hydroxide, potassium carbonate, and potassium borate.

Chemical Stability Assay was performed in 384 well plate format (volume = 450 µL). Plates were prefilled with 70 µL of the appropriate buffer (pH 1.2, 7.4, 10) after which the wells were spiked with 5 µL of test compounds (1 mM). Samples were incubated for their timepoint of interest (1 h, 4 h, 24 h) at 37 °C after which the samples were diluted in MeCN (and pH 12.8 buffer for experiment at pH 1.2), mixed with an internal standard and analysed by LCMS.

Aqueous buffer preparation: pH 3: 50 µL of 1N hydrochloric acid was added to 500 mL of deionized water. The solution was titrated with 1N hydrochloric acid to pH 3, and back titrated with 1N sodium hydroxide. pH 7.4: 100 mM sodium phosphate buffer is made by adding 56.4 g of dibasic sodium phosphate and 10.3 g of monobasic sodium phosphate to 4 L of deionized water. 250 µL of 100 mM sodium phosphate buffer was diluted with 250 µL of deionized water. The solution was titrated with 1N hydrochloric acid to pH 7.4, and back titrated with 1N sodium hydroxide. pH 10: Fisher Scientific pH 10 buffer (SB116-500) containing potassium hydroxide, potassium carbonate, and potassium borate. pH 12.8: 30 mL of 1N NaOH was added to 200 mL of deionized water. The solution was titrated with 1N sodium hydroxide to pH 12.8, and back titrated with 1N hydrochloric acid.

RRCK: Passive permeability assay were carried out using low-efflux MDCKII cells (Madin–Darby canine kidney cell line) also called Ralph Russ Canine Kidney cell (RRCK).[11]

Table S1: cLogP, elogD and *sf*LogD of compound 1–10.

Compound	Nb	clogP	elogD	*sf*LogD
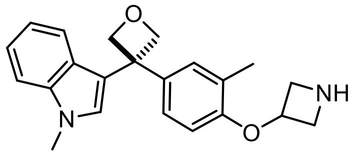	**1a**	3.42	2.13	1.37
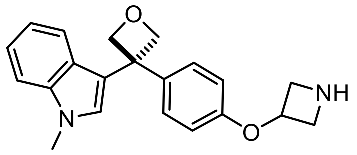	**2a**	2.92	1.70	0.99
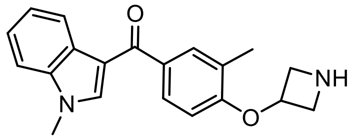	**1b**	4.03	-	1.07
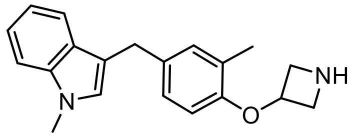	**1c**	4.67	3.10	1.68
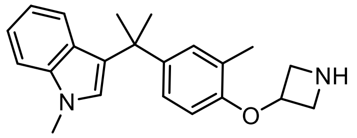	**1d**	5.47	2.90	2.59
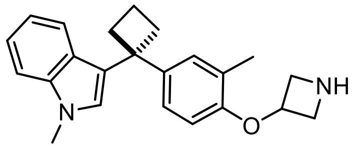	**1e**	5.66	3.20	2.81
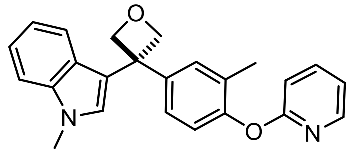	**3a**	4.51	4.60	4.36
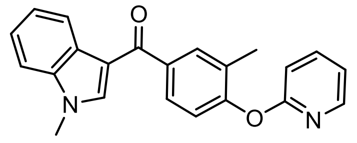	**3b**	4.92	3.99	1.92
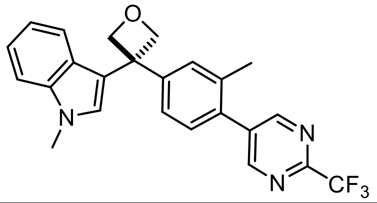	**4a**	3.99	5.1	-
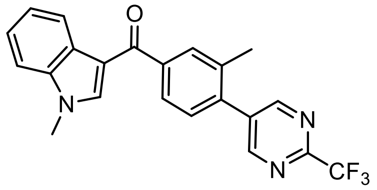	**4b**	4.43	4.30	4.21

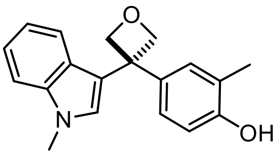	**5a**	3.19	3.73	3.63
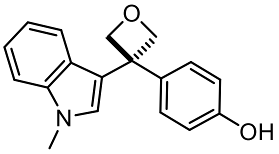	**6a**	2.74	3.28	3.27
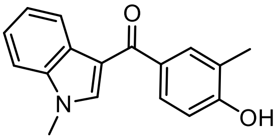	**5b**	4.07	3.30	3.33
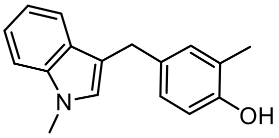	**5c**	4.45	4.78	2.79
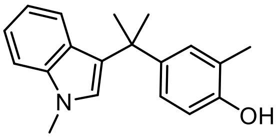	**5d**	5.25	5.05	3.72
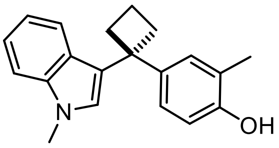	**5e**	5.44	5.40	4.40
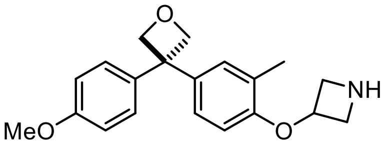	**7a**	2.88	1.43	1.06
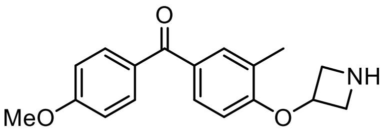	**7b**	3.50	1.74	1.21
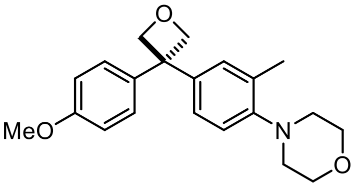	**8a**	2.83	3.68	3.47
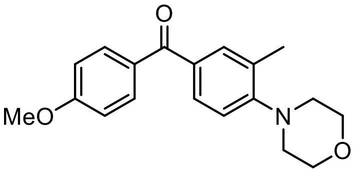	**8b**	3.49	3.90	3.06
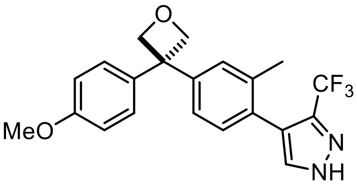	**9a**	4.33	4.50	4.62

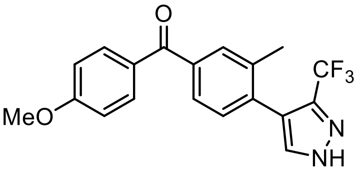	**9b**	4.89	4.80	4.74
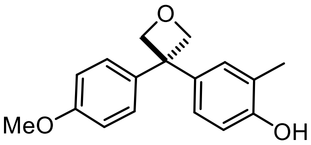	**10a**	2.65	3.07	3.22
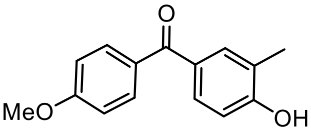	**10b**	3.54	-	3.49

^{1}H and ^{13}C NMR spectra of selected compounds

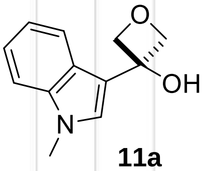

11a

^{1}H NMR (400 MHz, $CDCl_3$)

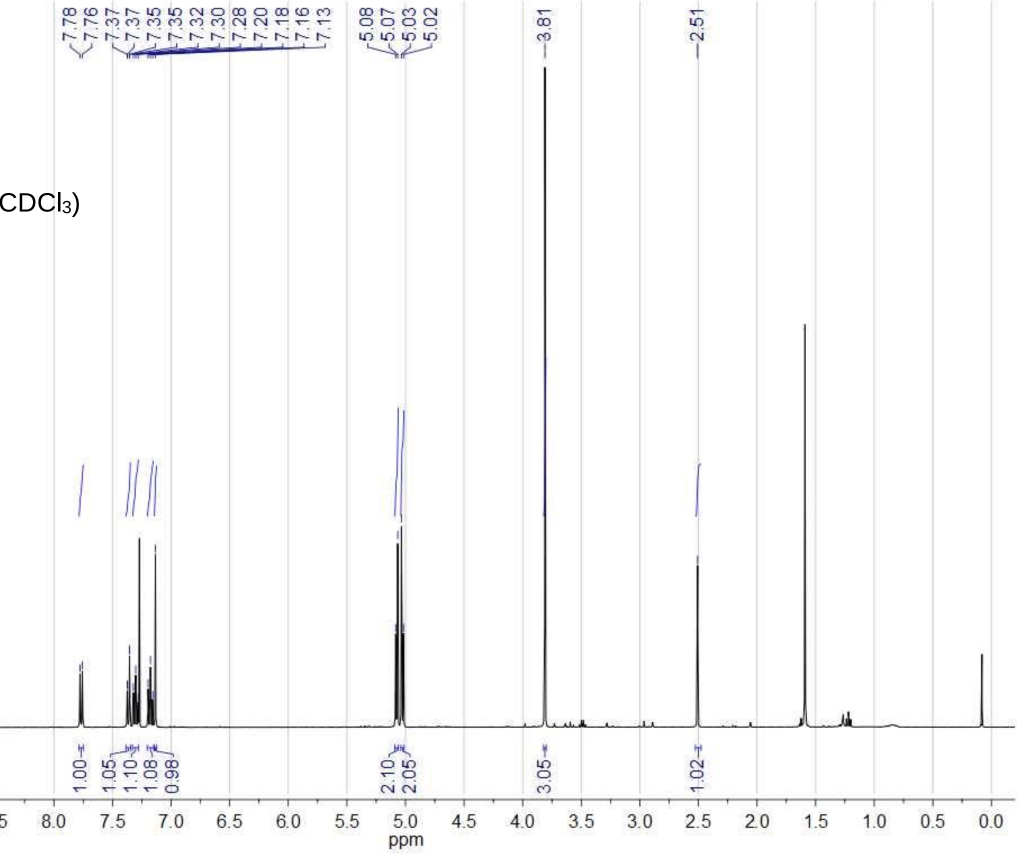

11a

^{13}C NMR (101 MHz, $CDCl_3$)

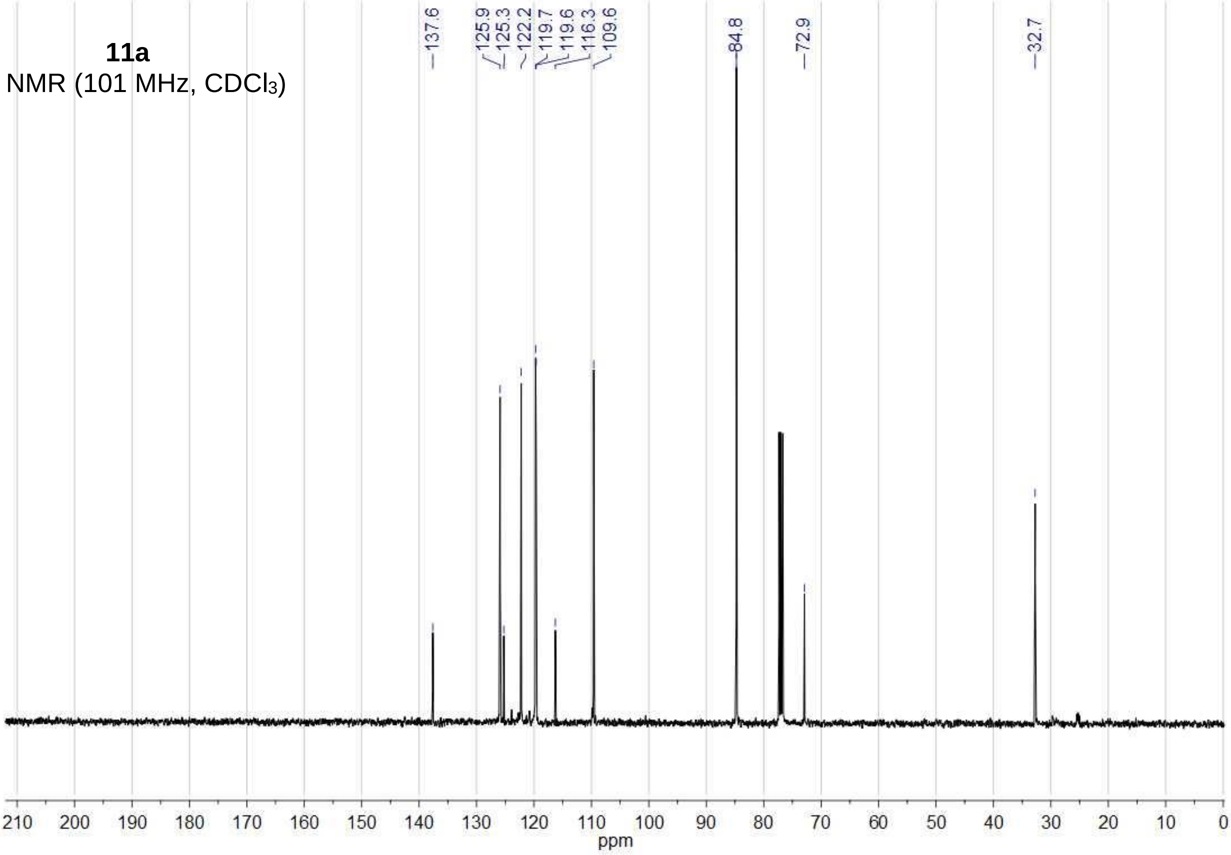

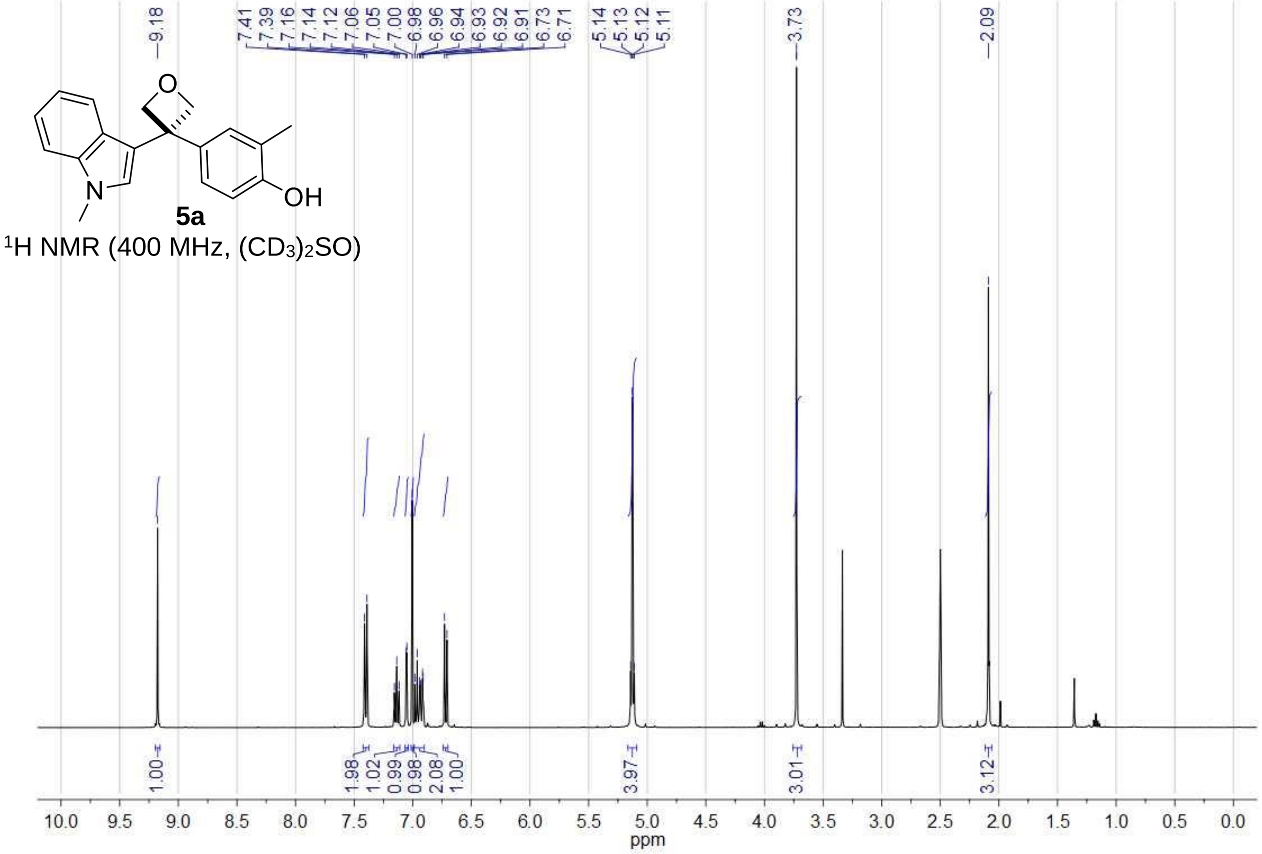

5a

^{1}H NMR (400 MHz, $(CD_3)_2SO$)

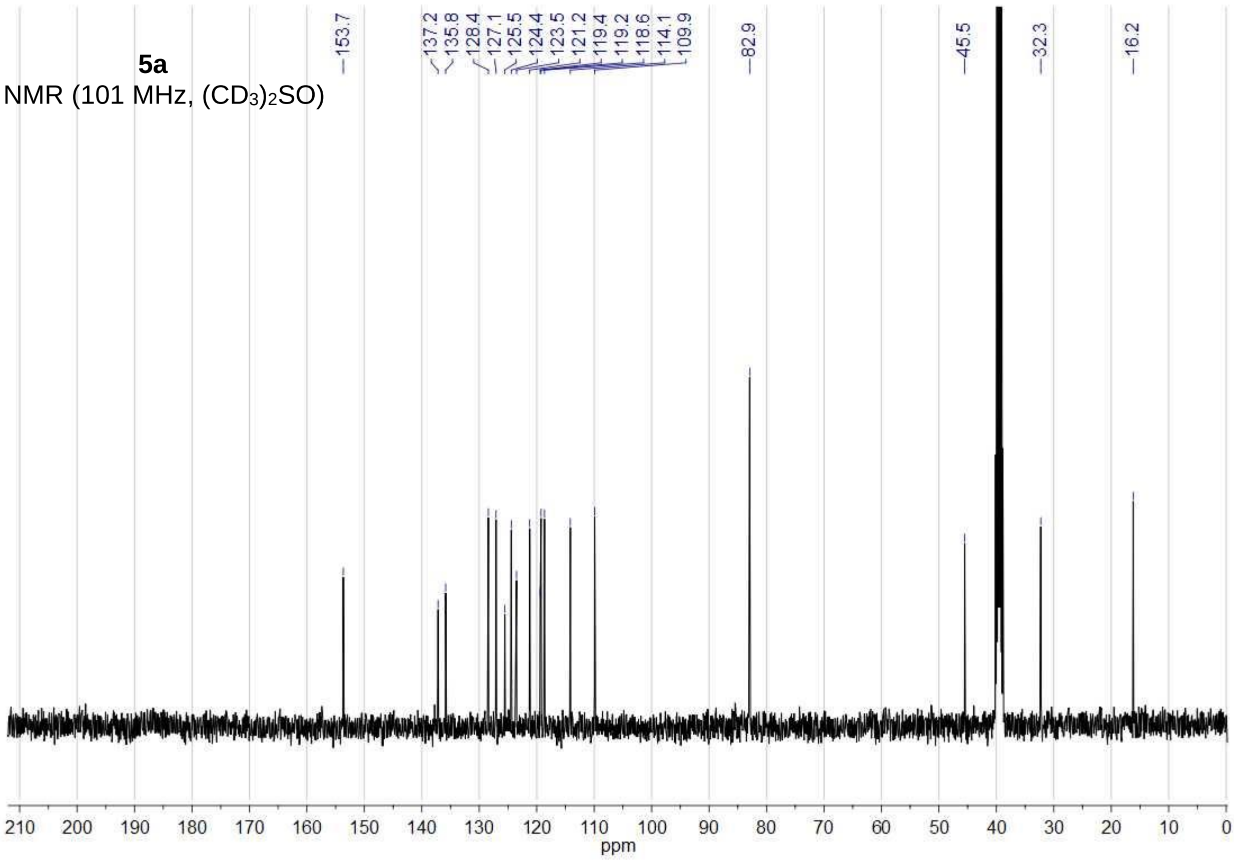

5a

^{13}C NMR (101 MHz, $(CD_3)_2SO$)

12a

^{1}H NMR (400 MHz, $CDCl_3$)

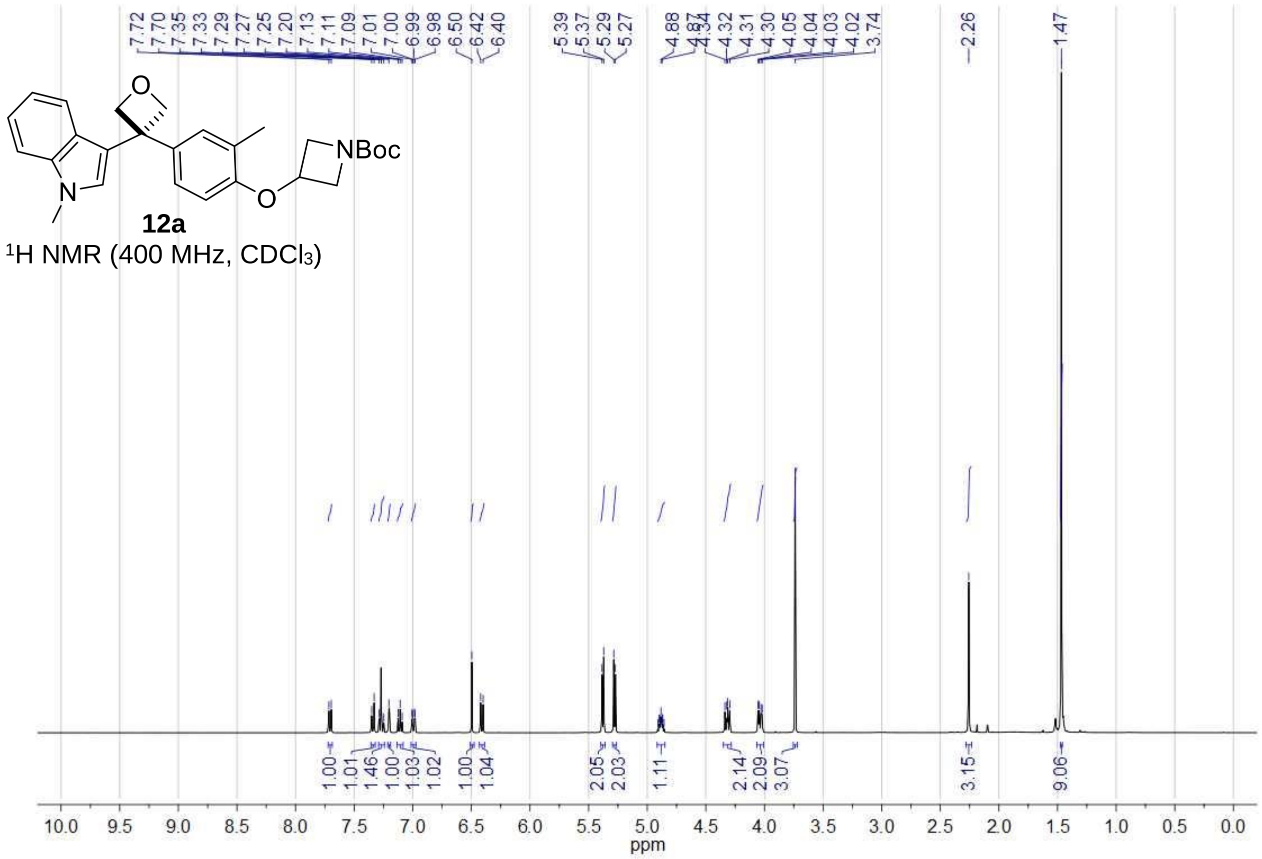

12a

^{13}C NMR (101 MHz, $CDCl_3$)

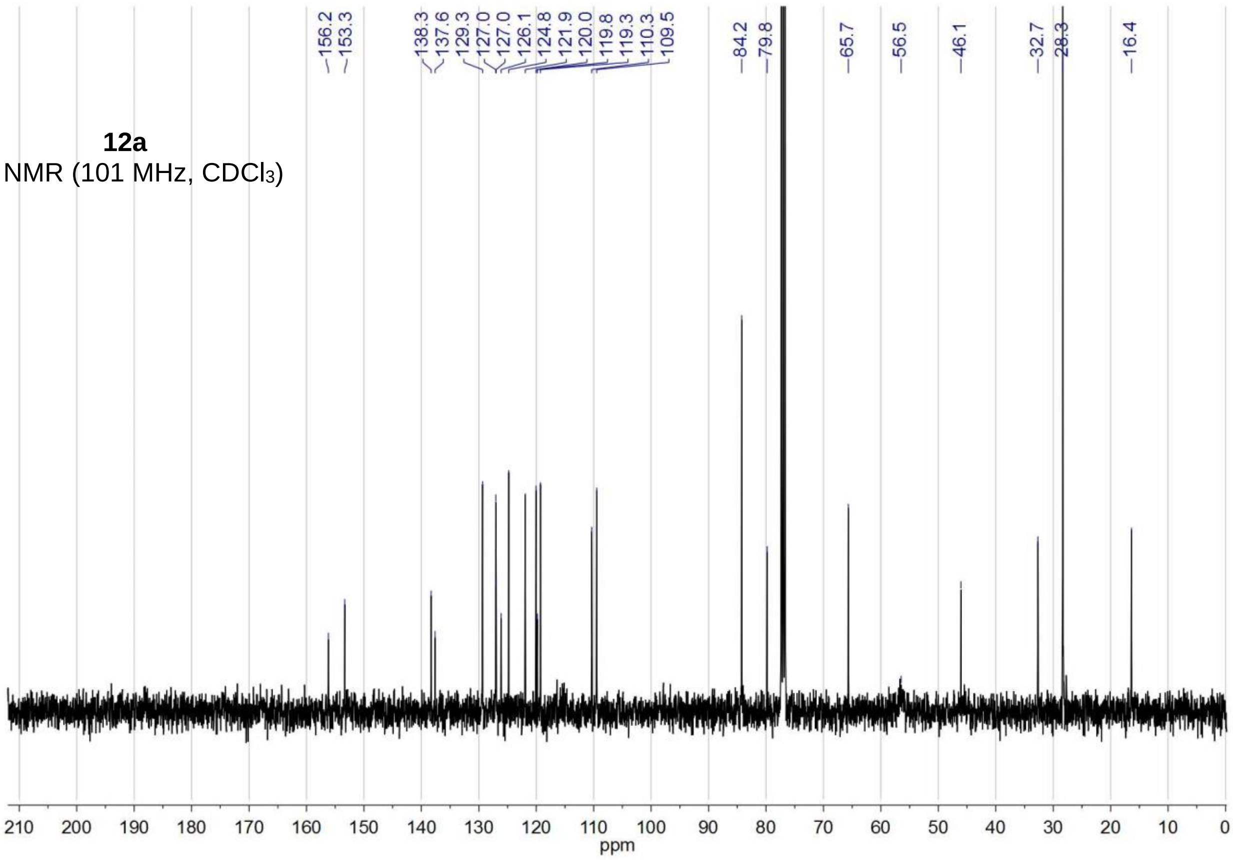

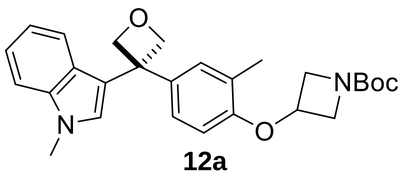

12a

[1]HSQC NMR ($CDCl_3$)

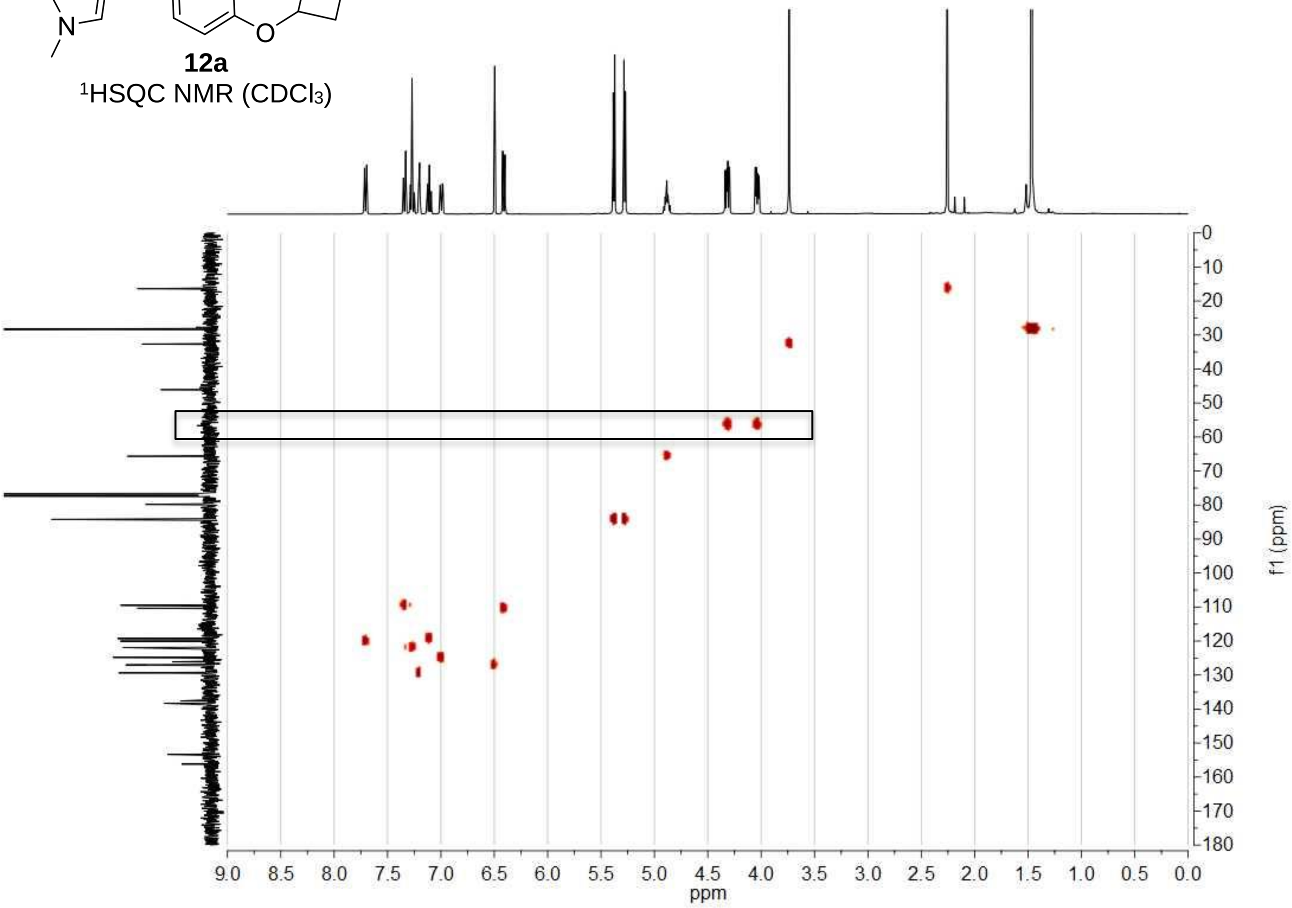

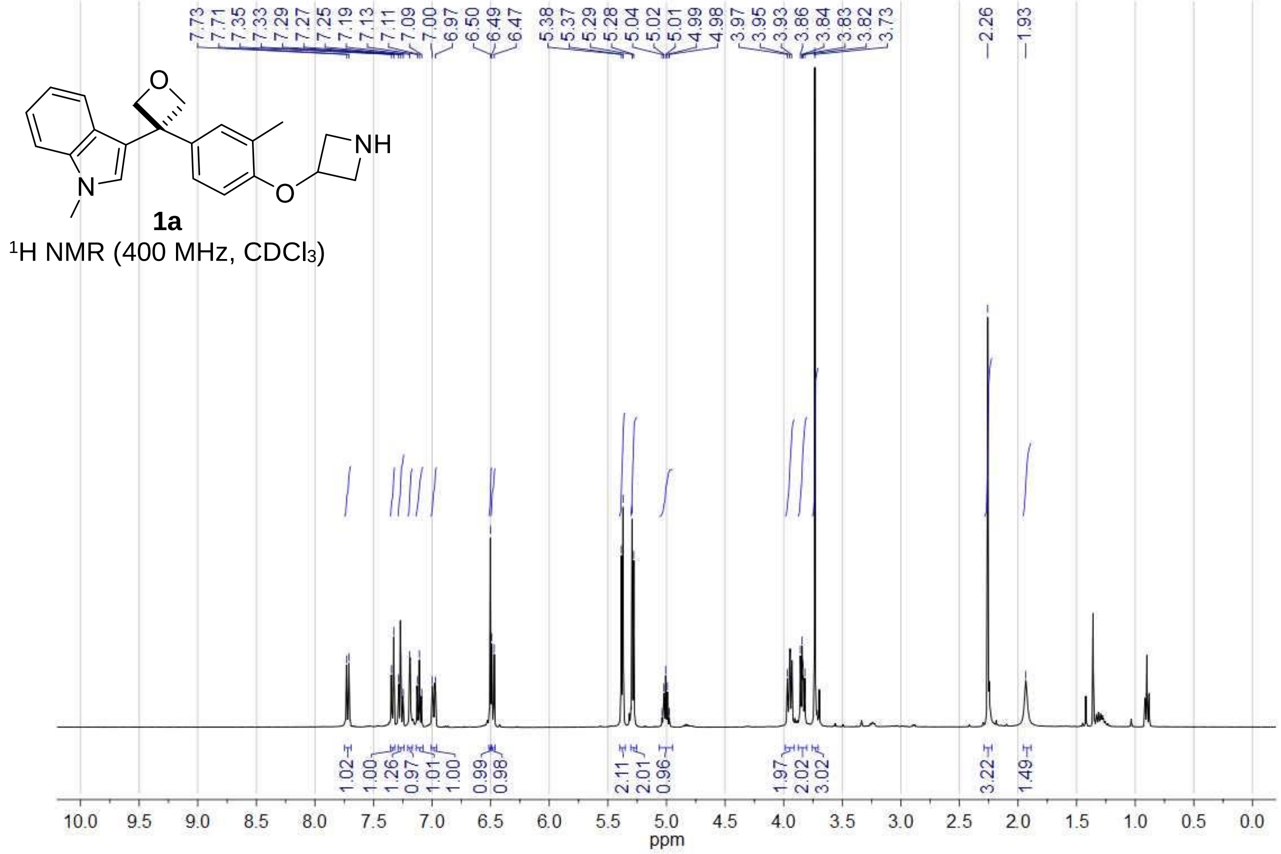
1a
1H NMR (400 MHz, CDCl3)
7.73 7.71 7.35 7.33 7.29 7.27 7.25 7.19 7.13 7.11 7.09 7.00 6.97 6.50 6.49 6.47 5.38 5.37 5.29 5.28 5.04 5.02 5.01 4.99 4.98 3.97 3.95 3.93 3.86 3.84 3.83 3.82 3.73 2.26 1.93
1.02 1.00 1.26 0.97 1.01 1.00 0.99 0.98 2.11 2.01 0.96 1.97 2.02 3.02 3.22 1.49
ppm

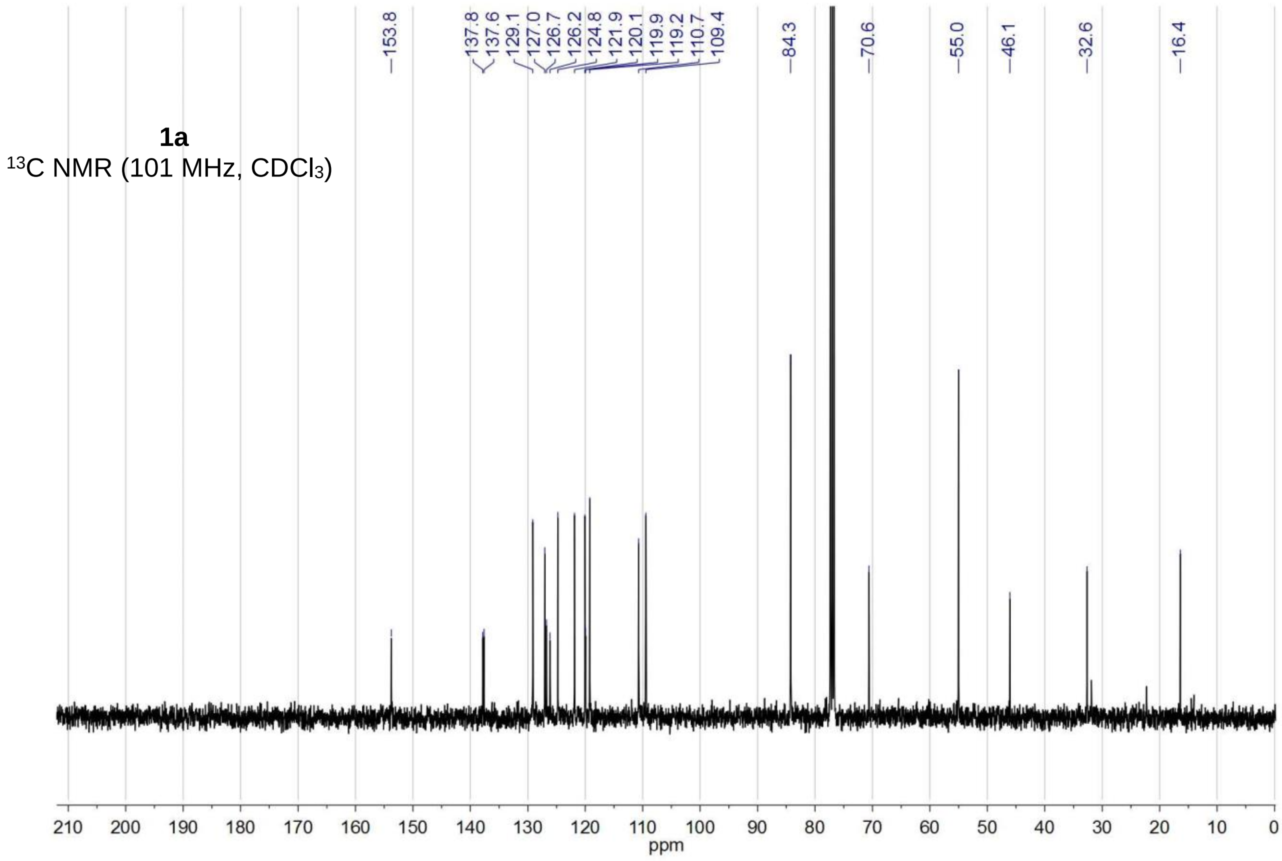
1a
13C NMR (101 MHz, CDCl3)
153.8 137.8 137.6 129.1 127.0 126.7 126.2 124.8 121.9 120.1 119.9 119.2 110.7 109.4 84.3 70.6 55.0 46.1 32.6 16.4
ppm

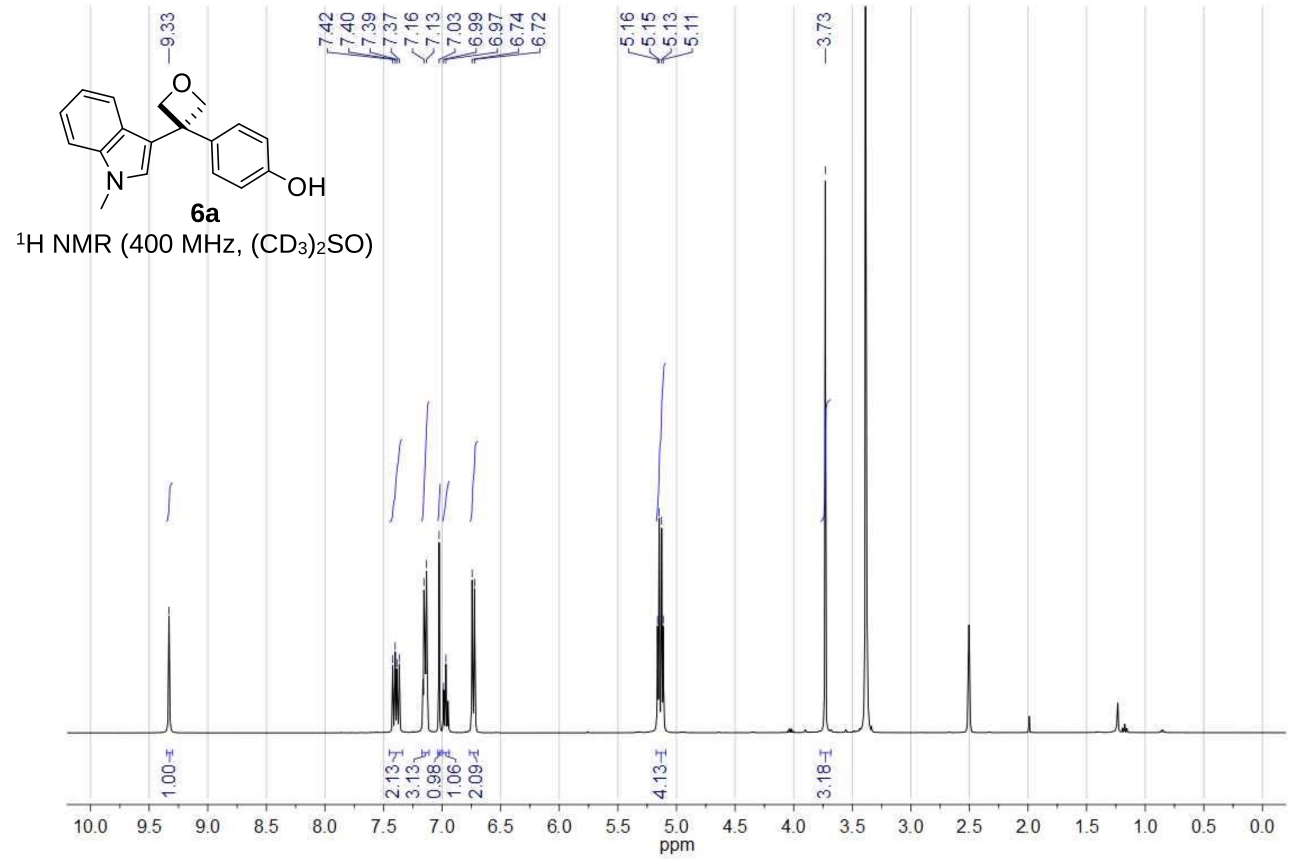

6a

^{1}H NMR (400 MHz, $(CD_3)_2SO$)

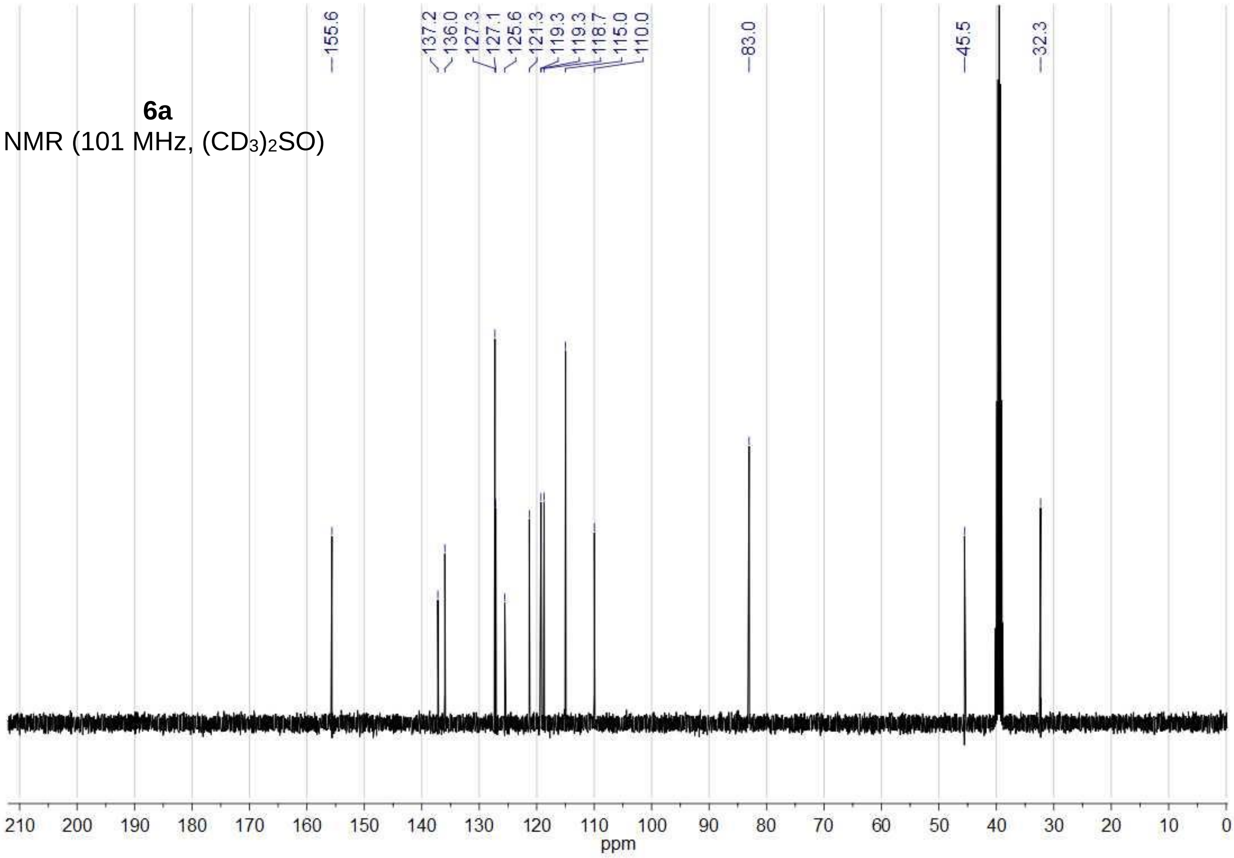

6a

^{13}C NMR (101 MHz, $(CD_3)_2SO$)

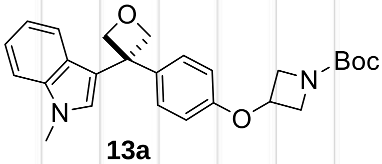

13a

^{1}H NMR (400 MHz, $CDCl_3$)

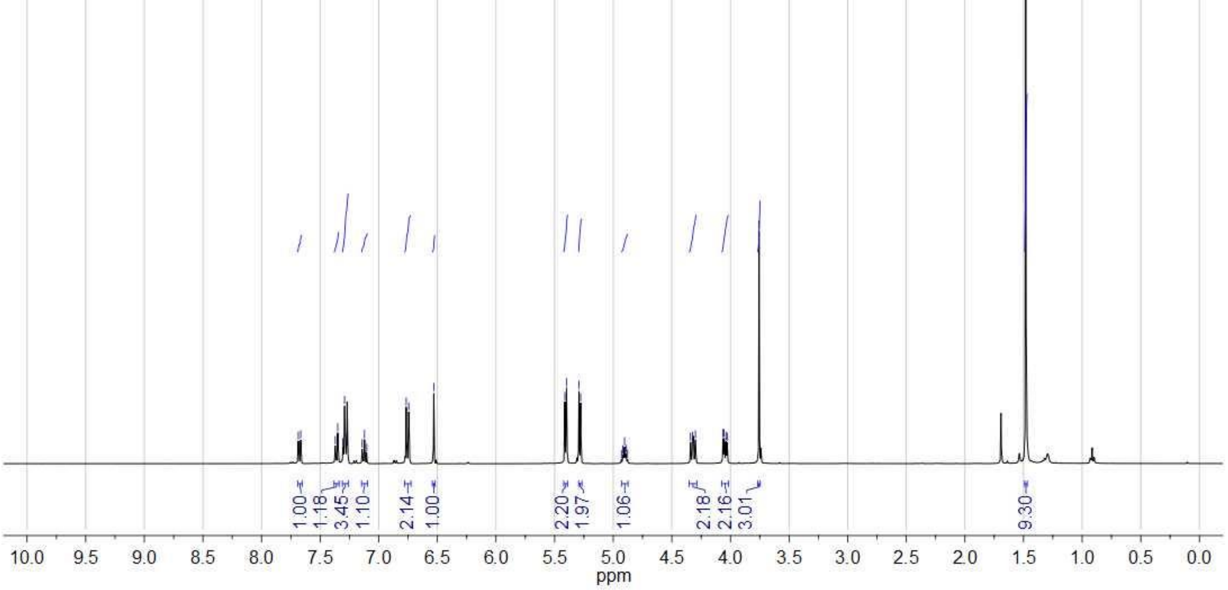

13a

^{13}C NMR (101 MHz, $CDCl_3$)

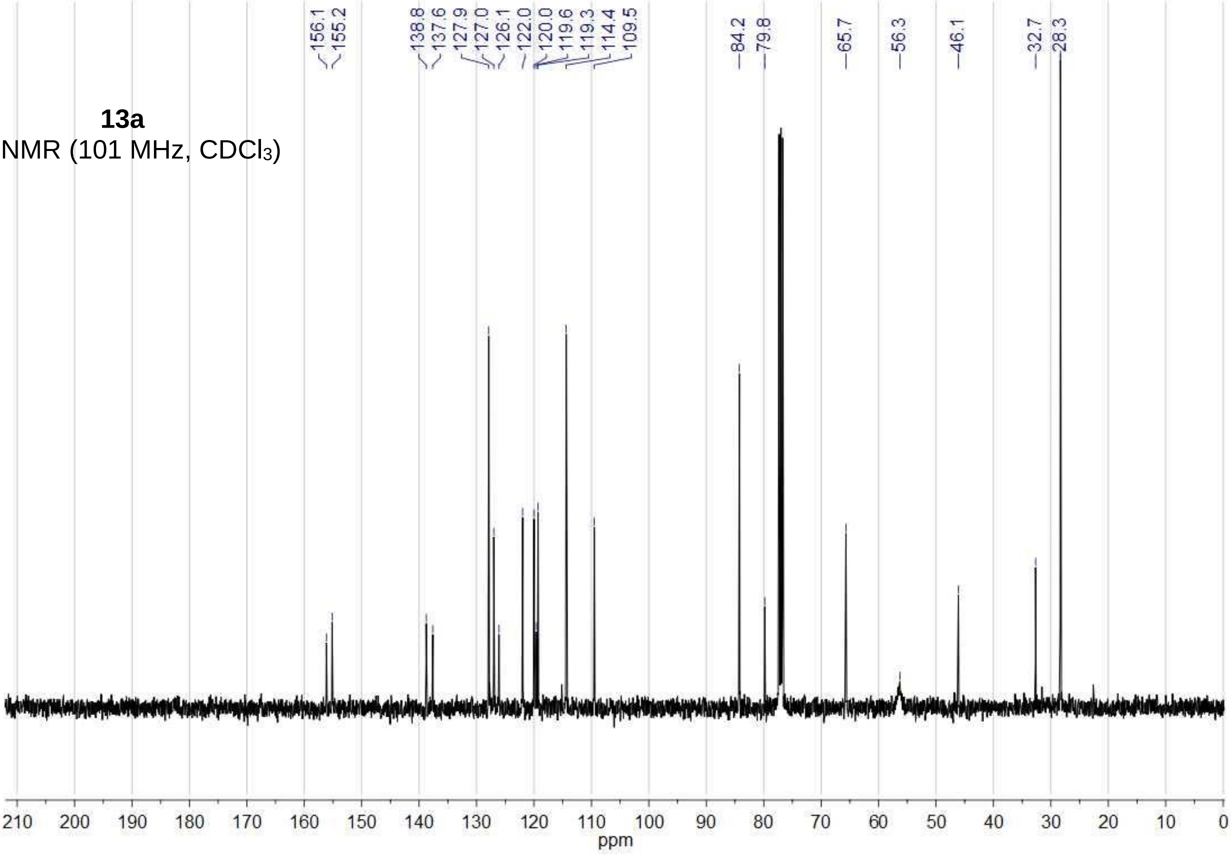

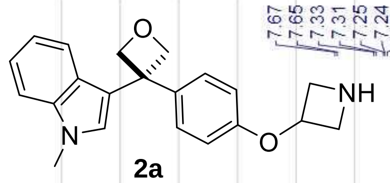

2a

^{1}H NMR (400 MHz, $CDCl_3$)

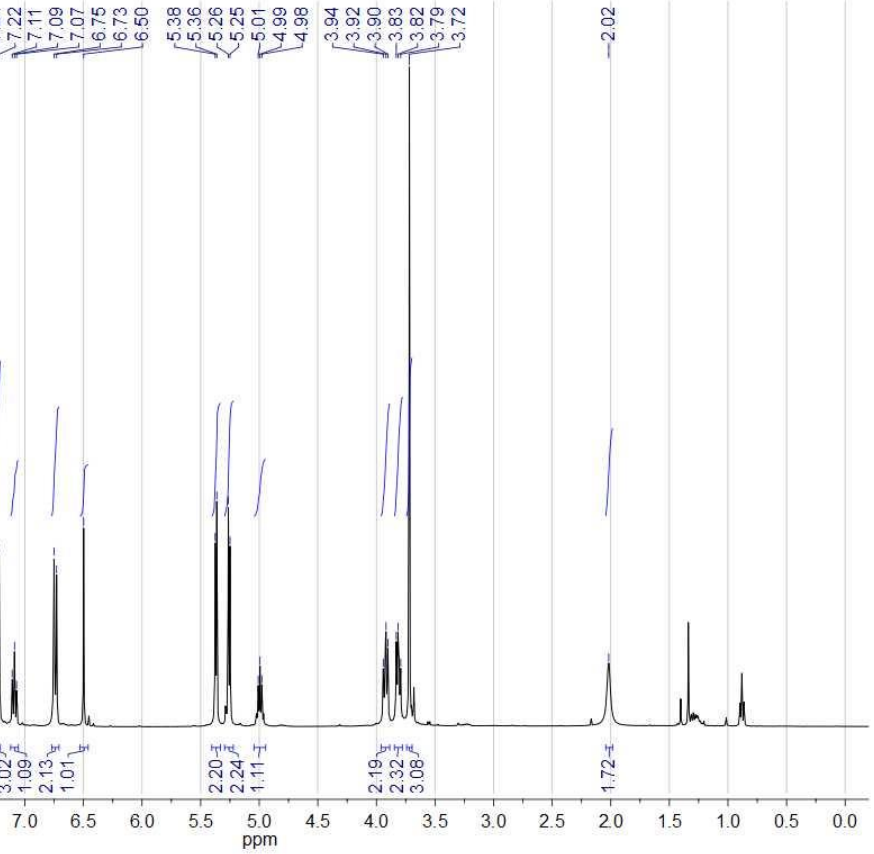

2a

^{13}C NMR (101 MHz, $CDCl_3$)

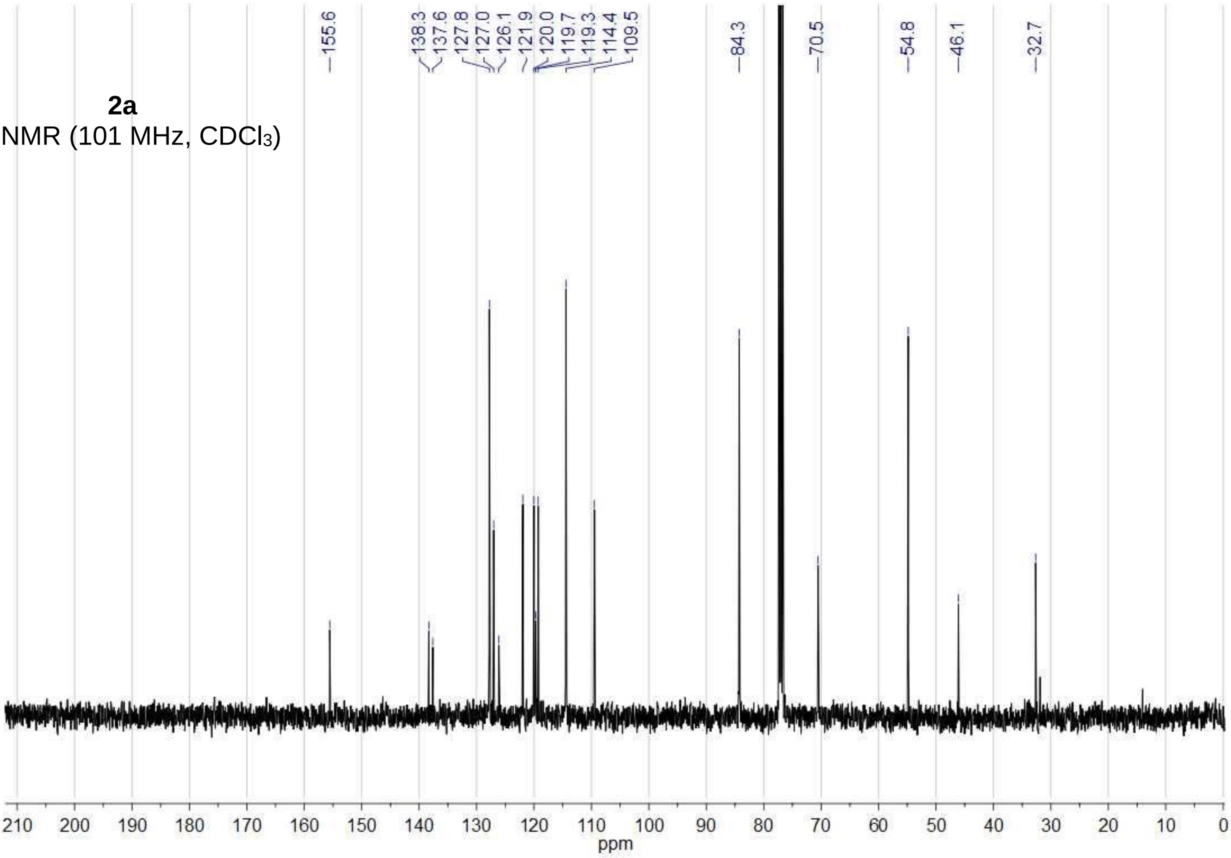

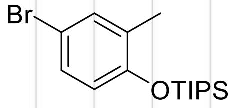

14
^{1}H NMR (400 MHz, $CDCl_3$)

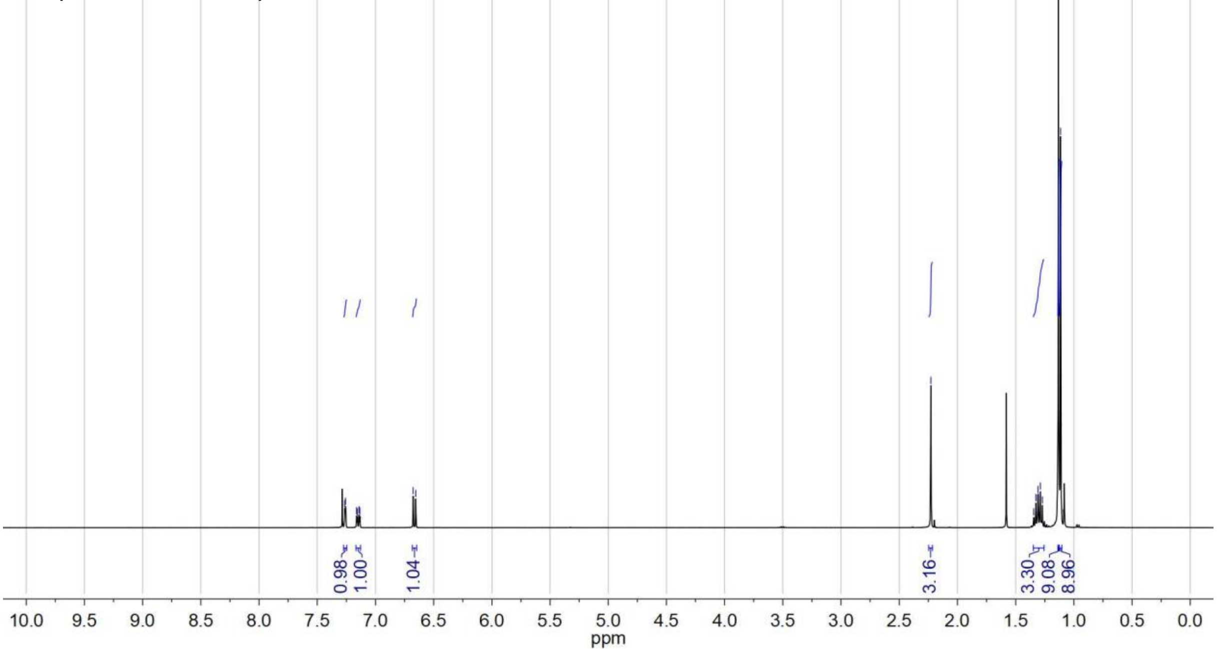

14
^{13}C NMR (101 MHz, $CDCl_3$)

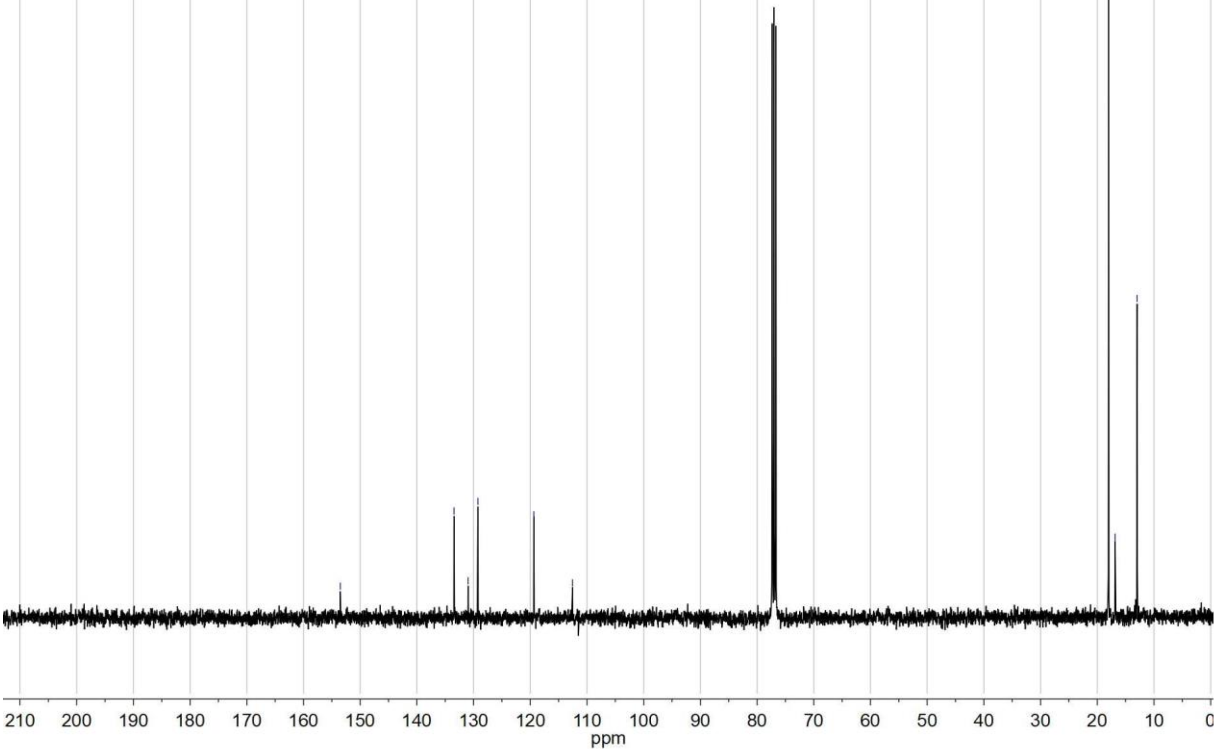

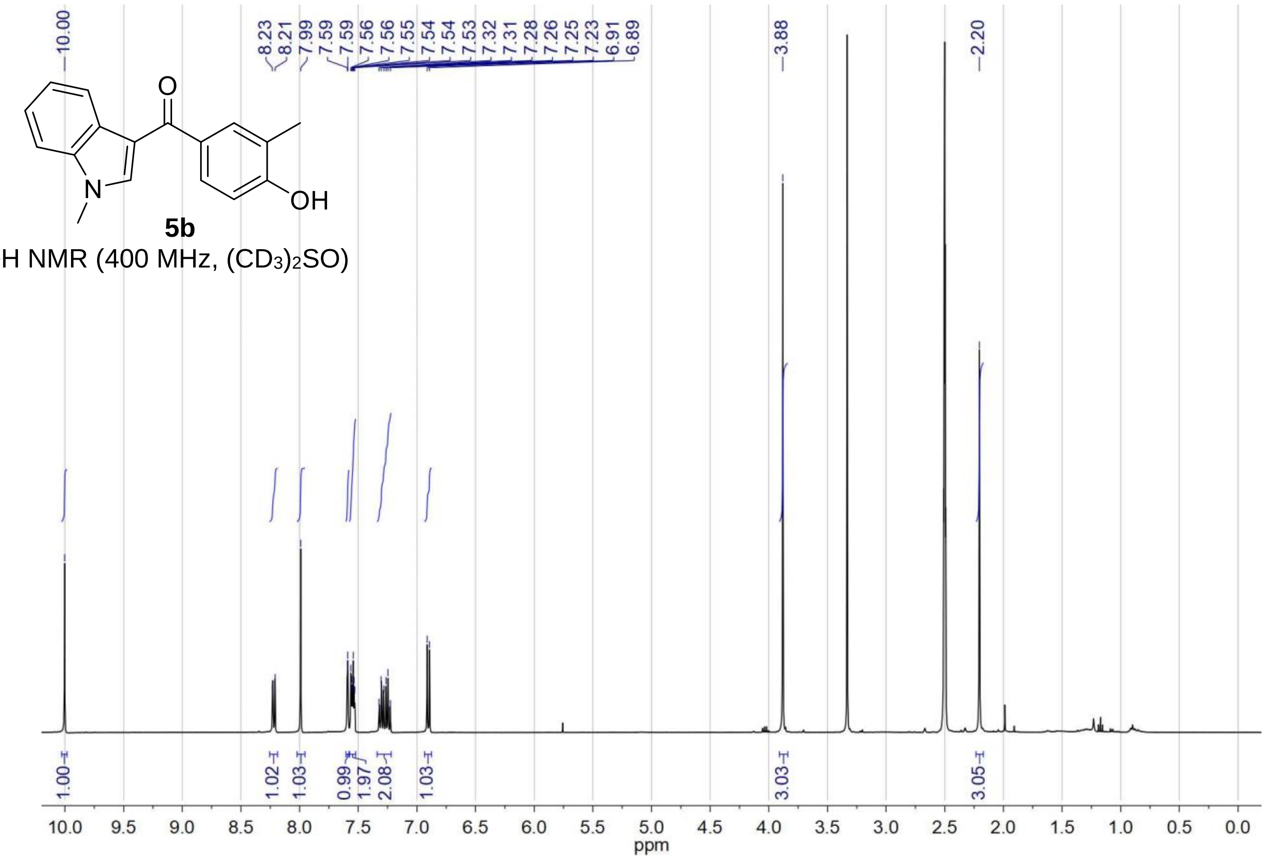

5b

^{1}H NMR (400 MHz, $(CD_3)_2SO$)

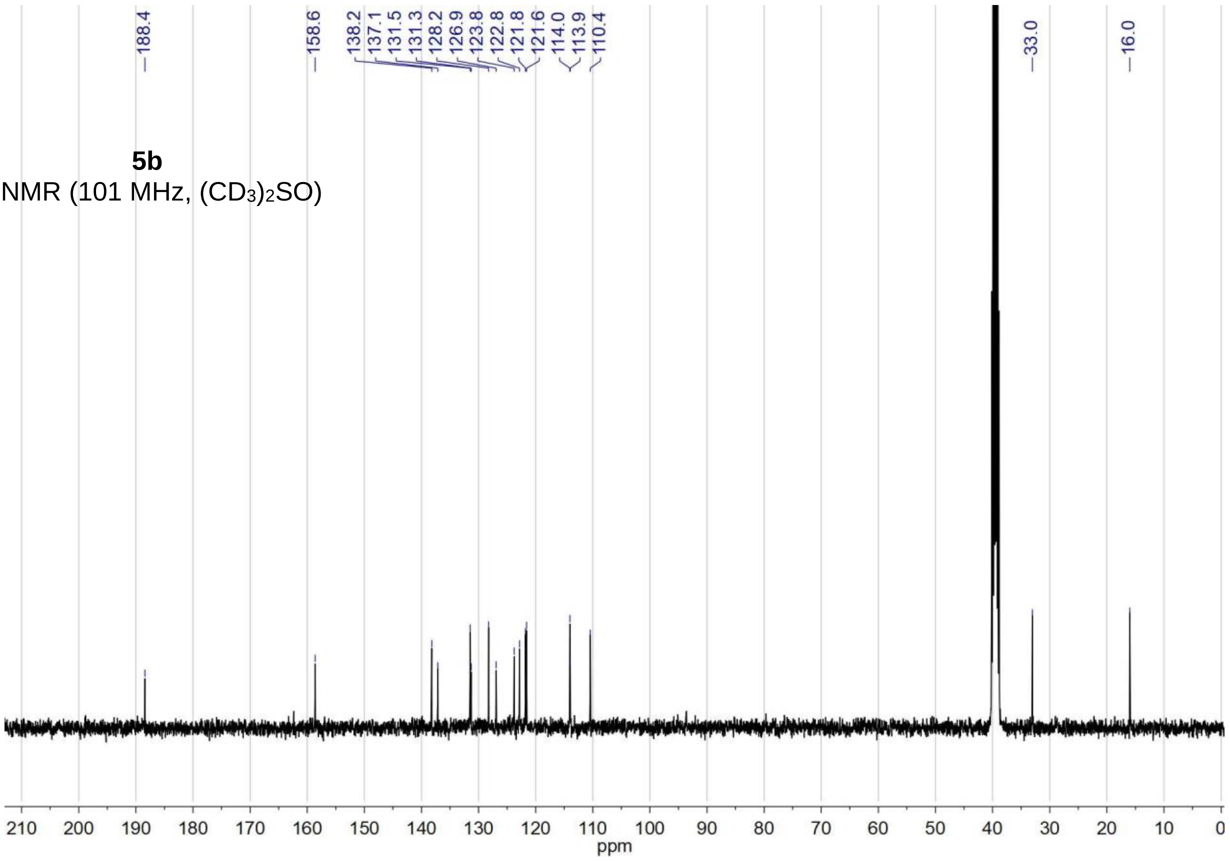

5b

^{13}C NMR (101 MHz, $(CD_3)_2SO$)

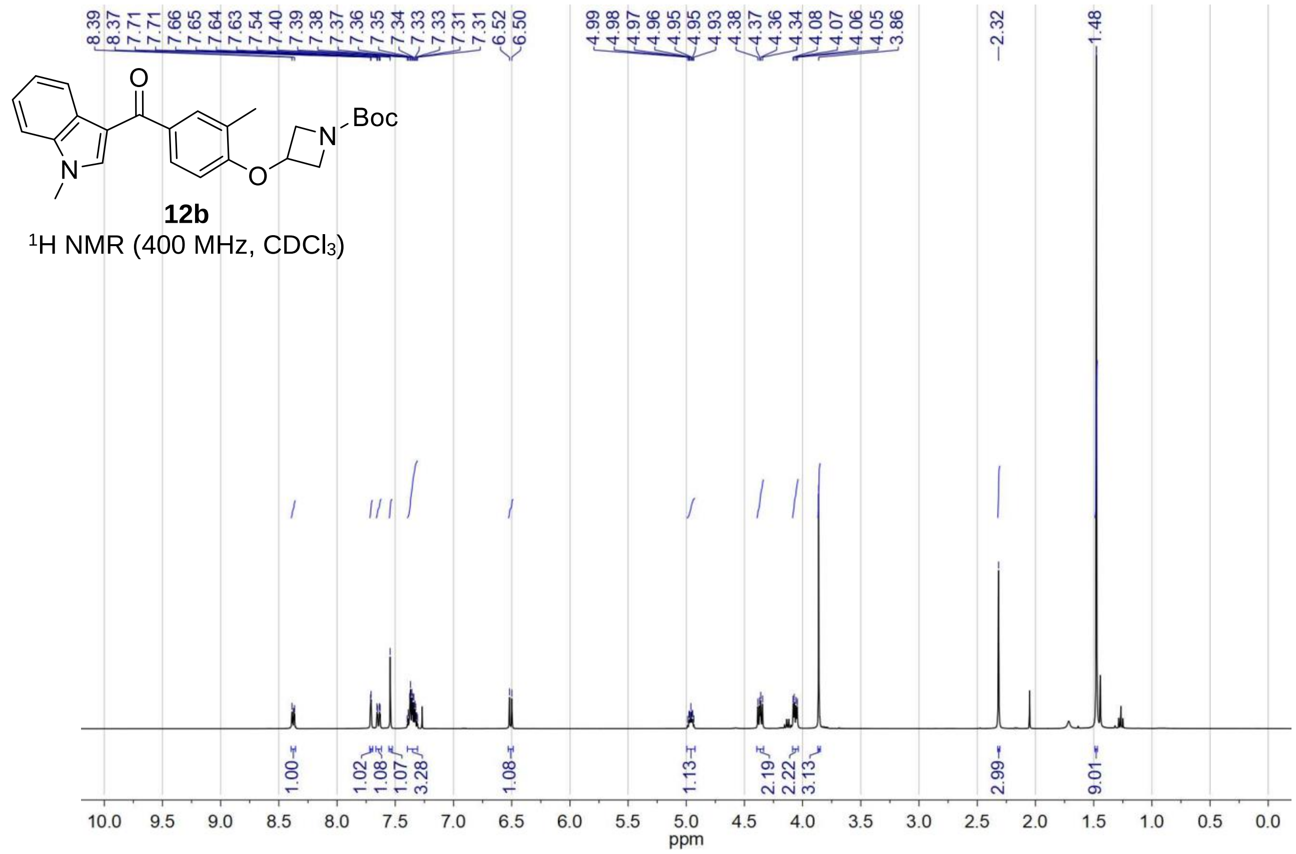

12b

^{1}H NMR (400 MHz, $CDCl_3$)

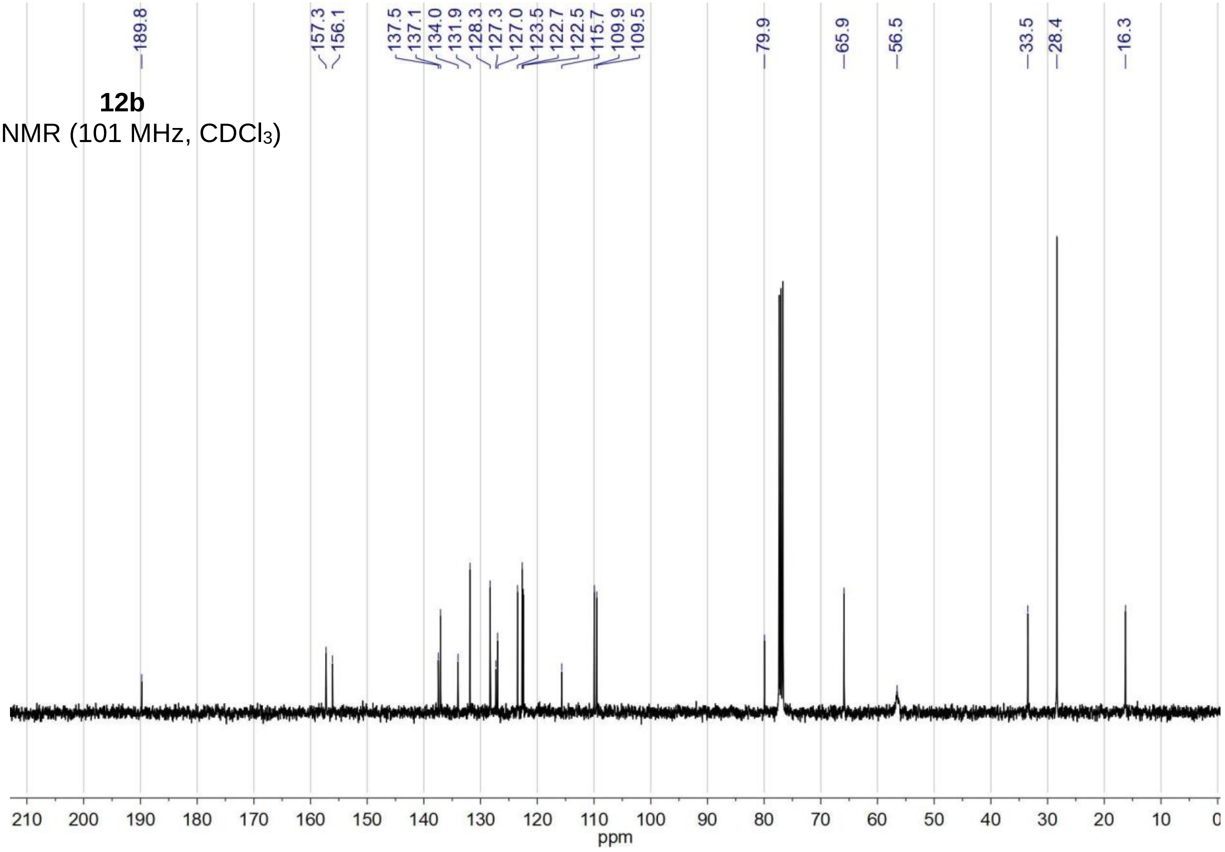

12b

^{13}C NMR (101 MHz, $CDCl_3$)

1b

^{1}H NMR (400 MHz, $CDCl_3$)

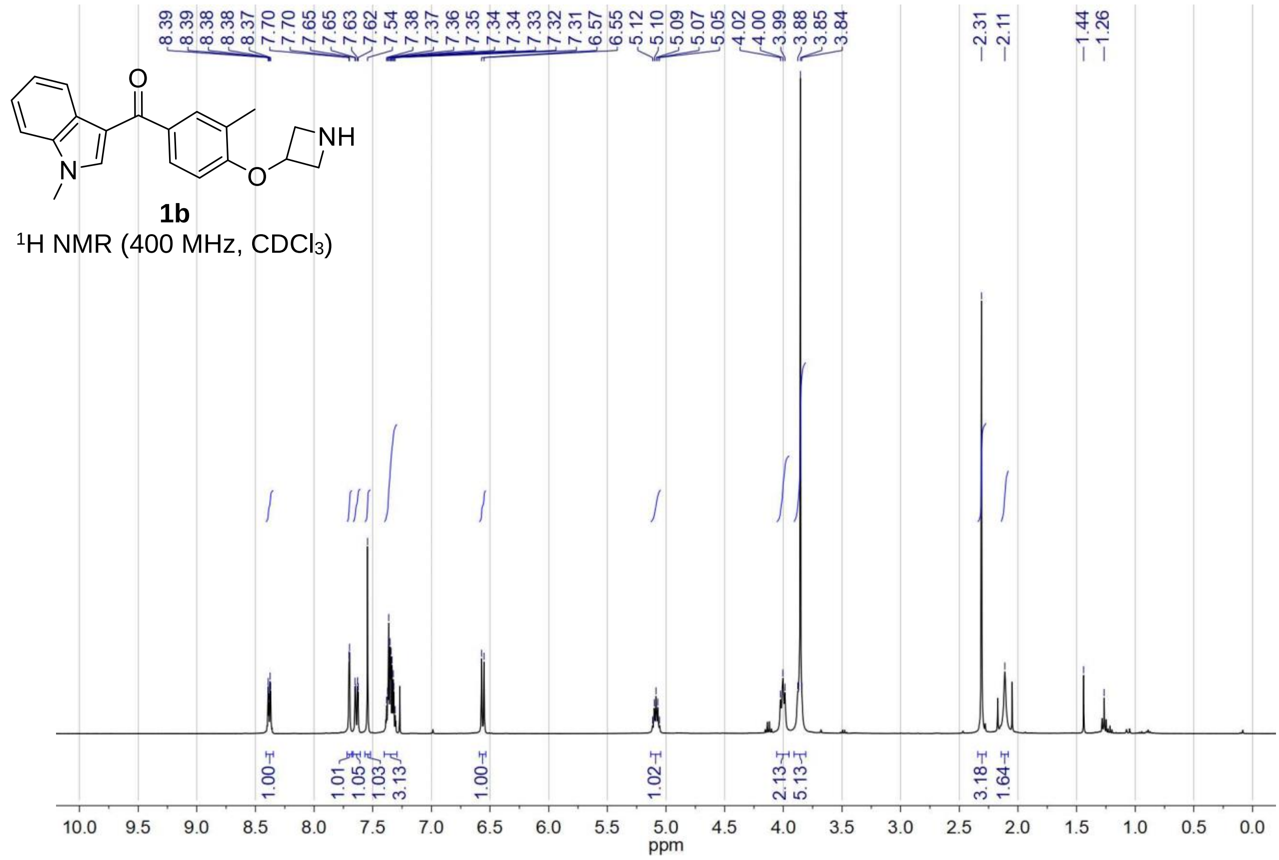

1b

^{13}C NMR (101 MHz, $CDCl_3$)

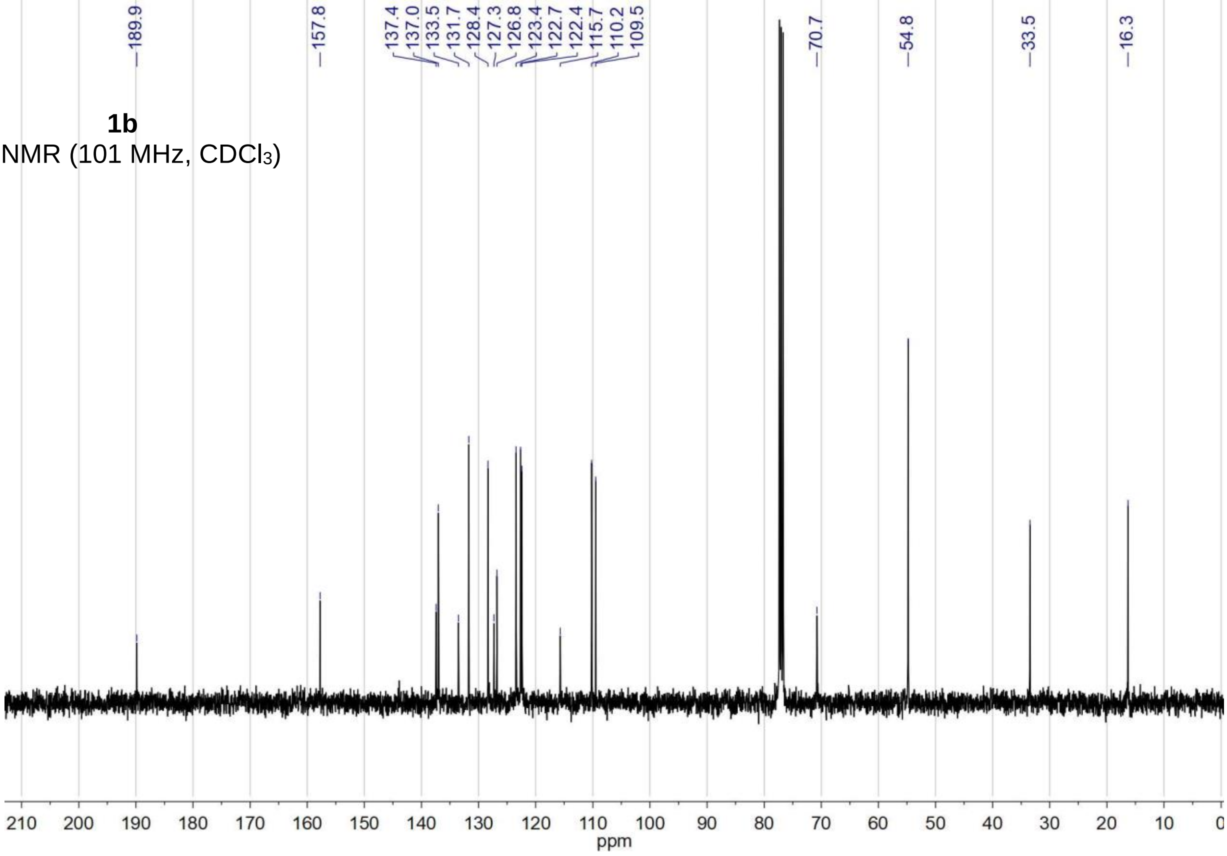

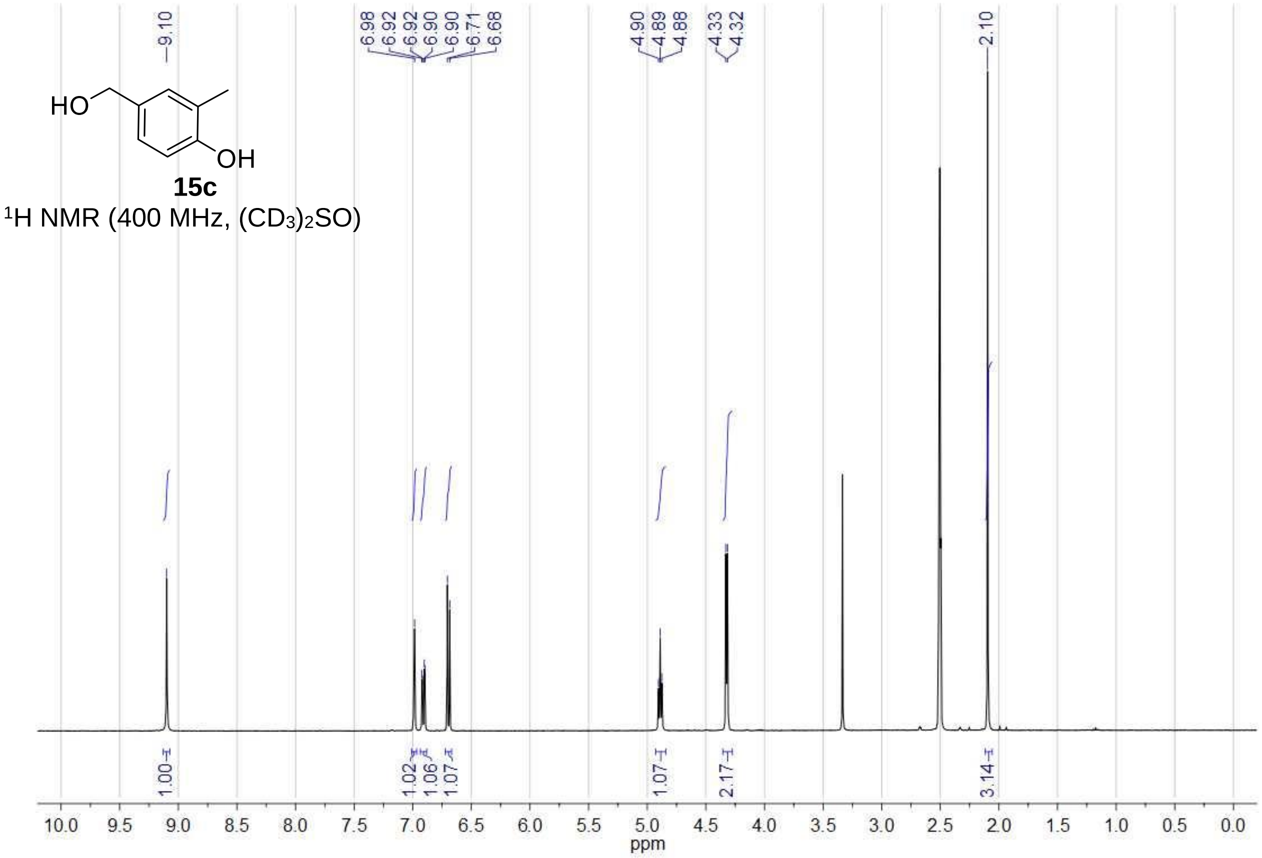
HO
OH
15c
1H NMR (400 MHz, (CD3)2SO)
9.10
6.98
6.92
6.92
6.90
6.90
6.71
6.68
4.90
4.89
4.88
4.33
4.32
2.10
1.00
1.02
1.06
1.07
1.07
2.17
3.14
ppm

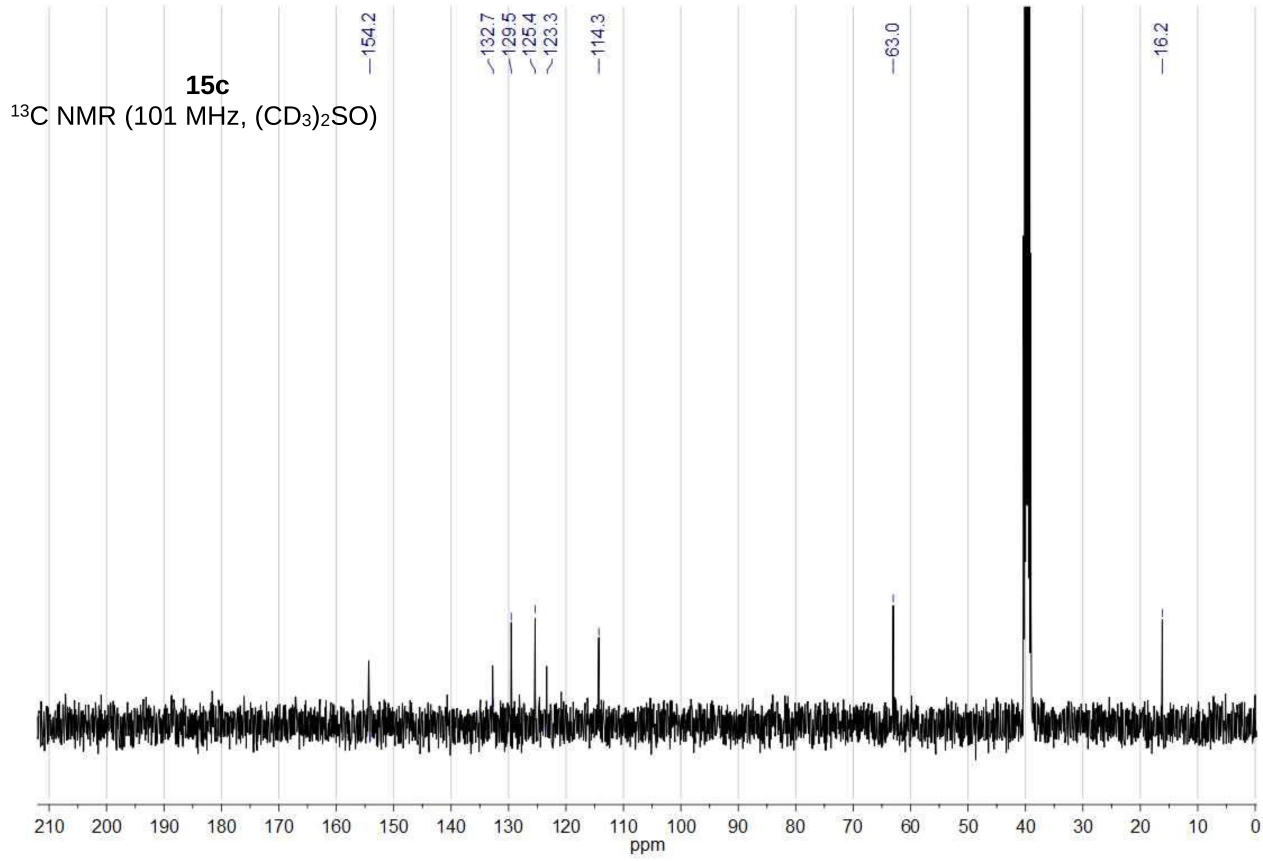
15c
13C NMR (101 MHz, (CD3)2SO)
154.2
132.7
129.5
125.4
123.3
114.3
63.0
16.2
ppm

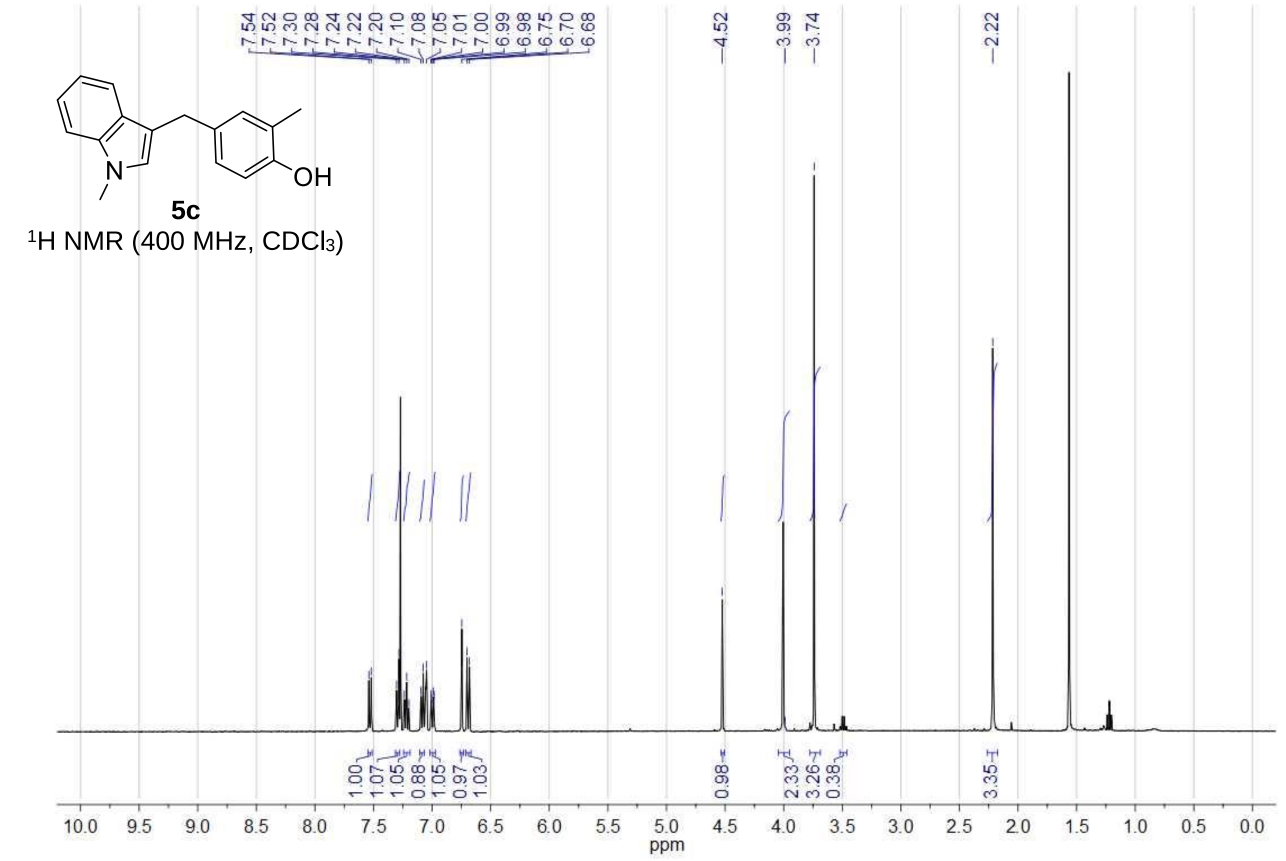
5c
^{1}H NMR (400 MHz, CDCl$_3$)
OH
N
7.54 7.52 7.30 7.28 7.24 7.22 7.20 7.10 7.08 7.05 7.01 7.00 6.99 6.98 6.75 6.70 6.68
4.52
3.99
3.74
2.22
1.00 1.07 1.05 0.88 1.05 0.97 1.03
0.98
2.33 3.26 0.38
3.35
ppm

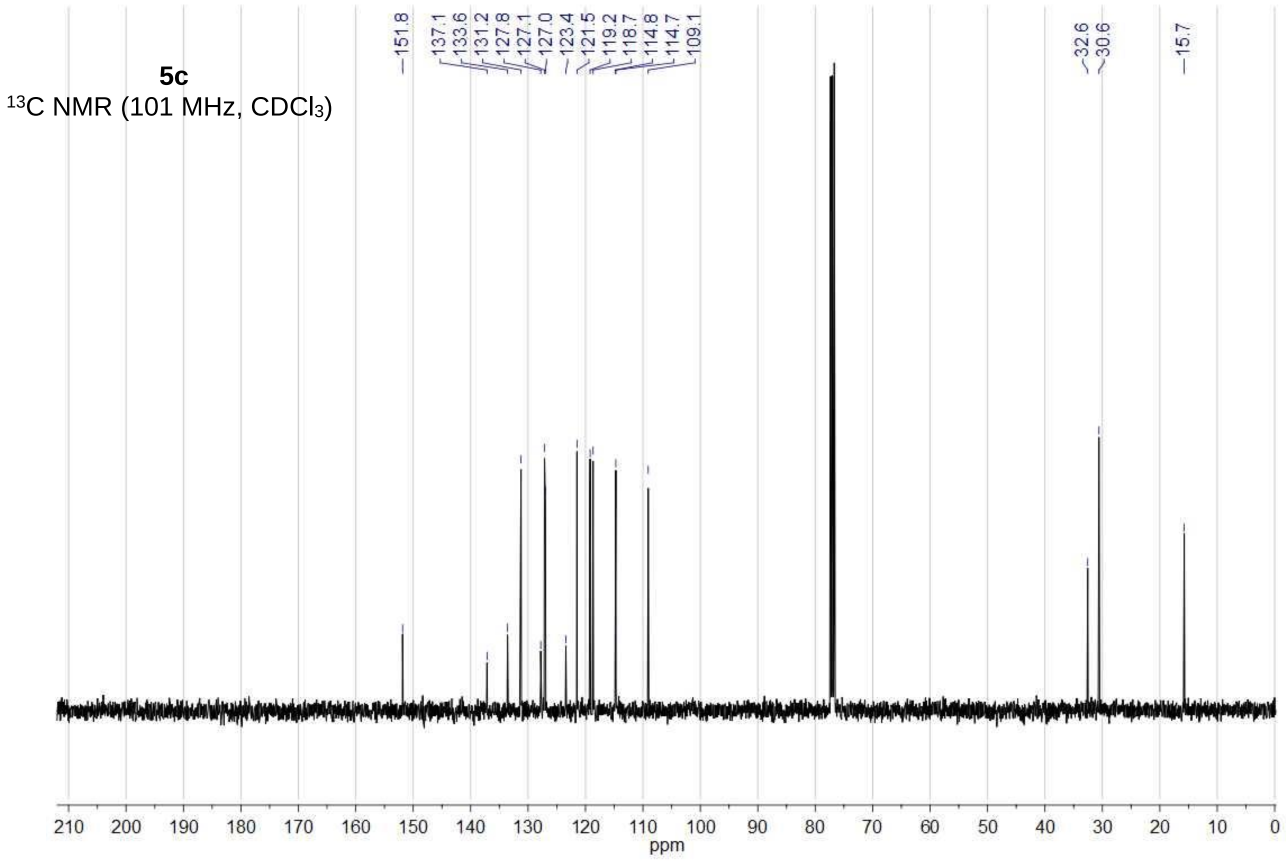
5c
^{13}C NMR (101 MHz, CDCl$_3$)
151.8
137.1 133.6 131.2 127.8 127.1 127.0 123.4 121.5 119.2 118.7 114.8 114.7 109.1
32.6 30.6
15.7
ppm

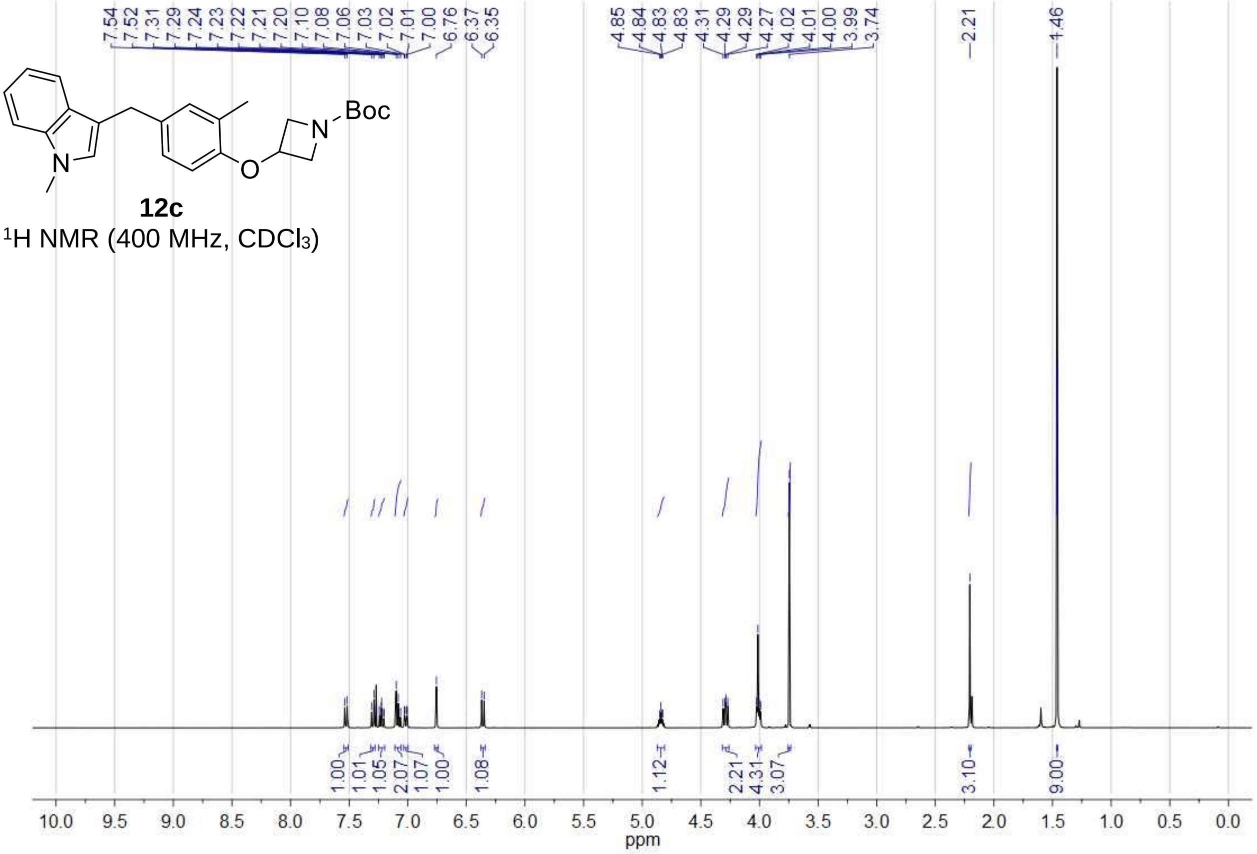

12c

^{1}H NMR (400 MHz, CDCl$_3$)

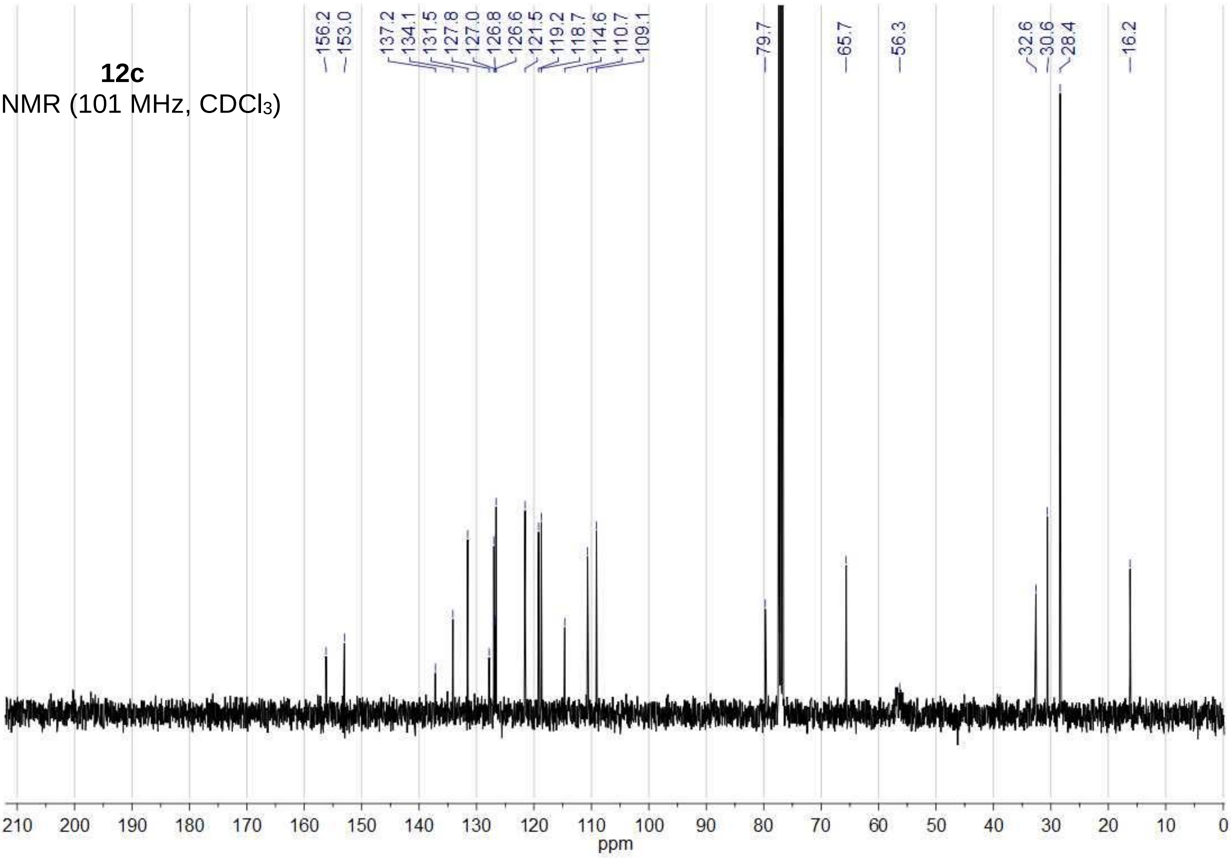

12c

^{13}C NMR (101 MHz, CDCl$_3$)

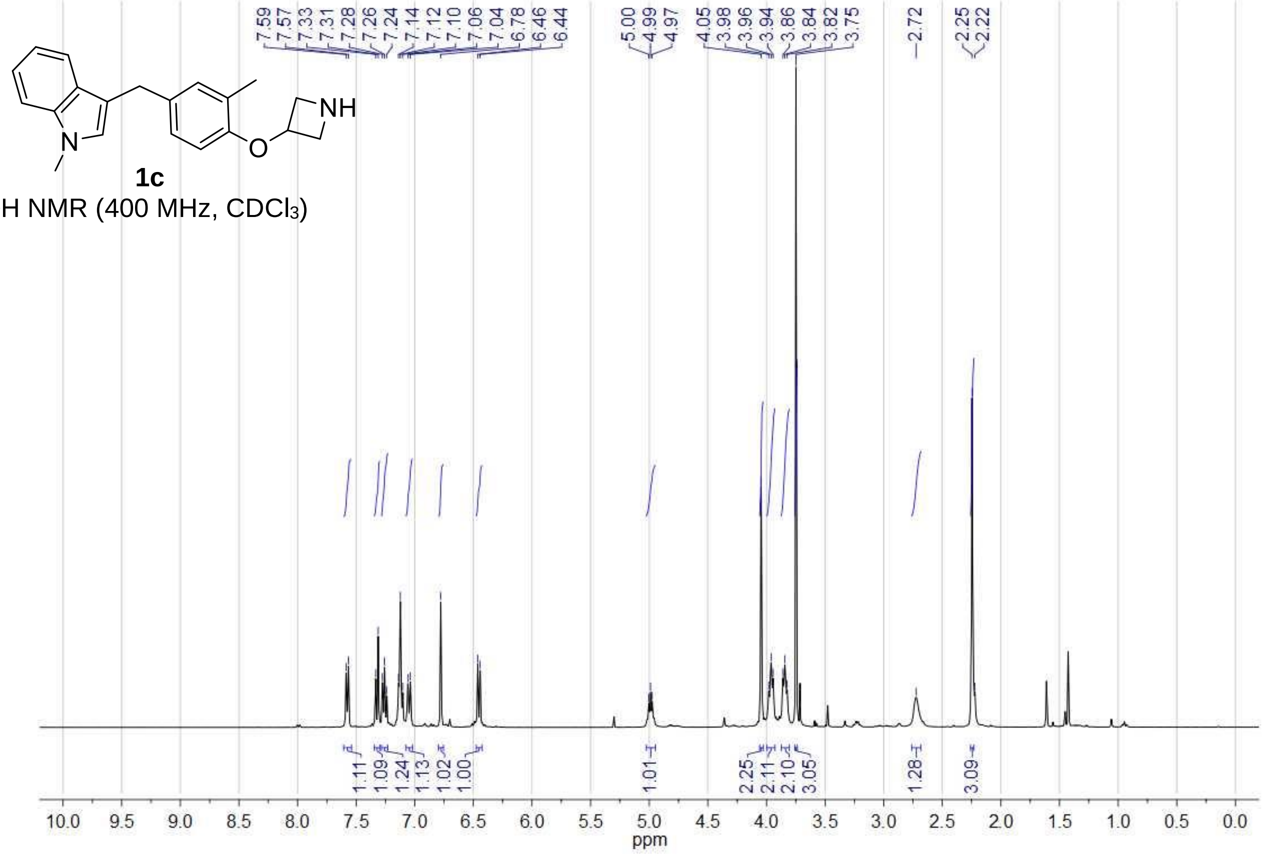

1c

^{1}H NMR (400 MHz, $CDCl_3$)

1c

^{13}C NMR (101 MHz, $CDCl_3$)

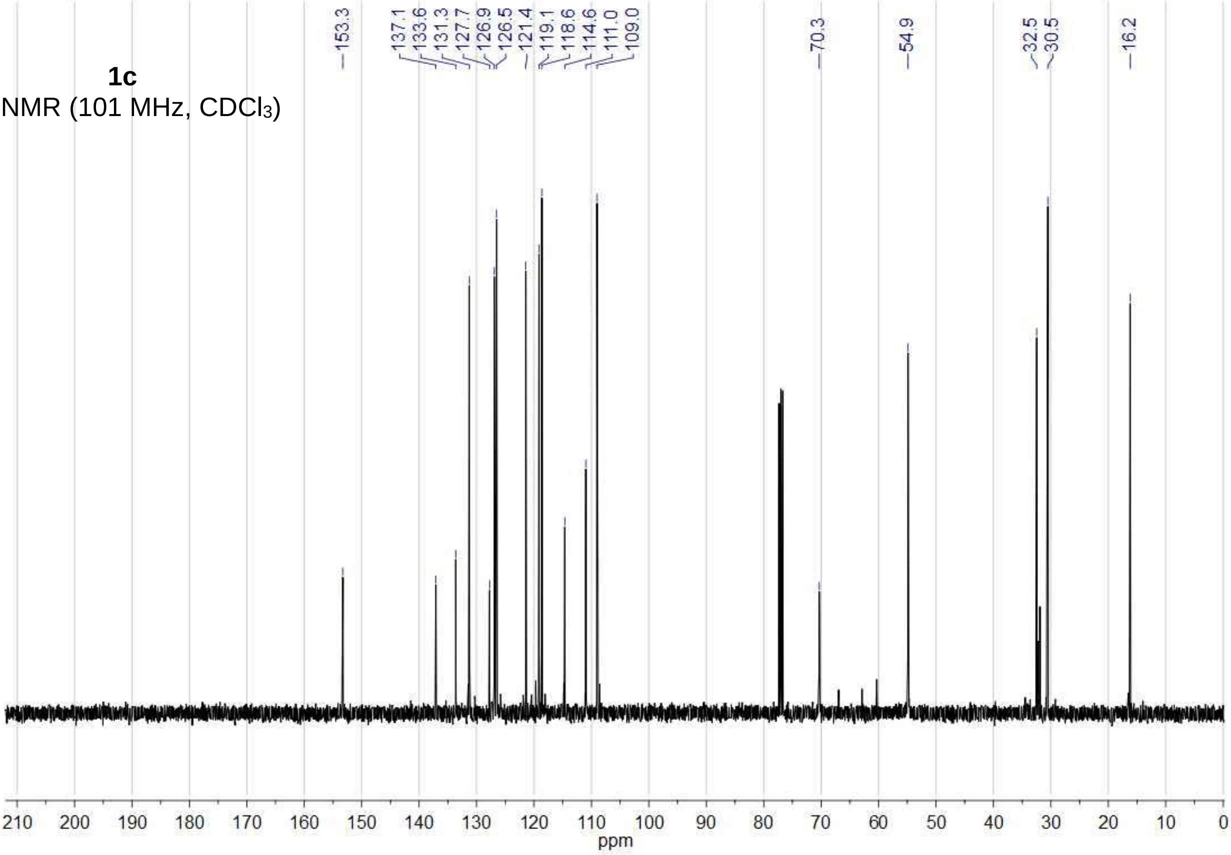

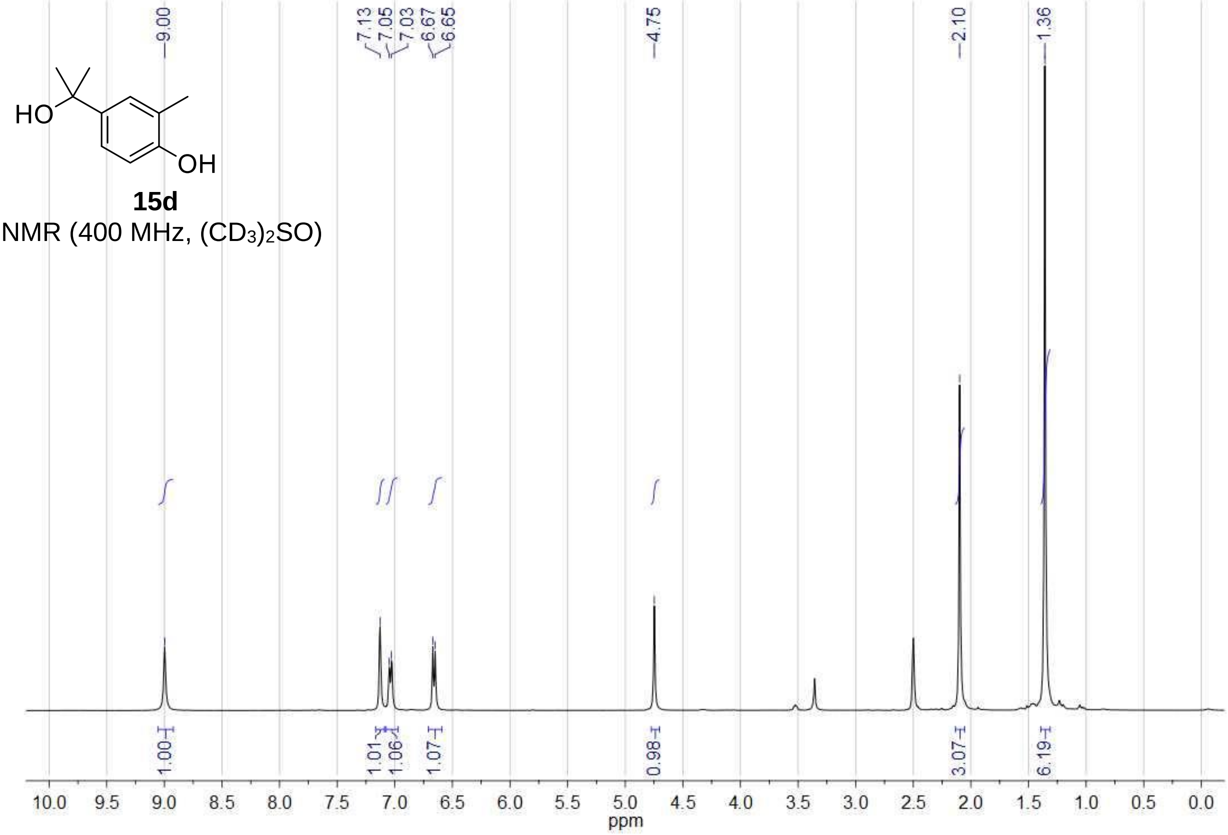

15d

^{1}H NMR (400 MHz, $(CD_3)_2SO$)

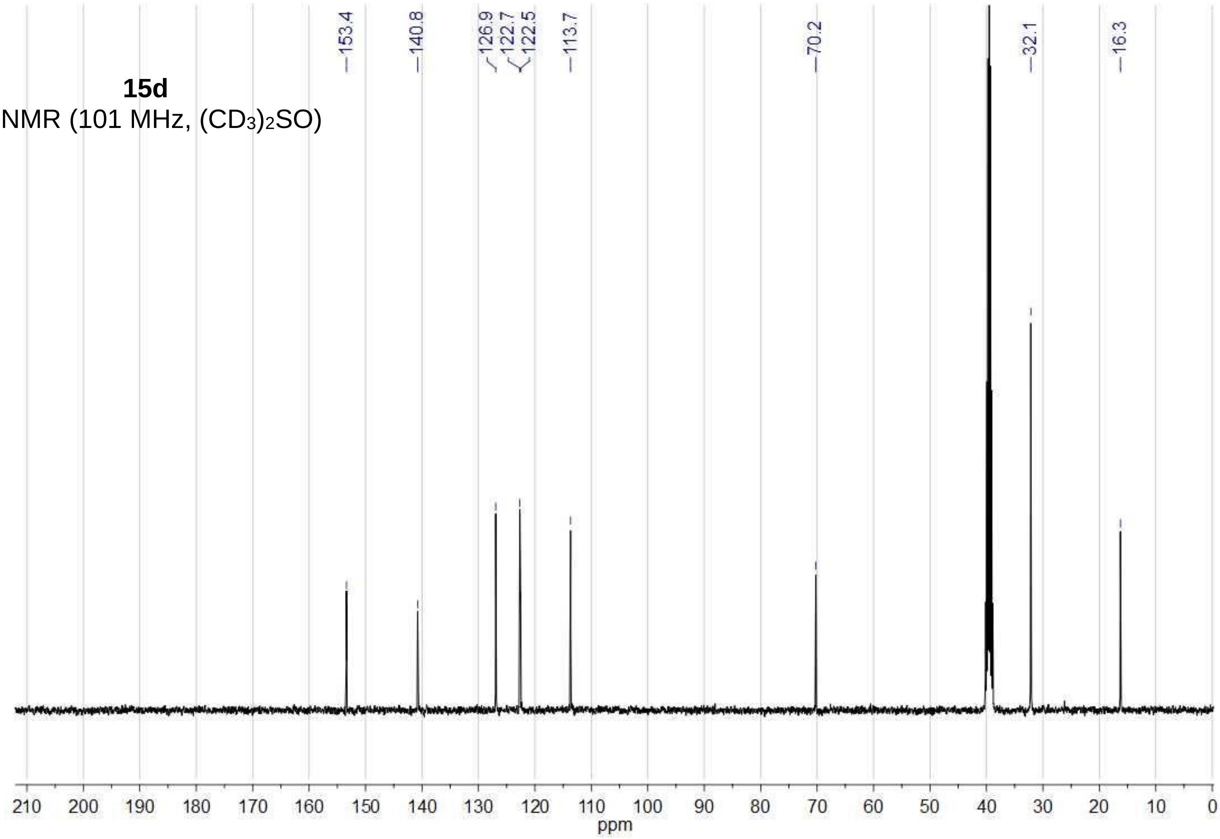

15d

^{13}C NMR (101 MHz, $(CD_3)_2SO$)

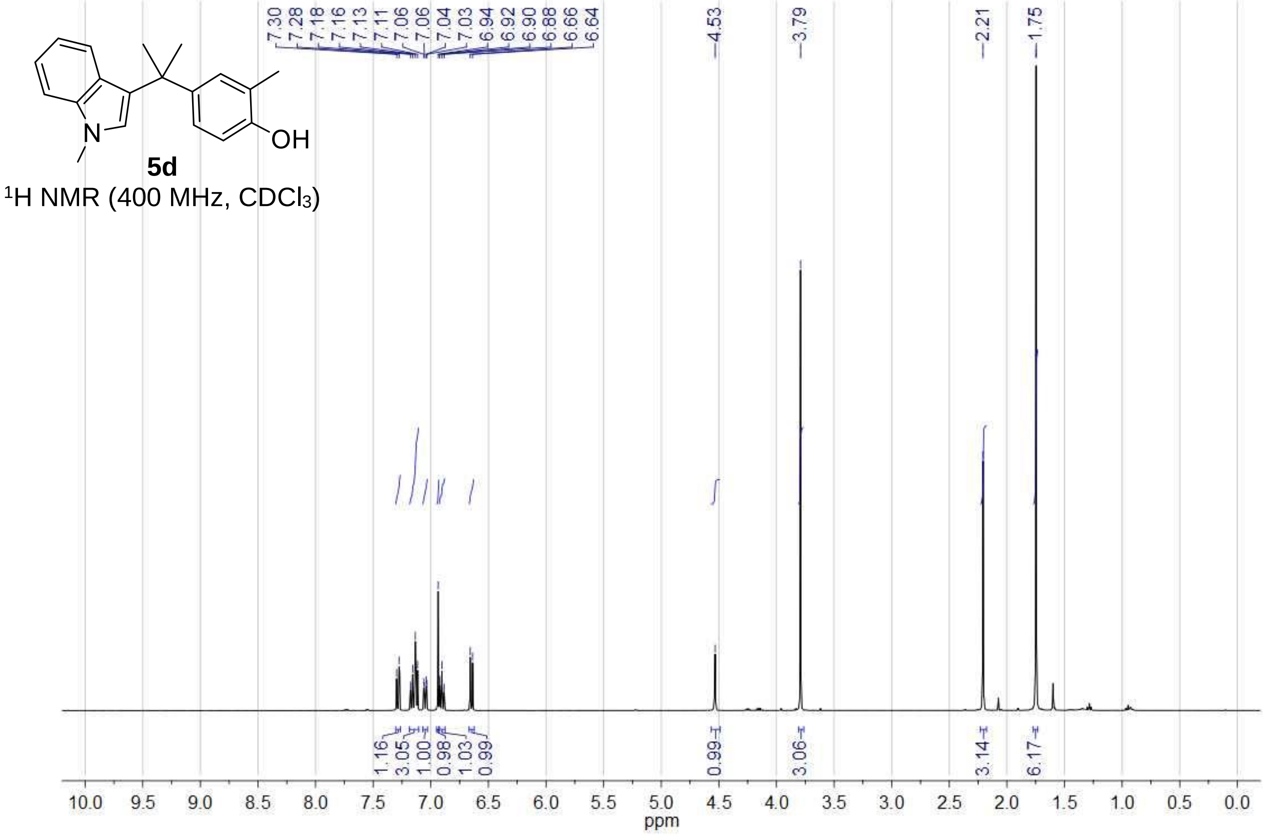
5d
1H NMR (400 MHz, CDCl3)
OH
N
7.30 7.28 7.18 7.16 7.13 7.11 7.06 7.06 7.04 7.03 6.94 6.92 6.90 6.88 6.66 6.64
4.53
3.79
2.21
1.75
1.16 3.05 1.00 0.98 1.03 0.99
0.99
3.06
3.14
6.17
ppm

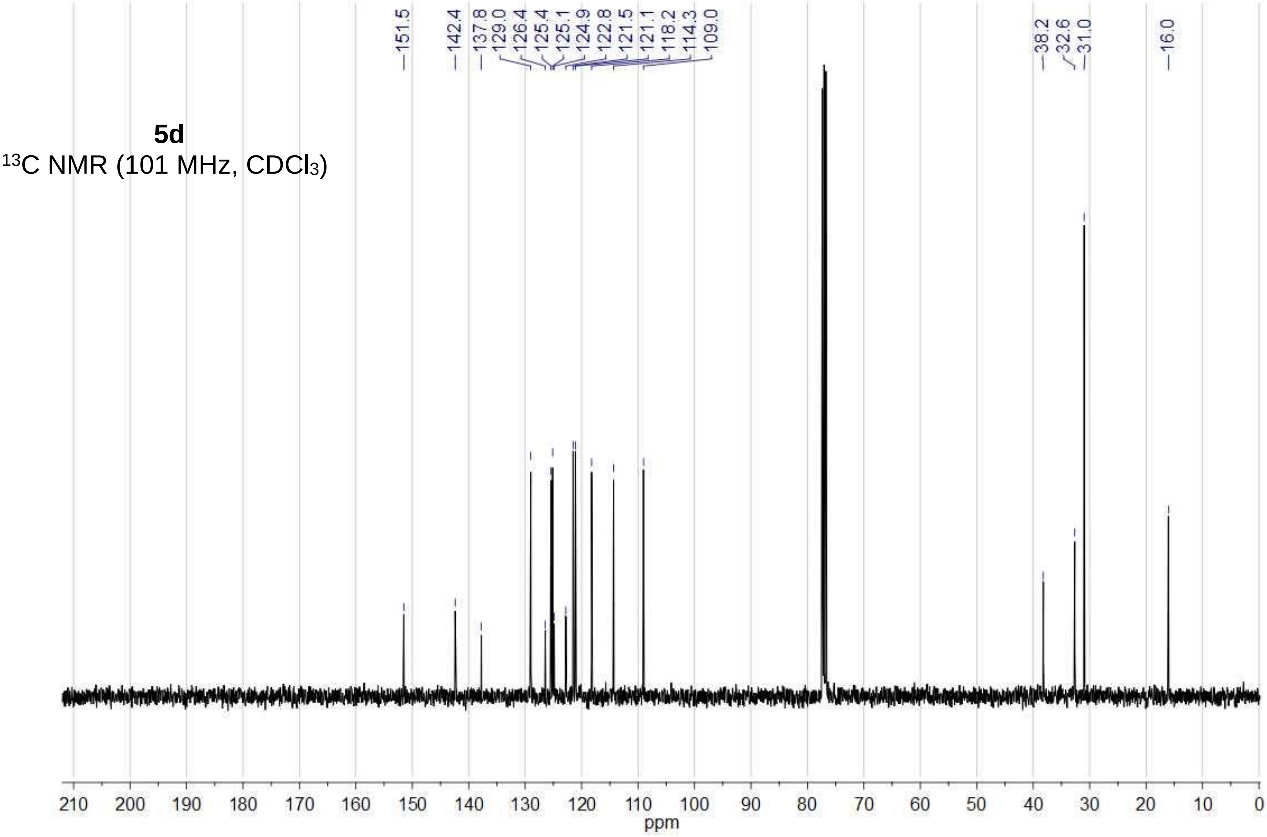
5d
13C NMR (101 MHz, CDCl3)
151.5
142.4 137.8 129.0 126.4 125.4 125.1 124.9 122.8 121.5 121.1 118.2 114.3 109.0
38.2 32.6 31.0
16.0
ppm

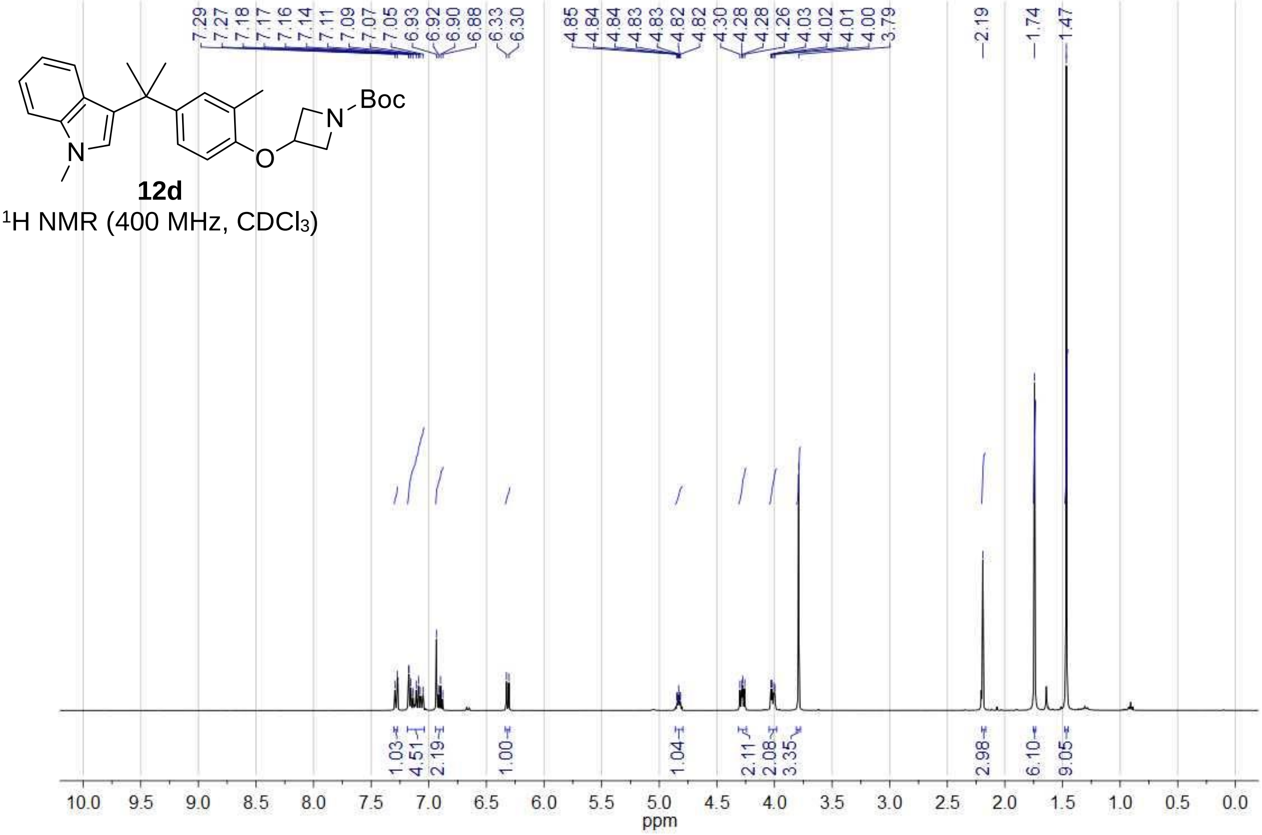

12d

^{1}H NMR (400 MHz, $CDCl_3$)

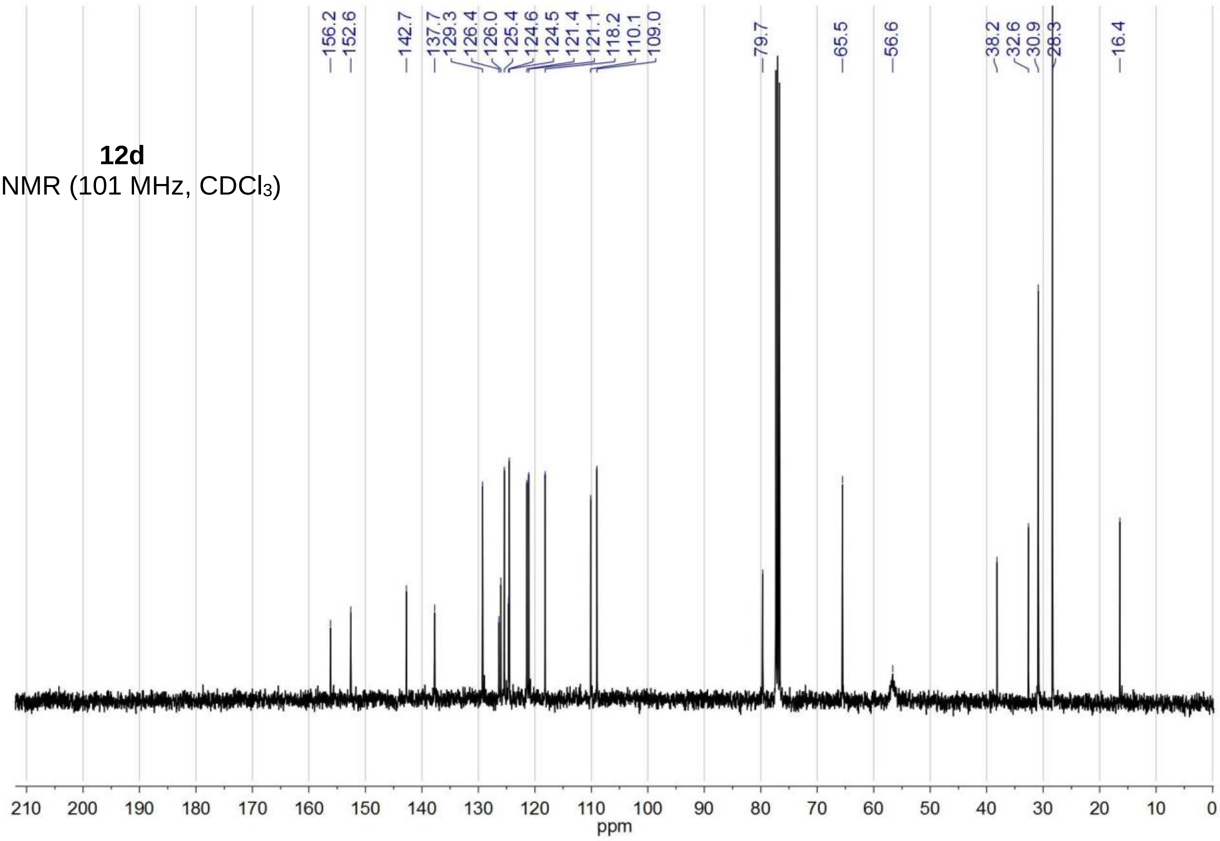

12d

^{13}C NMR (101 MHz, $CDCl_3$)

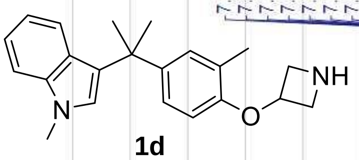

1d

^{1}H NMR (400 MHz, $CDCl_3$)

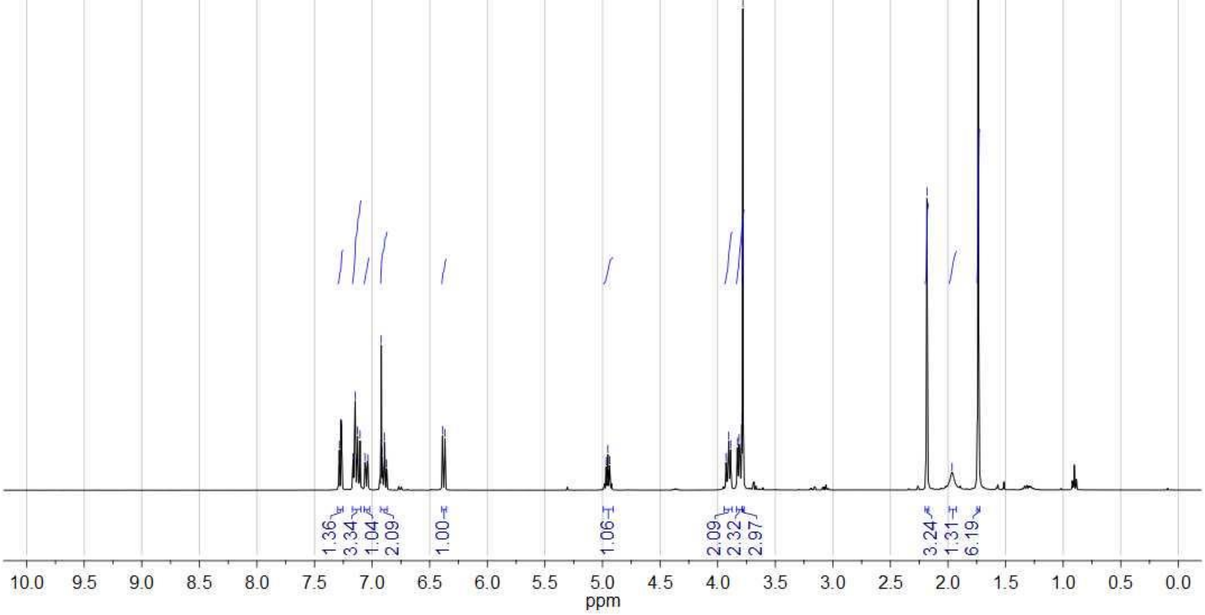

1d

^{13}C NMR (101 MHz, $CDCl_3$)

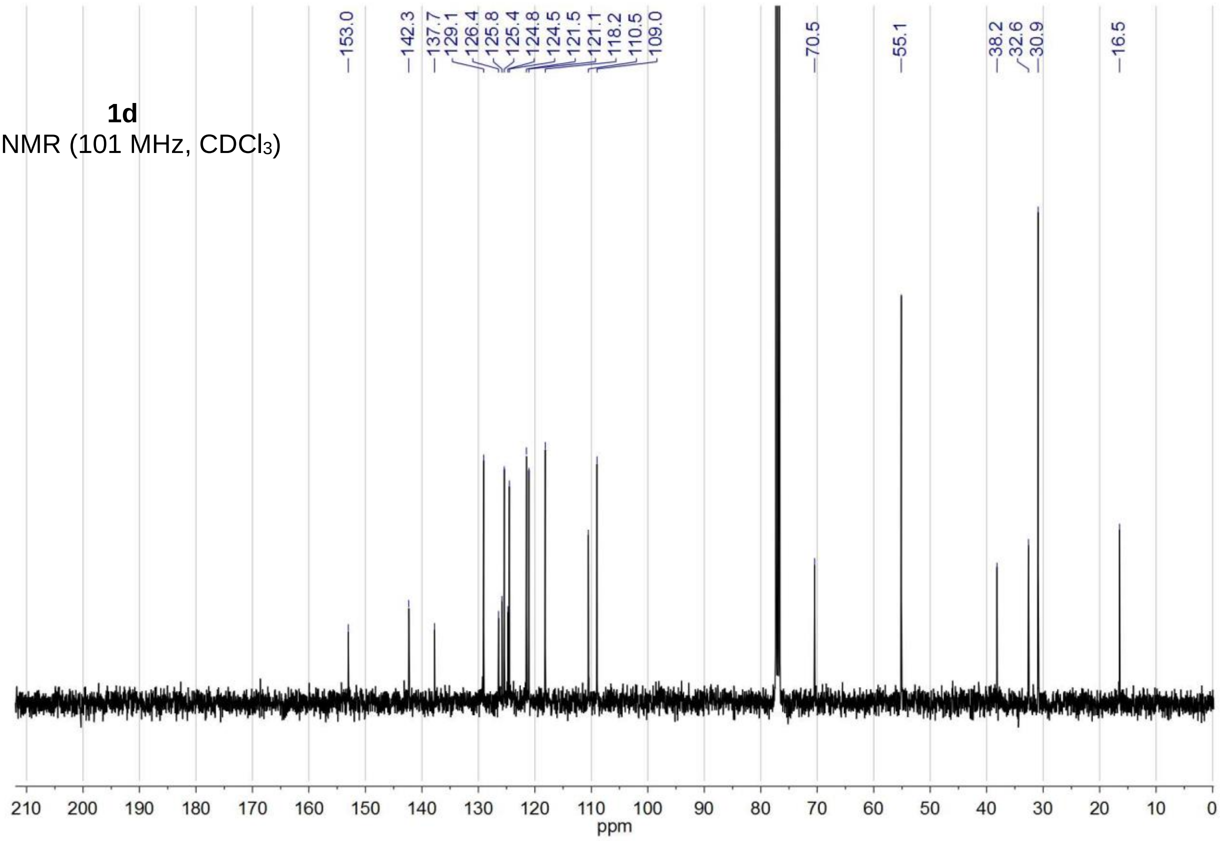

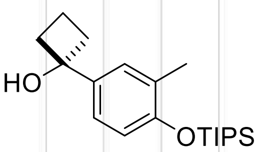

16e

^{1}H NMR (400 MHz, $CDCl_3$)

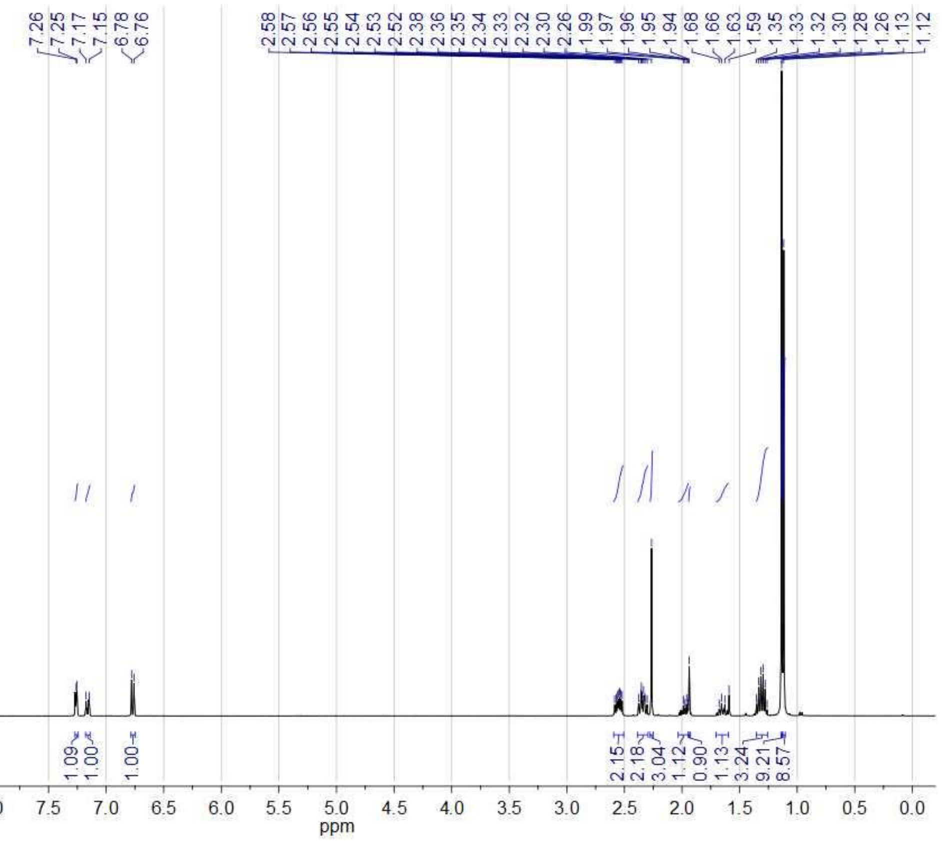

16e

^{13}C NMR (101 MHz, $CDCl_3$)

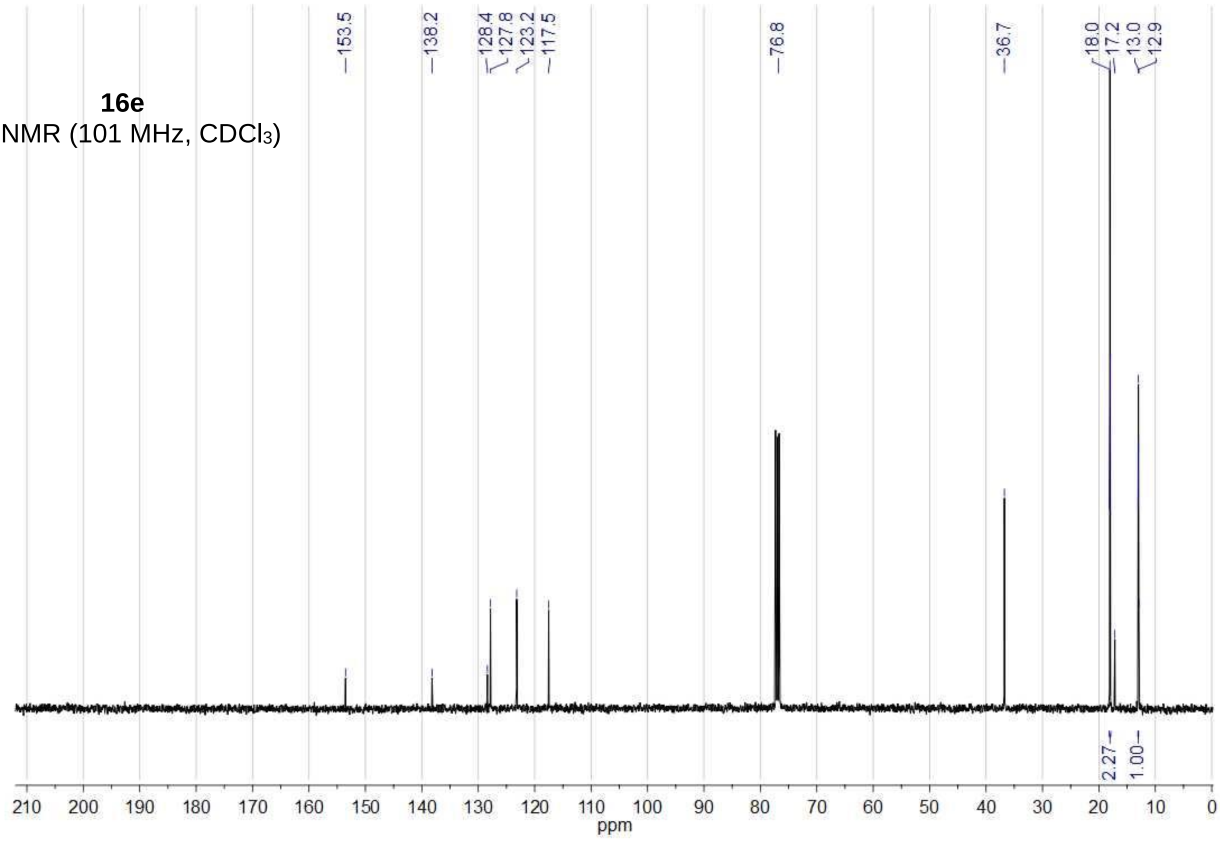

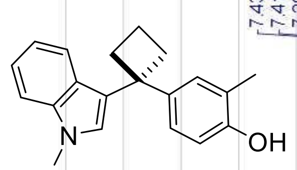

5e

^{1}H NMR (400 MHz, $CDCl_3$)

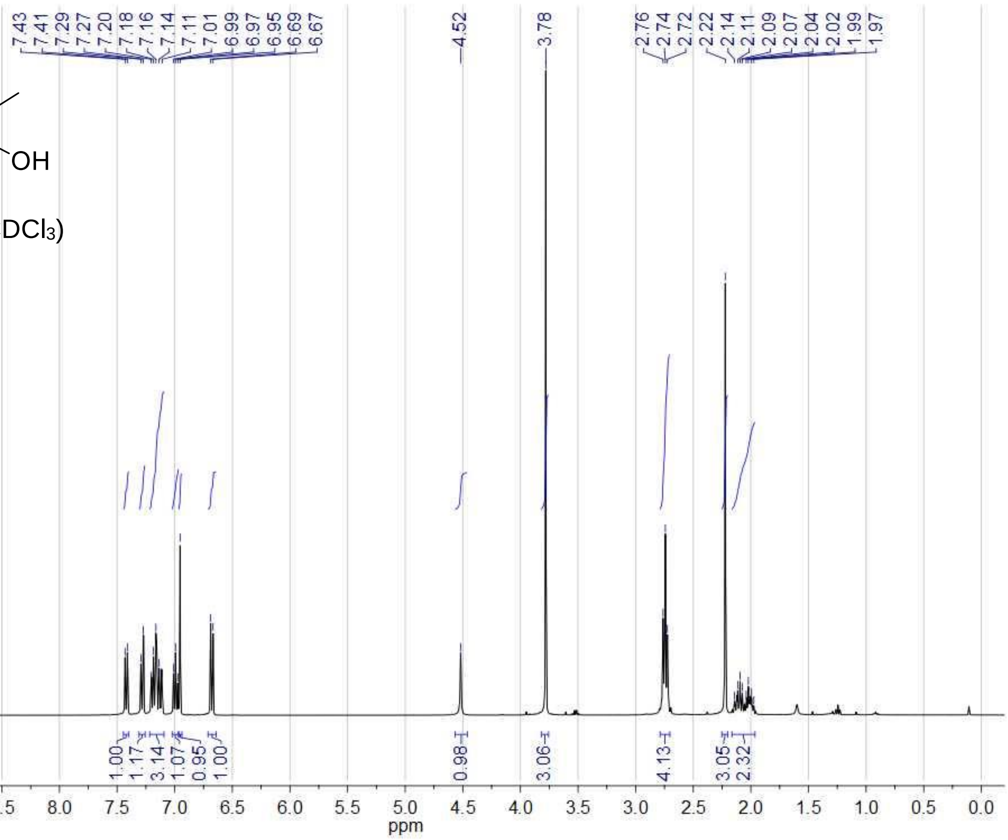

5e

^{13}C NMR (101 MHz, $CDCl_3$)

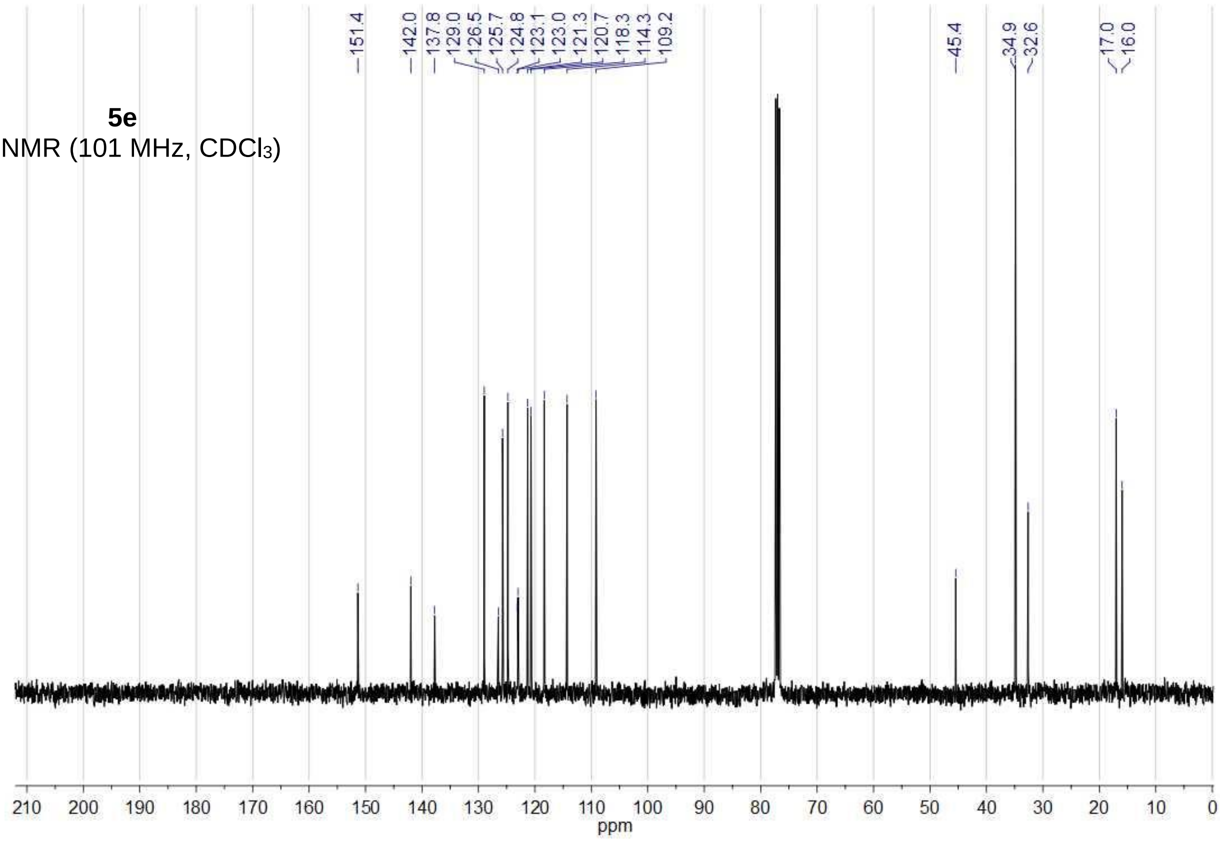

12e

^{1}H NMR (400 MHz, $CDCl_3$)

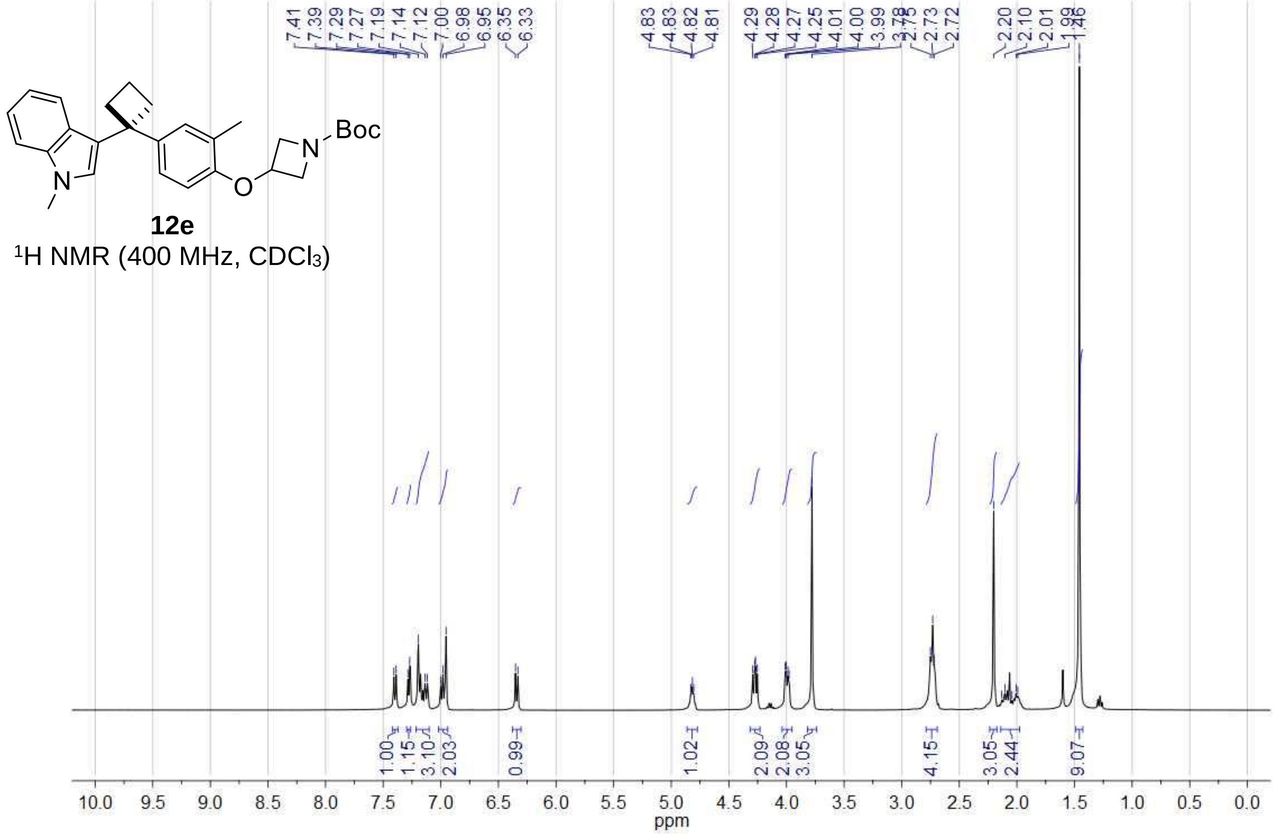

12e

^{13}C NMR (101 MHz, $CDCl_3$)

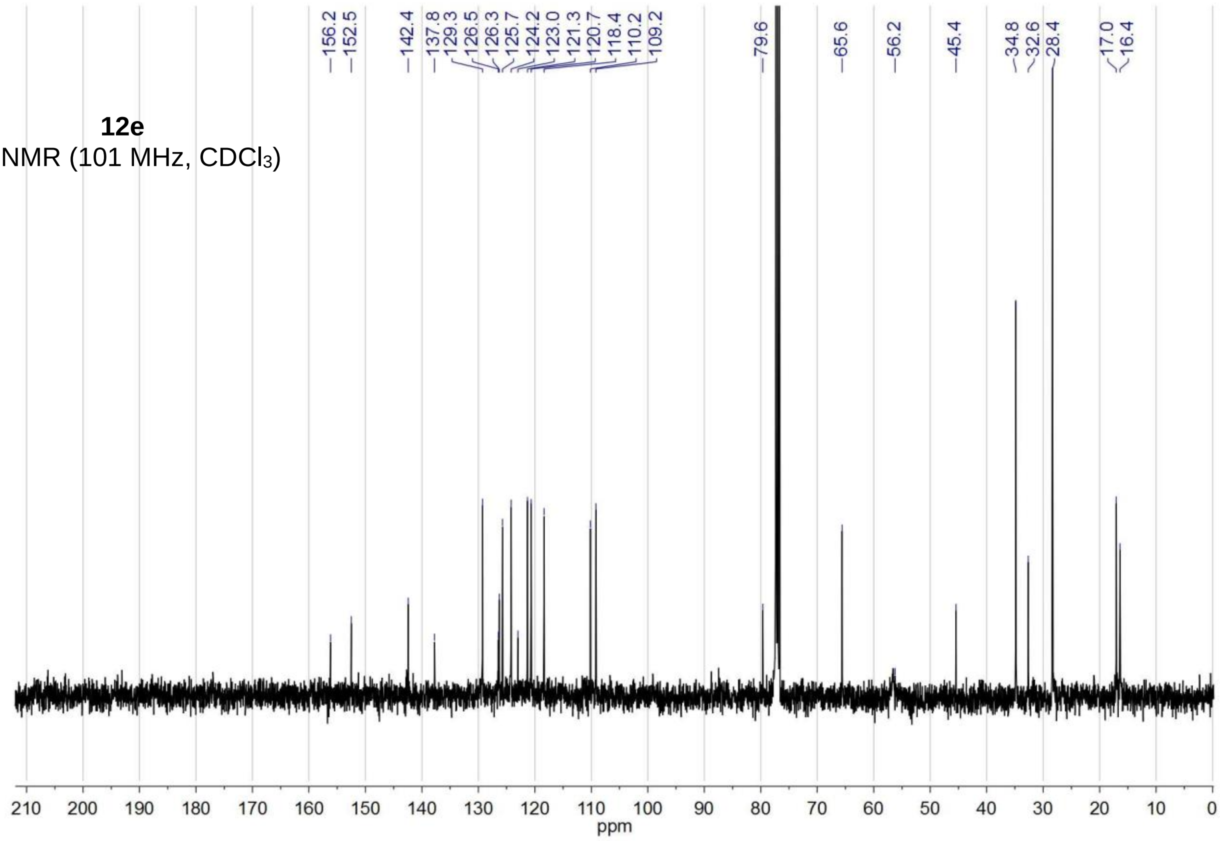

1e

^{1}H NMR (400 MHz, $CDCl_3$)

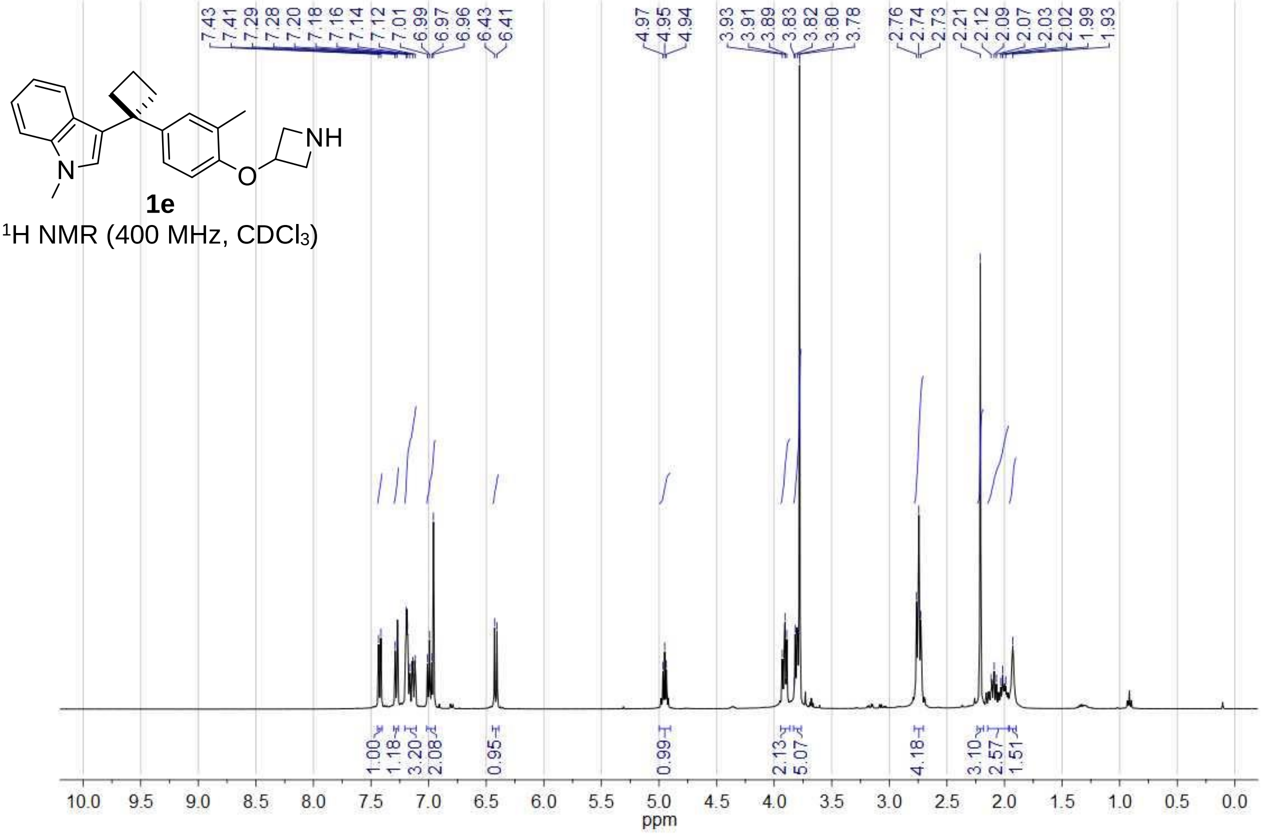

1e

^{13}C NMR (101 MHz, $CDCl_3$)

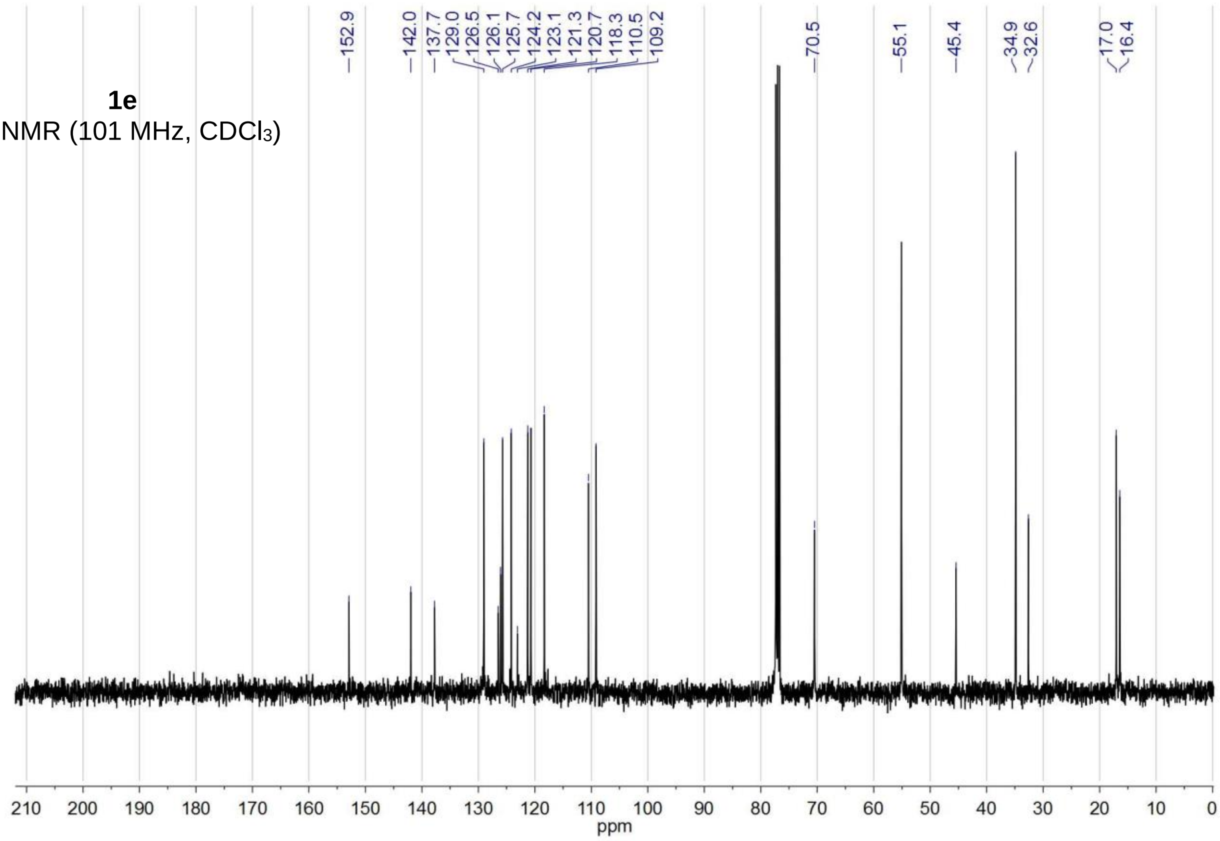

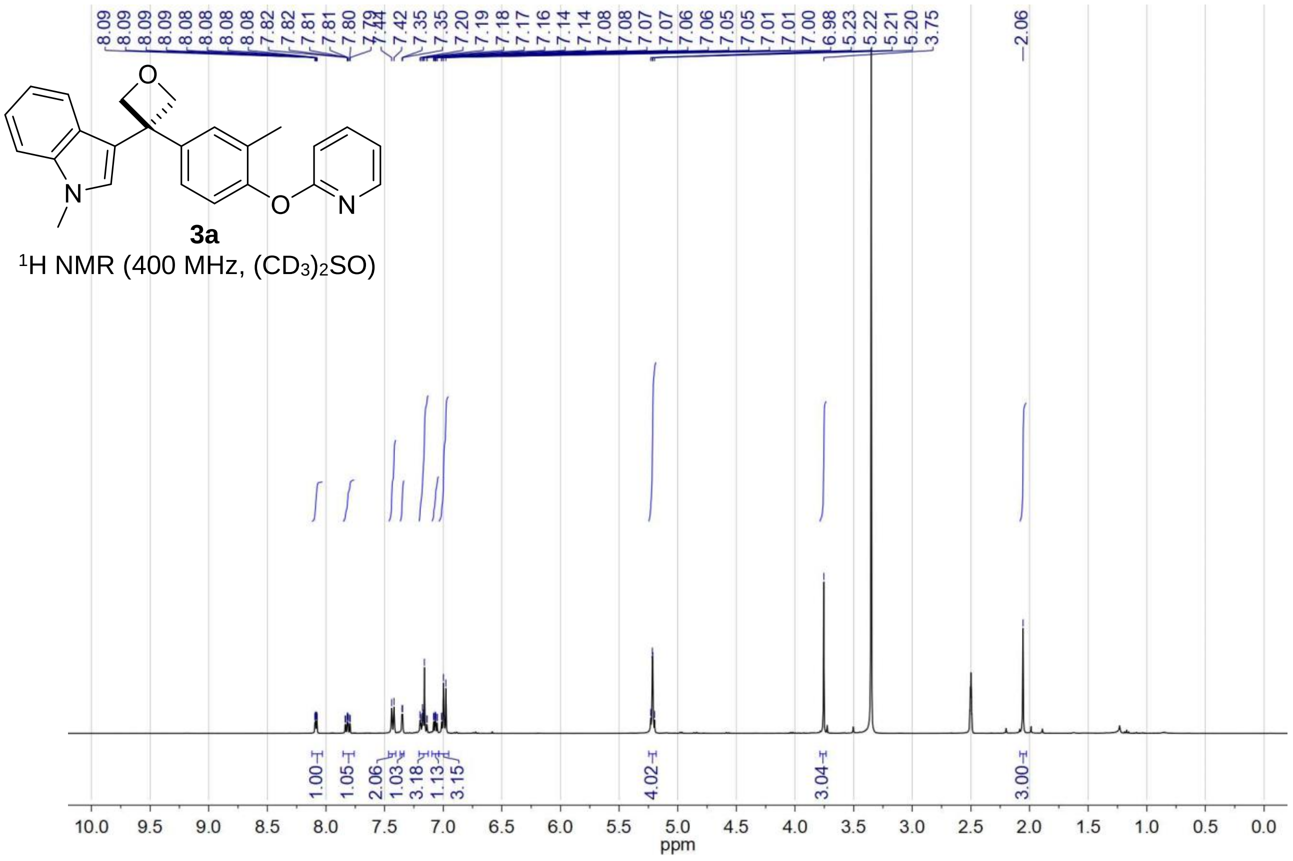

3a

^{1}H NMR (400 MHz, $(CD_3)_2SO$)

3a

^{13}C NMR (101 MHz, $(CD_3)_2SO$)

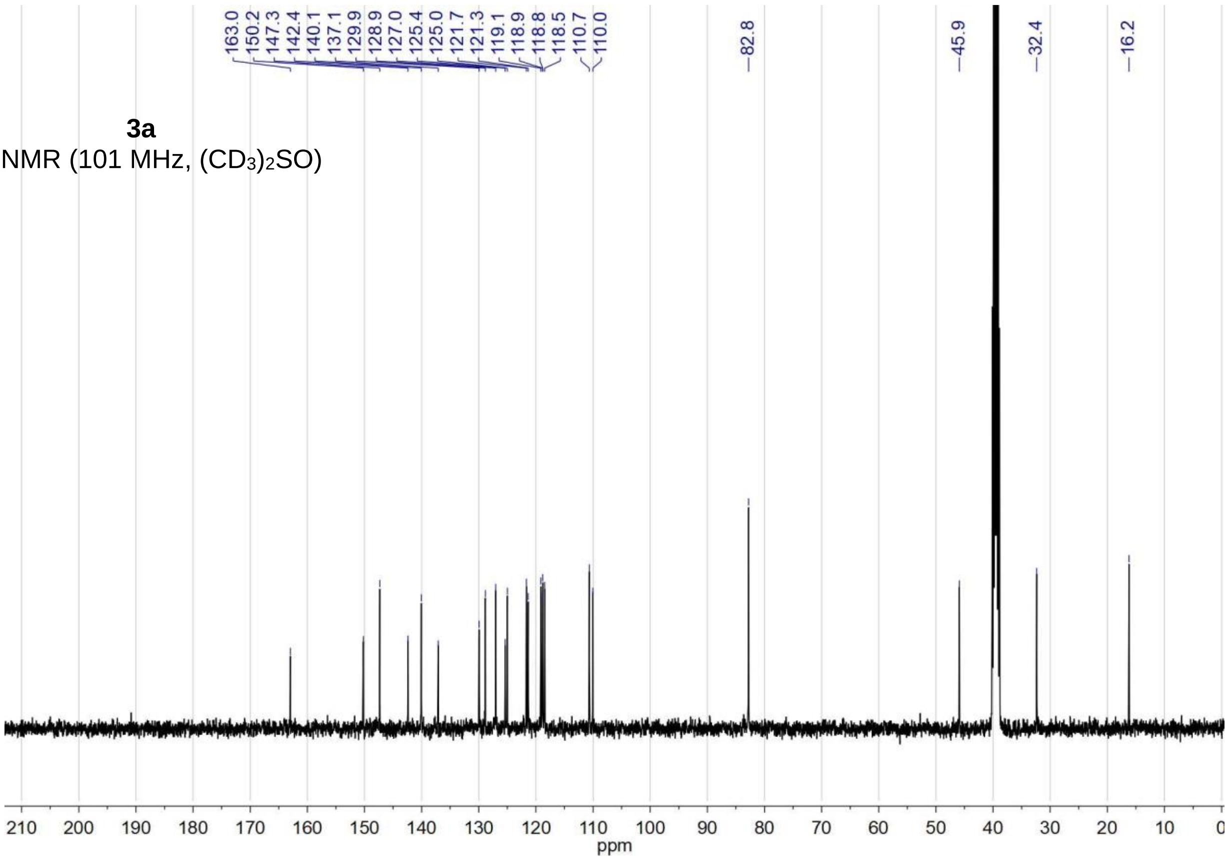

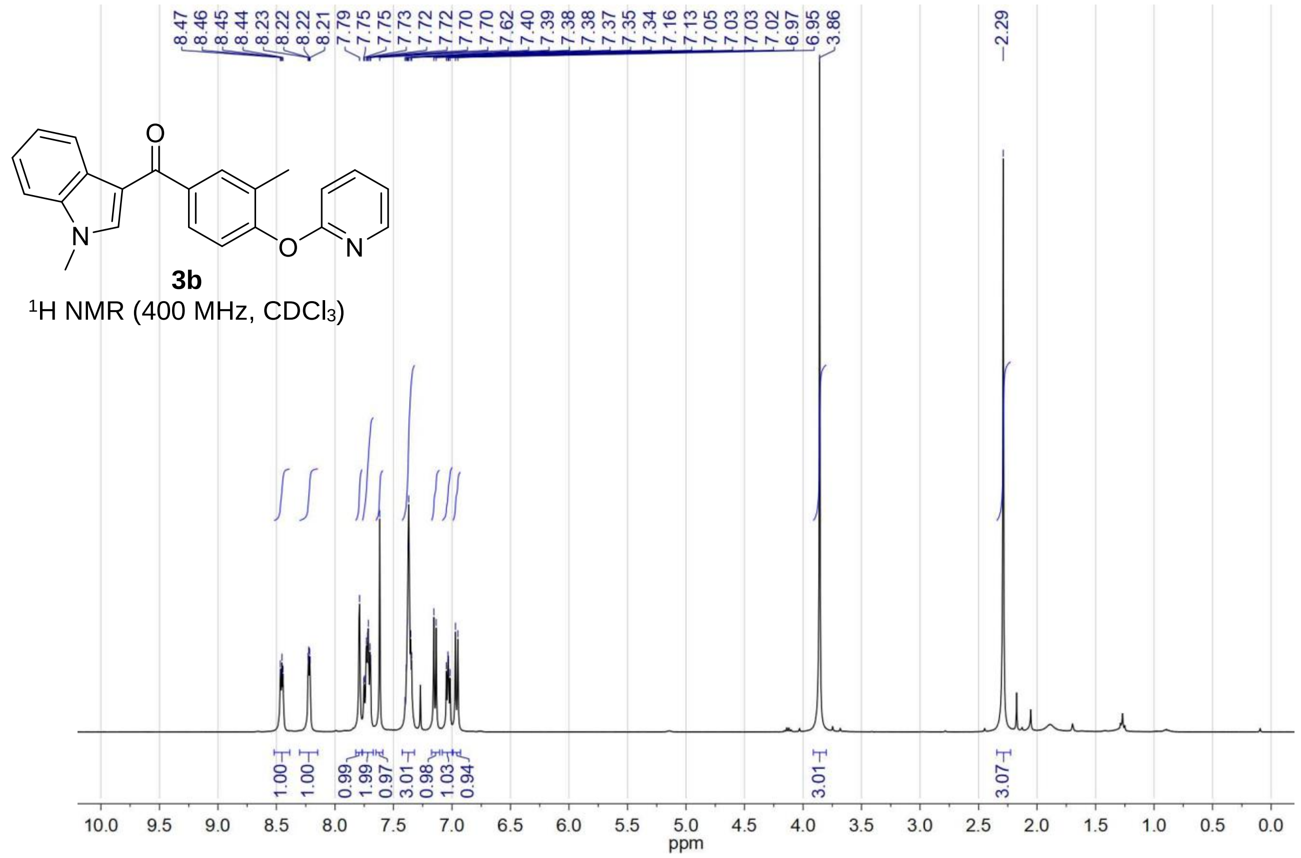

3b

^{1}H NMR (400 MHz, $CDCl_3$)

3b

^{13}C NMR (101 MHz, $CDCl_3$)

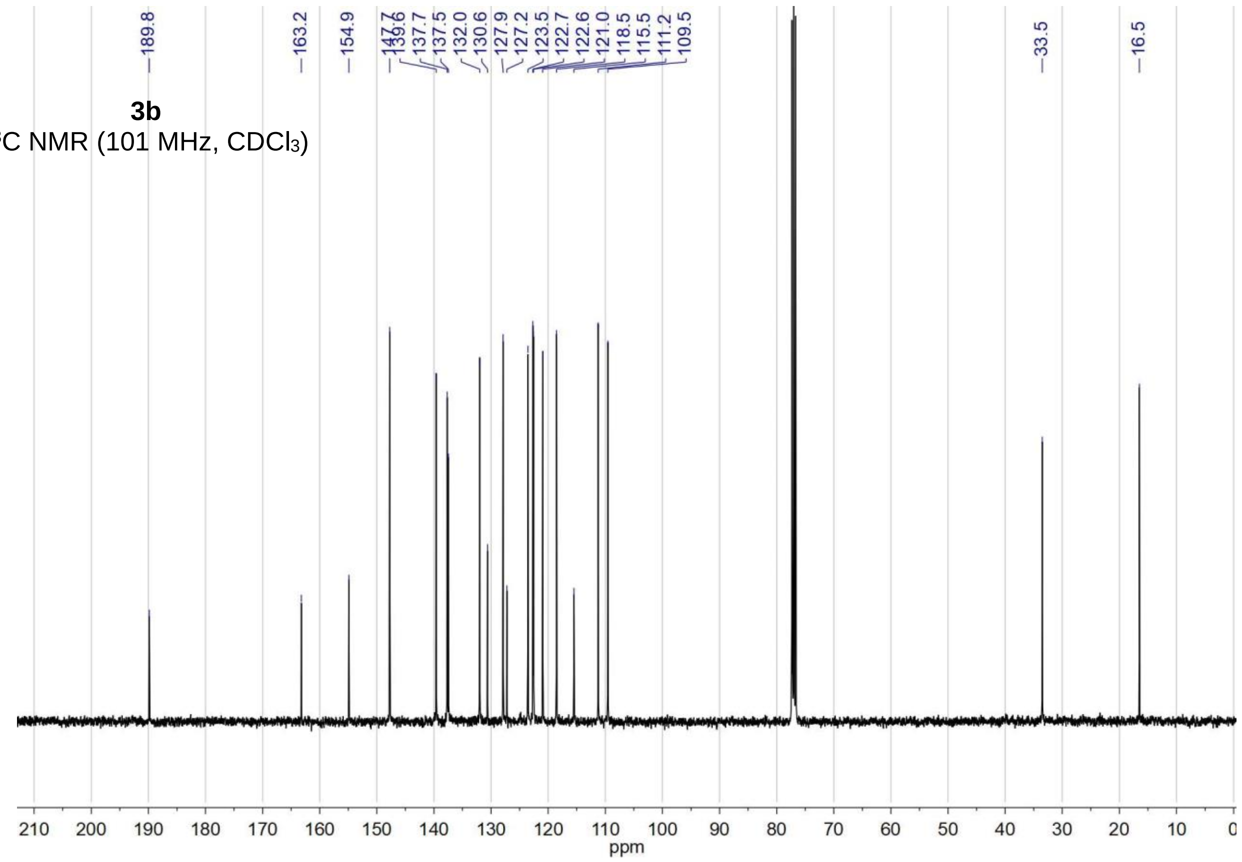

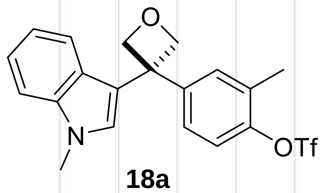

18a

^{1}H NMR (400 MHz, CDCl$_3$)

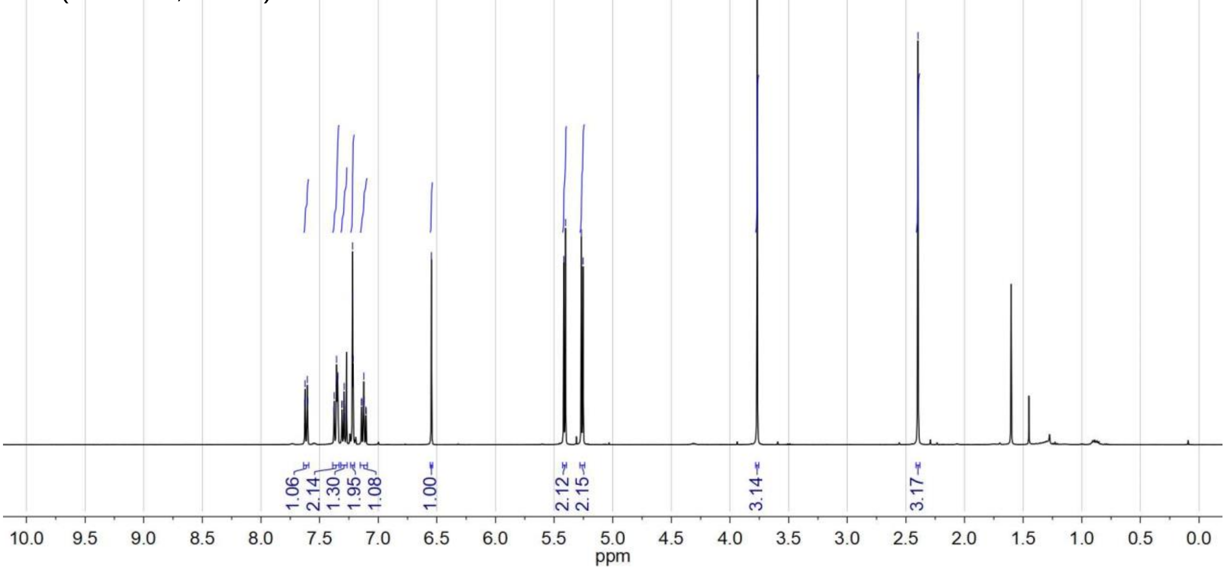

18a

^{13}C NMR (101 MHz, CDCl$_3$)

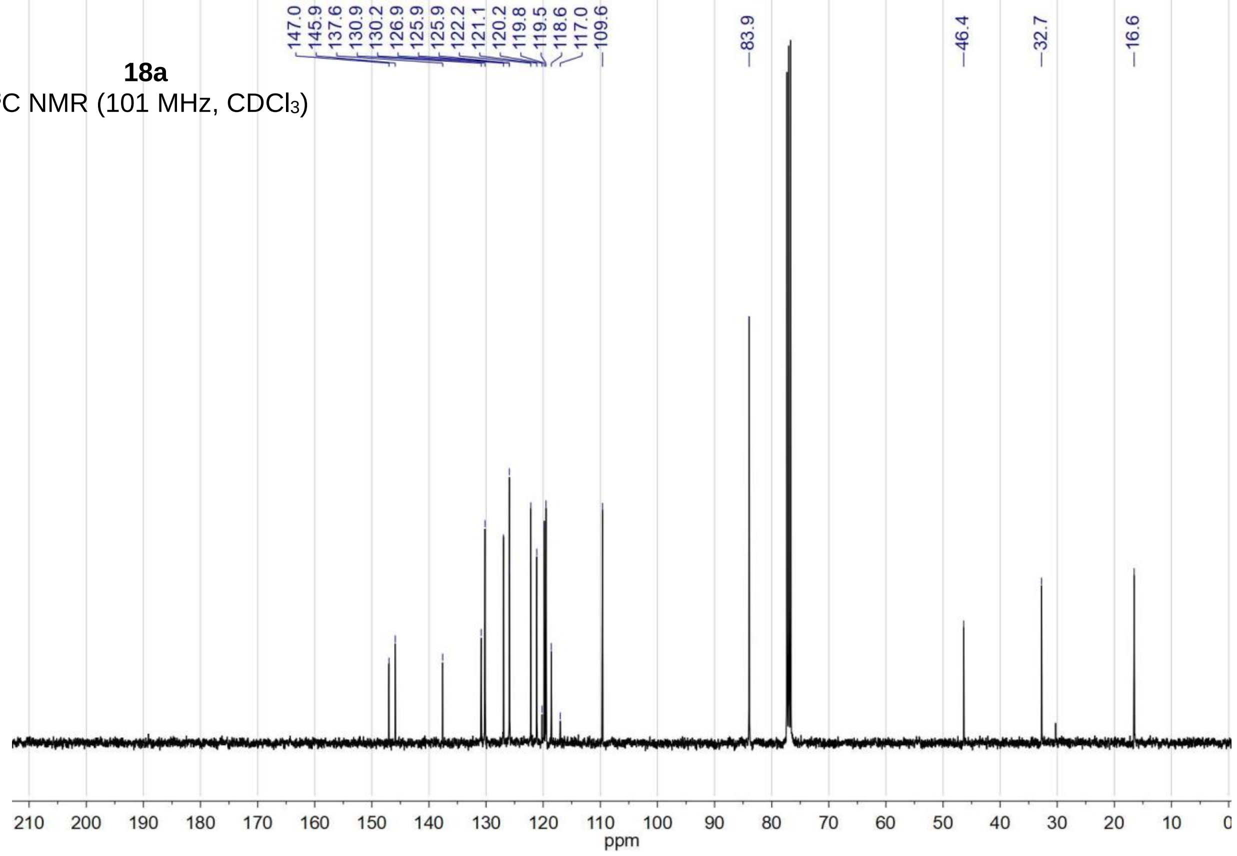

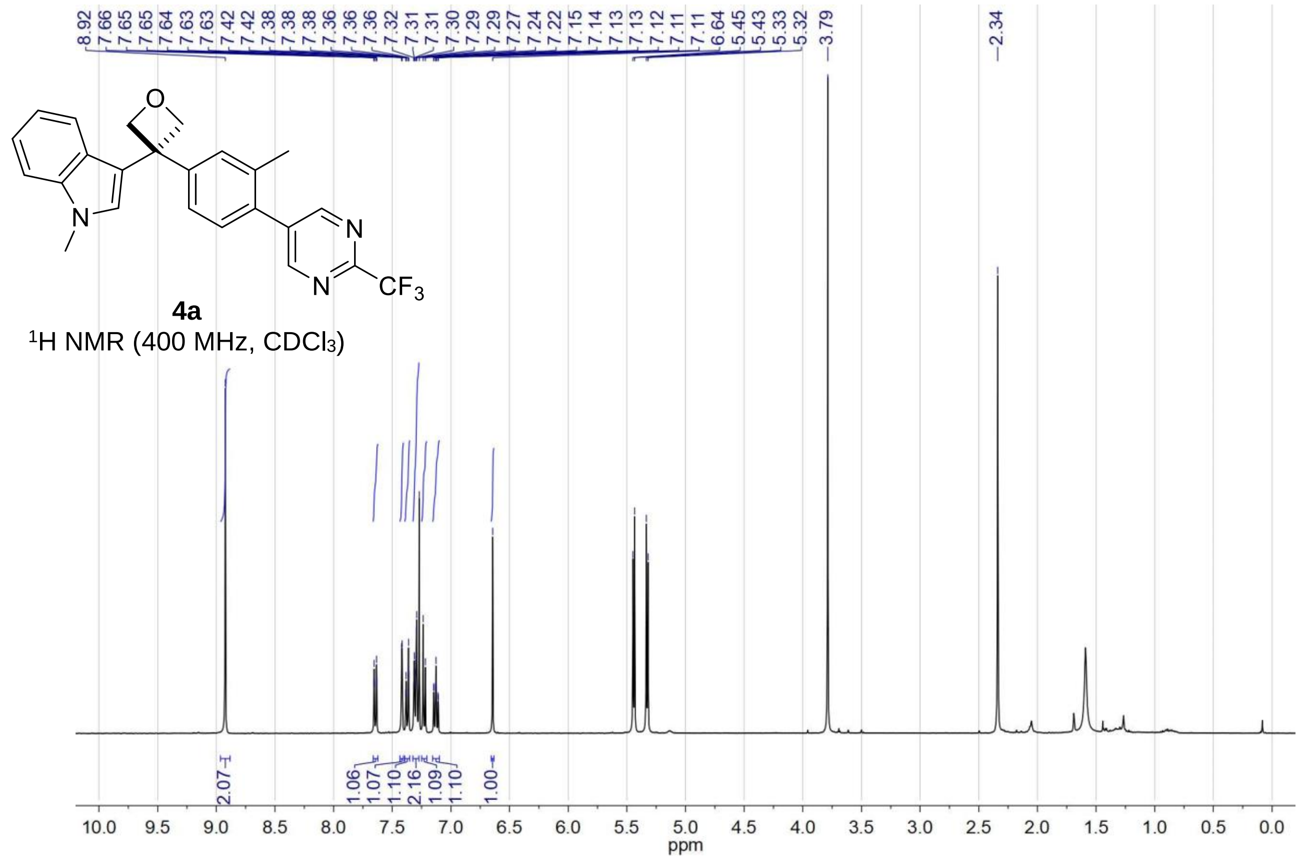
8.92
7.66
7.65
7.65
7.64
7.63
7.63
7.42
7.42
7.38
7.38
7.38
7.36
7.36
7.36
7.32
7.31
7.31
7.30
7.29
7.29
7.27
7.24
7.22
7.15
7.14
7.13
7.13
7.12
7.11
7.11
6.64
5.45
5.43
5.33
5.32
3.79
2.34
O
N
N
N
CF3
4a
1H NMR (400 MHz, CDCl3)
2.07
1.06
1.07
1.10
2.16
1.09
1.10
1.00
ppm

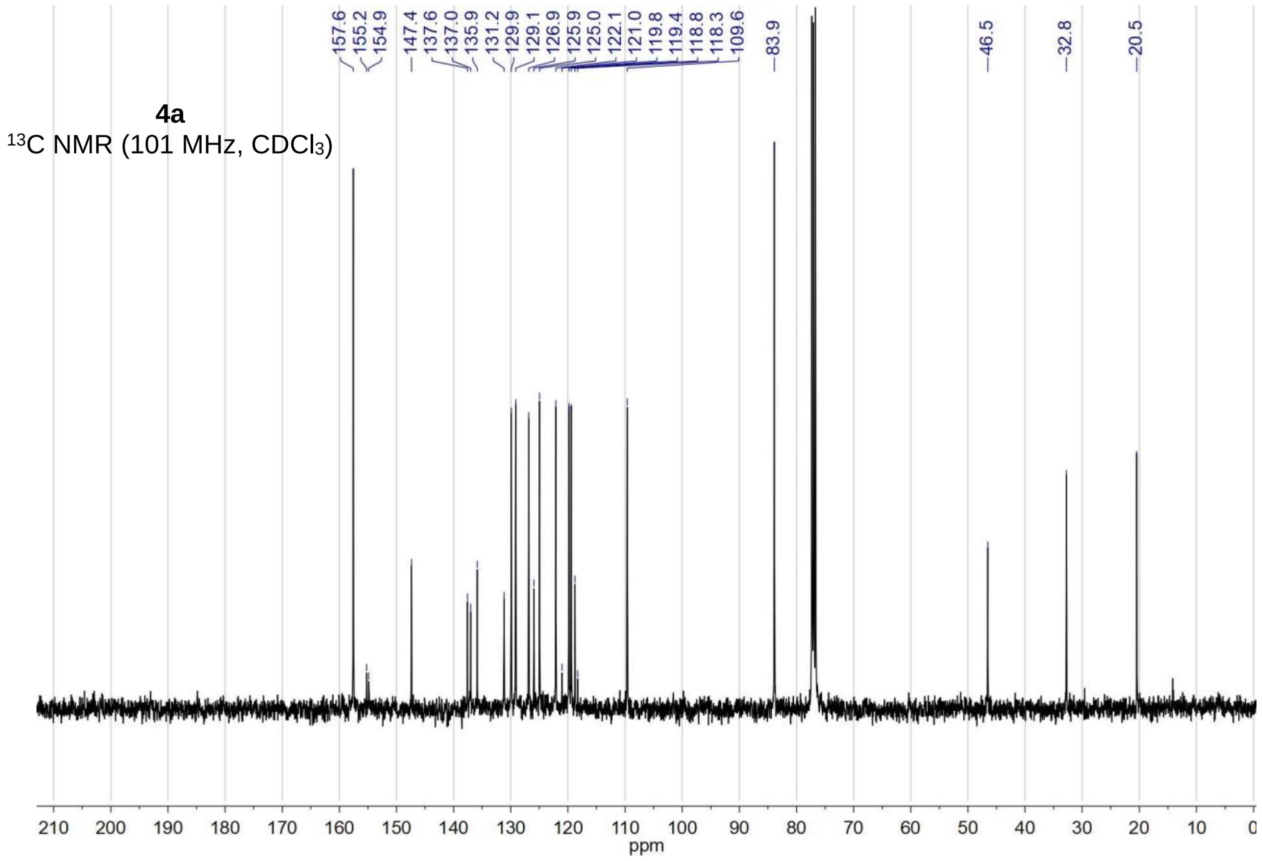
157.6
155.2
154.9
147.4
137.6
137.0
135.9
131.2
129.9
129.1
126.9
125.9
125.0
122.1
121.0
119.8
119.4
118.8
118.3
109.6
83.9
46.5
32.8
20.5
4a
13C NMR (101 MHz, CDCl3)
ppm

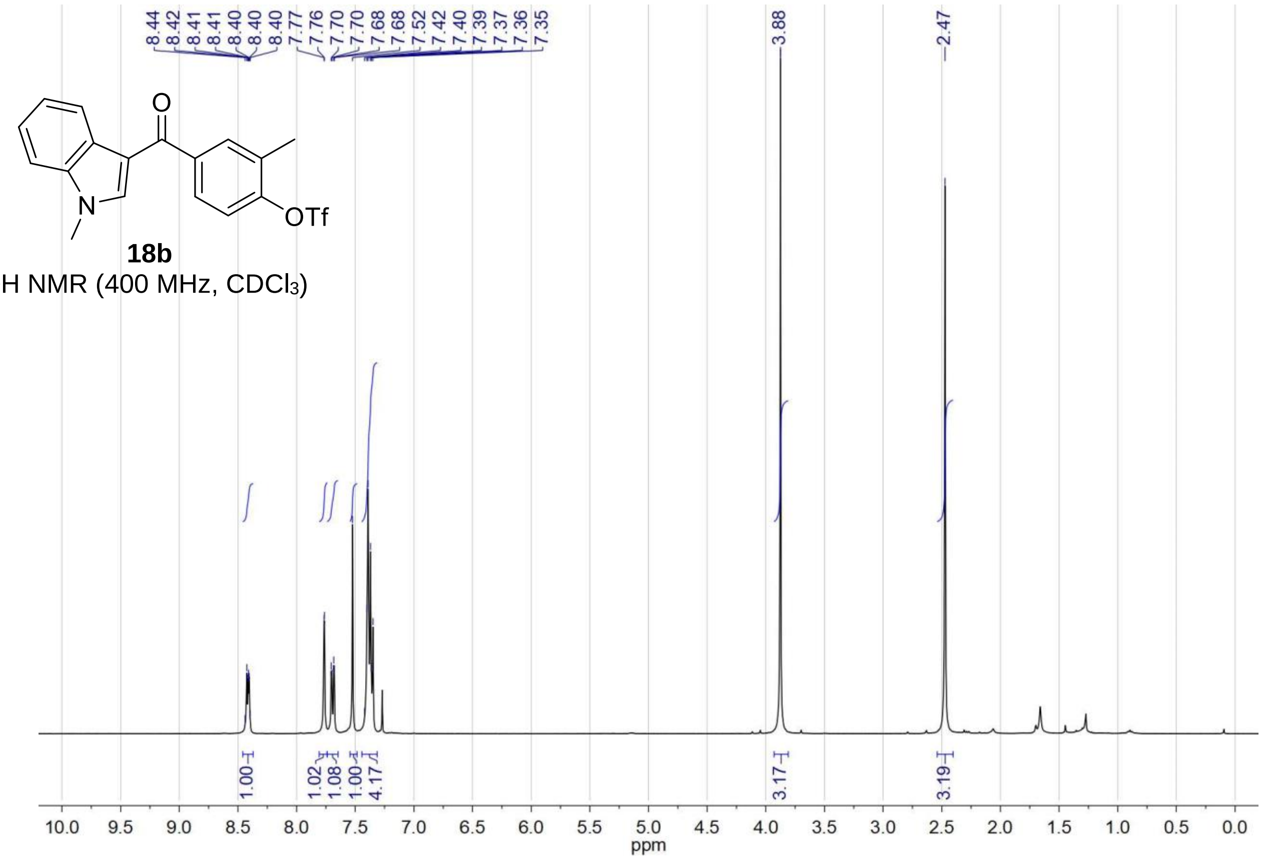

18b

^{1}H NMR (400 MHz, $CDCl_3$)

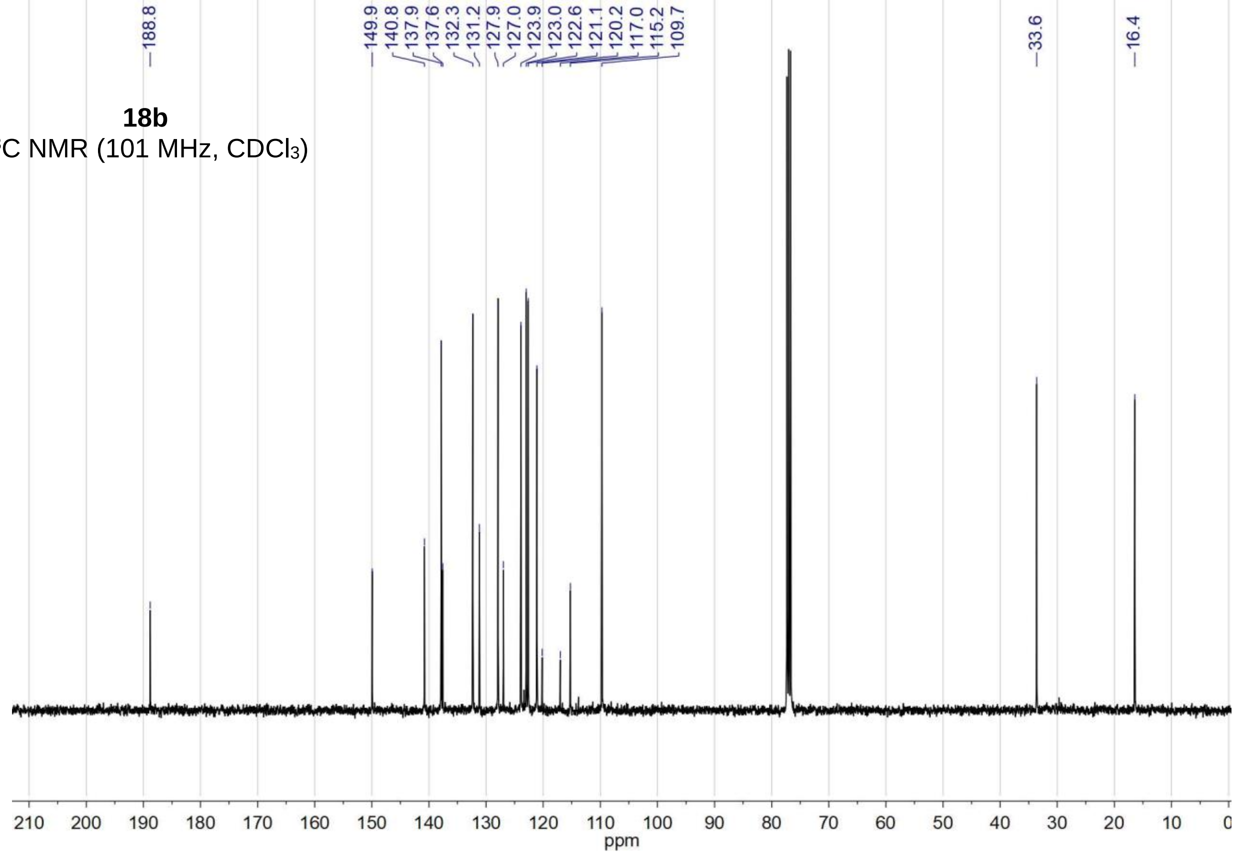

18b

^{13}C NMR (101 MHz, $CDCl_3$)

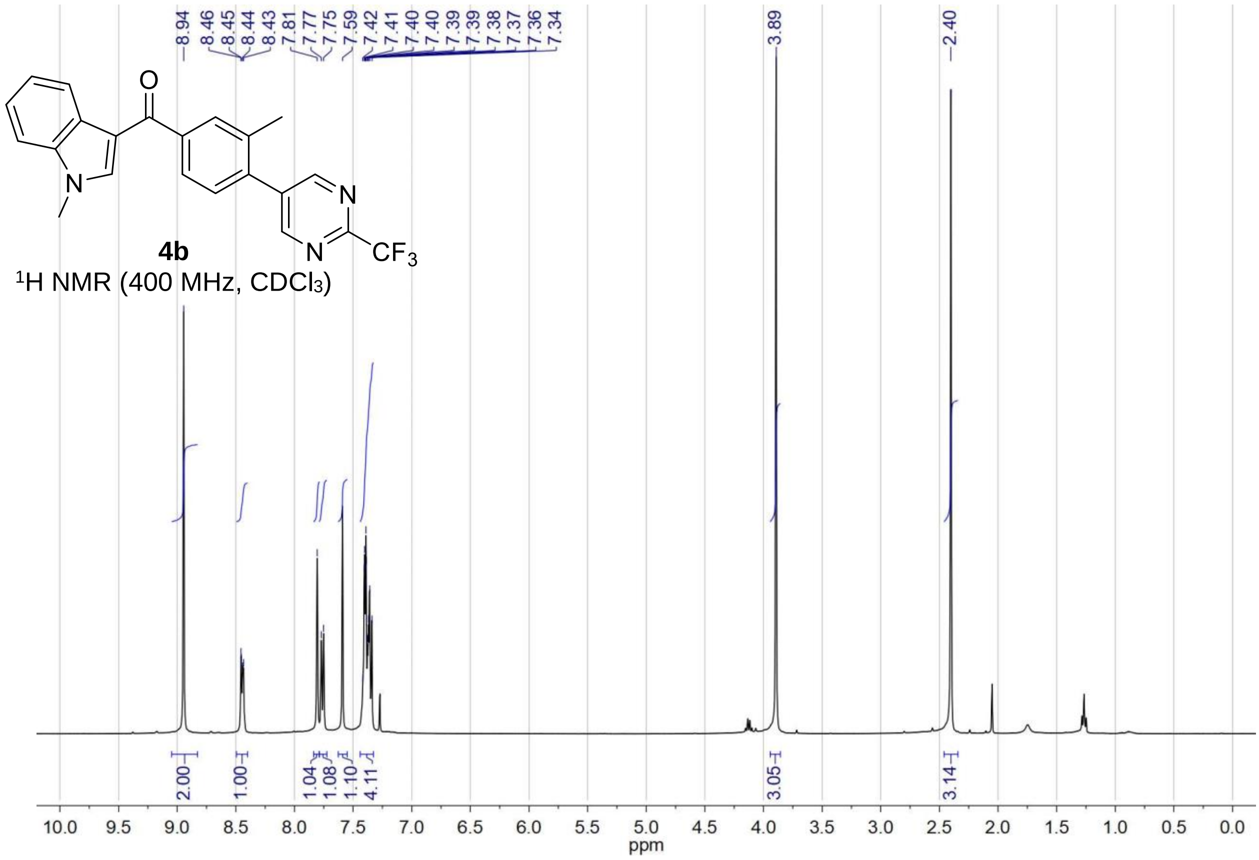
8.94
8.46
8.45
8.44
8.43
7.81
7.77
7.75
7.59
7.42
7.41
7.40
7.40
7.39
7.39
7.38
7.37
7.36
7.34
3.89
2.40
O
N
N
N
CF3
4b
1H NMR (400 MHz, CDCl3)
2.00
1.00
1.04
1.08
1.10
4.11
3.05
3.14
ppm

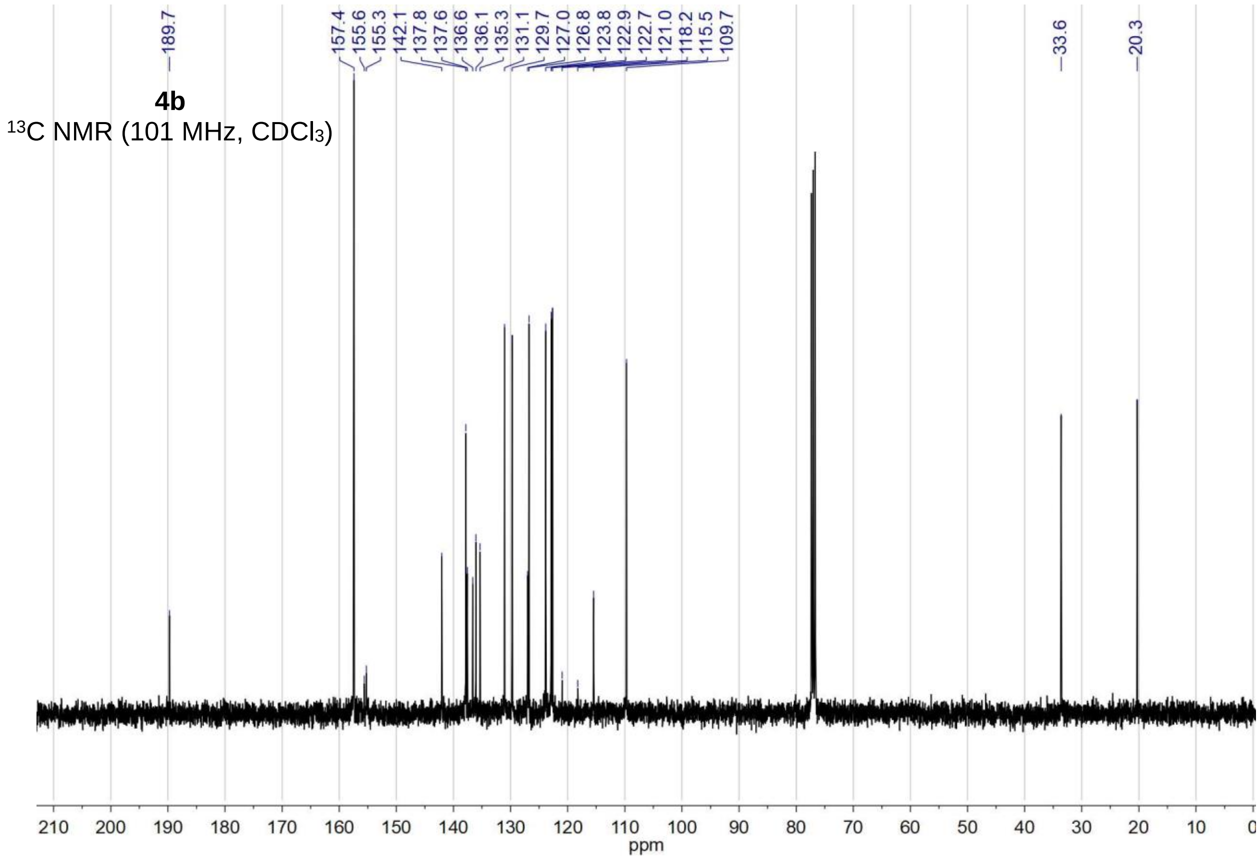
189.7
157.4
155.6
155.3
142.1
137.8
137.6
136.6
136.1
135.3
131.1
129.7
127.0
126.8
123.8
122.9
122.7
121.0
118.2
115.5
109.7
33.6
20.3
4b
13C NMR (101 MHz, CDCl3)
ppm

S3

^{1}H NMR (400 MHz, $CDCl_3$)

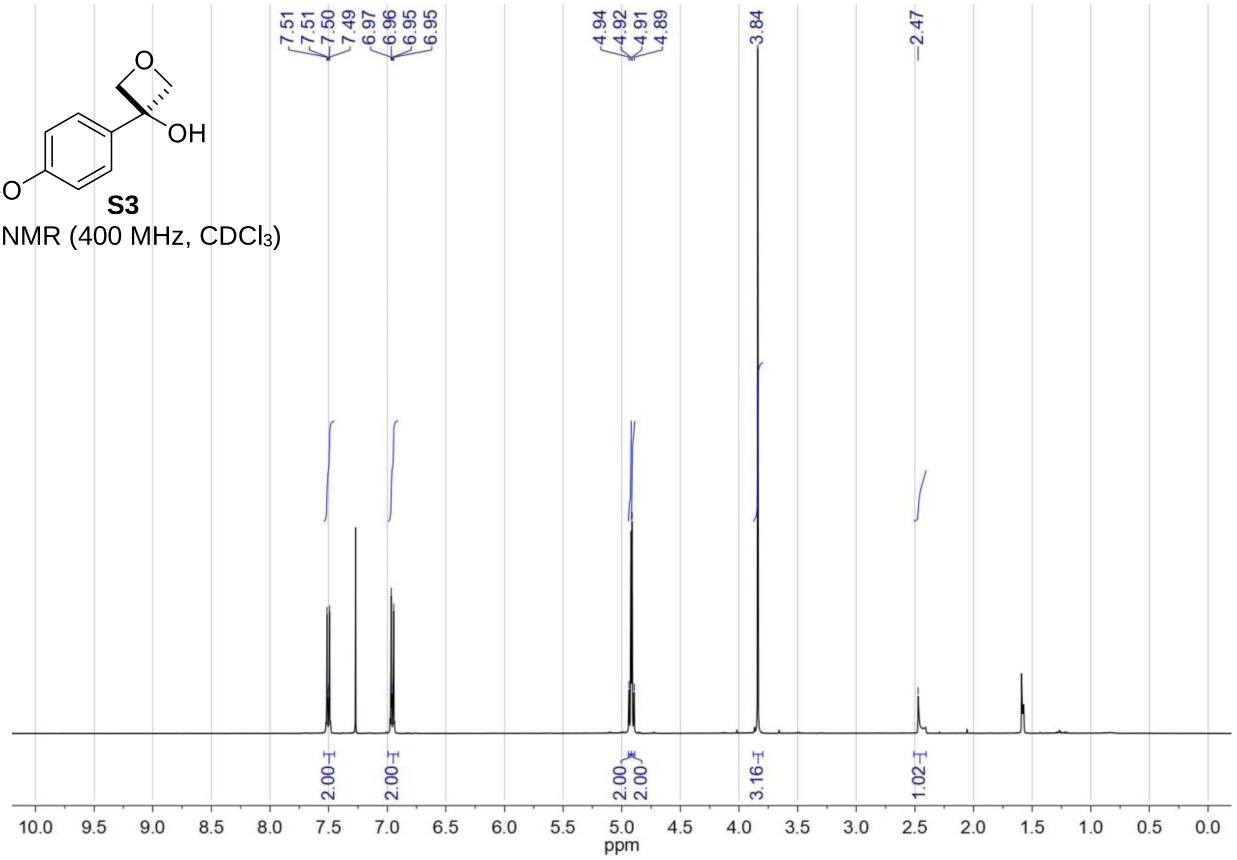

S3

^{13}C NMR (101 MHz, $CDCl_3$)

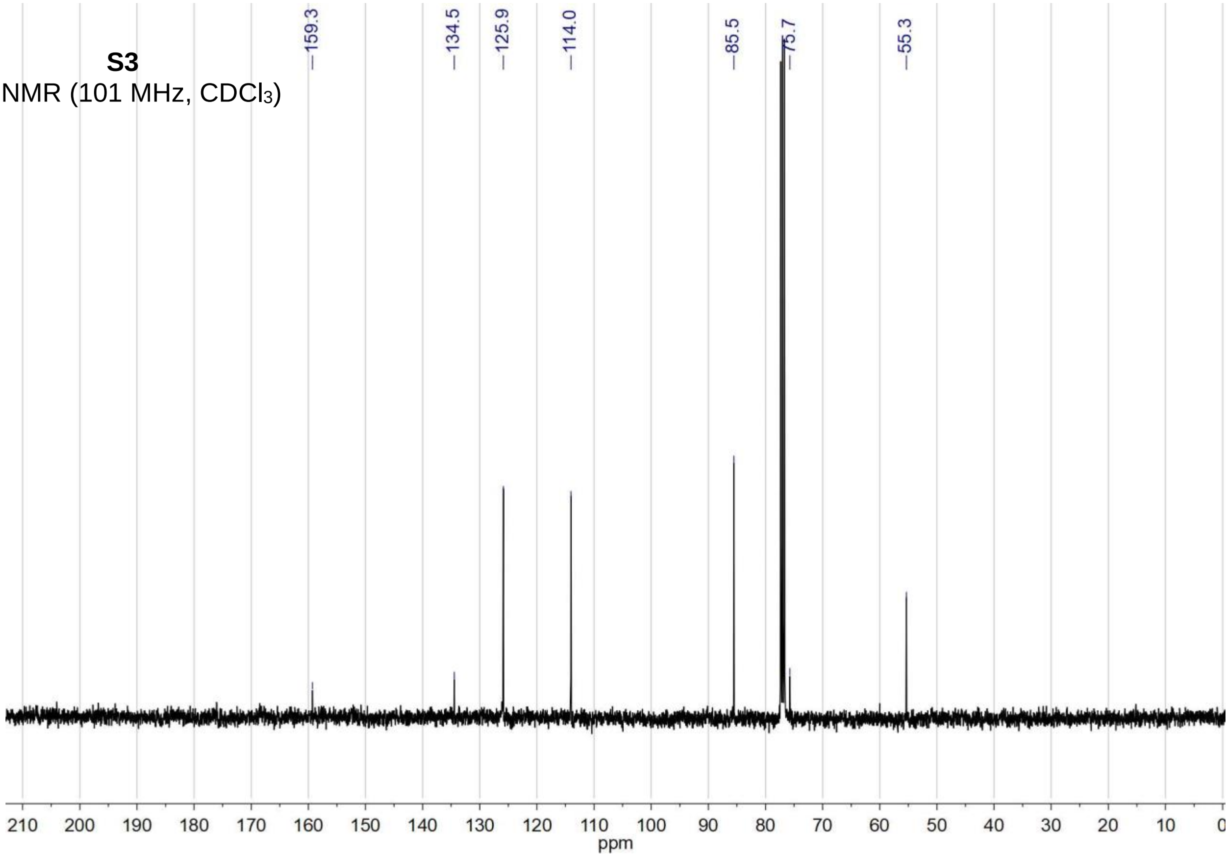

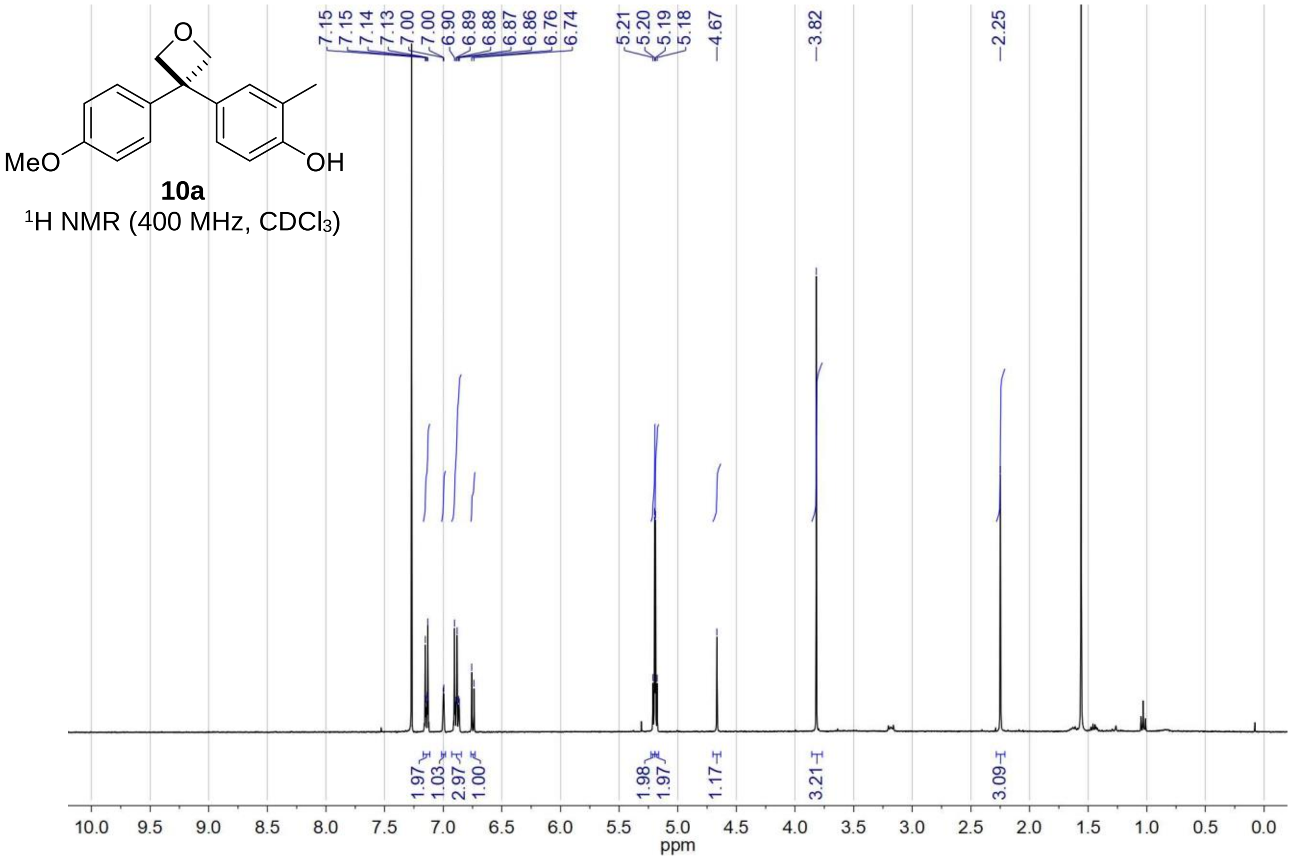

10a

^{1}H NMR (400 MHz, $CDCl_3$)

10a

^{13}C NMR (101 MHz, $CDCl_3$)

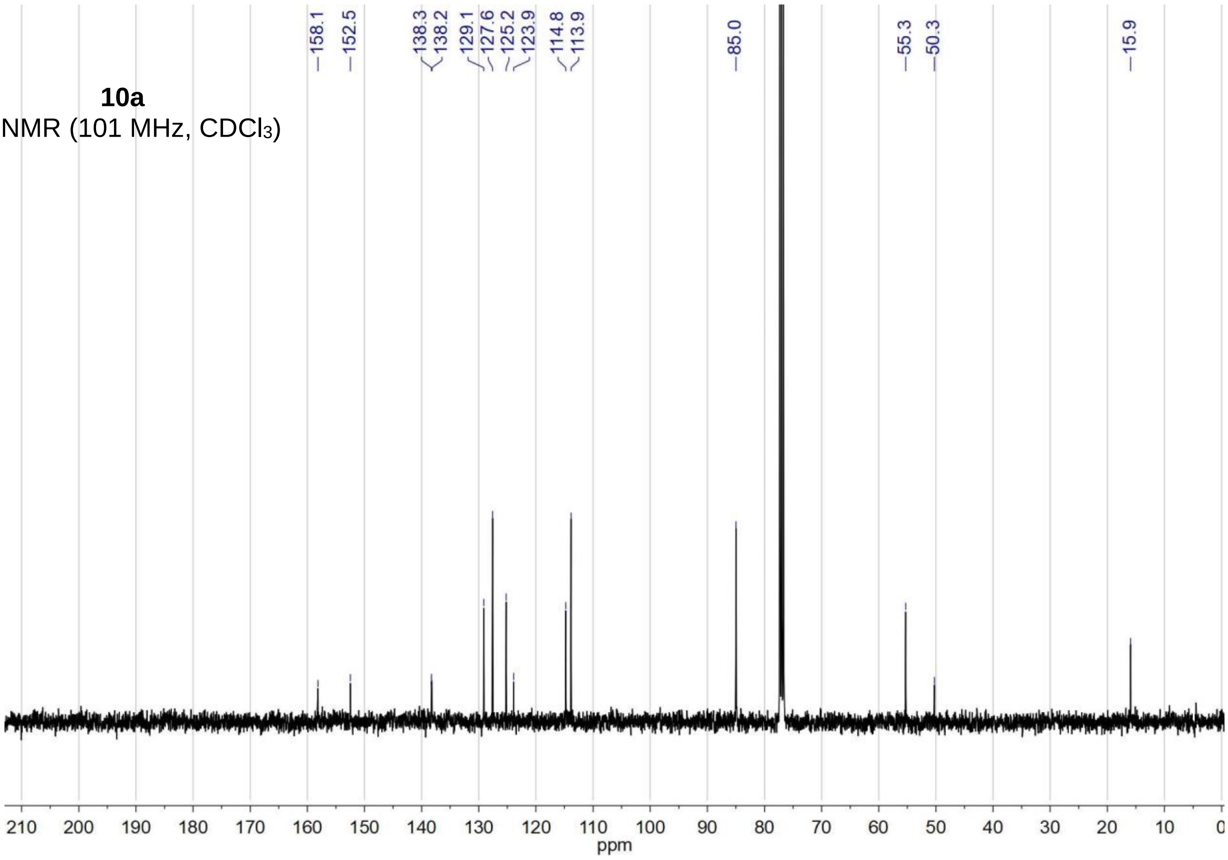

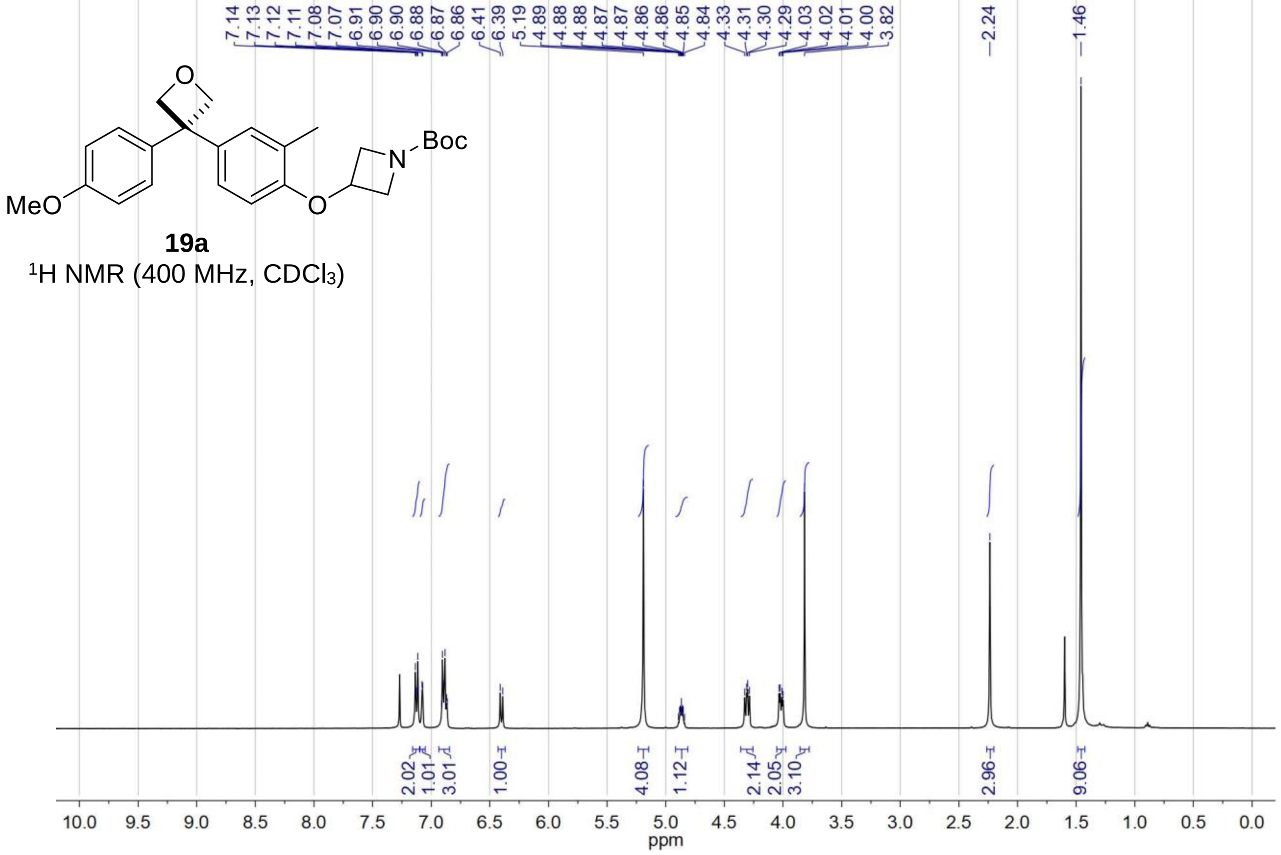

19a

^{1}H NMR (400 MHz, $CDCl_3$)

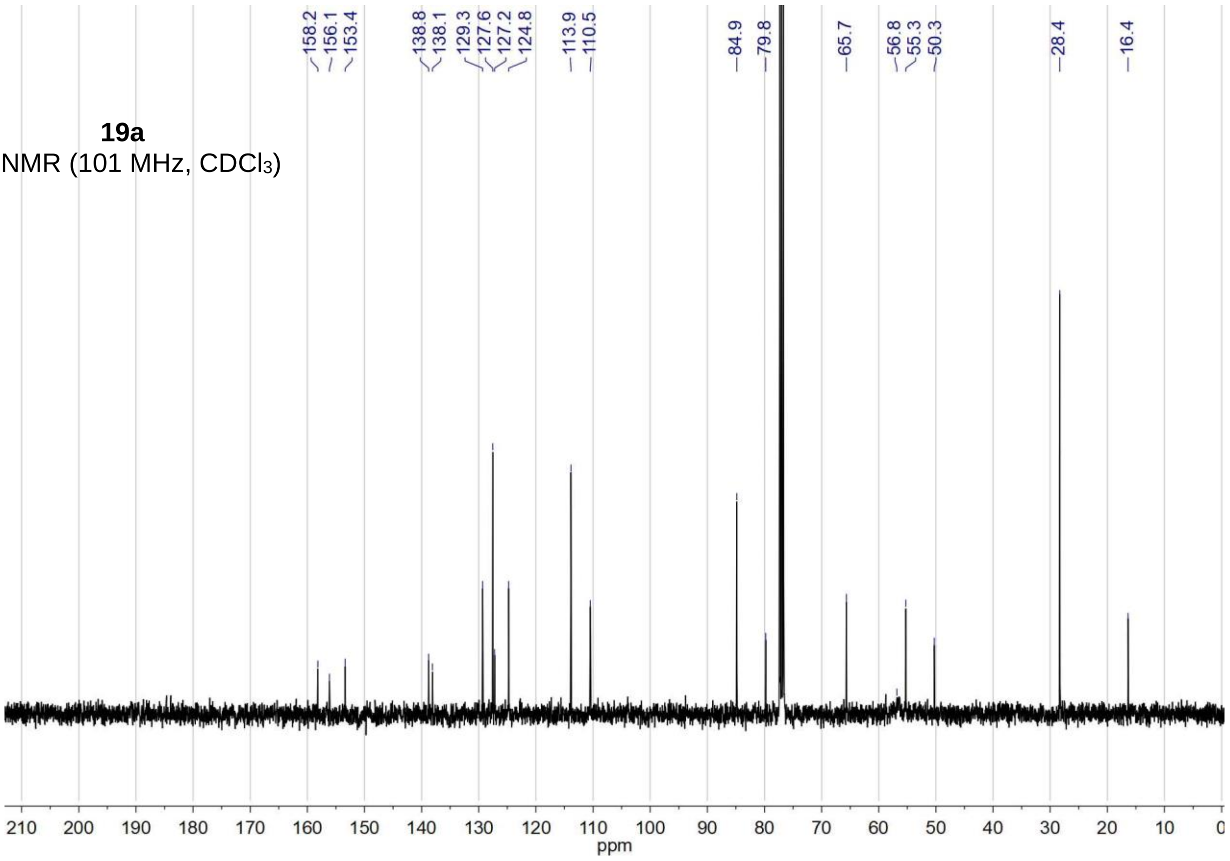

19a

^{13}C NMR (101 MHz, $CDCl_3$)

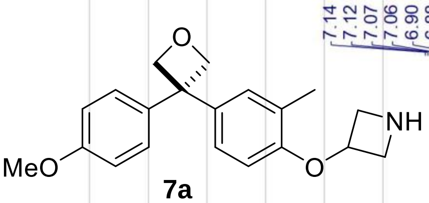

7a

^{1}H NMR (400 MHz, $CDCl_3$)

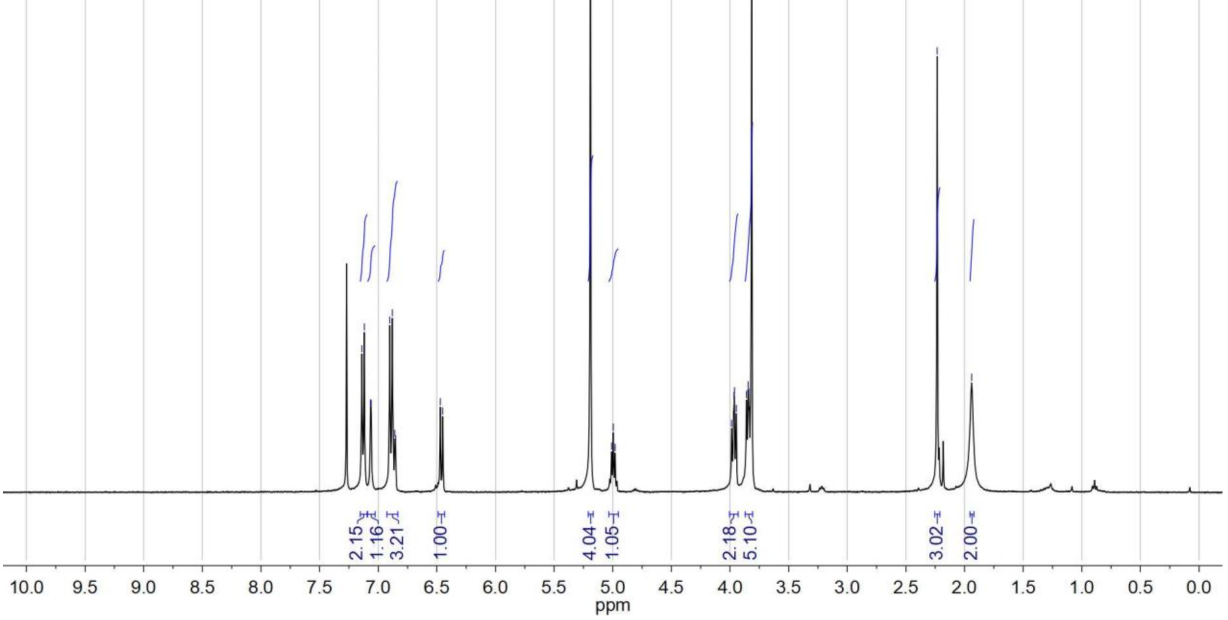

7a

^{13}C NMR (101 MHz, $CDCl_3$)

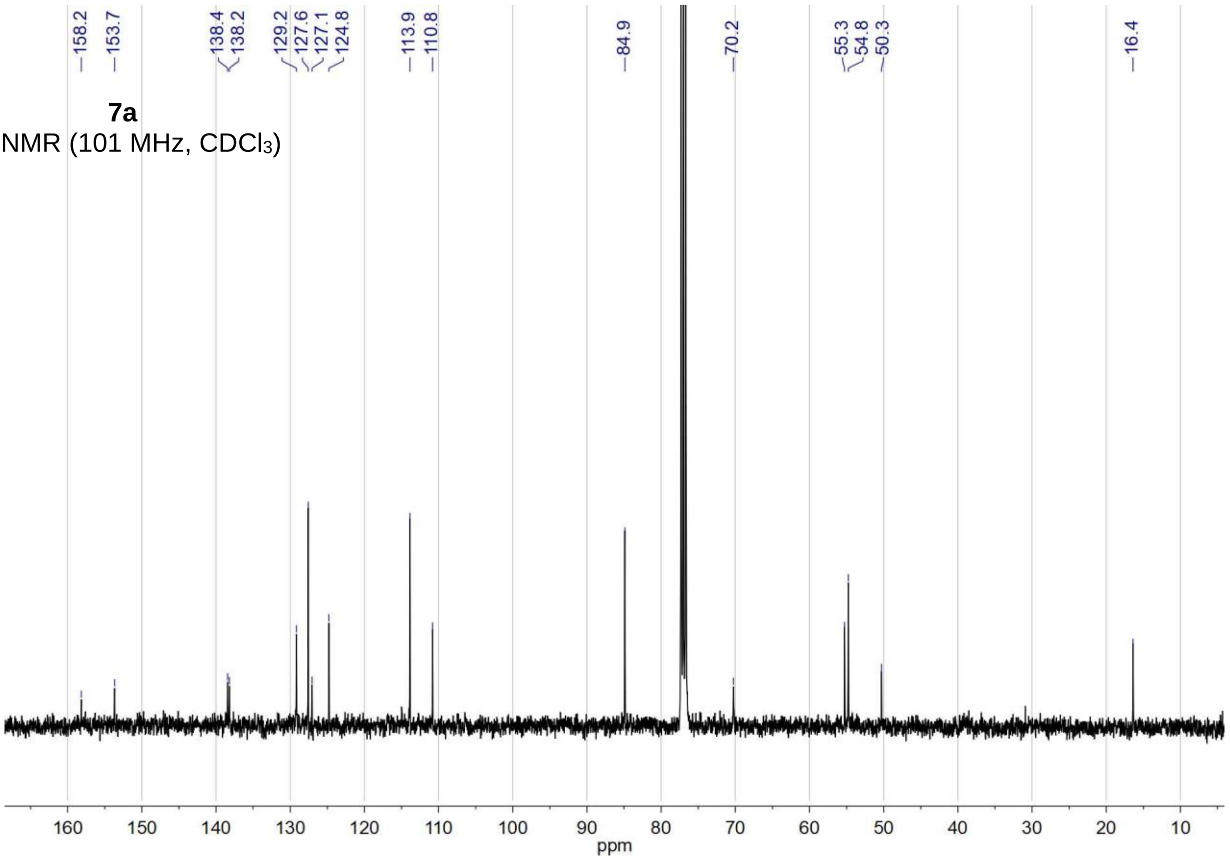

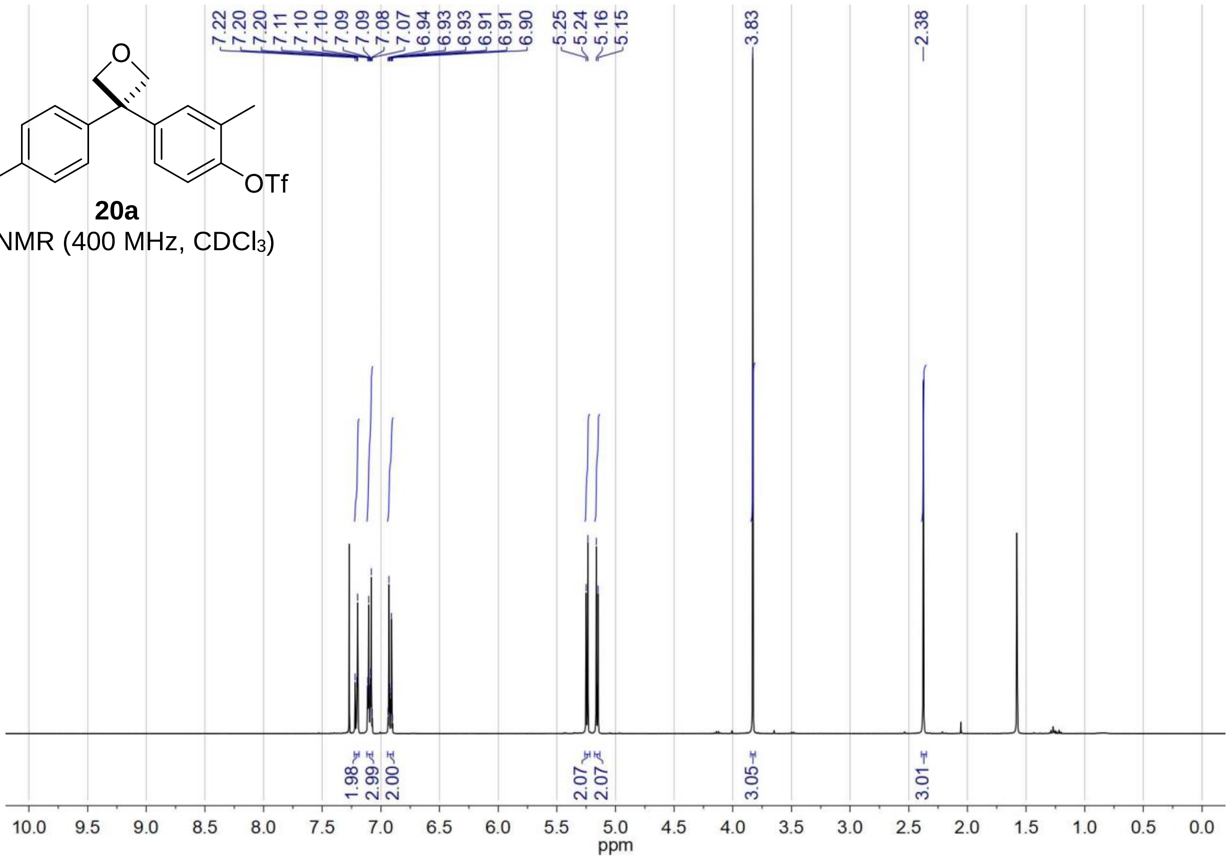

20a

^{1}H NMR (400 MHz, $CDCl_3$)

20a

^{13}C NMR (101 MHz, $CDCl_3$)

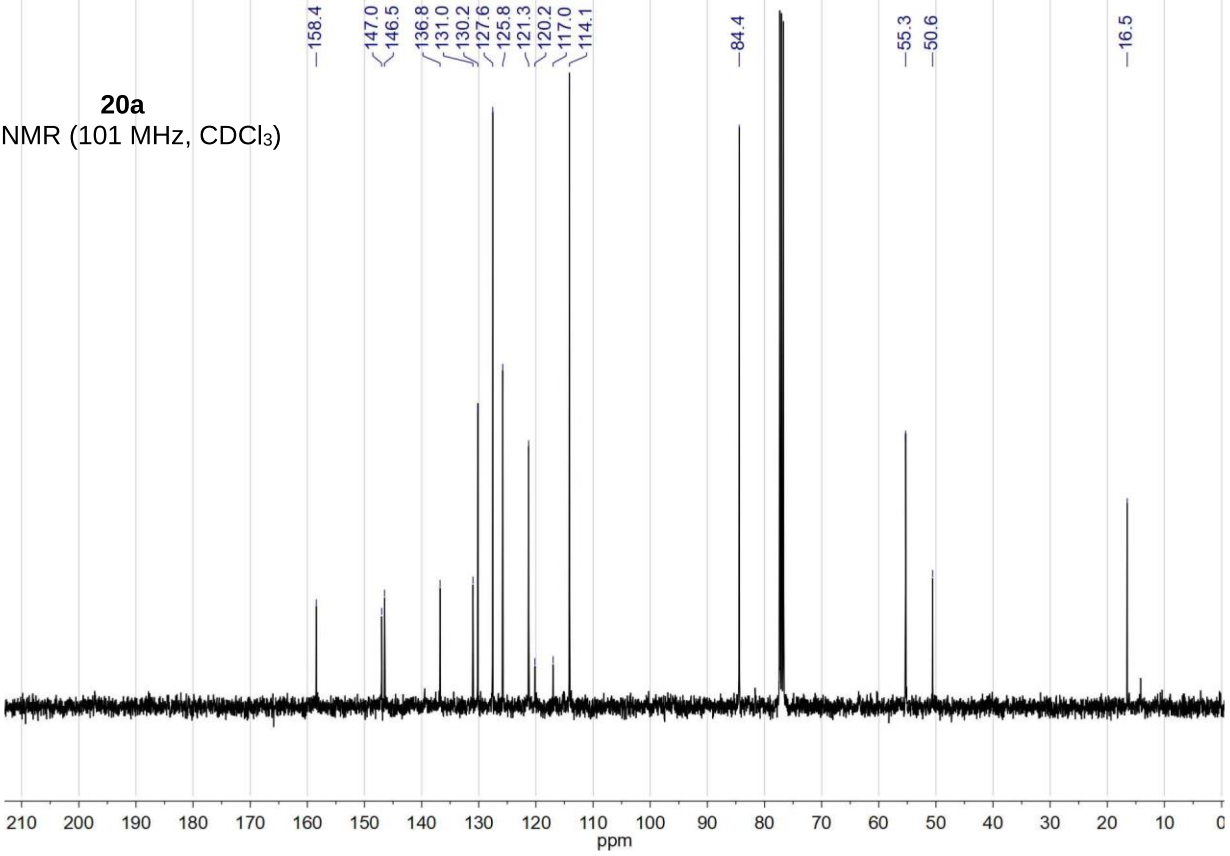

8a

^{1}H NMR (400 MHz, $CDCl_3$)

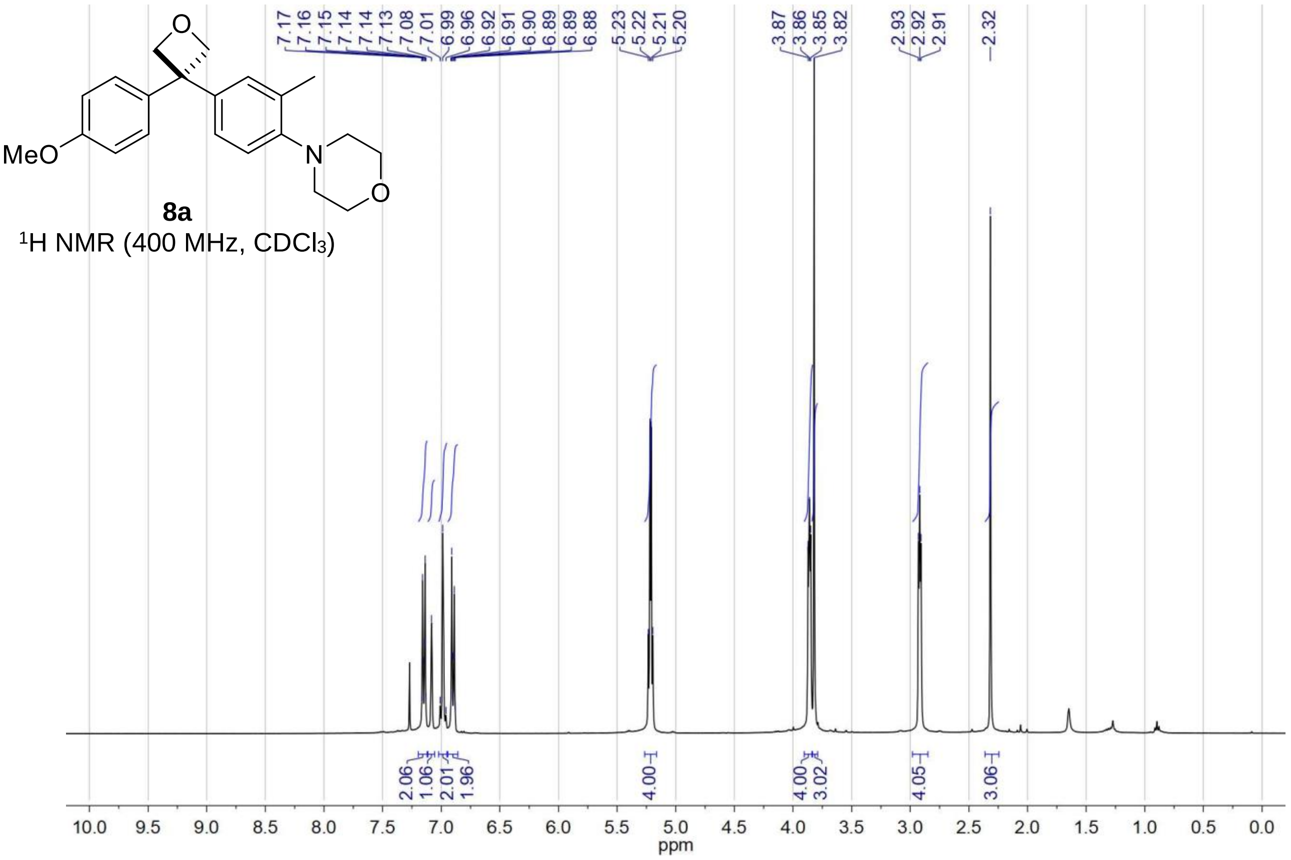

8a

^{13}C NMR (101 MHz, $CDCl_3$)

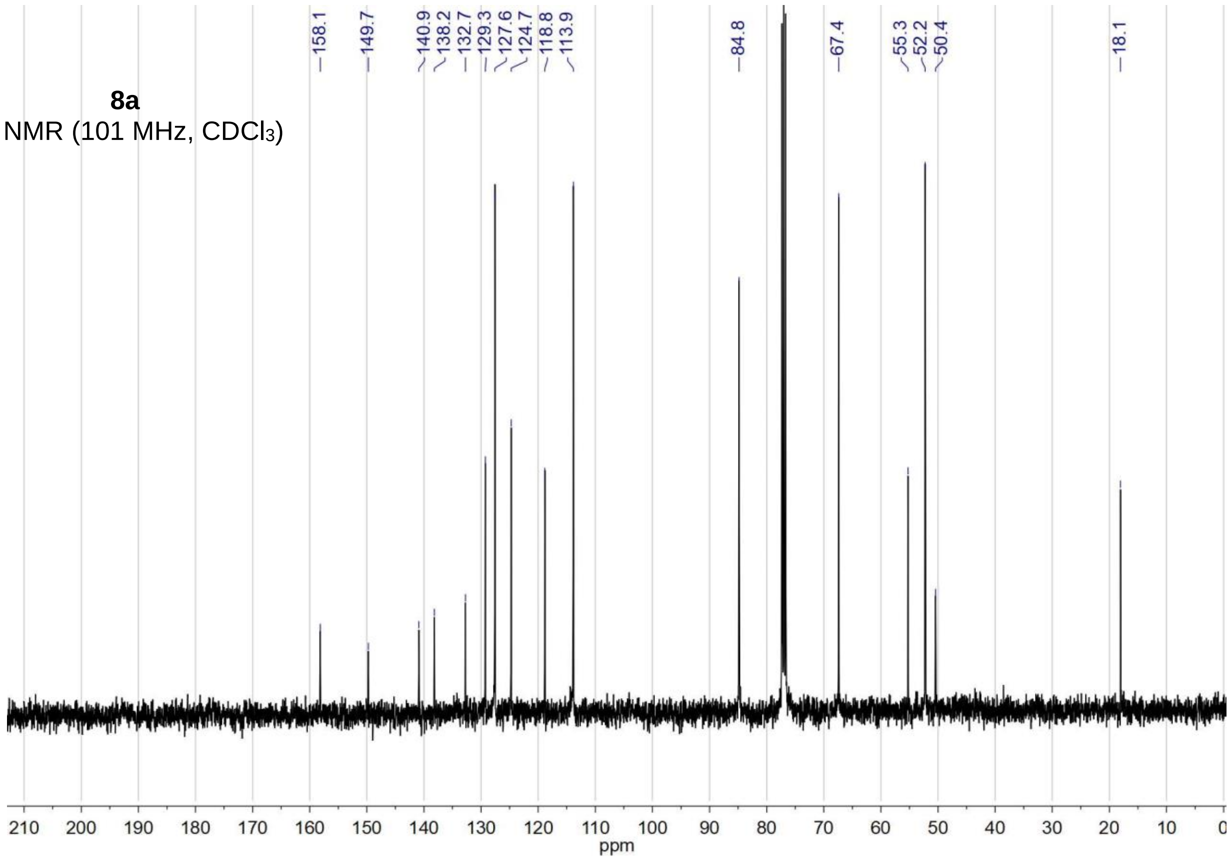

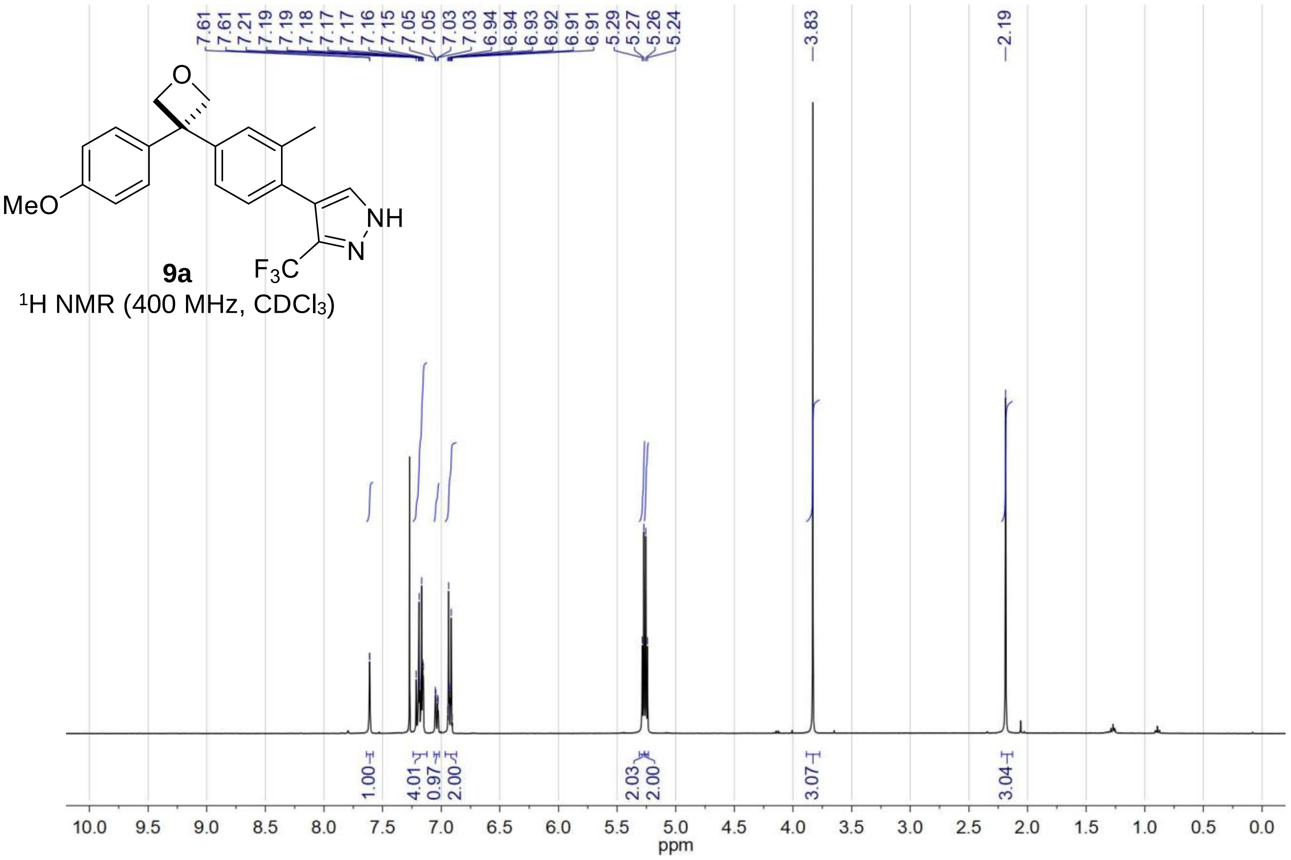
7.61 7.61 7.21 7.19 7.19 7.18 7.17 7.17 7.16 7.15 7.05 7.05 7.03 7.03 6.94 6.94 6.93 6.92 6.91 6.91 5.29 5.27 5.26 5.24
3.83
2.19
MeO
NH
N
F_3C
9a
^1H NMR (400 MHz, $CDCl_3$)
1.00 4.01 0.97 2.00 2.03 2.00 3.07 3.04
10.0 9.5 9.0 8.5 8.0 7.5 7.0 6.5 6.0 5.5 5.0 4.5 4.0 3.5 3.0 2.5 2.0 1.5 1.0 0.5 0.0
ppm

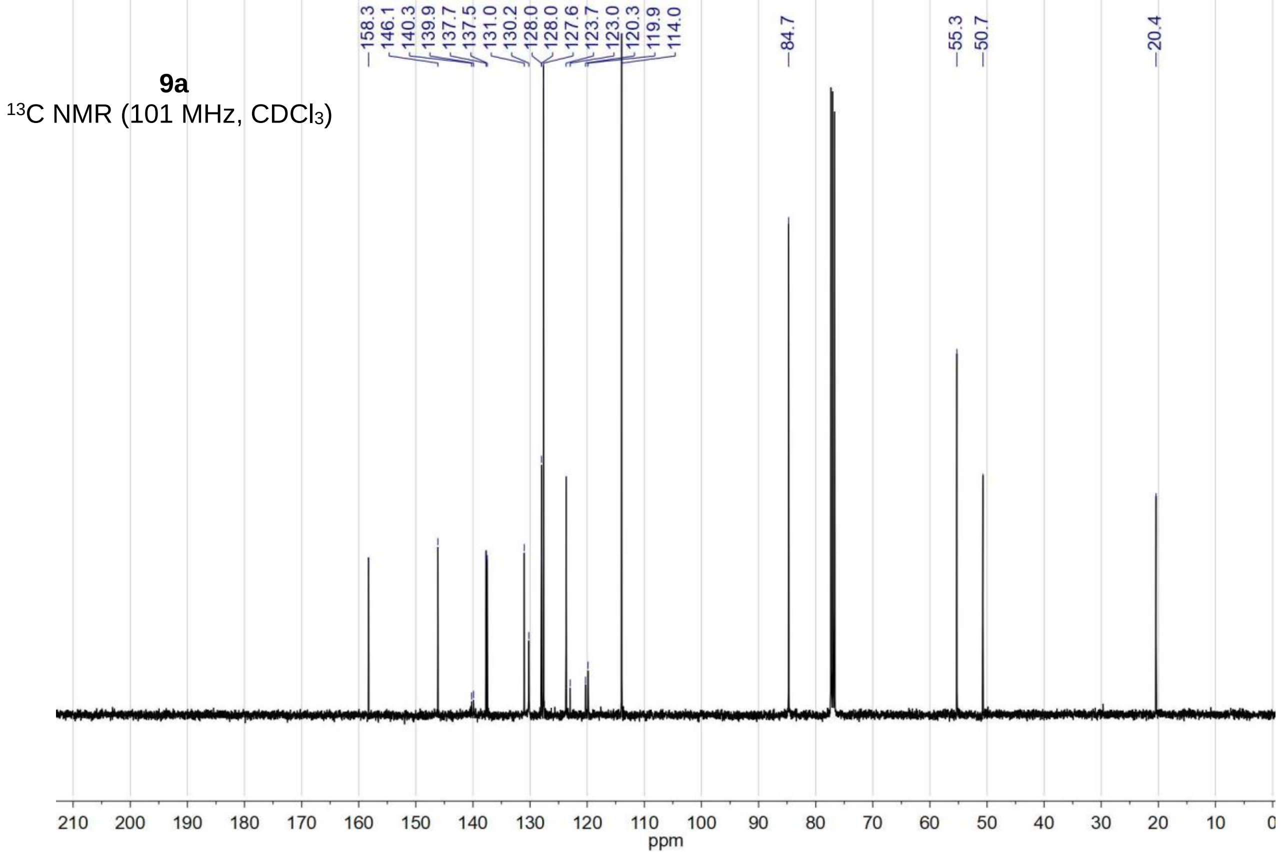
158.3 146.1 140.3 139.9 137.7 137.5 131.0 130.2 128.0 128.0 127.6 123.7 123.0 120.3 119.9 114.0
84.7
55.3 50.7
20.4
9a
^{13}C NMR (101 MHz, $CDCl_3$)
210 200 190 180 170 160 150 140 130 120 110 100 90 80 70 60 50 40 30 20 10 0
ppm

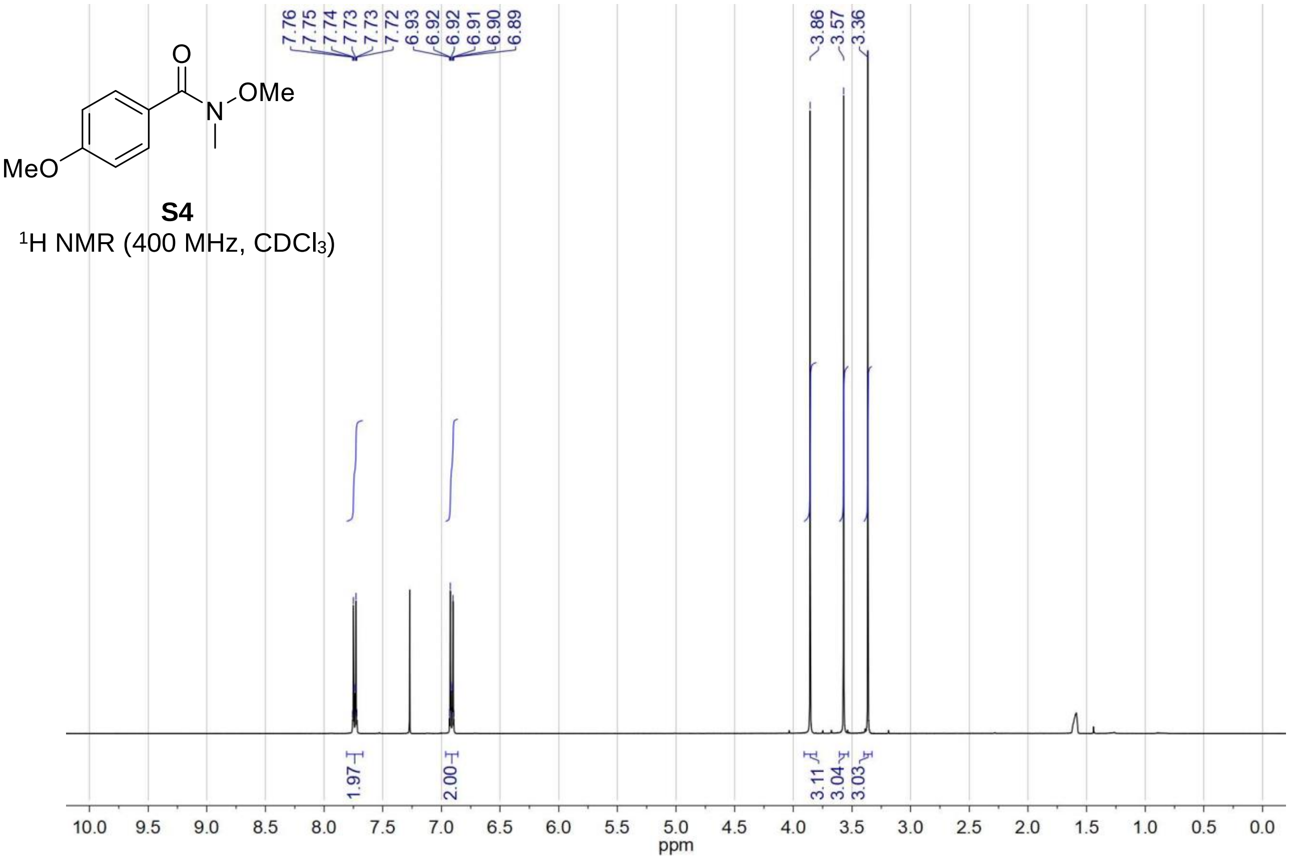

S4

^{1}H NMR (400 MHz, $CDCl_3$)

S4

^{13}C NMR (101 MHz, $CDCl_3$)

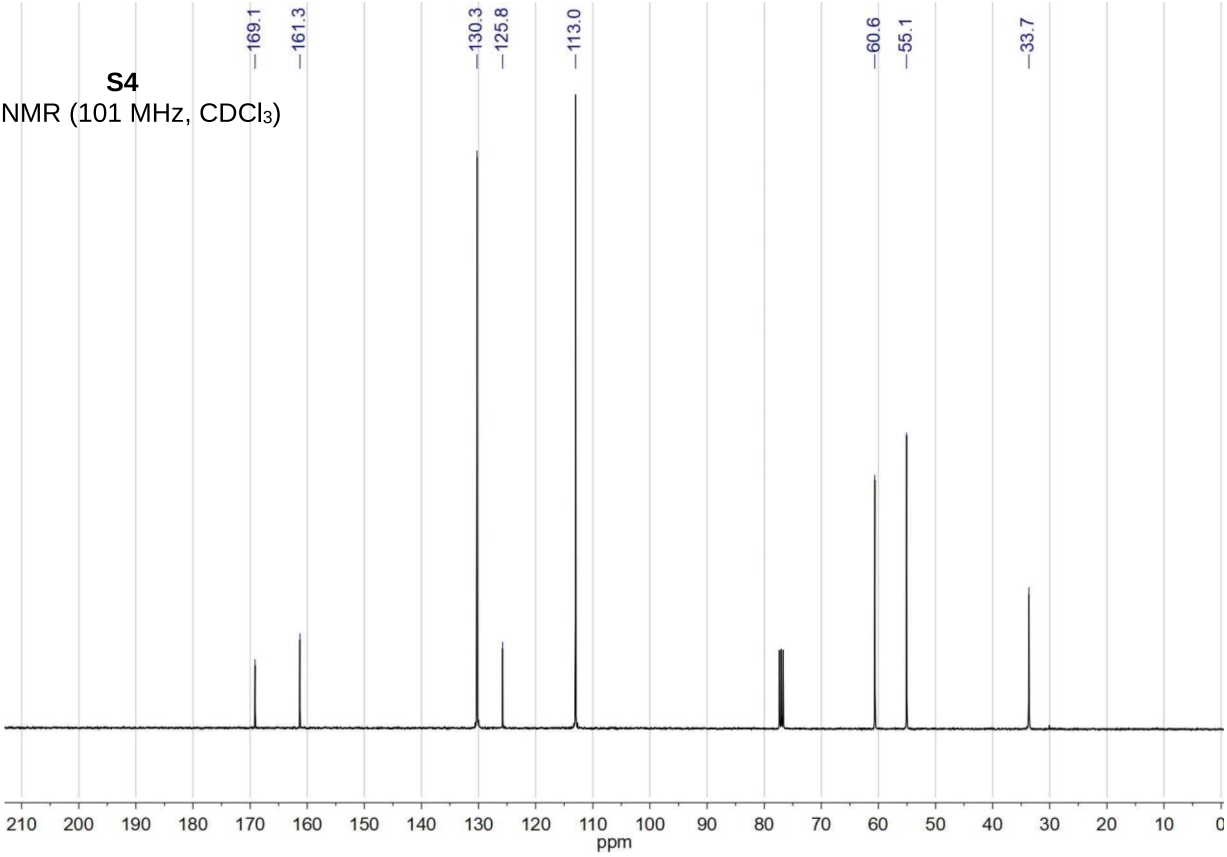

S5
^{1}H NMR (400 MHz, $CDCl_3$)

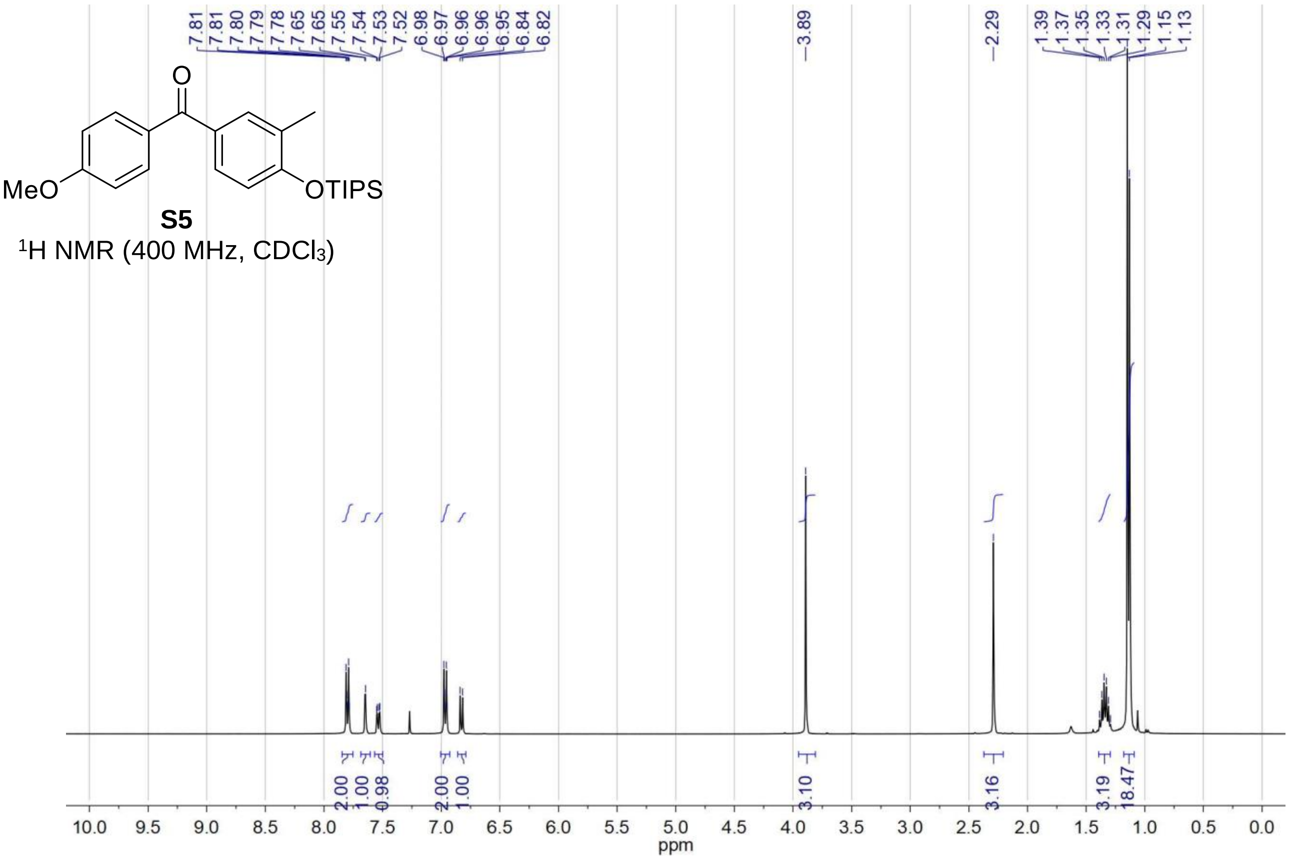

S5
^{13}C NMR (101 MHz, $CDCl_3$)

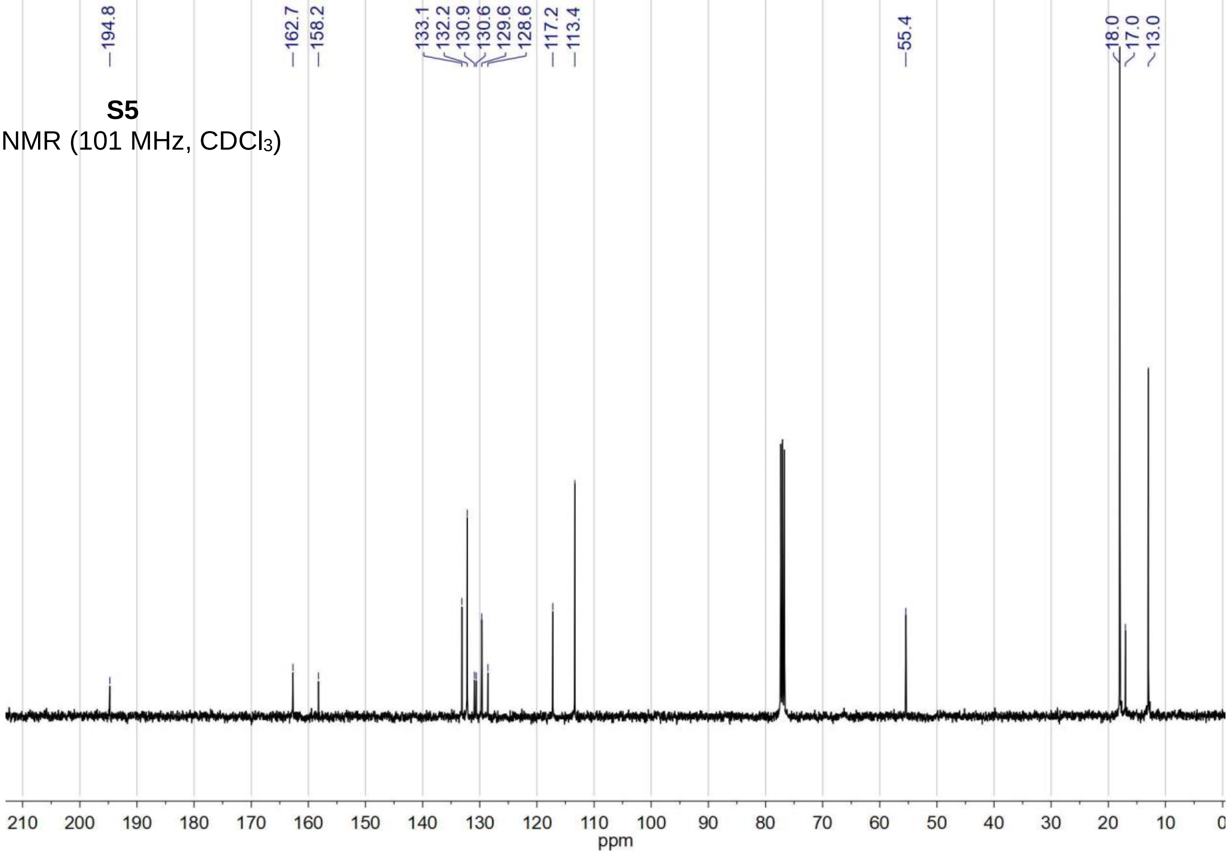

10b
^{1}H NMR (400 MHz, $(CD_3)_2SO$)

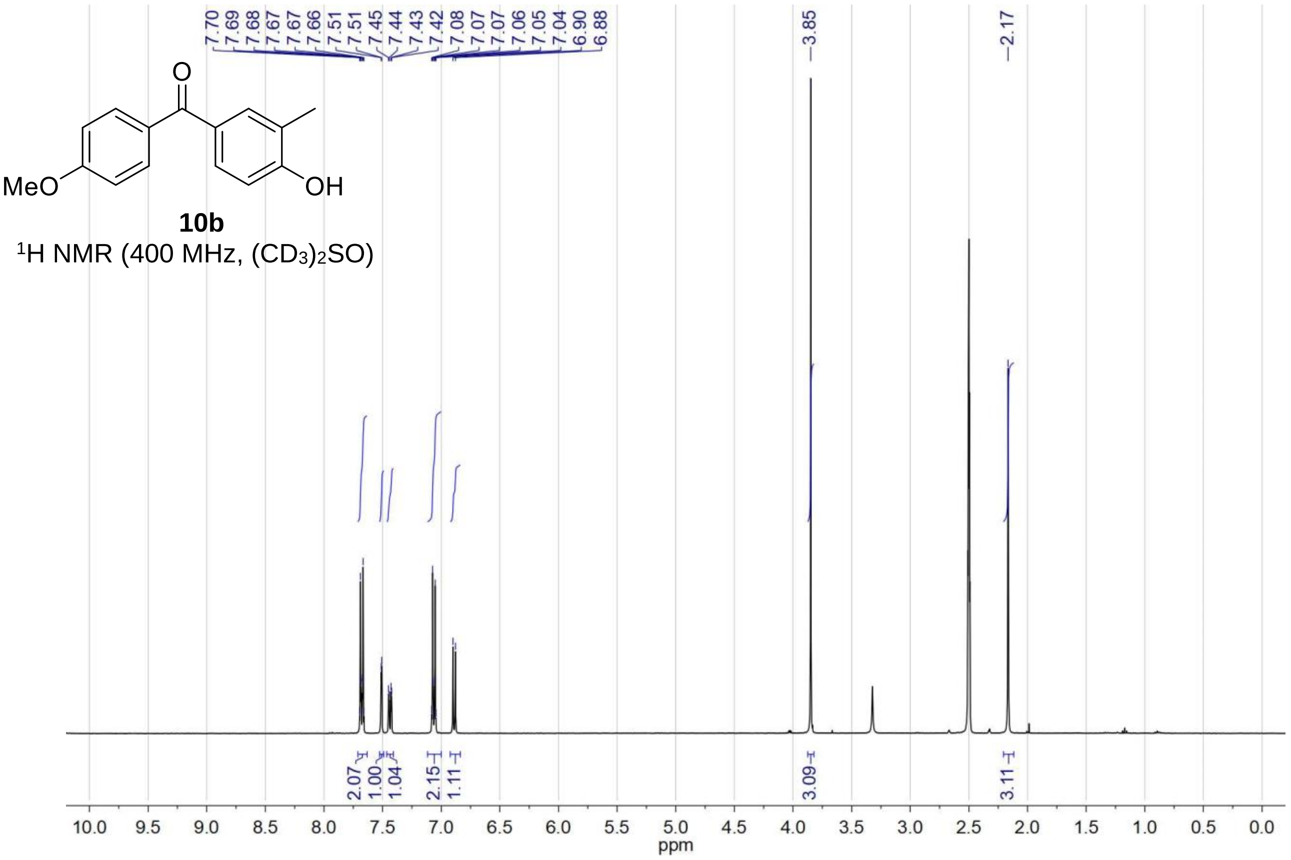

10b
^{13}C NMR (101 MHz, $(CD_3)_2SO$)

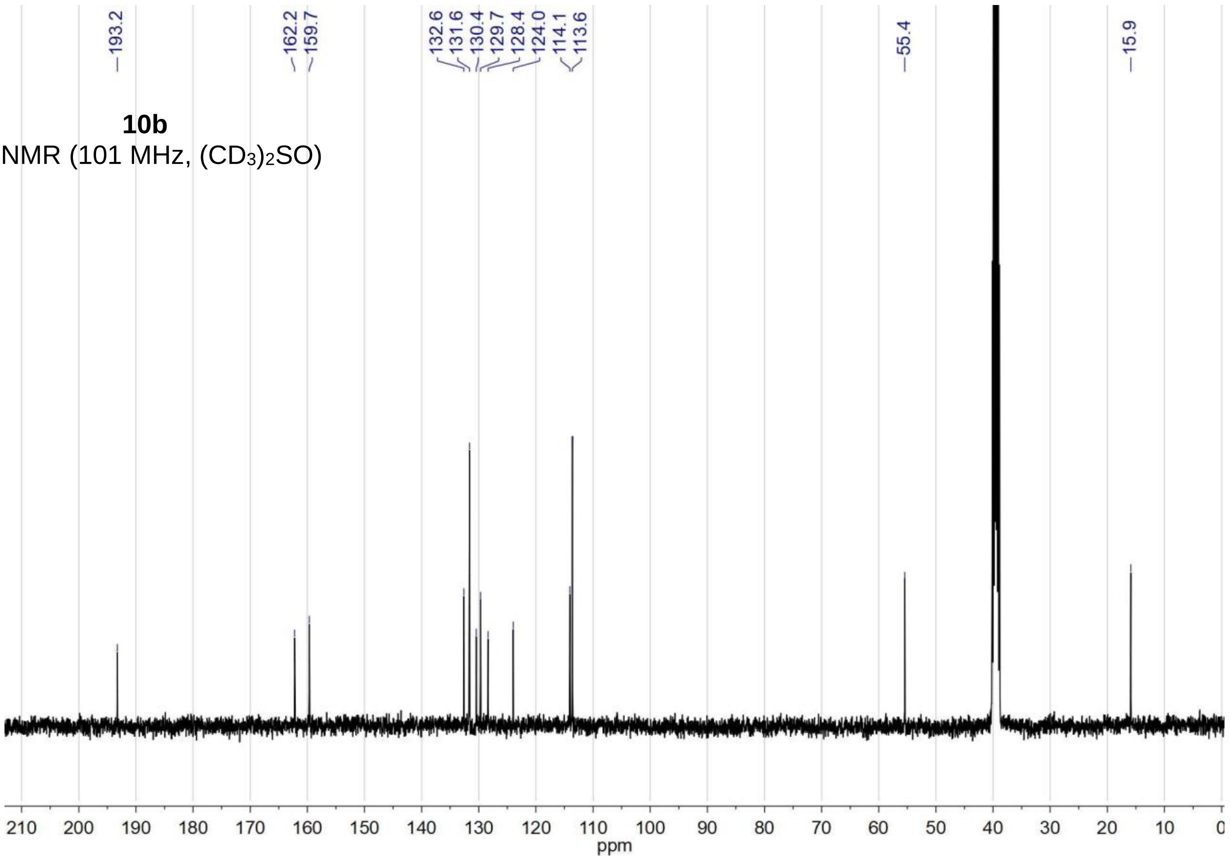

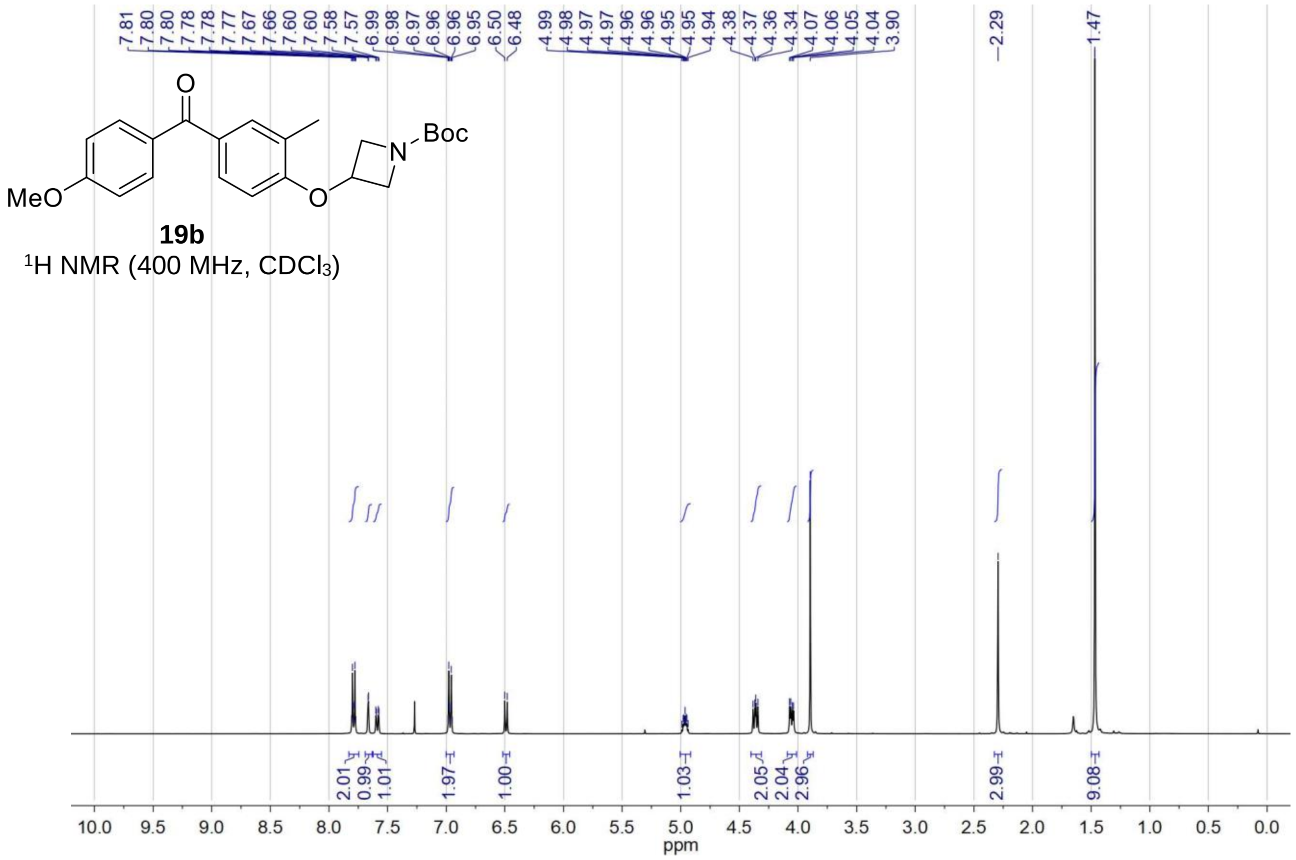

19b

^{1}H NMR (400 MHz, $CDCl_3$)

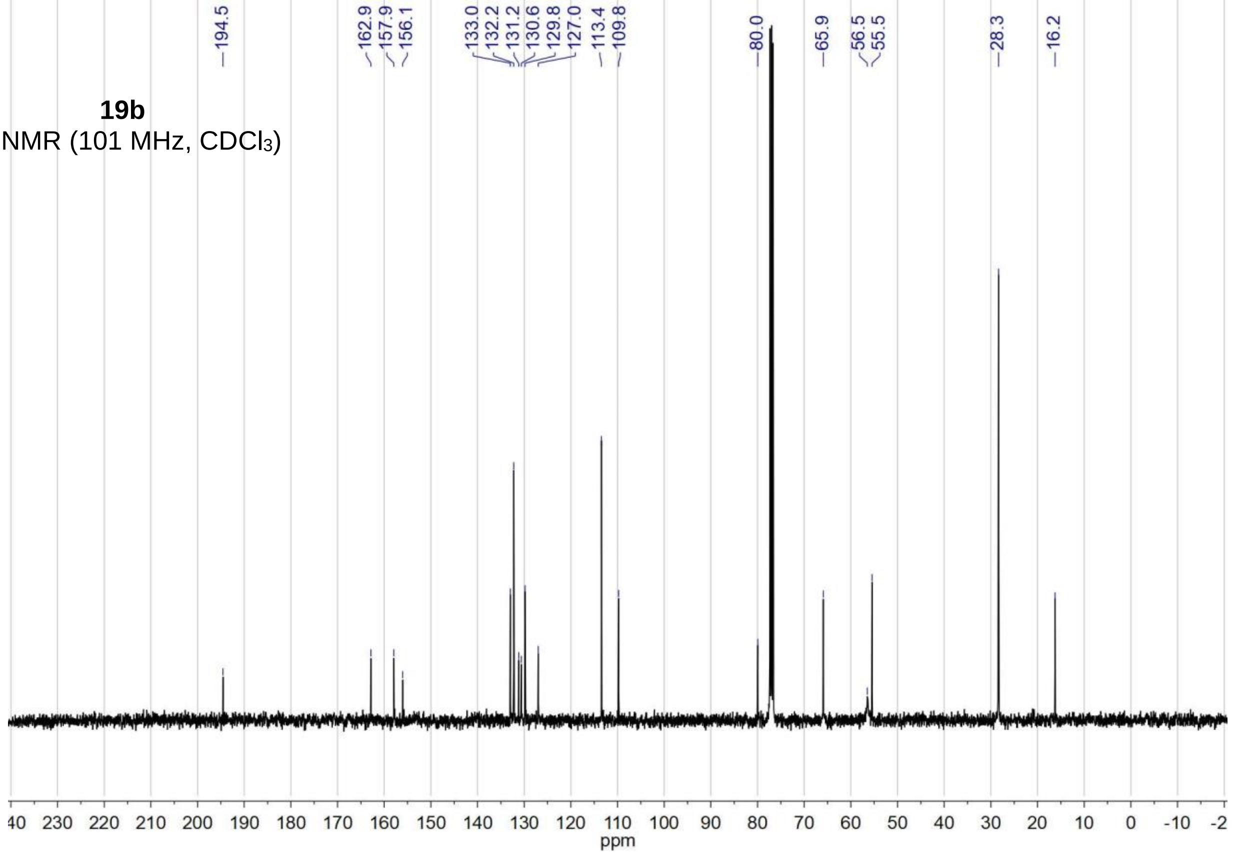

19b

^{13}C NMR (101 MHz, $CDCl_3$)

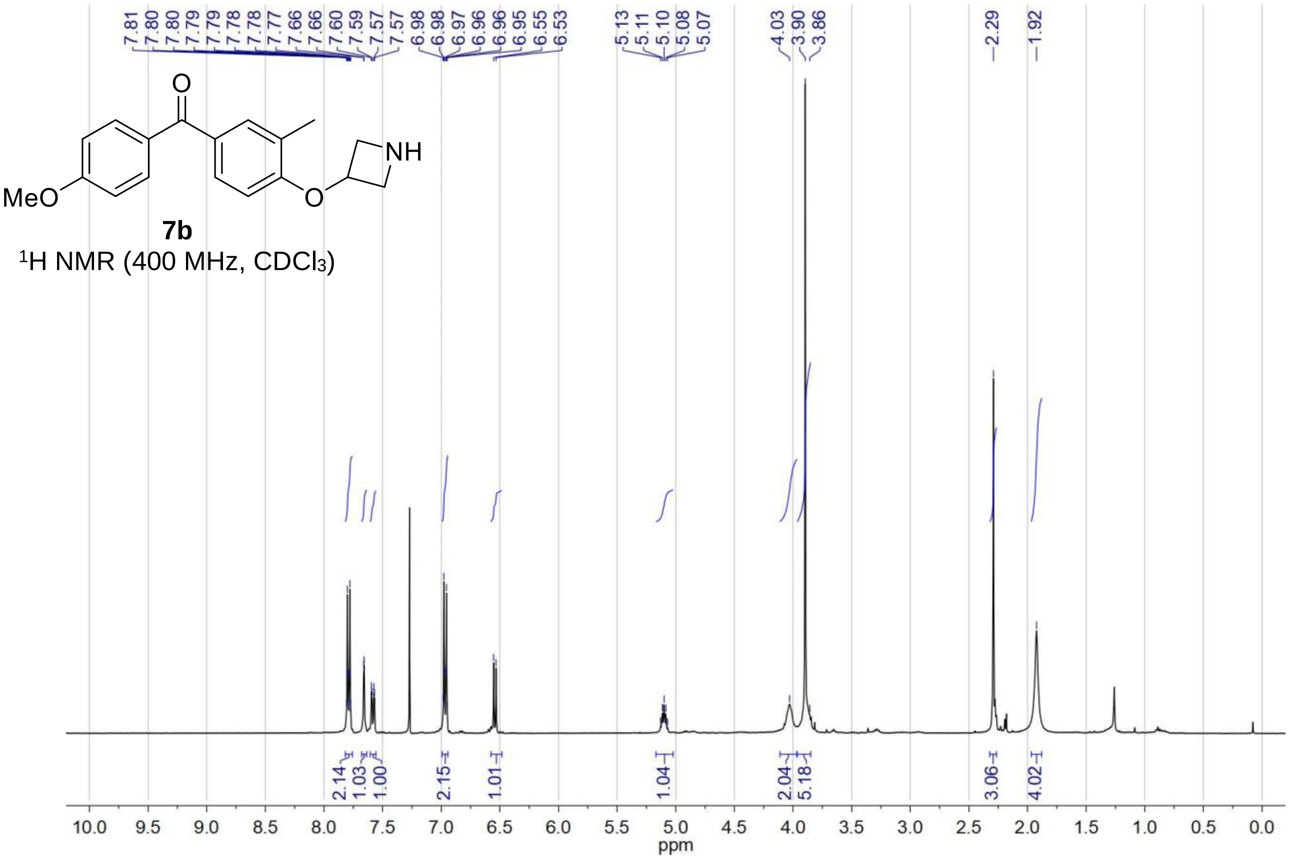

7b

^{1}H NMR (400 MHz, $CDCl_3$)

7b

^{13}C NMR (101 MHz, $CDCl_3$)

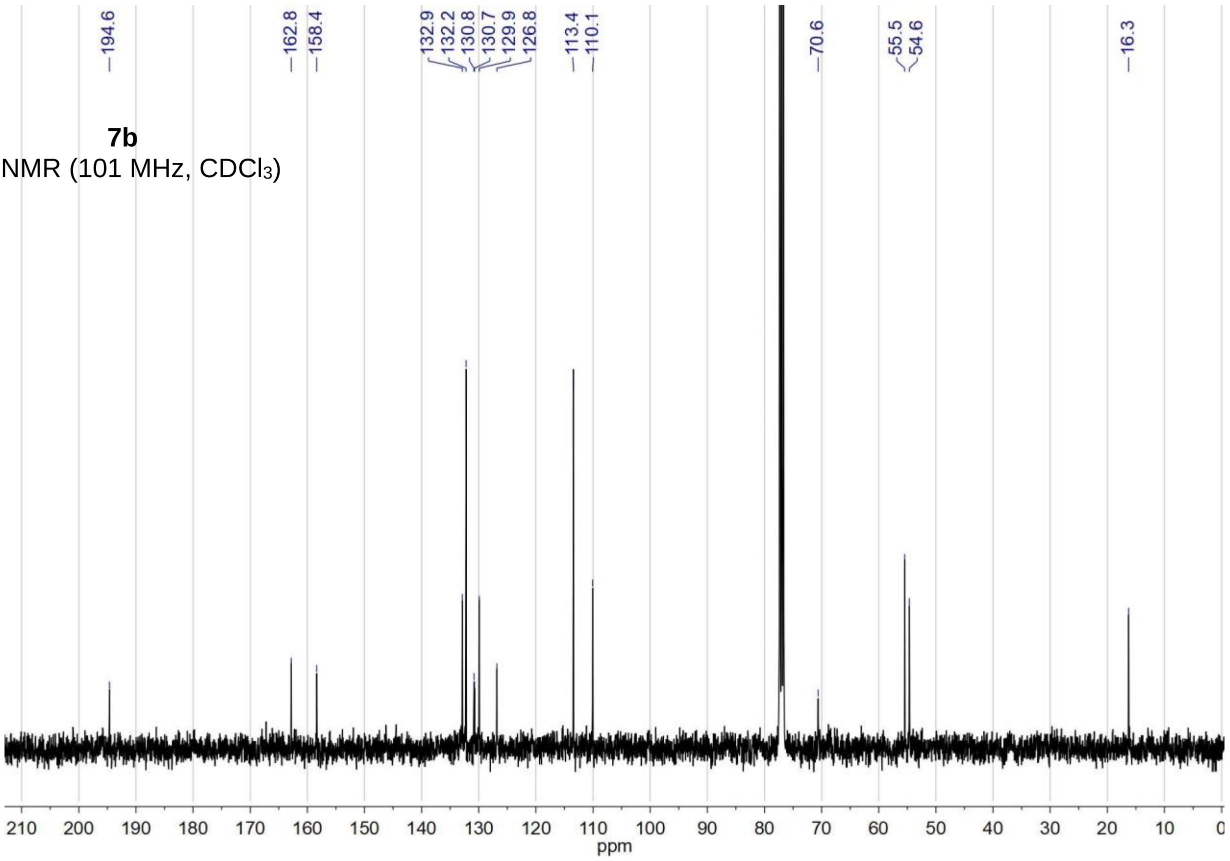

20b

^{1}H NMR (400 MHz, $CDCl_3$)

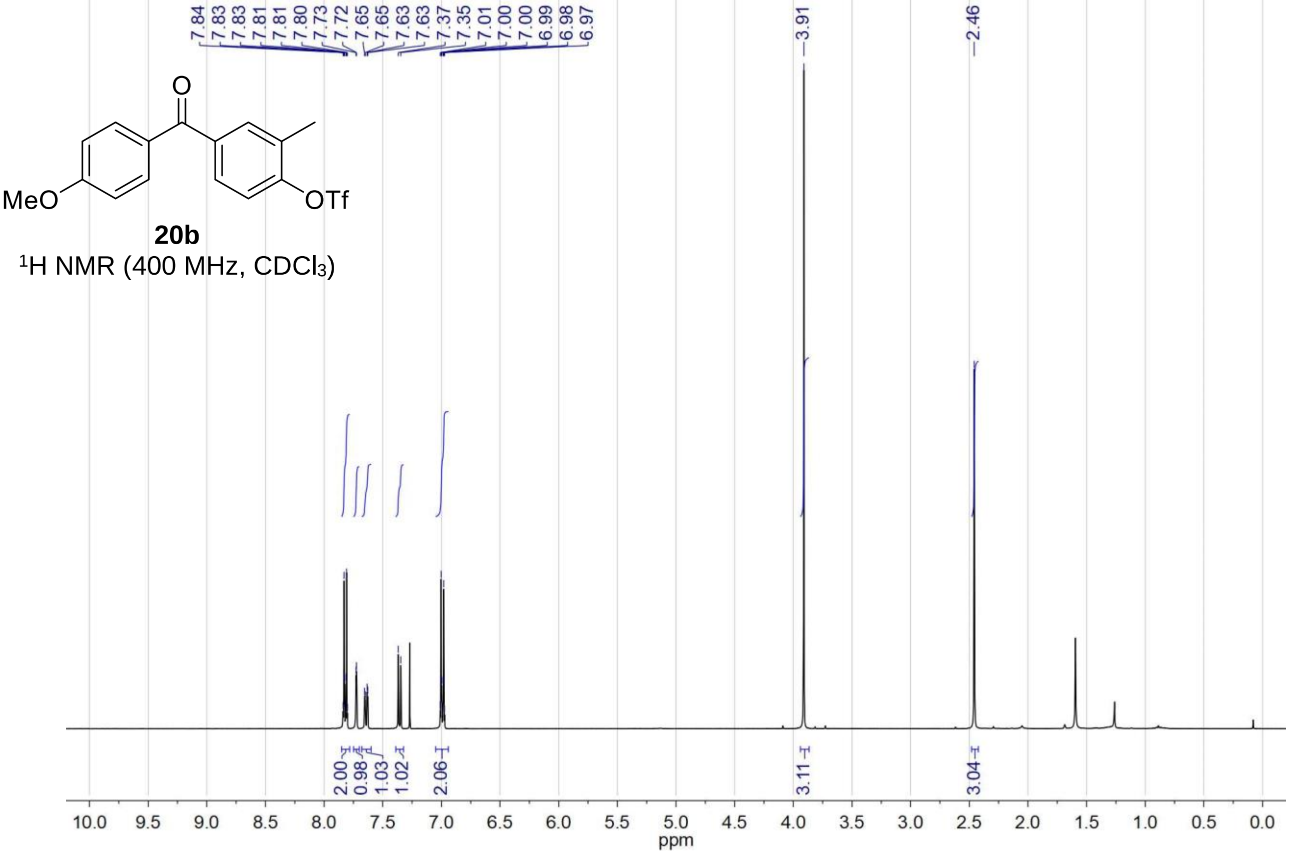

20b

^{13}C NMR (101 MHz, $CDCl_3$)

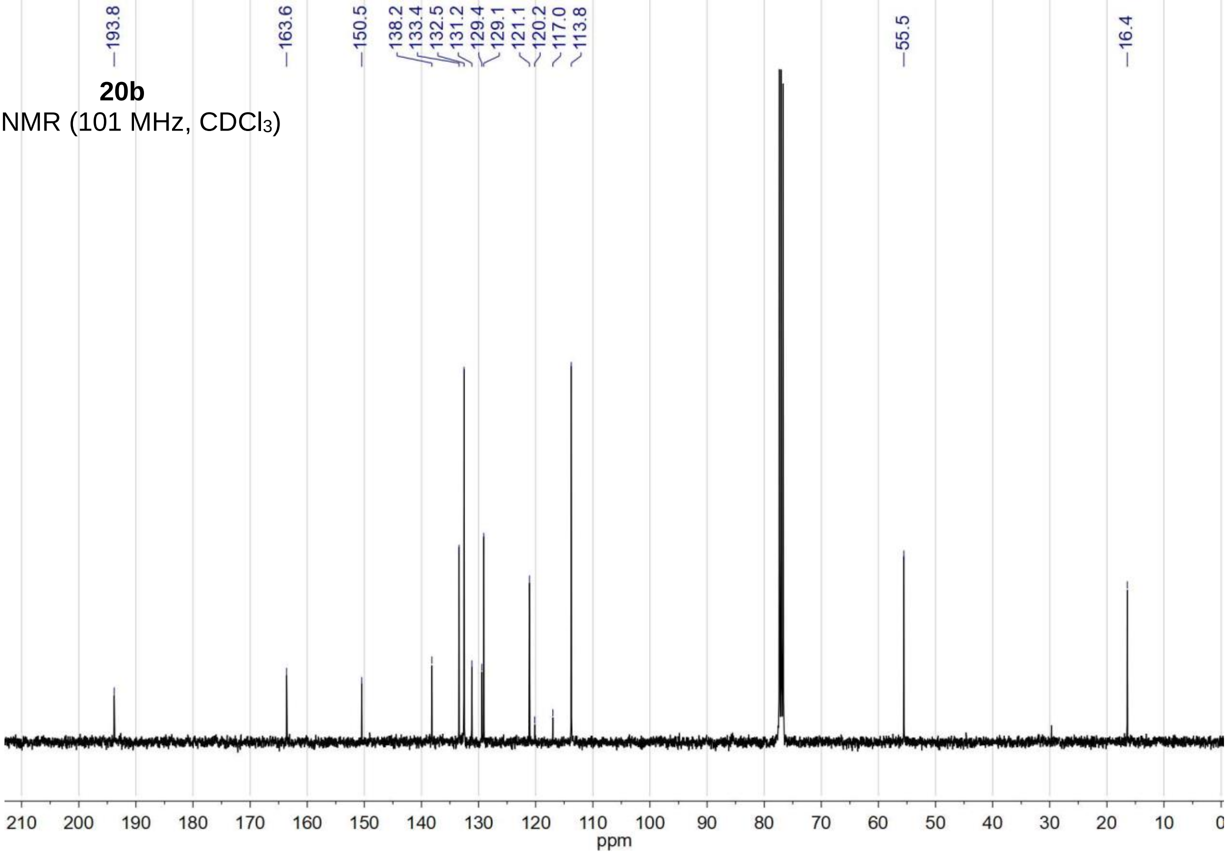

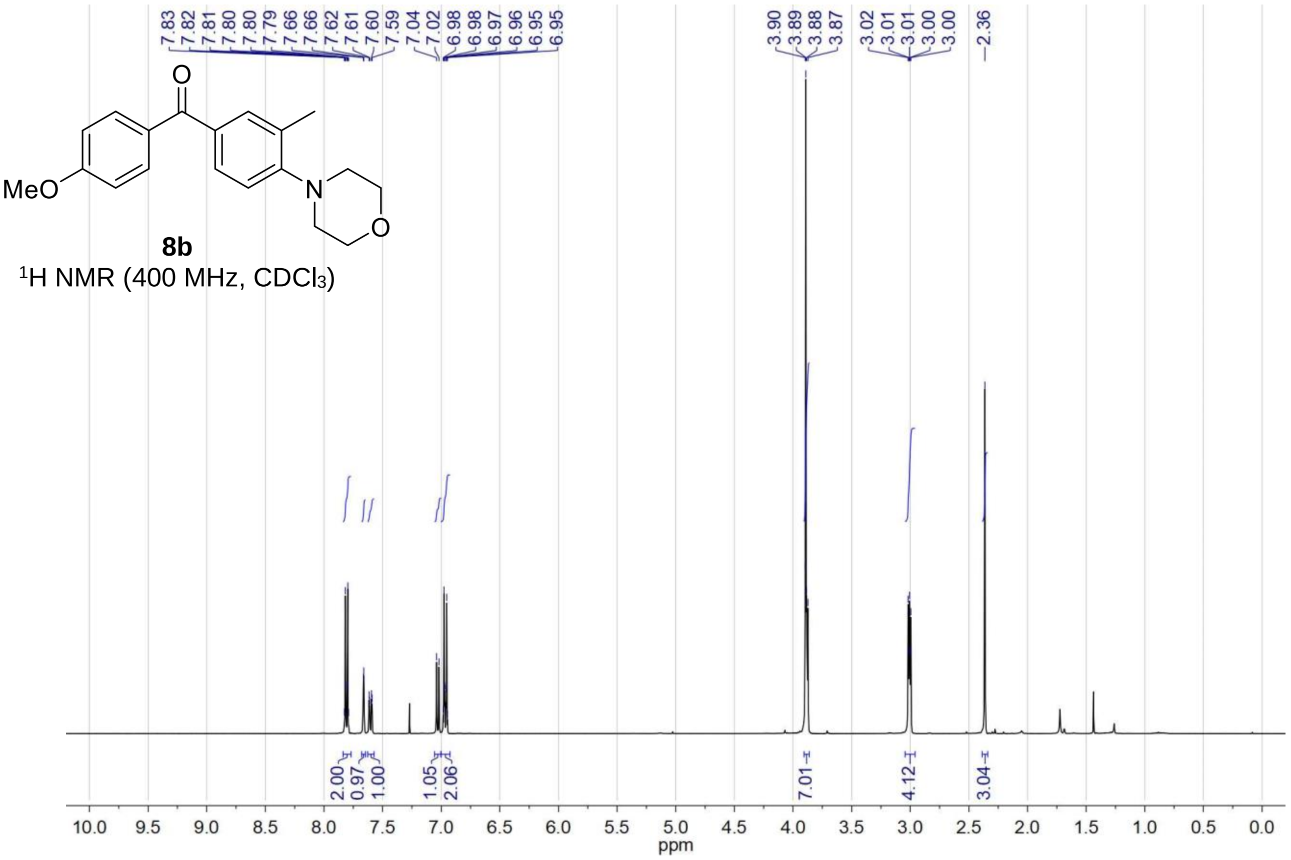

8b

^{1}H NMR (400 MHz, $CDCl_3$)

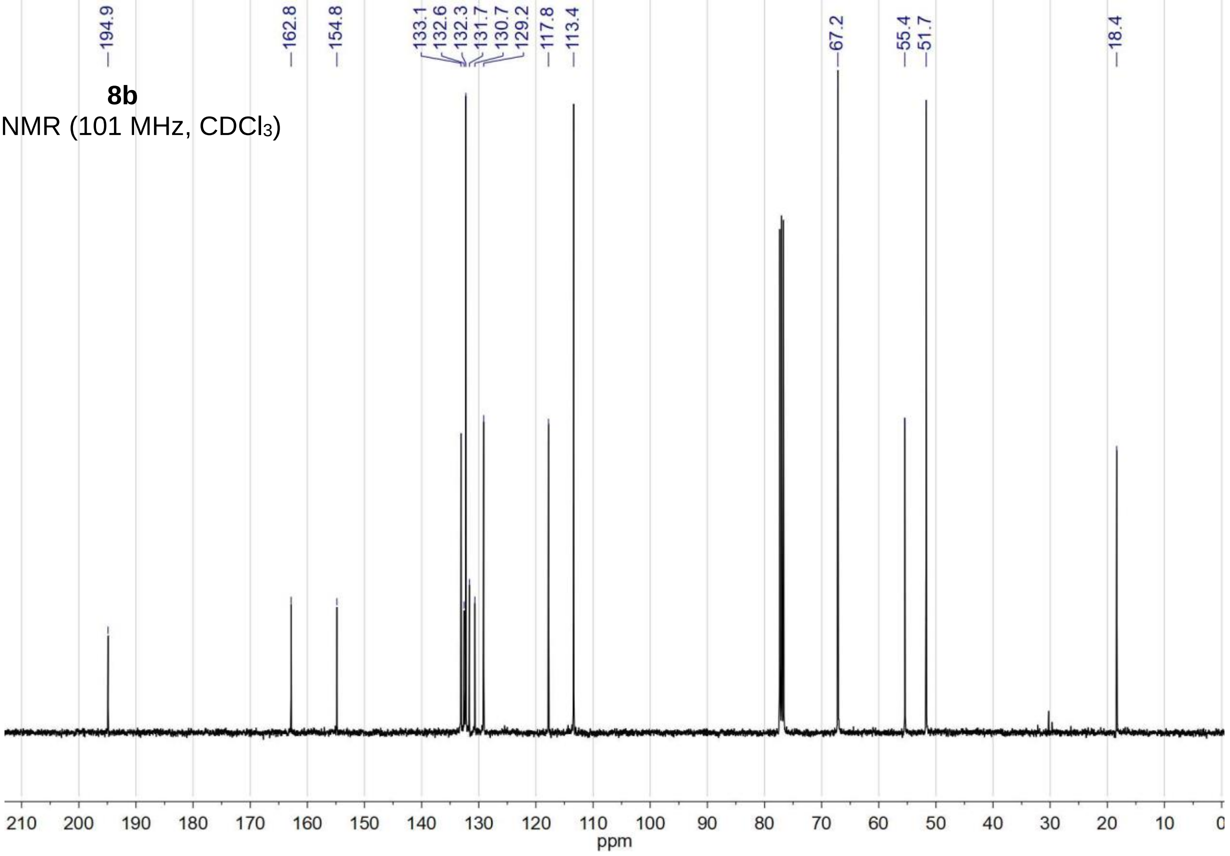

8b

^{13}C NMR (101 MHz, $CDCl_3$)

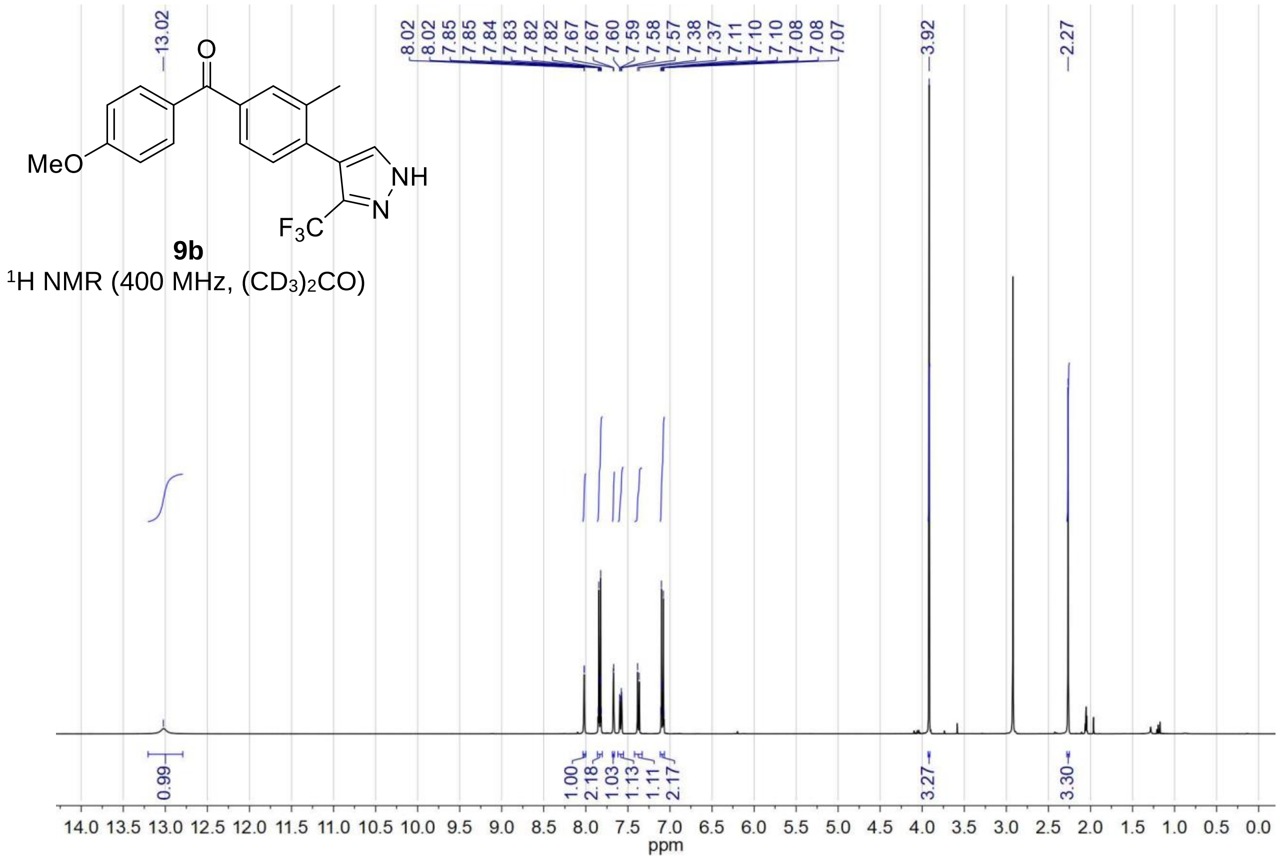

9b

^{1}H NMR (400 MHz, $(CD_3)_2CO$)

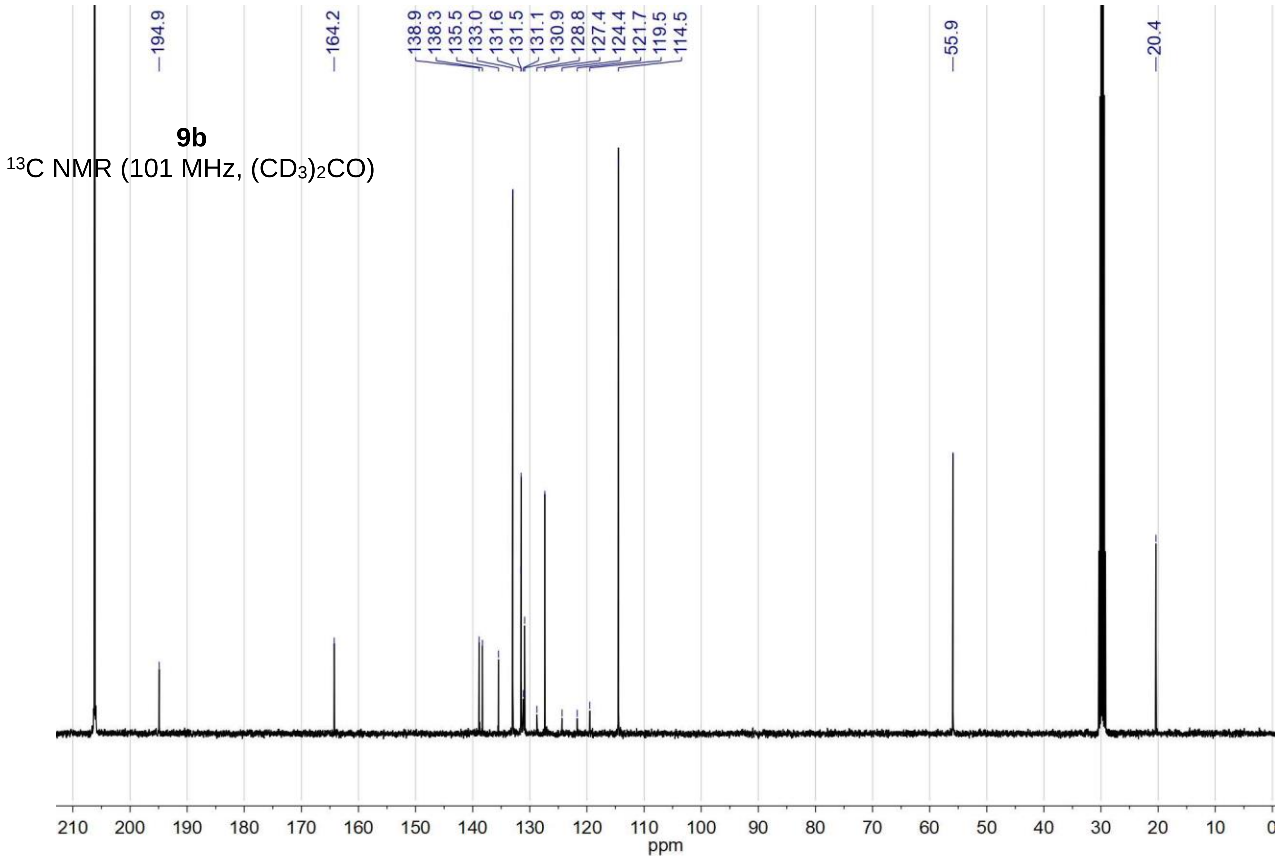

9b

^{13}C NMR (101 MHz, $(CD_3)_2CO$)

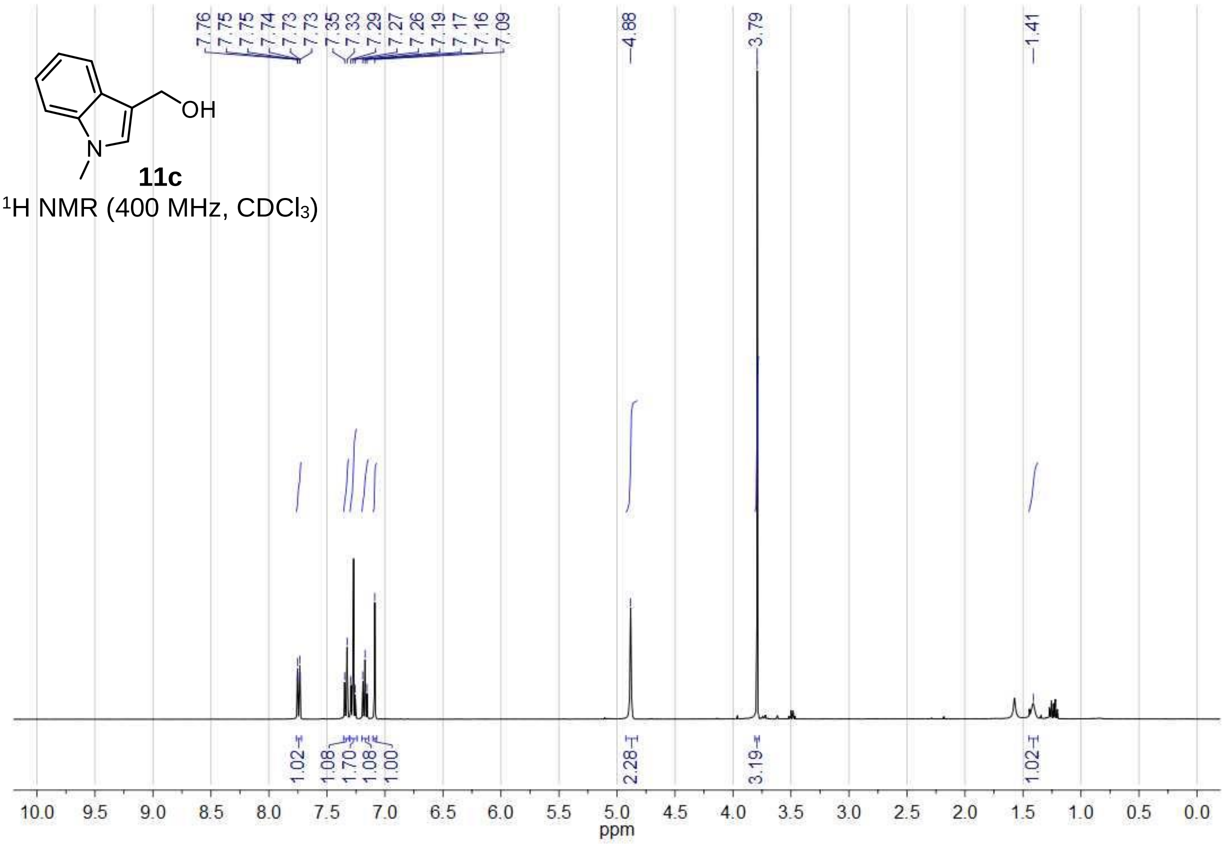

11c

^{1}H NMR (400 MHz, $CDCl_3$)

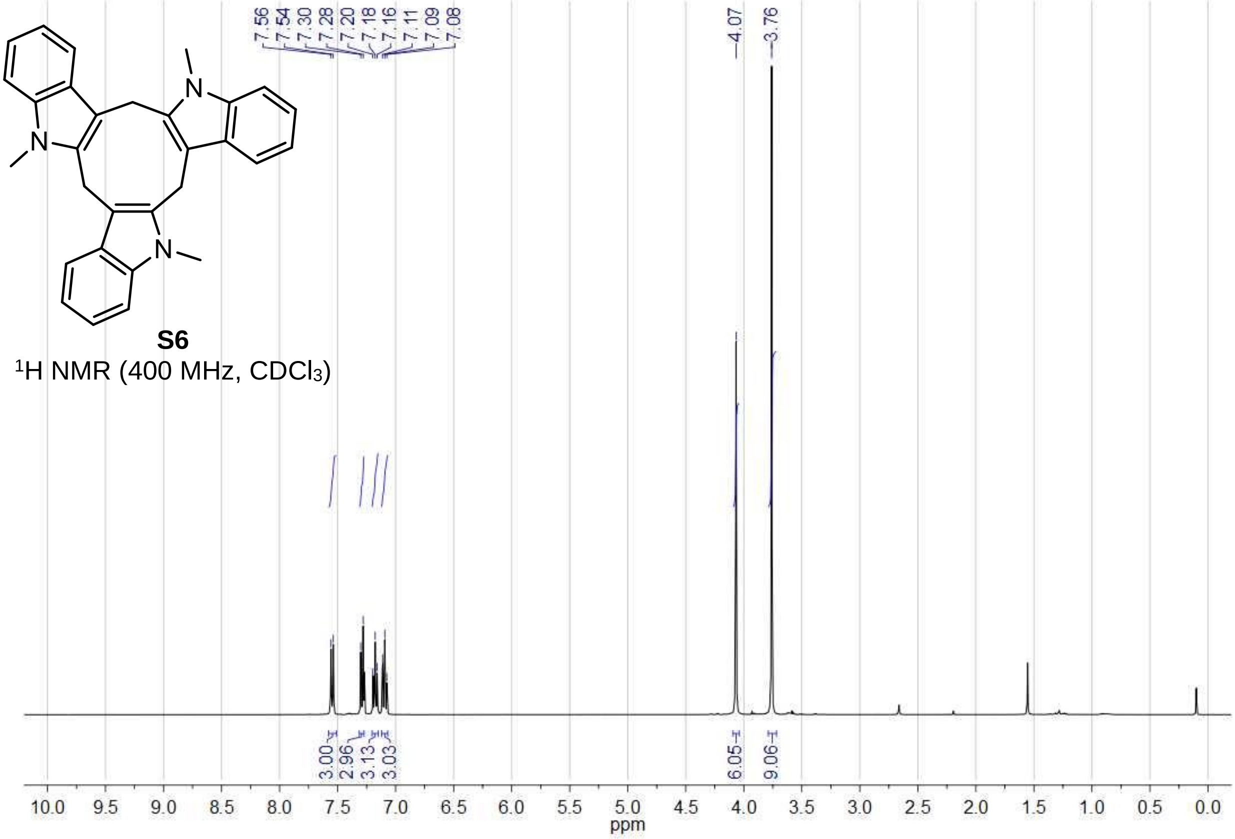

S6

^{1}H NMR (400 MHz, $CDCl_3$)

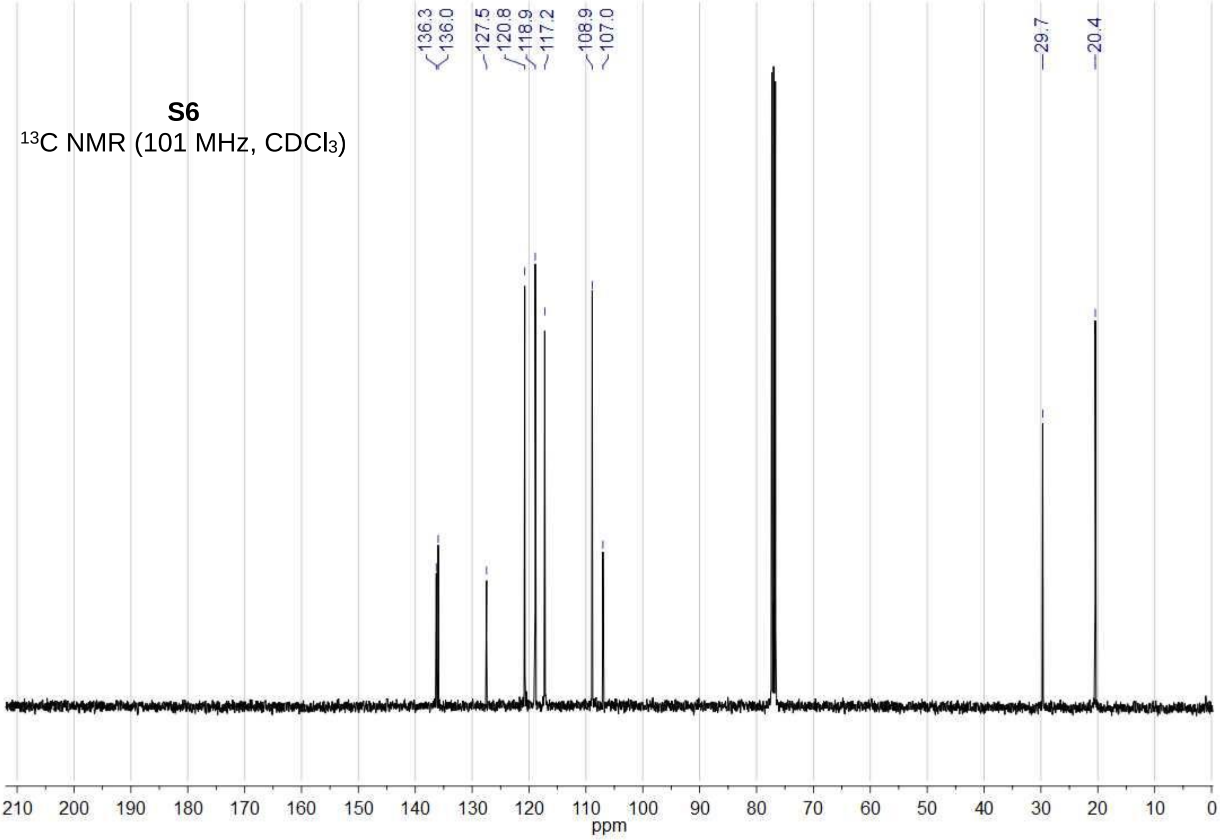

S6

^{13}C NMR (101 MHz, $CDCl_3$)

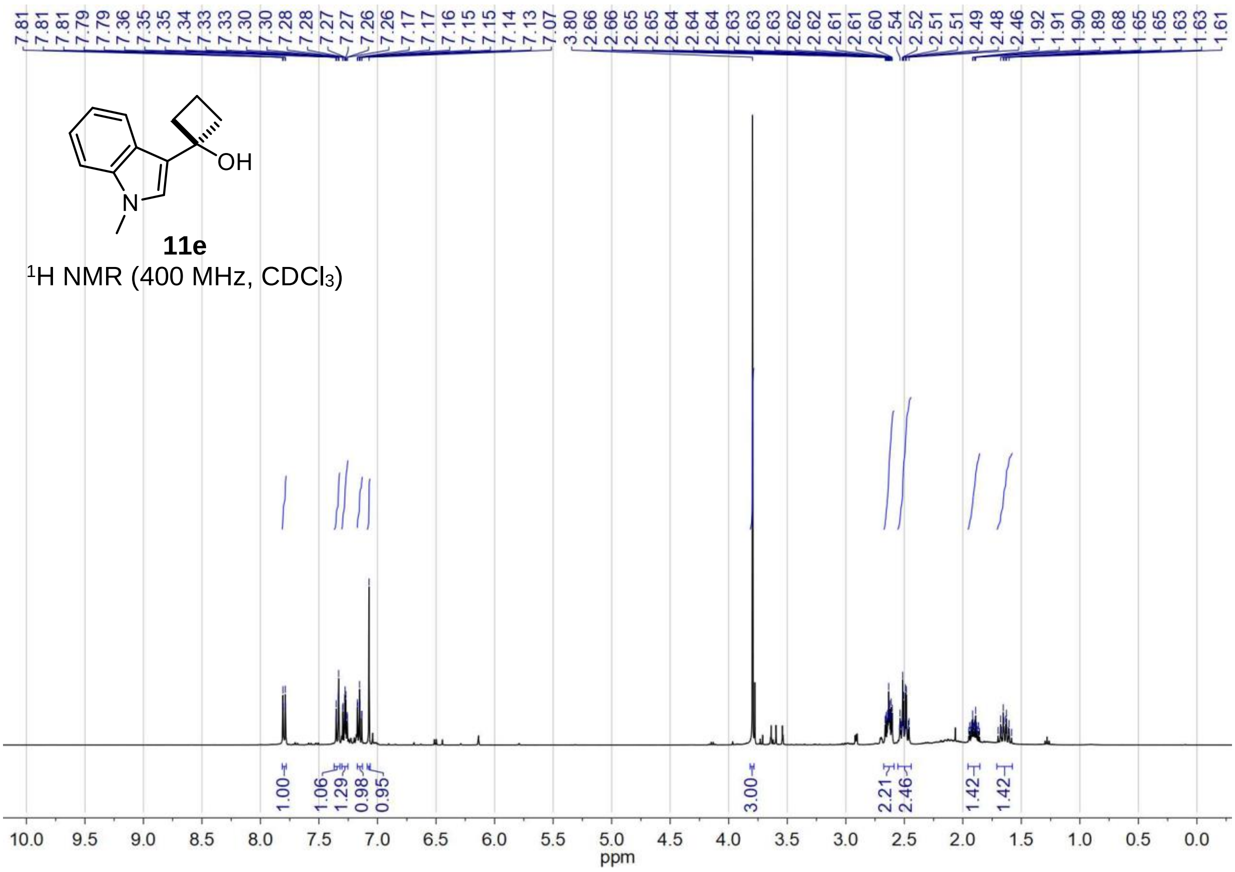

OH
N
11e
1H NMR (400 MHz, CDCl3)
ppm

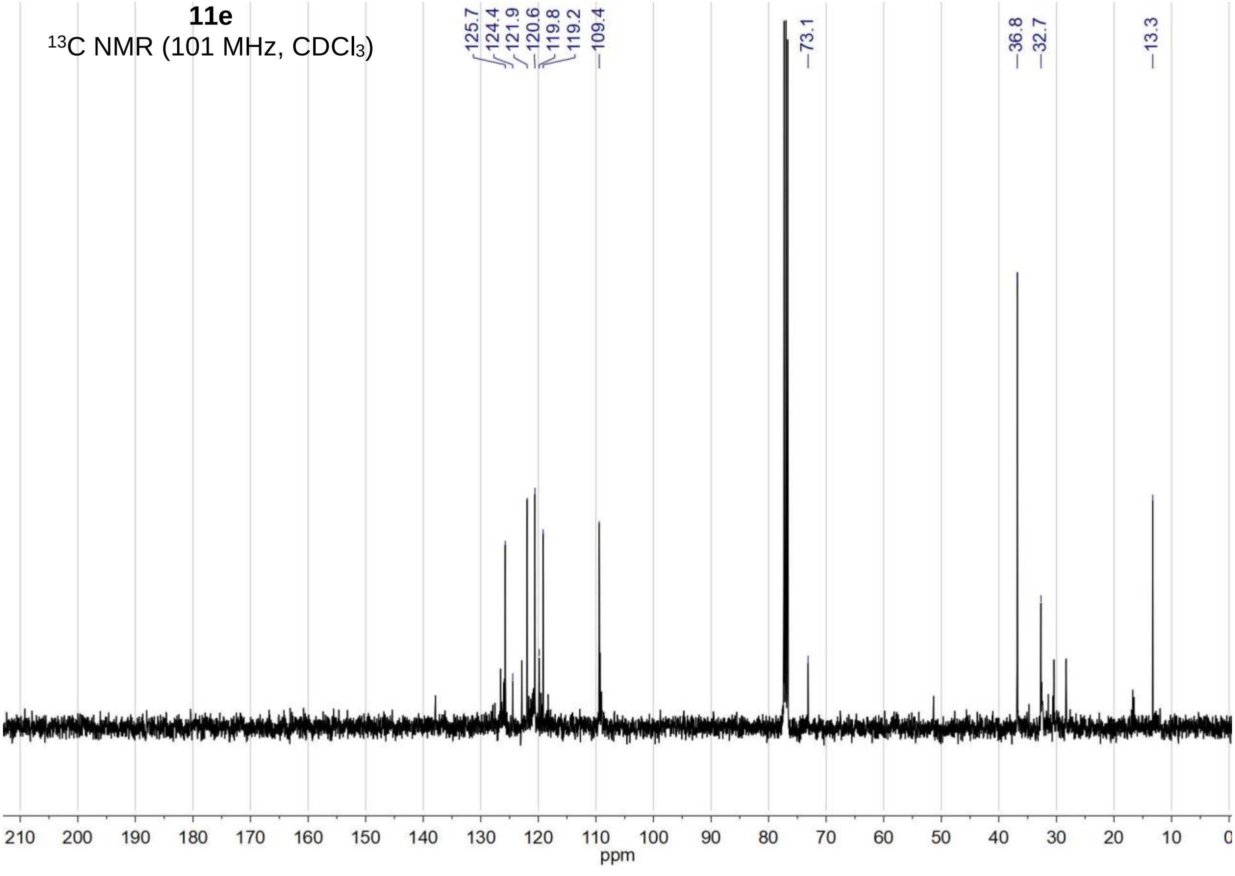

11e
13C NMR (101 MHz, CDCl3)
125.7
124.4
121.9
120.6
119.8
119.2
109.4
73.1
36.8
32.7
13.3
ppm

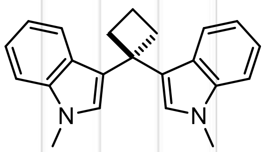

S7

^{1}H NMR (400 MHz, $CDCl_3$)

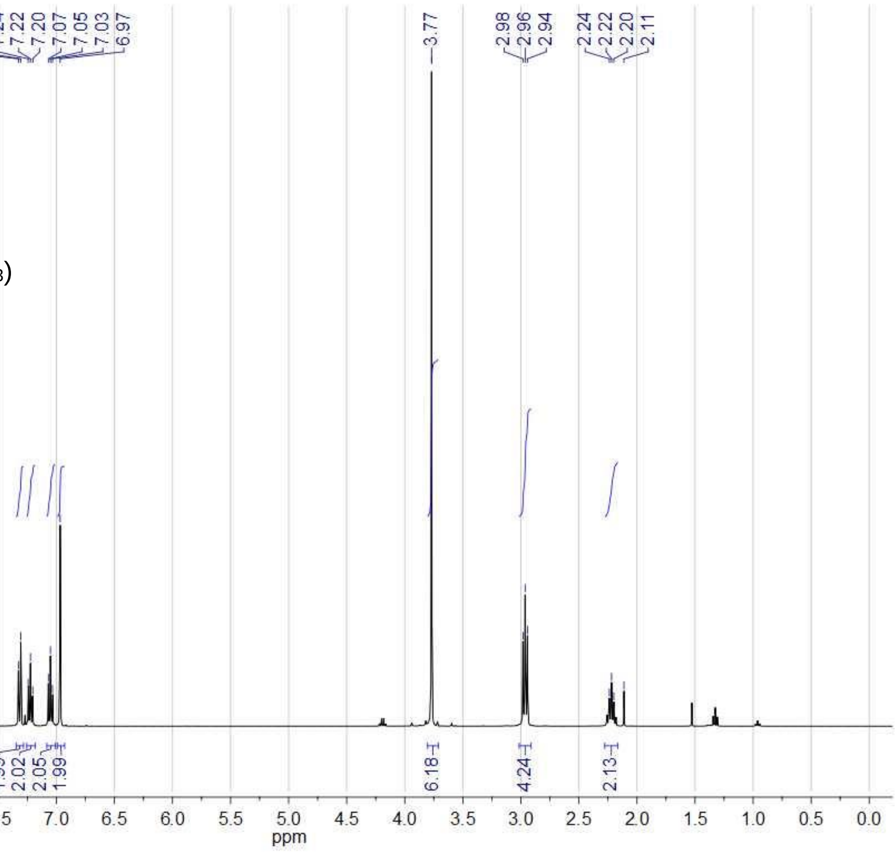

S7

^{13}C NMR (101 MHz, $CDCl_3$)

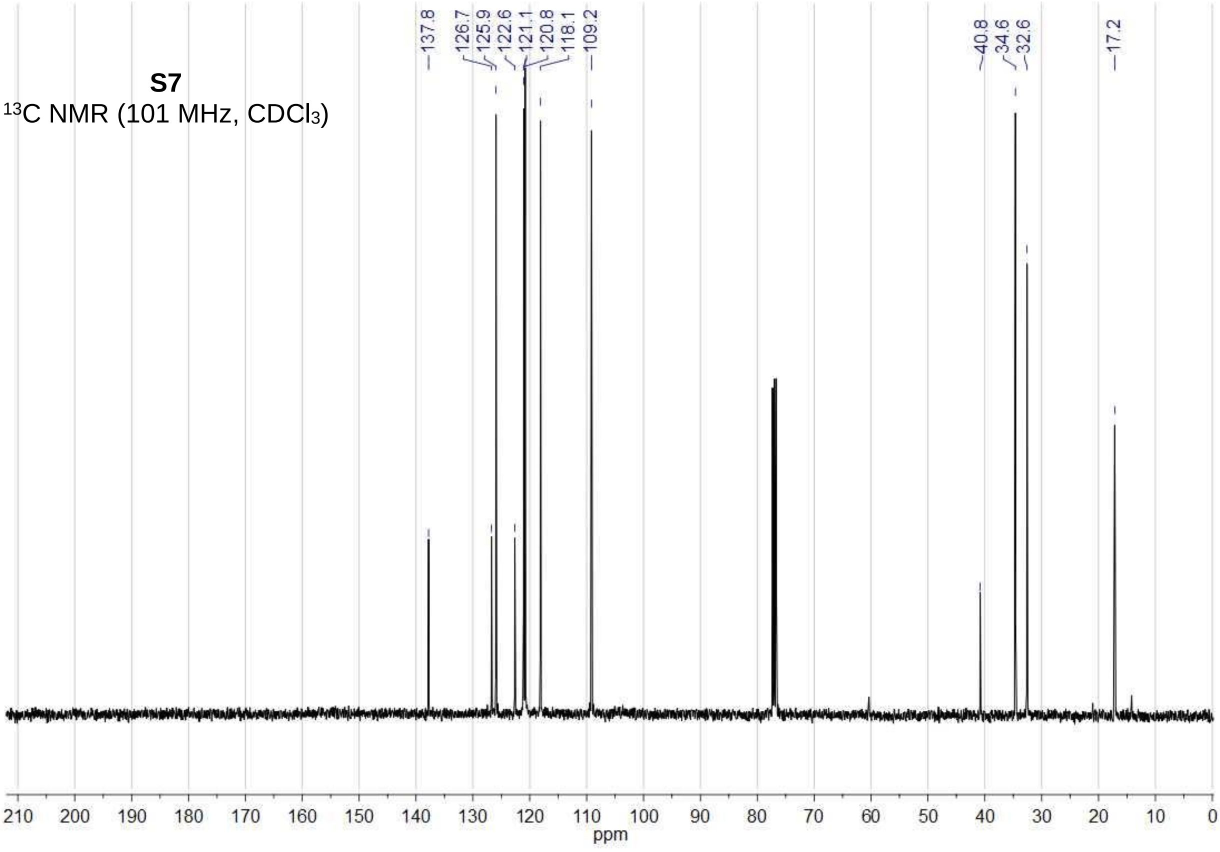

References

1 Perry, G. J. P.; Quibell, J. M.; Panigrahi, A.; Larrosa, I. Transition-Metal-Free Decarboxylative Iodination: New Routes for Decarboxylative Oxidative Cross-Couplings. *J. Am. Chem. Soc*. **2017**, *139*, 11527–11536.

2 Poppe, M.; Chen, C.; Liu, F.; Poppe, S.; Tschierske, C. Formation of a Cubic Liquid Crystalline Nanostructure with π-Conjugated Fluorinated Rods on the Gyroid Minimal Surface. *Chem. Eur. J.* **2017**, *23*, 7196–7200.

3 Beswick, P. J.; Harling, J. D.; Kleanthous, S.; Patel, V. K.; Simpson, J.; *Propionic acid derivatives and their use as HPPARS activators*. WO2004/000762 A3, **2003**.

4 Maiti, D.; Buchwald, S. L. Cu-Catalyzed Arylation of Phenols: Synthesis of Sterically Hindered and Heteroaryl Diaryl Ethers. *J. Org. Chem.*, **2010**, *75*, 1791–1794.

5 Croft, R. A.; Mousseau, J. J.; Choi, C.; Bull, J. A. Structurally Divergent Lithium Catalyzed Friedel–Crafts Reactions on Oxetan-3-ols: Synthesis of 3,3-Diaryloxetanes and 2,3-Dihydrobenzofurans. *Chem. Eur. J.* **2016**, *22*, 16271–16276.

6 Åhman, J.; Buchwald, S. L. An improved method for the palladium-catalyzed amination of aryl triflates. *Tetrahedron Lett.* **1997**, *38*, 6363–6366.

7 Jammula, S. R.; Anna, V. R.; Tatina, S.; Krishna, T.; Sreenivas, B. Y.; Pal, M. A new strategy for accessing (S)-1-(furan-2-yl) pent-4-en-1-ol: a key precursor of Ipomoeassin family of compounds and C1–C15 domain of halichondrins. *Tetrahedron Lett*., **2016**, *57*, 3924–3928.

8 Leete, E. 3-Hydroxymethylindoles. *J. Am. Chem. Soc.* **1959**, *81*, 6023–6026.

9 Santoso, M.; Somphol, K.; Kumar, N.; Black, D. S. Synthesis of indolocyclotriveratrylenes. *Tetrahedron* **2009**, *65*, 5977–5983.

10 Yan, J.; Ni, T.; Yan, F. Simple and efficient procedures for selective preparation of 3-haloindoles and 2,3-dihaloindoles by using 1,3-dibromo-5,5-dimethylhydantoin and 1,3-dichloro-5,5-dimethylhydantoin. *Tetrahedron Lett.* **2015**, *56*, 1096–1098.

11 Di, L.; Whitney - Pickett, C.; Umland, J. P.; Zhang, H.; Zhang, X.; Gebhard, D. F.; Lai, Y.; Federico, J. J.; Davidson, R. E.; Smith, R.; Reyner, E. L.; Lee, C.; Feng, B.; Rotter, C.; Varma, M. V.; Kempshall, S.; Fenner, K.; El - kattan, A. F.; Liston, T. E.; Troutman, M. D. Development of a new permeability assay using low-efflux MDCKII cells. *J. Pharm. Sci*. **2011**, *100*, 4974–4985.
